# Tuning Connectivity in a Three-Component Assembly of Metal–Organic Cage-Cross-Linked Polymer Networks

**DOI:** 10.1021/acs.macromol.5c02021

**Published:** 2025-10-22

**Authors:** Mostafa Ahmadi, Josep Duran, Albert Poater

**Affiliations:** † Department of Chemistry, 9182 Johannes Gutenberg-Universität Mainz, Duesbergweg 10−14, Mainz D-55128, Germany; ‡ Institut de Química Computacional I Catàlisi, Departament de Química, 117394 Universitat de Girona, C/ M. Aurèlia Capmany, 69, Girona, Catalonia 17003, Spain

## Abstract

Network connectivity strongly influences the dynamics and mechanical properties of materials such as natural tissues and hydrogels, which are known for their adaptability and self-healing. Metal–organic cages (MOCs), with modular structures and reversible coordination, provide a versatile platform to engineer connectivity in polymer networks. Here, we use an octahedral MOC to form transient poly­(ethylene glycol) (PEG)-based hydrogels and investigate their viscoelastic behavior by varying the junction functionality and polymer architecture. A distinct low-frequency relaxation mode emerges after annealing, reflecting the interplay between cage formation and a mixture of homo- and heteroleptic metal complexes. Cage formation is undermined at both high and low polymer concentrations due to steric hindrance and chain overstretching, respectively. At an optimal polymer concentration, reducing cage content preserves the cage integrity and reveals a transition from phantom to affine network behavior. In contrast, replacing polymeric ligands with small-molecule equivalents results in misconnectivity and a lower modulus. Kinetics analysis at the microscale using Fluorescence Resonance Energy Transfer (FRET) shows that incorporation into polymer networks destabilizes the cage, likely due to chain dynamics. DFT calculations further reveal that only Pd^2+^, among several tested transition metal ions, provides the appropriate coordination environment and bond stability for robust cage formation. Despite this, the high junction functionality enables rapid and efficient self-healing. This work examines how tuning connectivity in transient networks can guide the design of materials with tailored properties such as recyclability, self-healing, and stimuli-responsiveness.

## Graphical abstract

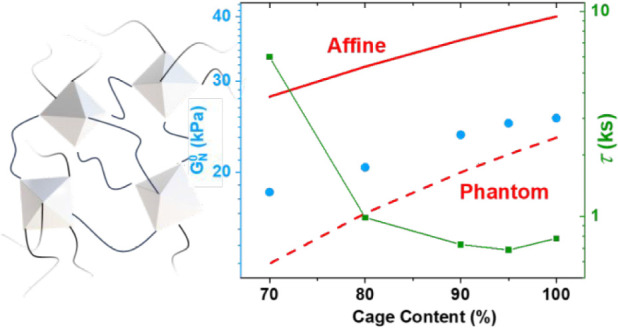

## 1 Introduction

Polymer network connectivity is a critical factor that defines many of its properties.^1^ Specifically, the viscoelastic properties, swelling behavior, stimuli-responsiveness, and self-healing of polymer networks are closely linked to the network architecture and connectivity.^2–5^ In traditional covalently cross-linked polymer networks, connectivity is fixed, leading to the entrapment of network heterogeneities and defects.^6,7^ In contrast, transiently cross-linked networks feature dynamic connectivity, enabling self-correction toward thermodynamically favorable configurations.^8^ As such, engineering connectivity in transient polymer networks has emerged as a promising route to develop new soft materials with defined structure and dynamics for advanced applications such as robotics and medicine.

The functionality or connectivity of transient junctions is a central design parameter in dynamic networks.^5,9^ Enhancing cross-link functionality has attracted significant interest due to its impact on network architecture and material performance. One important yet relatively underexplored aspect is how increased connectivity influences the self-healing behavior of transient polymer networks. In a seminal study, Leibler and coworkers developed supramolecular networks based on multifunctional hydrogen-bonding amide and urea groups.^10^ The outstanding self-healing of the resulting networks was rationalized by the high mobility of functional groups due to arrested crystallization and the high mutual accessibility of supramolecular bonds.^11^ Similarly, dendritic polymers with positively charged terminal groups in combination with negatively charged laponite nanoparticles produce self-healable hydrogels, presumably due to the high surface area of the nanoparticles and the high concentration of terminal groups in the hyperbranched polymer precursor.^12^ Likewise, good self-healing properties are reported for polymer networks that transiently interact with embedded nanoparticles.^13–15^ We have recently demonstrated that adding a small fraction of hyperbranched polymer with supramolecular terminal groups to a model-type transient polymer network enhances self-healing by increasing the mutual accessibility of supramolecular bonds, facilitating more effective exchange reactions.^3^ These findings emphasize that connectivity plays a crucial role in enhancing various functional properties of transient polymer networks, specifically those reliant on the exchange of supramolecular bonds, like self-healing and recycling.^16,17^

Among the wide range of supramolecular interactions, metallo-supramolecular bonds stand out for their ability to systematically vary junction connectivity.^9,18,19^ Besides the node-like metal complex junctions, whose connectivity can be controlled by the denticity of the ligand and the coordination geometry preference of the metal ion, recent advances have enabled network junctions with larger nuclearity. Specifically, two- and three-dimensional junctions, respectively formed by metallacycles and metal–organic cages (MOCs), provide a novel strategy for modular variation of cross-link functionality in a wide range.^20–22^ MOCs are supramolecular complexes formed by coordination-driven assembly of multitopic ligands and metal ions, which are characterized by their well-defined geometry, regular shape, and nanoscopic cavity size.^23^ The characteristics of MOCs can be rationally controlled by design parameters, for instance, by the coordination geometry of the selected metal ion, and the length and bend angle of the curved ligands.^23,24^ Accordingly, Fujita’s cages formed by bis-4-pyridyl ligands and Pd^2+^ metal ions can contain from 2 up to 24 metal ions upon increasing the angle between donor pyridyl groups.^23,25–27^ Such regularly shaped structures have potential for absorption and transport of cargos mainly by hydrophobic effects and their release upon disassembly. Moreover, the internal chemical space of the cage cavity can be manipulated to provide effective interactions with the cargo, resulting in selective absorption of a guest^28^ or even catalyzing specific chemical reactions.^29^ A prime example is the “plug-and-play” concept developed by Johnson and coworkers, where a fraction of bispyridyl ligands was endohedrally functionalized by various catalytic groups, whose simple mixing with unfunctionalized ligands results in cages with new catalytic abilities.^30^ On top of that, the chemical design of the ligands or the cargo, besides the reversibility of metal coordination bonds, can provide stimuli-responsive cage assemblies, so that a cargo can be selectively absorbed, transported, and released on demand or a reaction can either occur or be halted upon the application of an external stimulus.^28,31–33^ A well-known example is provided by Clever’s group, where the photoinduced cyclization of a diarylethene group placed along the bispyridyl ligand’s backbone can reversibly change the ligand’s bend angle, and accordingly, the cage size.^34,35^ MOCs can be actively incorporated inside polymer networks, as multifunctional cross-linking points, by replacing small-molecule ligands with telechelic or side-chain functionalized polymeric ligands.^20,21^ For instance, Johnson et al. have functionalized tetra-arm poly­(ethylene glycol) (tetraPEG) chains with bispyridyl ligands and developed star polyMOC gels upon forming cross-links by assembly of MOCs.^36^ However, ill-defined cages were reported to form in crowded networks with high-functionality junctions.^36^ Accordingly, many sophisticated polymer structures with advanced applications have been developed based on MOC cross-linked networks.^37–39^ However, fundamental studies aiming at a deeper understanding of these systems are still lacking.^40^

To address this knowledge gap, we present a detailed study of MOC-cross-linked polymer networks designed to tune junction functionality beyond the limits of conventional metal–ligand systems. For this, we use an octahedral MOC with six auxiliary ligands that can be connected by long PEG chains to provide polymer network hydrogels with 6-fold junctions. We systematically vary supra- and macromolecular parameters associated with the cage design and polymer precursor architecture to map out the landscape of potential network connectivity. Additionally, Density Functional Theory (DFT) simulations provide insight into the energetic and structural factors governing the cage stability and integration. We use such microscopic measures of the cage structure and stability to explain the macroscopic structure and dynamics of hydrogels, as measured by rheology. Together, our findings lay the foundation for designing next-generation soft materials with programmable junction architecture, self-healing behavior, and stimuli-responsiveness.

## 2 Experimental Section

### 2.1 Materials

Linear PEG (*M*_w_ = 10 kg mol^–1^, PDI = 1.03), 5-chloro-1,10-phenanthroline (Cl-Phen), 5-amine-1,10-phenanthroline (Phen-NH_2_), and 4-chloropyridine (Cl-Py) were purchased from Sigma-Aldrich. 5/6-Carboxytetramethylrhodamine succinimidyl ester (NHS-Rhodamine) and 5/6-carboxyfluorescein succinimidyl ester (NHS-Fluorescein) were purchased from Thermo Fisher Scientific. TetraPEG (*M*_w_ = 20 kg mol^–1^, PDI = 1.03) was purchased from Creative PEGWorks. 2,4,6-Tri­(4-pyridyl)-1,3,5-triazine (TPT) was purchased from Fluorochem. All other chemicals used were commercially available and used without further modification. All used solvents were dry and sealed and purchased from Acros. Milli-Q distilled water was used for hydrogel formation.

### 2.2 Synthesis

#### 2.2.1 Polymer Precursors

Linear PEG was functionalized with Cl-Phen (LPhen10k) following a nucleophilic aromatic substitution (SNAr) reaction.^41^ PEG (10 kg mol^–1^, 1 g, 0.2 mmol of OH) was first dried under vacuum at 75 °C and then dissolved in dimethyl sulfoxide (DMSO, 20 mL). Potassium hydroxide (0.09 g, 1.6 mmol) was ground, dried under vacuum, added to the solution, and stirred at 60 °C for 90 min. Cl-Phen (0.09 g, 0.4 mmol) was subsequently added, and the mixture was stirred at 60 °C for 24 h. The reaction mixture was poured into 100 mL of brine, extracted with DCM (3 × 100 mL), dried over MgSO_4_, filtered, concentrated in a rotary evaporator, and precipitated in 200 mL of cold diethyl ether. The precipitate was filtered and dried to yield the product as a white solid (LPhen10k, 0.9 g, 90%). The functionalization degree (almost quantitative) was quantified by adding a known amount of 3-(trimethoxysilyl)­ethyl acrylate (TMS-HEA) as the external standard, as shown in Figure S1. ^1^H NMR (400 MHz, DMSO) δ 9.13 (dd, *J* = 4.3, 1.8 Hz, 1H), 8.92 (dd, *J* = 4.3, 1.7 Hz, 1H), 8.67 (dd, *J* = 8.3, 1.8 Hz, 1H), 8.34 (dd, *J* = 8.2, 1.7 Hz, 1H), 7.84–7.59 (m, 2H), 7.38 (s, 1H), 6.34 (dd, *J* = 17.3, 1.6 Hz, 1H), 6.19 (dd, *J* = 17.2, 10.3 Hz, 1H), 5.97 (dd, *J* = 10.3, 1.7 Hz, 1H), 0.08 (s, 9H).

TetraPEG was functionalized with Cl-Phen (TetraPhen20k) following the same reaction and workup as those used for the synthesis of LPhen10k. Similarly, a near-quantitative yield was obtained using TMS-HEA as the external standard, as shown in Figure S2. ^1^H NMR (400 MHz, DMSO) δ 9.13 (dd, *J* = 4.3, 1.8 Hz, 1H), 8.92 (dd, *J* = 4.3, 1.7 Hz, 1H), 8.67 (dd, *J* = 8.3, 1.8 Hz, 1H), 8.34 (dd, *J* = 8.2, 1.7 Hz, 1H), 7.90–7.73 (m, 1H), 7.69 (dd, *J* = 8.1, 4.3 Hz, 1H), 7.38 (s, 1H), 6.43–6.28 (m, 1H), 6.20 (dd, *J* = 17.3, 10.3 Hz, 1H), 5.97 (dd, *J* = 10.3, 1.6 Hz, 1H), 0.09 (s, 9H).

Linear PEG and tetraPEG were also functionalized with Cl-Py (LPy10k and TetraPy20k) following the same reaction and workup used for the synthesis of LPhen10k and TetraPhen20k. The reaction yields were found to be nearly quantitative, determined using either a known amount of maleic acid or TMS-HEA as external standards, as shown in Figures S3 and S4, respectively. ^1^H NMR (400 MHz, DMSO, LPy10k shown in Figure S3): δ 7.88–7.44 (m, 1H), 6.63–6.30 (m, 1H), 5.24 (s, 9H). ^1^H NMR (400 MHz, DMSO, TetraPy20k shown in Figure S4): δ 8.41–8.35 (m, 3H), 7.01–6.94 (m, 3H), 6.44–5.90 (m, 3H), 4.22–4.12 (m, 6H), 0.09 (s, 9H). The absence of free, unreacted ligands was confirmed by comparing the product spectra with those of free ligands. Specifically, after PEG functionalization, the H-6 of Cl-Phen exhibited an upfield shift of ∼1 ppm, while all protons of Cl-Py showed an upfield shift of ∼0.5 ppm, which served as evidence that no unbound ligands remained.

#### 2.2.2 Dye Labeled Ligands

Phen-NH_2_ was functionalized with active NHS-Rhodamine and NHS-Fluorescein under similar mild basic conditions. For this purpose, Phen-NH_2_ (5 mg, 25.6 μmol) and either NHS- Rhodamine (14.9 mg, 1.1 equiv) or NHS-Fluorescein (13.3 mg, 1.1 equiv) were mixed in dimethylformamide (DMF, 0.5 mL), followed by the addition of triethylamine (15 μL, 4.4 equiv). The reaction was continued for 2 days at 50 °C. The product was passed through a silica column and washed with a mixture of chloroform and methanol, with an increasing fraction of methanol over time until the eluent became almost colorless. The organic solution was dried under reduced pressure, dissolved in water, and lyophilized to provide the products (PhenFlu and PhenRho) as colorful powders. ^1^H NMR (Phen-NH_2_, 400 MHz, DMSO, Figure S5) δ 9.06 (dd, *J* = 4.2, 1.6 Hz, 1H), 8.72–8.64 (m, 2H), 8.05 (dd, *J* = 8.2, 1.7 Hz, 1H), 7.74 (dd, *J* = 8.3, 4.2 Hz, 1H), 7.51 (dd, *J* = 8.1, 4.2 Hz, 1H), 6.87 (s, 1H), 6.14 (s, 2H). ^1^H NMR (PhenFlu, 400 MHz, DMSO, Figure S5) δ 9.06 (s, 1H), 8.68 (s, 1H), 8.06 (d, *J* = 14.0 Hz, 1H), 7.50 (s, 1H), 7.10 (d, *J* = 7.9 Hz, 1H), 6.87 (s, 1H), 6.56 (d, *J* = 8.5 Hz, 2H), 6.51 (s, 3H), 6.15 (s, 1H), 3.93 (s, 1H), 3.51 (s, 2H), 2.59 (s, 7H), 2.43 (q, *J* = 7.1 Hz, 7H), 1.32 (d, *J* = 10.1 Hz, 1H), 1.24 (s, 3H), 1.15 (s, 1H), 1.08–0.97 (m, 1H), 0.93 (t, *J* = 7.1 Hz, 10H), 0.84 (s, 2H). ^1^H NMR (PhenRho, 400 MHz, DMSO, Figure S5) δ 9.06 (s, 1H), 8.69 (d, *J* = 8.1 Hz, 2H), 8.35 (s, 1H), 8.24 (d, *J* = 8.0 Hz, 1H), 8.14–7.98 (m, 2H), 7.84 (d, *J* = 7.8 Hz, 1H), 7.75 (s, 1H), 7.50 (d, *J* = 10.6 Hz, 2H), 7.10 (d, *J* = 7.6 Hz, 1H), 6.87 (s, 1H), 6.50 (q, *J* = 2.1 Hz, 9H), 6.16 (s, 1H), 3.51 (s, 1H), 3.25 (d, *J* = 2.9 Hz, 1H), 2.95 (s, 2H), 2.94 (s, 17H), 2.59 (s, 7H), 2.49–2.41 (m, 5H), 1.24 (s, 2H), 1.19–1.06 (m, 4H), 0.95 (t, *J* = 7.1 Hz, 10H).

As the starting NHS dyes contain two structural isomers, assigning the ^1^H NMR spectra could not confirm the synthesis. Accordingly, attenuated total reflection (ATR) FTIR spectra of Phen-NH_2_ before and after functionalization with dyes are compared in Figure S6. The primary amine groups of Phen-NH_2_ reflect a strong absorption band in the range of 3300–3500 cm^–1^ due to the stretching vibration of the N–H bonds. This band is not present in the dye-labeled ligands, confirming the successful completion of the click reaction.

#### 2.2.3 Synthesis of Cages and Cage-Cross-Linked Hydrogels

Employing a small-molecule ligand (Cl-Phen) would result in the formation of soluble isolated cages; instead, interconnected cages would form a hydrogel if multitopic polymeric ligands were used. Unlike former reports,^42,43^ our purchased TPT was not soluble in water, even at high temperatures, and in the presence of coordination metal ions and auxiliary Phen ligands. Direct mixing of all cage components in water and annealing overnight at 80 °C provided hydrogels with clear TPT precipitates as a white powder. After trial and error, we found that TPT was more soluble in chloroform at high temperatures. Therefore, TPT was weighed in its solid state inside a glass vial, polymeric ligand (LPhen10k, TetraPhen10k, LPy10k, or TetraPy10k) and/or the small-molecule ligand (Cl-Phen) were dissolved in chloroform, and the metal ion was dissolved in DMF. Both solutions were added to the glass vial containing TPT along with a small magnetic stir bar and sealed by screw caps. The vial was placed in a water bath and mixed at 60 °C for 4 h, resulting in a homogeneous solution or gel depending on the functionality of the polymer precursor and the type of metal ion, with no sign of TPT precipitation. The clear solution or gel was subsequently dried, first at room temperature and then in a vacuum oven at 80 °C overnight. The isolated cage formed by Cl-Phen was readily soluble in water; however, for the cage cross-linked polymer networks, the dried film on the glass surface was gently scratched with a small spatula to help homogeneous swelling in water. After the desired amount of water was added, the vial was sealed and placed in a water bath at 80 °C for 10 h. All measurements were performed at least 24 h later to make sure all condensed water was absorbed by the hydrogel. The ^1^H NMR spectra of plain Cl-Phen, before and after complexation by Pd^2+^, are compared with that of the isolated cage in Figure S7. The seven protons of Cl-Phen are significantly downfield-shifted upon complexation with Pd^2+^; however, they are upshifted in the cage structure compared to the complex, which confirms the formation of the cage with a less crowded structure. ^1^H NMR (Cl-Phen, 400 MHz, DMSO, Figure S7) δ 9.21 (dd, *J* = 4.3, 1.7 Hz, 1H), 9.13 (dd, *J* = 4.3, 1.7 Hz, 1H), 8.71 (dd, *J* = 8.4, 1.7 Hz, 1H), 8.50 (dd, *J* = 8.1, 1.8 Hz, 1H), 8.31 (s, 1H), 7.94 (dd, *J* = 8.4, 4.3 Hz, 1H), 7.82 (dd, *J* = 8.1, 4.3 Hz, 1H). ^1^H NMR (Cl-Phen_2_Pd^2+^ complex, 400 MHz, D_2_O, Figure S7) δ 9.28–9.19 (m, 2H), 9.16 (d, *J* = 5.5 Hz, 1H), 8.89 (d, *J* = 8.2 Hz, 1H), 8.42 (s, 1H), 8.26 (dd, *J* = 8.5, 5.5 Hz, 1H), 8.15 (dd, *J* = 8.3, 5.5 Hz, 1H). ^1^H NMR (cage assembly with auxiliary Cl-Phen ligands, 400 MHz, D_2_O, Figure S7) δ 9.49 (d, *J* = 5.9 Hz, 4H), 9.20–9.12 (m, 1H), 8.91 (d, *J* = 5.8 Hz, 4H), 8.82 (d, *J* = 8.3 Hz, 1H), 8.41 (d, *J* = 12.8 Hz, 1H), 8.20–8.08 (m, 1H), 8.03 (d, *J* = 5.4 Hz, 1H), 7.95 (dd, *J* = 8.6, 5.4 Hz, 1H), 7.84 (dd, *J* = 8.4, 5.4 Hz, 1H). Dye-labeled cages were prepared following the same procedure, using dye-labeled small-molecule ligands instead of Cl-Phen.

### 2.3 Characterization

Rheological measurements were carried out on a stress-controlled Anton Paar Physica MCR 301 rheometer with a 25 mm cone–plate geometry. Samples were loaded onto the lower plate and pressed to fill the gap (∼250 μm). A few drops of silicone oil were placed around the sample to seal the gap. The standard Anton Paar solvent trap filled with water was used to minimize sample drying. Rheological measurements consist of a frequency sweep and self-healing. Particularly, an equilibration step including an oscillatory shear (10 min, *ϒ* = 0.01, ω = 1 rad s^–1^) at 40 °C, was followed by five frequency-sweep measurements (*ϒ* = 0.01, ω = 100–0.01 rad s^–1^) at 40, 30, 25, 20, and 10 °C. After adjusting the temperature to 25 °C, the self-healing measurement was performed, including a time-sweep (5 min, *ϒ* = 0.01, ω = 10 rad s^–1^), followed by an amplitude sweep (*ϒ* = 0.01–10, ω=10 rad s^–1^), and an immediate time-sweep (5 min, *ϒ* = 0.01, ω = 10 rad s^–1^). A new set of samples was used for alternating high- and low-amplitude oscillations (six 1 min segments, *ϒ*_H_ = 500%, *ϒ*_L_ = 0.5%, ω=10 rad s^–1^) to evaluate the repeatability of self-healing and the long-term stability of the material. This was followed by a stress relaxation measurement (*ϒ* = 10%) to be compared with frequency-sweep results.

UV–vis spectra were recorded on a Cary 300 UV–vis spectrophotometer (Agilent Technologies) at room temperature. Wavelengths between 200 and 700 nm were scanned at a rate of 200 nm min^–1^. Emission of dye-labeled ligands and cages was measured on a JASCO FP-8000 fluorescence spectrometer at room temperature. Samples containing fluorescein and rhodamine dyes were, respectively, excited at 490 and 530 nm. The kinetics of ligand exchange were measured by time-course fluorescence spectroscopy measurements upon excitation at 490 nm and monitoring the emission at 570 nm.

Magic Angle Spinning (MAS) ^1^H NMR spectroscopy measurements were performed on a Bruker Avance DSX 400 NMR spectrometer operating at 399.87 MHz 1H frequency using 4 mm rotors and inserts specially developed to investigate gels and soft matter. The ^1^H single-pulse excitation NMR spectra were recorded by using a commercial three-channel Bruker 4 mm probe head at 4 kHz MAS, averaging 512 scans with a 5 s recycle delay.

### 2.4 Computational Details

DFT calculations were carried out using the Gaussian 16 software.^44^ Geometry optimizations were performed with the BP86 functional,^45,46^ combined with Grimme’s D3 dispersion correction.^47^ A split-valence basis set with double polarization functions (Def2SVP) was employed.^48,49^ All stationary points were verified through frequency calculations. Single-point energy refinements were conducted using the M06L functional^50,51^ and the Def2TZVPP basis set. Solvent effects (water) were modeled using the SMD continuum solvation approach.^52^ Reported free energies include zero-point energy and thermal and entropic corrections at 298 K (gas phase).

## 3 Results and Discussion

To study the role of network connectivity on viscoelastic properties and self-healing behavior of transient polymer networks, we employ a MOC-cross-linked system that enables the formation of robust dynamic cross-links with higher functionality than those enabled by traditional node-like metal complex junctions. We chose PEG as the polymer backbone, as it is commercially available at difference chain lengths and architectures, with narrow microstructural distributions.^53^ We make networks by transiently connecting terminal groups of the PEG chains in a hydrogel system.^9^ As such, the network dynamics in dilute conditions represent those of the transient bonds, providing a model system to study the network structure and dynamics.^18,54–57^ As the MOC, we select one of the first reported cages, which has an octahedral geometry with six vertices. This cage is formed by a three component assembly, including 4 eq TPT ligands forming the faces, 6 eq Pd^2+^ metal ions forming the vertices, and 6 eq auxiliary Phen ligands fulfilling the square-planar coordination geometry requirement of Pd^2+^ ions on the vertices. This cage was first reported by Fujita et al. using cis-protected Pd^2+^ metal ions at each vertex.^58^ They also formed a similar cage using bipyridine (BPy) auxiliary ligands at the vertices and used it for host–guest interaction^59^ and catalyzing the Diels–Alder reaction inside the cavity.^60^ However, to the best of our knowledge, this cage is not reported using auxiliary Phen ligands. The NMR data support the formation of the discrete Pd-cage rather than a mixture of free ligand and simple bis-complex as the cage spectrum is not a weighted superposition of the two others. On complexation to Pd, the phenanthroline aromatic protons are deshielded, with the bis-complex showing the largest downfield shifts (consistent with strong local electron withdrawal at ligated phenanthrolines). Coordination of the phenanthroline N atoms to Pd withdraws electron density from the ligand π-system, specifically from the α-protons (adjacent to the N). This typically produces downfield shifts of protons closest to the N (2,9) and, to a lesser extent, nearby protons (3,8 and 4,7). In practice, presumably due to the square-planar preference of Pd^2+^, the electron density around α-protons is less disturbed. In the isolated cage, the same phenanthroline signals are displaced compared to the free ligand but are shifted to a lesser extent than in the bis-complex, as listed in Table S1. The pattern of resonances is simplified in a way that is consistent with the increased symmetry imposed by the cage structure. Taken together, the direction and magnitude of chemical-shift changes, the changed multiplicities and relative integrals, and the single, well-defined set of resonances (rather than a superposition of free ligand + bis-complex) are consistent with the formation of a single, symmetric cage species. Moreover, the single doublets at 9.49 and 8.91 ppm, which could be assigned to the symmetric TPT protons, confirm the presence of only one type of coordinated TPT. The integration of these peaks compared to the whole Cl-Phen protons confirms that about 5% of added Cl-Phen is not present in the cage structure. However, the extra singlet at 6.42 ppm, which can be associated with the bis-complex, suggests that about 11% of the Cl-Phen ligands are in bis-complexes. Accordingly, the yield of cage formation should be 90–95%.

Schmidt et al. have recently integrated this cage with auxiliary BPy ligands in polymeric systems and studied the mechano-responsive release of drugs loaded inside the cavity upon cage disassembly by sonication.^42,43^ We further revised this cage-cross-linked system by employing Phen-functionalized telechelic polymer chains, as the auxiliary ligand, forming transient networks with 6-fold cross-links, as illustrated in Figure 1. An equivalent polymer network with 6-fold junctions can be formed by simple node-like metal complexes, employing monodentate ligands in the presence of metal ions with octahedral coordination geometry. However, monodentate ligands can barely form stable enough coordinative bonds, which combined with the steric hindrance of such a crowded complex, would result in a viscous solution rather than a self-standing gel.^61^

**Figure 1:**
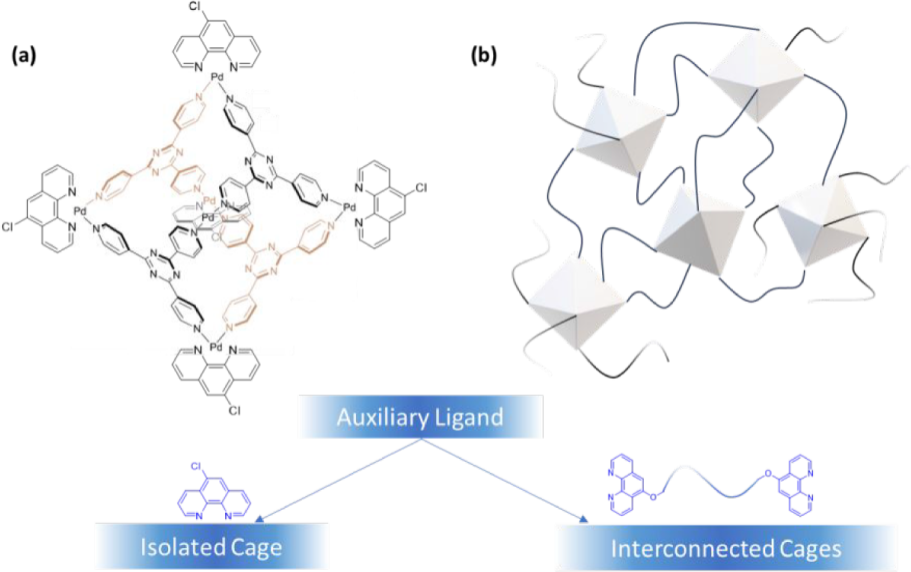
(a) Formation of the isolated cage using Cl-Phen as the auxiliary ligand. (b) Integration of the octahedral cage as 6-fold cross-links in polymer networks using the polymeric LPhen10k ligand as the auxiliary ligand.

As TPT was insoluble in water, even at high temperatures and in the presence of coordinating metal ions and auxiliary Phen ligands, we dissolved all three components in a mixture of organic solvents, as described in the Experimental Section, dried them, and redissolved them in water. This process contains three steps of high-temperature treatment, including dissolution, drying under vacuum, and swelling, respectively, at 60, 80, and 80 °C, for 4, 10, and 10 h. This would result in complete dissolution and complexation of TPT, and the formation of ordered cages. Our initial attempts using shorter annealing times, specifically during the drying and reswelling steps (each for 1 h), did not form the expected cages, as explained later.

To tune the network connectivity, we first considered varying two supramolecular parameters related to the cage structure. Specifically, using metal ions demanding more than four coordinative bonds would allow coordinating more auxiliary ligands on the vertices. For instance, Fe^2+^ with its octahedral coordination geometry, could provide the 90° angle to bond TPT ligands on the vertices and stabilize the cage, and at the same time allow two Phen ligands to coordinate on the external planes of each vertex, or be satisfied with nitrate counterions. As such, the additional auxiliary ligands would increase the functionality of the cross-linking point from 6 to 12. Accordingly, we mixed TPT:LPhen10k:M^2+^ at 4:12:6 in addition to 4:6:6 molar ratios to study if the octahedral geometry would be satisfied with two auxiliary Phen ligands or with one Phen and two nitrate counterions, respectively. However, none of the employed metal ions, except for Pd^2+^, including Fe^2+^, Ni^2+^, Co^2+^, Zn^2+^, and Cu^2+^, were able to form a hydrogel under the employed preparation conditions. To the best of our knowledge, octahedral TPT-based cages are only reported based on Pd^2+^ and Pt^2+^.^58,59,62^ We will further investigate this observation using DFT calculations to determine whether the failure to form a gel is intrinsic or if it may depend on the preparation conditions.

Moreover, using two pyridine ligands instead of Phen could double the number of network strands expanding at each vertex and increase the cross-link functionality from 6 to 12. Accordingly, we mixed TPT:LPy10k:Pd^2+^ at 4:12:6 and even 4:6:6 molar ratios, and still could not make a hydrogel. Our qualitative conclusion was that MOCs are very sensitive to structural variations. Accordingly, the octahedral coordination geometry of Fe^2+^ could not be satisfied with two bidentate ligands, and the combination of three ligands could already make too much steric crowding at the cage vertices. Also, ions with a tetrahedral coordination preference (like Zn^2+^) or trigonal bipyramidal preference (like Cu^2+^) could result in structural distortions that are not tolerable. Moreover, the coordination of two monodentate ligands could not afford the required stabilization effect that could be provided by one chelating bidentate ligand, as illustrated in Figure S8. Accordingly, we further studied the cage formation using different metal ions and ligands by DFT calculations.

The interaction of the ligands was first studied using a model that included the entire system, i.e., six metal centers each with a +2 charge, connected by the TPT ligand and one Phen ligand per metal center, resulting in an overall charge of +12. Next, the potential coordination of nitrate ligands was explored by including two units to neutralize the overall charge. However, in the case of palladium, this neutralization does not contribute to stabilizing the system, as palladium adopts a well-defined square planar geometry with four strong Pd–N bonds—2.044 Å from the TPT and 2.051 Å from the Phen. These bond lengths remain unchanged upon inclusion of nitrate ligands, which interact only weakly with the palladium center, with the closest distances being 3.224 Å (Pd···O) and 3.026 Å (Pd···N). To further support this observation and facilitate comparison with the other metals under investigation, we calculated the Mayer Bond Orders (MBOs),^63–65^ which were found to be 0.463 for TPT and 0.636 for Phen. This indicates that Phen ligands are more strongly bonded than TPTs, which explains the fragility of the cage structure.

Energetically, forcing the formation of an adduct with two nitrate ligands around each palladium center is endergonic—contrary to what might be expected—and therefore does not provide any stabilization in terms of Gibbs free energy. Quantitatively, although there is an enthalpic stabilization of 111.3 kcal mol^–1^, the overall process results in a destabilization of 52.9 kcal mol^–1^ in Gibbs energy. This discrepancy must be attributed to entropy, which worsens the energetics by nearly 7 kcal mol^–1^ per nitrate ligand. This result is not driven by the specific structure of the cage: a simplified monomer model, in which the bidentate cage ligand is replaced by two simple pyridyl ligands (while retaining the Phen ligands), leads to the same conclusion—a destabilization of 14.7 kcal mol^–1^. The slightly higher stability observed in the cage system is likely due to additional hydrogen-bonding interactions between the nitrate ligands and the hydrogen atoms of the cage. To note that we did not include the chloride of the Cl-Phen ligand, and instead, the model Phen was used for the sake of simplicity and particularly to avoid asymmetry. However, we performed seminal calculations to confirm that the simplification affected neither the energetics nor the sterics of the system. For the complete cage, the overall binding energy decreased modestly from 169.7 to 164.4 kcal mol^–1^ relative to the sum of six Cl-Phen ligands, three linker ligands, and six Pd­(NO_3_)_2_ species. This corresponds to a change of only 5.3 kcal mol^–1^ in total, which is less than 1 kcal mol^–1^ per Cl-Phen ligand. Moreover, this stabilization is likely overestimated due to the solvent model as the relative energy difference in the gas phase is only 1.1 kcal mol^–1^. Structurally, the MBO values for the Pd–N bonds remained unchanged. Additionally, the simplified square-planar complex with two nitrate ligands and Cl-Phen was found to be 2.0 kcal mol^–1^ less stable, indicating that the chloride ligand has a minimal destabilizing effect on the entire cage. If anything, its presence may slightly destabilize the system while potentially enhancing solubility, particularly in polar solvents. Next, the replacement of the metal, Pd by Ni, gives a similar result (55.4 kcal mol^–1^), demonstrating the similar behavior of transition metals of the same chemical group. When analyzing the Ni-based monomer, we observe a similar performance, with a destabilization of 12.1 kcal mol^–1^ upon the addition of two nitrate ligands. However, it is particularly interesting to note that the formation of the monomer is exergonic by 43.2 kcal mol^–1^ with respect to that of Pd­(NO^3^)^2^, whereas it is endergonic by 66.2 kcal mol^–1^ for the analogous Ni species. However, we wanted to delve deeper into the analysis and also investigate Zn, as it is the only other first-row transition metal experimentally tested that reliably maintains a singlet ground state. Interestingly, the destabilization associated with nitrate coordination to Zn is again comparable at 50.9 kcal mol^–1^. This is unexpected given that the nitrate ligands bind strongly to the zinc centers, leading to a stable octahedral geometry. Returning to the MBOs, a surprising observation arises, as shown in Table 1: the MBO values decrease significantly when nitrates coordinate to zinc, and the total sum of MBOs across all bonds drops from 1.993 to 1.975. This hinted at a possible explanation for why only palladium successfully forms the desired cages. However, it is also true that in the absence of the nitrate ligands the geometry around each zinc metal center is tetrahedral, indicating a rather constrained and strained environment.

**Table 1:** Mayer Bond Orders for the M^2+^ Based Cages with Four TPT Ligands and 6 Phen Ligands with or without Nitrates

M	Number of nitrate ligands	M···N1 (TPT)	M···N2 (TPT)	M···N3 (Phen)	M···N4 (Phen)	M···O1 (nitrate)	M···O2 (nitrate)	Sum of MBOs around M
Pd	0	0.548	0.549	0.637	0.637	0.000	0.000	2.371
Pd	2	0.590	0.590	0.647	0.646	0.000	0.000	2.473
Ni	0	0.385	0.386	0.596	0.596	0.000	0.000	1.963
Ni	2	0.482	0.532	0.648	0.617	0.173	0.000	2.452
Zn	0	0.461	0.461	0.535	0.535	0.000	0.000	1.993
Zn	2	0.319	0.319	0.313	0.314	0.355	0.356	1.975

Examining the total MBO values for palladium and nickel further supports this hypothesis—2.371 for Pd and only 1.963 for Ni—highlighting the structural weakness of the latter. Extending the analysis to open-shell metal ions such as copper, cobalt, and iron, we observe that these metals adopt octahedral geometries, although copper shows slight distortion. However, due to their open-shell character, assigning a definitive ground state for the entire resulting cage becomes challenging, limiting the reliability of geometrical interpretations.

We also considered the insertion of monodentate pyridine ligands, i.e., replacing each Phen ligand with two pyridine molecules. The failure to locate a stable structure for this system is entirely consistent with the observed destabilization of 119.0 kcal mol^–1^. Although this modification was not pursued experimentally, focusing on the nature of the bidentate ligand, we also investigated the effect of replacing Phen with bipyridine (BPy). This substitution results in a stabilization of 13.6 kcal mol^–1^, which can be reasonably attributed to the greater flexibility of the BPy ligand. However, this effect is relatively modest when distributed across the six metal centers, amounting to roughly 2 kcal mol^–1^ per center.

After discussing the Phen ligand, we also wanted to better understand whether the TPT ligand undergoes significant or minor distortion upon coordination. The deformation energies were found to be 6.0, 4.6, and 2.2 kcal mol^–1^ for the Pd, Ni, and Zn cages, respectively. This indicates that although it might initially seem unfavorable, palladium actively participates in coordinating with the TPT ligand. In contrast, the other metals do not, which is reflected in a much lower binding energy.

Experimentally, we do not observe coordination of two Phen ligands to each palladium center to form an octahedral geometry at the cage vertices. Nevertheless, for the sake of consistency, we made a concerted effort to model the corresponding structure, even though it involves six additional Phen ligands in total. In this model, one of the Pd–N bonds broke, as shown in Figure 2, specifically the bond to a nitrogen atom that is one of the three linking atoms of a TPT ligand. To confirm this behavior, we carried out several computational attempts and consistently observed the same outcome. These results indicate that the opening of the cage structure is not a matter of serendipity but a reproducible and inherent feature of the system under these coordination conditions. In addition to the breaking of the aforementioned bond, it should also be noted that at least one of the Pd–N bonds from the Phen ligands becomes activated to the point of de facto dissociation with a distance greater than 2.8 Å. Furthermore, the Pd center that loses its bond with the TPT ligand also shows another Pd–N bond stretching to 2.684 Å. The MBOs fully confirm this, with negligible values of 0.097 and 0.005, respectively. Repeating the same analysis with nickel results in a similar outcome, and upon reviewing the MBOs, the total sums are significantly lower compared to those of palladium, once again explaining why cage formation is not feasible. In fact, for each metal center, two of the Ni–N bonds are practically broken. Moving on to zinc, the effect is even more pronounced, leading to the complete fragmentation of the cage into three nearly identical units—except in one case, where a Zn center has an additional ligand. In the case of palladium, it is interesting to note that this effect is even more evident, resulting in the rupture of the unit due to its inability to adopt a hexacoordinated geometry. Remarkably, the stabilizing energies for Pd, Ni, and Zn are 33.7, 41.8, and 41.2 kcal mol^–1^, respectively.

**Figure 2:**
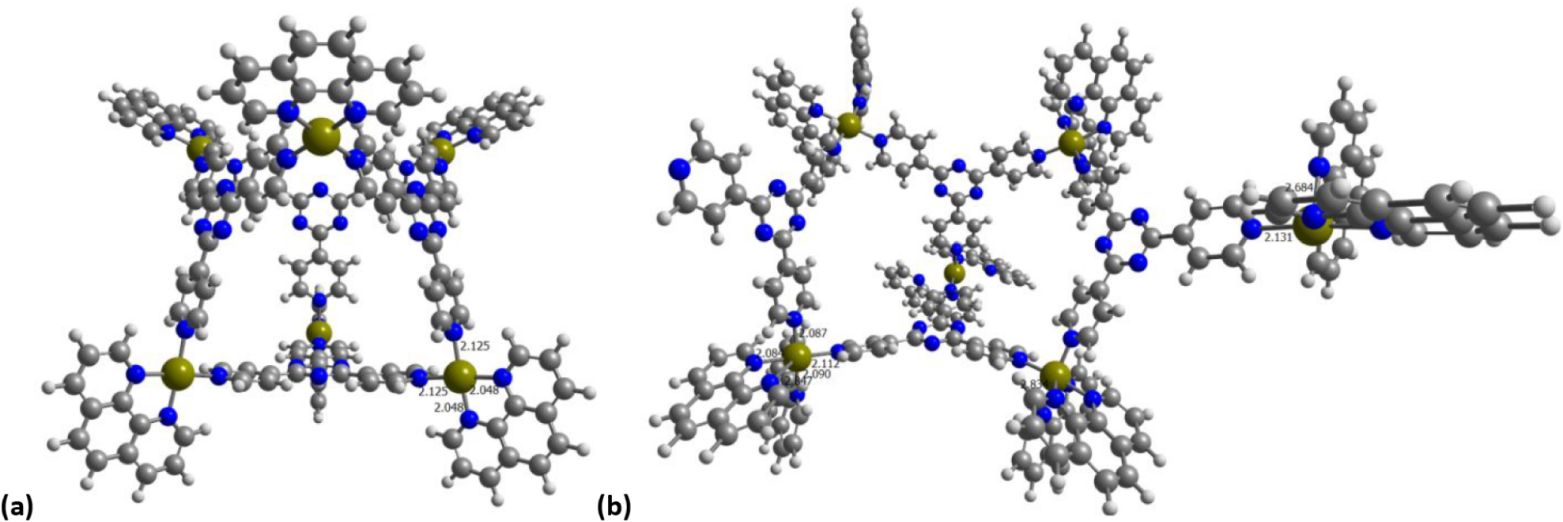
Pd^2+^ based cage including 4 TPT and (a) 6 or (b) 12 Phen ligands (selected distances in Å).

Turning to conceptual DFT,^66,67^ if there were still any doubts as to why palladium is the ideal candidate to act as the linker between TPT and Phen ligands and enable cage formation, they are dispelled by its significantly lower chemical hardness (0.035 (Pd), 0.031 (Ni), 0.028 (Zn) a.u.), that is, a much smaller HOMO–LUMO gap, indicating a lower reactivity that makes it less prone to react with any nucleophile.

Consequently, in subsequent studies, we kept using the cage formed by TPT:Phen:Pd^2+^ in a 4:6:6 ratio and instead focused on macromolecular parameters to tune the network connectivity. The concentrations of all components used in the preparation of all hydrogel samples are listed in Table S2. Our first choice of polymer precursor was the linear LPhen10k because it can form model-like networks with purely transient bonds. Moreover, the formation of bis Phen-Pd^2+^ complexes with square-planar geometry only results in chain extension; therefore, any sign of gelation in the presence of LPhen10k is indicative of higher-order assemblies. The chain overlap concentration, c*, for PEG10k dissolved in water is about 27 g L^–1^, equivalent to 2.7 w/v%. This corresponds to having two chain ends coming together at the cross-linking point. To have six chain ends coming together around the cage, we need three times the c* concentration. Accordingly, we formed the hydrogel at a concentration of 80 g L^–1^. Of course, gels could also be made at lower concentrations; however, to form a percolated network, chains should stretch beyond their coil conformation at thermodynamic equilibrium.^2,68^ Also, higher concentrations are normally accompanied by compromised properties, indicating the formation of further defects, likely due to steric hindrance of polymer chains.^18^ To evaluate the connectivity and stability of transient networks, we studied their behavior under small-amplitude oscillatory shear (SAOS) deformation and followed the storage and loss moduli at different temperatures. We apply horizontal shifts and form the master curves at the reference temperature of 25 °C, according to the time–temperature superposition (TTS) principle, as demonstrated in Figure 3.

**Figure 3:**
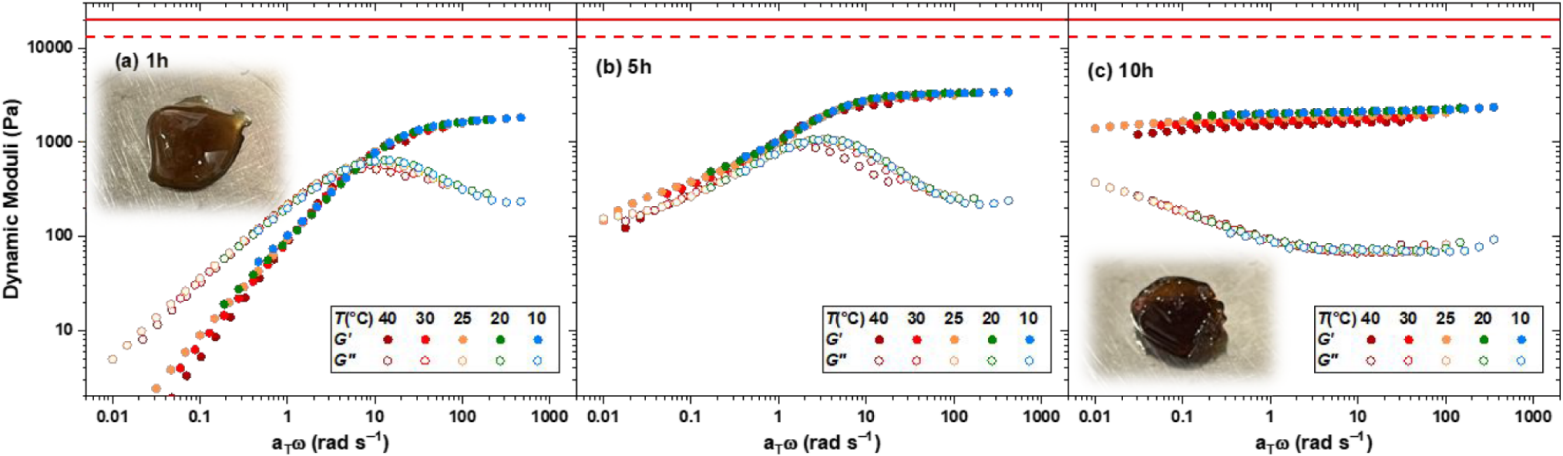
Dynamic moduli master curves of the cage-cross-linked hydrogels formed by LPhen10k at φ = 80 g L^–1^, as a function of annealing time: (a) 1 h, (b) 5 h, and (c) 10 h, and the corresponding prediction of plateau modulus by the affine (solid line) and phantom (dashed line) network models.

The sample obtained after a short two-step annealing process (1 h of drying at 80 °C and 1 h of reswelling in water at 80 °C) was a firm hydrogel, as shown in the inset of Figure 3a, with a plateau modulus of 2 kPa, as shown in Figure 3a. The affine $\left(G_{N}^{0}=\nu RT\right)$ and the phantom $\left(G_{N}^{0}=\left(\nu -\mu \right)RT\right)$ network models, with ν and μ, respectively, denoting the concentration of network strands and the concentration of cross-links, predict a plateau modulus of 20 and 13.3 kPa for an ideal network with 6-fold junctions, as shown by the solid and dashed lines in Figure 3.^9^ However, the experimentally obtained plateau moduli are about an order of magnitude lower than expected. A similar hydrogel, formed by BPy-functionalized linear PEG6k at φ = 100 g L^–1^, was reported by Schmidt et al. to have a plateau modulus of 3 kPa,^42^ far below the expected values of 83 and 55 kPa according to the affine and phantom network models, respectively. These observations are indicative of the presence of network defects or incomplete cage formation when a linear precursor is used.

In addition, we were expecting to have a more stable gel in the presence of Phen rather than BPy, due to the rigid planar structure of the former. Counterintuitively, the hydrogel formed by a short annealing time (Figure 3a) demonstrated a crossover frequency of ∼10 rad s^–1^. Whereas, in the presence of BPy,^42^ the crossover was not achieved down to the measured frequency of 0.1 Hz (∼0.63 rad s^–1^). Therefore, to enhance the possibility of cage formation in the presence of long polymer chains, we increased the annealing time. Surprisingly, a second relaxation mode emerged after 5 h of annealing and raised to dominate the relaxation after 10 h of annealing, as shown in Figure 3b,c. Consequently, the hydrogel contains two modes of transient bonding and consequent relaxation. If we assign the slow relaxation mode to the ligand exchange of intact cages, the fast mode should be associated with a combination of homoleptic bis-Phen–Pd^2+^ complexes and heteroleptic complexes made by the collaboration of pyridine ligands from TPT and Phen. Otherwise, the sole bis-complexation is unable to form a gel and create a plateau modulus, as reflected in Figure 3a, even in the presence of a small fraction of intact cages.

Furthermore, all master-curves shown in Figure 3 demonstrate a slight deviation from TTS, as the curves do not perfectly overlap. The violation of TTS is further proof for the presence of two relaxation mechanisms with different temperature dependencies. This is also reflected in the monotonically decreasing storage modulus in Figure 3c and the broad minimum observed in the loss modulus. This type of thermo-rheological complexity is quite common in side-chain supramolecular polymers as well, since the high-frequency dissociation of supramolecular bonds has a different activation energy compared to the low-frequency polymeric dynamics.^69,70^ In the current study, however, polymeric dynamics are absent, as chains are not entangled; instead, two modes of supramolecular association dominate, namely the node-like association (homo- or heteroleptic) and the collective cage assembly. To verify this, we fit the dynamic moduli master curves with a weighted summation of two generalized Maxwell modes, each represented by a log-normal distribution function. The obtained relaxation time spectra, as shown in Figure S9, reveal that annealing does not significantly change the lifetimes of these two modes but does increase the relative contribution of the slow mode associated with the cage assembly.

To further distinguish the identity of the fast and slow modes, we formed hydrogels by homoleptic complexation of TetraPhen20k, at a Phen:Pd^2+^ ratio of 2:1, and the chain overlap concentration of φ = 40 g L^–1^. We compared the crossover frequency with that of the hydrogel formed by the heteroleptic complexation of TetraPhen20k and TetraPy20k, at a Phen:Py:Pd^2+^ ratio of 1:2:1, and a concentration of φ = 60 g L^–1^. The concentration difference accounts for fulfilling the square-planar coordination geometry of Pd^2+^ in homo- and heteroleptic complexes, achieved by bringing together either two Phen ligands or one Phen and two Py chain ends, respectively. The hydrogels demonstrate crossover frequencies of roughly 1 and 0.2 rad s^–1^, respectively, as shown in Figure 4. Considering that the apparent relaxation time in networks at the percolation threshold appears faster than the bond dissociation time, these results can support the association of the fast mode to homo- and heteroleptic Phen and Py complexes. Intuitively, we can conclude that the collective assembly as a cage brings additional stabilization beyond that of individual complexes. The corresponding MAS ^1^H NMR spectra of hydrogels obtained at various annealing times, as shown in Figure S10, sharpen and show better-resolved aromatic signals after 10 h of annealing, which can be interpreted as an increased fraction of intact, symmetric cages, with reduced structural heterogeneity and slower ligand exchange, consistent with the growth of the slow relaxation mode in rheology.

**Figure 4:**
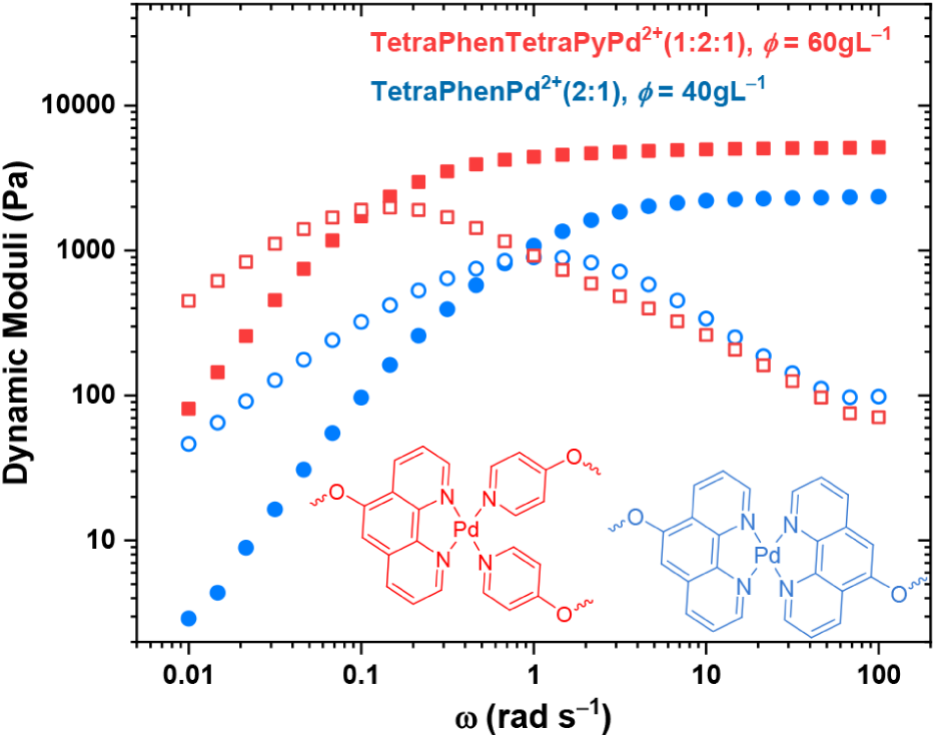
Dynamic moduli of hydrogels made by homoleptic TetraPhen20k (φ = 40 g L^–1^) and heteroleptic TetraPhen20k and TetraPy20k complexes (φ = 60 g L^–1^) with Pd^2+^ at 25 °C.

The subsequent studies on hydrogels formed by the linear precursor, as described in Figures S11–S14, demonstrated a significant and inevitable contribution of the fast mode, which undermines distinguishing the cage contribution; therefore, we switched to use the tetra-arm precursor, TetraPhen20k, to form the hydrogels, as illustrated in Figure S15. In contrast to LPhen10k, the tetra-arm precursor inevitably introduces 1.5 covalent cross-linking points per transient bond formed by cages; therefore, the network percolation is further preserved after the fast relaxation mode, and the cage contribution at lower frequencies is highlighted.

Increasing the concentration from 1.5c* to 3c*, at constant TPT:Phen:Pd^2+^ = 4:6:6, leads to a higher plateau modulus, as it is evident in the master curves shown in Figure 5a–d. The shift factors obtained from these data fit well to an Arrhenius equation, yielding an activation energy of 102 ± 6 kJ mol^–1^, which is in the same range already obtained for homo- and heteroleptic Phen complexes with Pd^2+^.^71^ We fit the master curves with the two-mode generalized Maxwell model. The fitted curves are shown as solid black curves in Figure 5a–d, and the corresponding relaxation time spectra are compared in Figure 5f. Contributions of the slow and fast modes were quantified by integrating the area under each peak. Accordingly, the plateau modulus and the contribution of the fast mode are shown in the main plot of Figure 5g, while the fast and slow relaxation times are depicted in the inset plot. Interestingly, while the plateau modulus follows the phantom network model prediction at low polymer concentrations, it saturates as concentration increases and drops below the expectations from the phantom model. In parallel, the contribution of the fast mode increases at the highest concentration, suggesting that cage formation is hindered at the high polymer concentration limit due to steric crowding. At the low concentration limit, the slow mode demonstrates much faster dynamics, as reflected in the corresponding relaxation time shown in the inset plot of Figure 5g, while the stability of the fast mode remains unchanged. These results imply that either chain stretching destabilizes the cage or the network approaches the percolation threshold, leading to a rapid decay in connectivity by the onset of ligand exchange, as reflected in the rheology.

**Figure 5:**
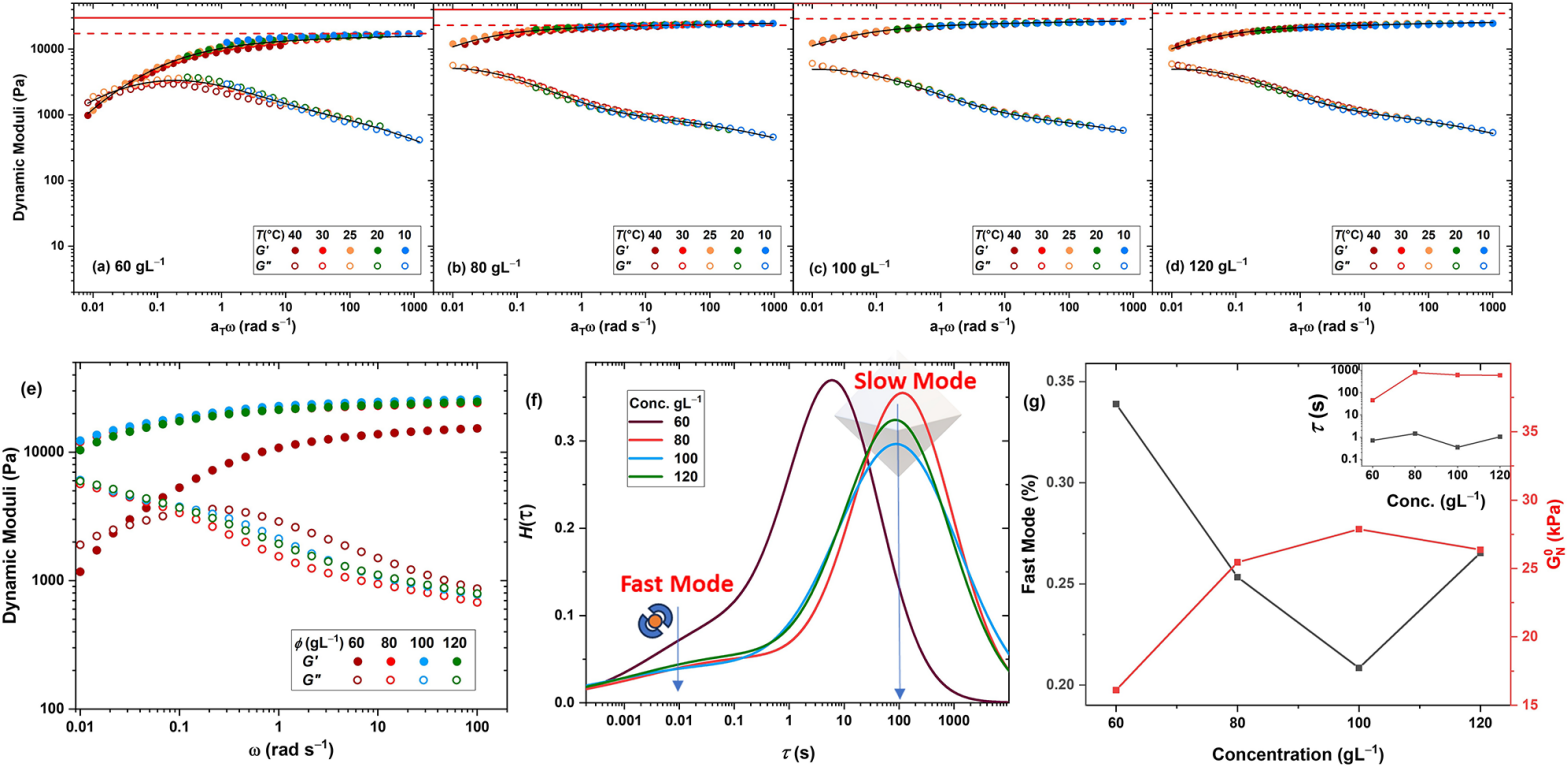
Dynamic moduli master curves of the cage-cross-linked hydrogels formed by TetraPhen20k at various polymer concentrations: (a) 60 g L^–1^, (b) 80 g L^–1^, (c) 100 g L^–1^, and (d) 120 g L^–1^. The affine (solid) and phantom (dashed) network model predictions of plateau modulus are shown by red lines. (e) Effect of concentration on dynamic moduli at 25 °C. (f) Relaxation time spectra and (g) the plateau moduli and the contribution of fast mode (main plot) and the relaxation time of the fast and slow modes (inset plot).

To tune the network connectivity, the cage content was reduced while maintaining a constant polymer concentration (φ = 80 g L^–1^). This means, as we decrease the TPT concentration, some polymeryl Phen ligands remain unassociated. We added extra Pd^2+^ (Phen:Pd^2+^ = 2:1) to encourage these loose ends to create extended network strands by forming bis-complexes, as explained in Table S2. Accordingly, as cage content is reduced, the cross-link concentration correspondingly decreases, but the network strand length increases due to the formation of new bis-Phen complexes, as illustrated in Figure S16. For instance, as shown in Table S2, the sample with 70% cage content has 30% loose Phen groups, which are encouraged to form extended network strands by the added ∼18% extra Pd^2+^ ions. The dynamic moduli reflect a very smooth trend, as demonstrated by the master curves shown in Figure 6a–e. To decouple the contribution and stability of the two modes, we similarly fit the master curves by the weighted summation of two generalized Maxwell modes. The fit curves are shown by solid black curves in Figure 6a–e, and the corresponding relaxation time spectra are compared in Figure 6g. Accordingly, while the modulus agrees with the phantom network model prediction at high cage content, it gradually approaches the prediction of the affine network model as the cage content decreases. Such a transition between phantom and affine network models has already been reported for Sakai’s model-type covalently cross-linked tetraPEG networks, when the polymer content was increased beyond the chain overlap concentration.^68^ The phantom model assumes that junction fluctuations reduce the effective number of elastically active chains, while the affine model assumes junctions are fixed in space.^1^ In our system, varying the cage content alters the extent of complete versus defective cages, which directly changes the connectivity of the network strands. At high cage content, misconnectivities and incomplete cage formation increase junction fluctuations, leading to phantomlike behavior. As the cage content decreases, more complete cages are formed, reducing defects and stabilizing 6-fold junctions, thereby moving the system toward affine behavior. Thus, this transition provides a useful measure for how molecular-scale connectivity defects manifest in macroscopic elasticity. Moreover, in our previous studies on metallo-supramolecular networks with node-like junctions,^8,18,72^ the mechanical response always fell below the phantom network model prediction due to connectivity defects. In contrast, the fact that our MOC-cross-linked networks surpass the phantom prediction at reduced cage content suggests that defects are minimized and the system approaches a more homogeneous network structure. This further supports that the phantom–affine transition is meaningful as a microstructural signature. Counterintuitively, reducing the cage content leads to a decreased contribution of the fast mode, as shown in the main plot of Figure 6h, while increasing the relaxation time of the slow mode, as shown in the inset plot. Together, these observations suggest that intact cages are more prevalent at low cage contents, whereas higher cage contents favor the formation of defective cages, whose relaxation is reflected as the fast mode.

**Figure 6:**
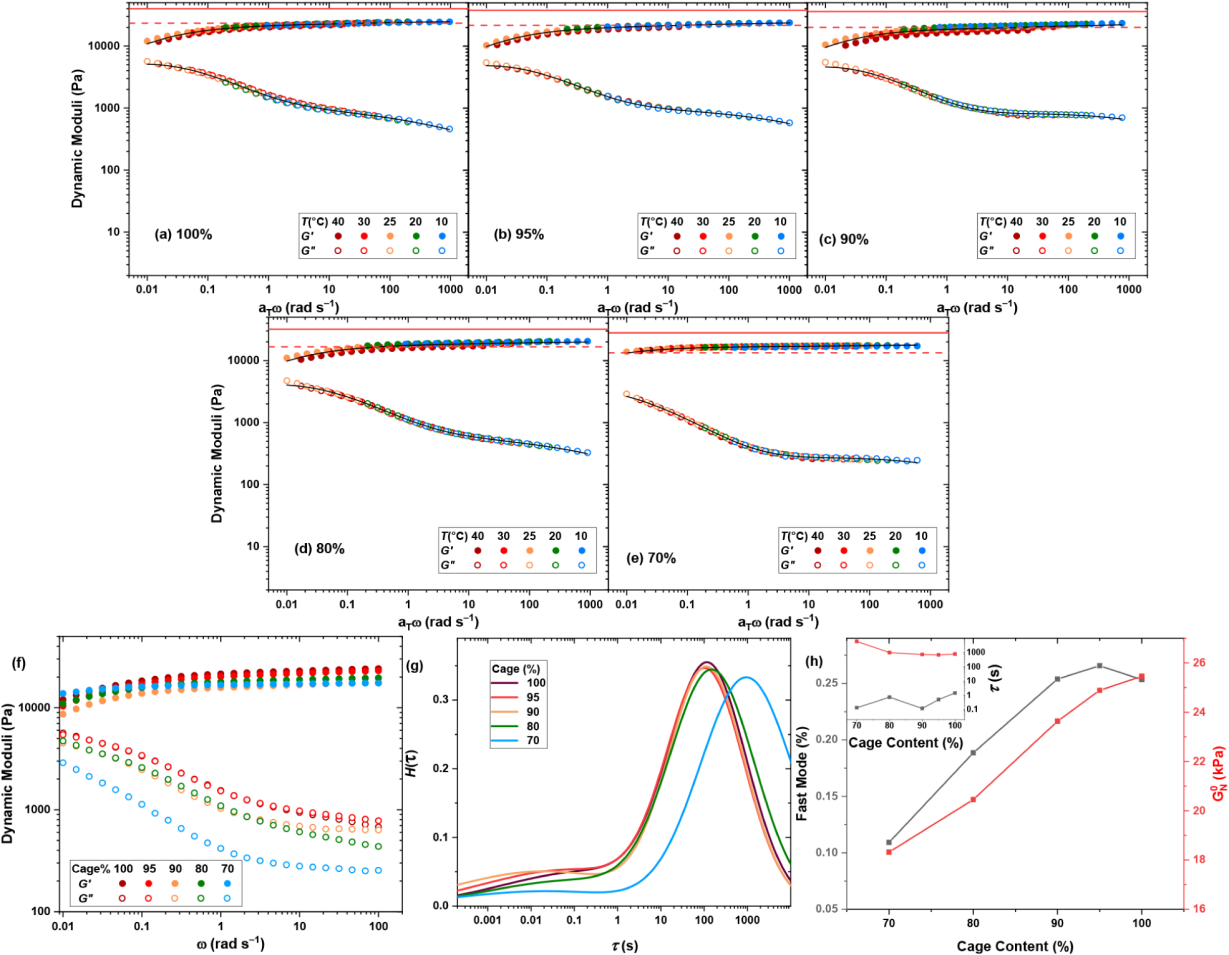
Dynamic moduli master curves of the cage-cross-linked hydrogels formed by TetraPhen20k at constant polymer concentration φ = 80 g L^–1^, but various cage contents: (a) 100%, (b) 95%, (c) 90%, (d) 80%, and (e) 70%. The affine (solid) and phantom (dashed) network model predictions of plateau modulus are shown by red lines. (f) Effect of cage content on dynamic moduli at 25 °C. (g) Relaxation time spectra, and (h) plateau moduli and the contribution of fast mode (main plot) and the relaxation time of the fast and slow modes (inset plot).

An alternative way to tune the network connectivity, which was already used by Johnson et al., is to replace a fraction of polymeric ligands with the small-molecule equivalent.^36,73^ Fujita’s cages, based on the two-component assembly of bis-4-pyridyl ligands and Pd^2+^ has been used in their work, which do not have the possibility of forming competitive bis complexes. In contrast, the additional small-molecule ligand is capable of forming bis complexes in our three-component system, which can significantly undermine network connectivity. The extent of bis-complexation depends on its thermodynamic preference over the cage assembly, which can be temporarily manipulated by external factors, such as thermal annealing.

Accordingly, we replace fractions of the polymeric ligand with the small-molecule equivalent in the feed composition by proportionally decreasing the polymer concentration from φ = 80 g L^–1^, while keeping the TPT and Pd^2+^ concentrations constant (TPT:Phen:Pd^2+^ = 4:6:6). This means, as summarized in Table S2, the concentrations of TPT and Pd^2+^ remain constant, whereas the Phen concentration represents the sum of Cl-Phen and polymeryl Phen, where the fraction of Cl-Phen increases from 0% to 30% among samples. Ideally, the cage concentration remains unchanged, but the cross-link functionality decreases, as illustrated in Figure S16. In sharp contrast to the effect of decreasing the cage content, the storage modulus drops upon the introduction of Cl-Phen, as demonstrated by the master curves shown in Figure 7a–e. We similarly fit the master curves by the weighted summation of two generalized Maxwell modes. The fit curves are shown by solid black lines in Figure 7a–e, and the corresponding relaxation time spectra are compared in Figure 7g. Accordingly, while the modulus agrees with the phantom network model prediction at high polymer content, it gradually decreases and significantly drops below the expectation at the largest Cl-Phen content. Therefore, as expected, a fraction of Cl-Phen creates bis complexes with polymeryl Phen ligands, forming loose ends and directly lowering the network connectivity. This is reflected as the increase in the contribution of the fast mode, as shown in the main plot of Figure 7h. In parallel, the relaxation times of both the fast and the slow modes are accelerated, as demonstrated in the inset plot. Consequently, the rheological study can hardly reflect the cage contribution in cross-link functionality due to the parallel introduction of misconnectivities.

**Figure 7:**
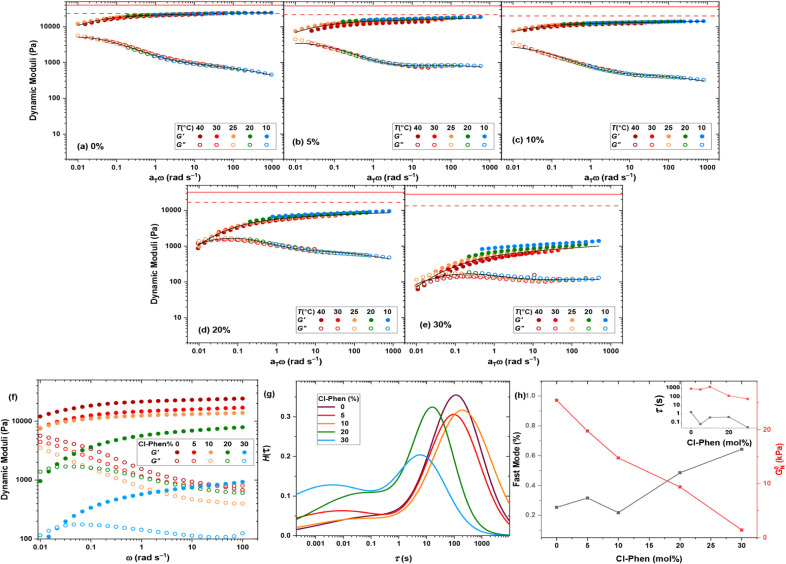
Dynamic moduli master curves of the cage-cross-linked hydrogels formed by TetraPhen20k at constant cage content, but replacing a fraction of polymeric ligands with Cl-Phen: (a) 0%, (b) 5%, (c) 10%, (d) 20%, and (e) 30%. The affine (solid line) and phantom (dashed line) network model predictions of plateau modulus are shown by red lines. (f) The effect of Cl-Phen fraction on dynamic moduli at 25 °C. (g) Relaxation time spectra, and (h) the plateau moduli and the contribution of fast mode (main plot), and the relaxation time of the fast and slow modes (inset plot).

To confirm the results obtained by SAOS measurements, we study the effect of polymer concentration, cage content, and introducing a small-molecule ligand using stress-relaxation measurements. The relaxation moduli, shown in Figure S17, were fitted to a two-mode generalized Maxwell model, and the obtained relaxation time spectra, shown in Figure S18, were used to analyze the contribution of the slow and fast relaxation modes. The major observation, specifically the counterintuitive increased contribution of the fast mode at high polymer concentrations, large cage contents, and in the presence of Cl-Phen, was noted.

To study the cage stability on the molecular scale, independent from the network connectivity, we use Fluorescence Resonance Energy Transfer (FRET). Accordingly, we label the small-molecule Phen-NH_2_ ligand with fluorescein and rhodamine dyes through a click reaction between the amine functionality of the ligand and the NHS functionality of the dyes. Fluorescein and rhodamine are donor–acceptor FRET pairs, since the emission of the former at 530 nm, when excited at 490 nm, can be absorbed by the latter and turned into a lower-energy emission at 570 nm, if they are in close proximity. This means, if we form individual cages with either Phen-Flu or Phen-Rho auxiliary ligands and mix them; the exchange of dye-labeled ligands should bring the dyes into close proximity to raise the FRET emission at 570 nm, when excited at 490 nm, as illustrated in Figure 8. The absorption spectra of the dye-labeled ligands and the corresponding dye-labeled cages, shown in Figure S19, demonstrate that the absorption peaks of both dyes are not shifted after functionalization and assembly. Similarly, the emission bands of the dye-labeled ligands and the corresponding dye-labeled cages are not shifted after functionalization and assembly, as demonstrated in Figure S20. Interestingly, when mixed, the dye-labeled ligands immediately return a FRET emission at 570 nm, when excited at 490 nm, as shown in Figure S20a. This suggests that dye-labeled ligands are not completely soluble in water at the molecular scale; instead, they aggregate due to their hydrophobicity. In contrast, the mixture of the dye-labeled cages does not show any sign of FRET intensity at short times, as shown in Figure S20b. Nevertheless, the intensity at 570 nm increases over time, as demonstrated in Figure 8b. We explain the exchange profile by a first-order kinetic equation, specifically by fitting the normalized time-course intensity development with a stretched exponential function. This results in a cage stability of around τ = 2.4 × 10^4^ s, which is four times longer than the largest relaxation time of 6 × 10^3^ s that was obtained for the sample with 70% cage content (Figure 6e) based on rheological measurements. Accordingly, we hypothesize that the integration of cages into polymer networks can cause destabilization, mostly due to the chain dynamics induced by thermal energy, but further in-depth studies are required to validate this hypothesis.

**Figure 8:**
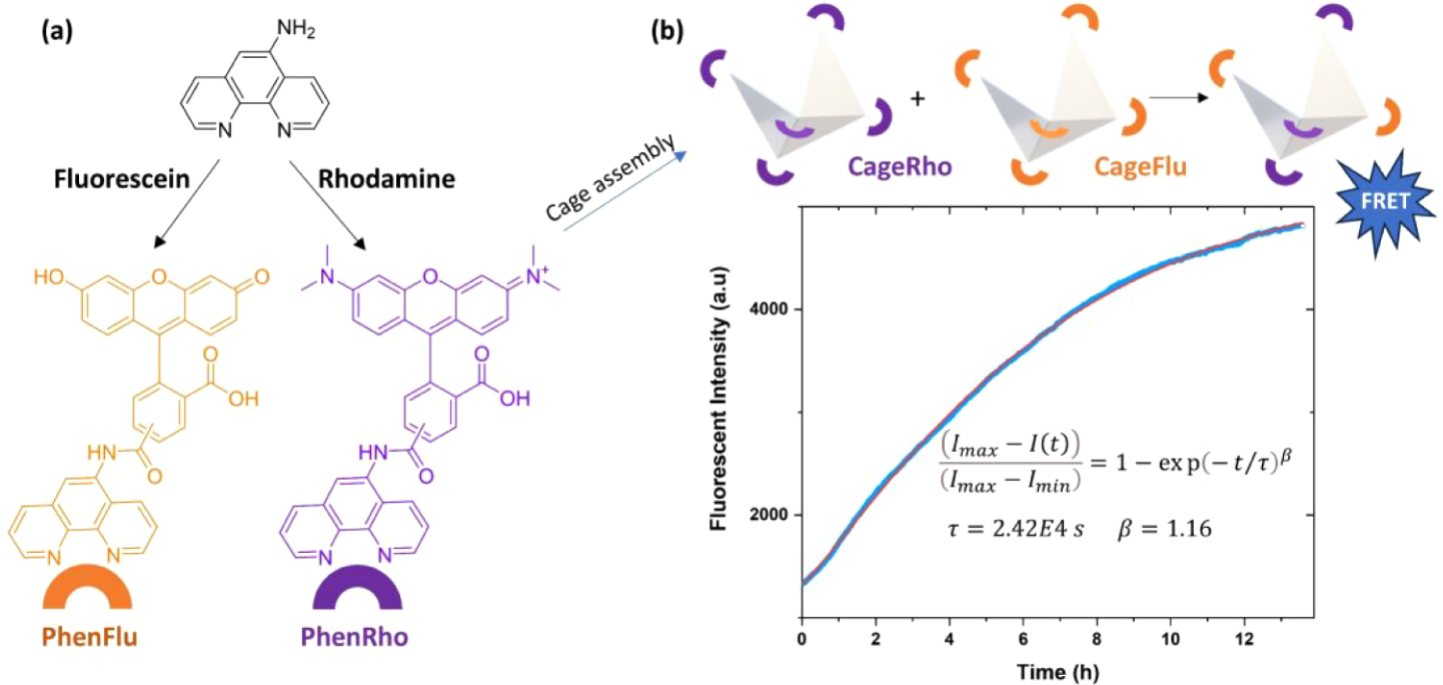
(a) Synthesis of the dye-labeled ligands and the corresponding cages. (b) Time-course fluorescence spectroscopy demonstrating a rise in FRET intensity, as captured by a stretched exponential function.

Finally, we study the self-healing of the cage-cross-linked hydrogels. We expect that increased cross-link functionality should facilitate the ligand exchange process and as such improve the self-healing capacity.^3,10,12^ For that, we break the structures in an amplitude-sweep measurement by applying an oscillatory shear deformation beyond the linear viscoelastic (LVE) regime and study the immediate network recovery in a subsequent time-sweep at the same oscillation frequency. While all samples show a network breakup and enter the non-LVE regime at ∼100% deformation, as shown in Figures S21–S23, they indeed demonstrate outstanding instantaneous self-healing, as demonstrated by the immediate recovery of the storage and loss moduli, as shown for representative samples in Figure 9. The intact hydrogel film with a thickness of 200 μm could be recovered after the measurement, as shown in Figure 9a, which demonstrates the structural stability of the cages and the self-healing capacity of the network. As the entanglement molar mass of PEG, which is reported to be ∼2 kg mol^–1^, will increase to ∼20 kg mol^–1^ at 10 w/v% polymer concentration, the network strands are expected to be shorter than an entanglement, and therefore, do not significantly contribute to the network formation. Nevertheless, such entanglements are effective only on time scales shorter than the dissociation time of transient bonds, as they will subsequently relax immediately by the fast Rouse mechanism.^1,74^ This means, after structure breakup at high-amplitude oscillations, many of the transient bonds are open, and entanglements are not expected to stay effective anymore. Therefore, their role in self-healing in the model-type structure under study is not expected to be significant. Furthermore, our networks are built only based on physical bonds and contain ∼90% water; therefore, they have weak shape integrity and high adhesion. Therefore, imaging the healing process, as conventionally demonstrated by the adhesion of cut surfaces and recovery of mechanical properties after healing, as traditionally done by tensile tests, is not relevant and applicable. Nevertheless, to verify the repeatability of self-healing and the long-term stability of cage-cross-linked networks, we perform three cycles of high- and low-amplitude oscillations. The representative data shown in Figure 9, and the complete set of results shown in Figures S21–S23, indeed demonstrate the same level of fast and effective network recovery, as it was witnessed in single-step recovery after amplitude-sweep measurements.

**Figure 9:**
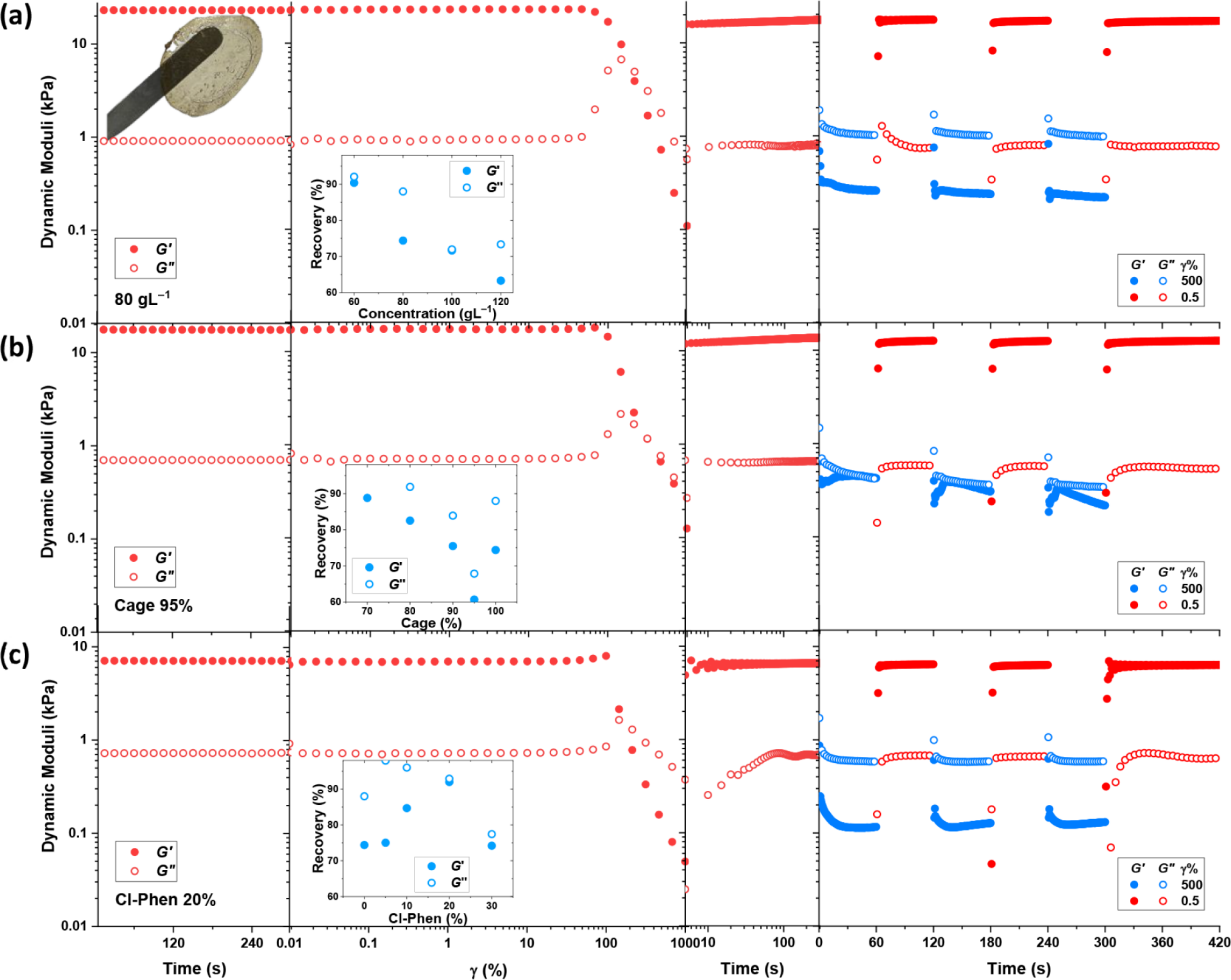
Amplitude sweep between two time-sweep measurements at the same oscillation frequency of 10 rad s^–1^ (main plot), followed by alternating large- and low-amplitude oscillations for representative samples: (a) φ = 80 g L^–1^ (physical appearance of hydrogel film recovered after rheological measurement), (b) cage content = 90%, and (c) Cl-Phen content = 20%. The average recovery of dynamic moduli after amplitude sweep tests as a function of polymer concentration, cage content, and Cl-Phen content is shown in the inset plots.

Transient metallo-supramolecular polymer networks with node-like cross-links based on single metal complexes, with this level of plateau modulus and long relaxation times, normally do not demonstrate such a good self-healing ability.^13,75^ To verify this, we prepared two metallo-supramolecular polymer networks using the tetraPhen20k precursor, right at the chain overlap concentration of 40 g L^–1^, in the presence of Ni^2+^ and Co^2+^ at the M^2+^:Phen ratio of 1:2. SAOS measurements, as shown in Figure S24, demonstrate a relaxation time quite longer than that of cage-cross-linked networks for the hydrogels made by Ni^2+^, while the relaxation time for the hydrogel made by Co^2+^ is shorter. The recovery rate of the sample made by Ni^2+^ after network breakdown by amplitude sweep is surprisingly slow, and a plateau could be reached after almost 10 s, as shown in Figure S25. After the measurement, the sample was clearly crushed and thrown out of the gap between the upper and lower plates. Therefore, the final storage modulus, after alternating high- and low-amplitude oscillations, was logically quite lower than the starting one. This is in sharp contrast to the behavior of the cage-cross-linked networks, where an intact film was recovered after the self-healing measurements. The sample made by Co^2+^, despite not being crashed and thrown out of the gap by the high torque, shows a similar slow network recovery after amplitude sweep. Both samples are not quite in the non-LVE region at 500% deformation; therefore, *G*′ stays above *G*″ at high-amplitude oscillation segments, while cage-cross-linked networks demonstrate a sol-like behavior with *G*′ lower than *G*″. Nevertheless, the cage-cross-linked networks demonstrate faster and more stable recovery after each high-amplitude oscillation segment, all suggesting that cage-cross-linked systems have better self-healing capacity presumably due to higher functionality junctions.

## 4 Conclusions

This study highlights the role of network connectivity on viscoelastic properties of transient polymer networks that employ metal–organic cage cross-links as transient junctions. Unexpectedly, the chosen three-component cage did not provide a wide range of accessible functionality, as we could not increase the junction connectivity beyond six, neither by employing metal ions with octahedral coordination geometry nor with monodentate auxiliary ligands. We exploited DFT calculations to demonstrate the efficiency of Pd^2+^ versus other transition metal ions, and bi- versus monodentate ligands, in forming the desired octahedron cage. This supports the experimental observation that only Pd^2+^-based cages reliably form with the necessary structural rigidity and stability. Using the rheological results, we demonstrated that decreasing the junction functionality can result in a transition between phantom and affine network model behavior. Moreover, hydrogels demonstrated good and instantaneous self-healing, which can also be associated with the high functionality of junctions. Nevertheless, the formation of intact cages inside polymer networks is a key challenge for achieving this behavior. Specifically, high polymer concentration can undermine cage formation due to steric hindrance, whereas cage formation at low concentrations demands chain overstretching, resulting in the destabilization of cages. Accordingly, decreasing the cage content at constant polymer concentration is the best approach for enhancing cage formation, but replacing polymeric with small-molecule ligands amplifies misconnectivities. Consequently, depending on the cage structure, strategies to tune the network connectivity and improve cage formation can differ. These findings pave the way for more precise engineering of network connectivity in cage-cross-linked systems and establish a robust material platform to study how network design influences material properties, such as self-healing and stimuli responsiveness.

## Supplementary Material



## Notes

Additional polymer characterizations, rheological data, and DFT simulation results (PDF)
