# Supplementary material for: Tuning Connectivity in a Three-Component Assembly of Metal–Organic Cage-Cross-Linked Polymer Networks
Source: Macromolecules. 2025 Oct 22;58(21):11703–17. doi: 10.1021/acs.macromol.5c02021 (PMC12613800; doi:10.1021/acs.macromol.5c02021)
Supplement: Supplementary file 1 [file ma5c02021_si_001.pdf]

# - Supporting Information-

Tuning Connectivity in Three-Component Assembly of Metal-Organic Cage-Crosslinked Polymer Networks

Mostafa Ahmadi,[1] Josep Duran,[2] Albert Poater[2]

[1]*Department of Chemistry, Johannes Gutenberg-Universität Mainz, Duesbergweg 10–14, D-55128 Mainz, Germany.*

[2] *Institut de Química Computacional i Catàlisi, Departament de Química, Universitat de Girona, c/ Mª Aurèlia Capmany 69, 17003 Girona, Catalonia, Spain*

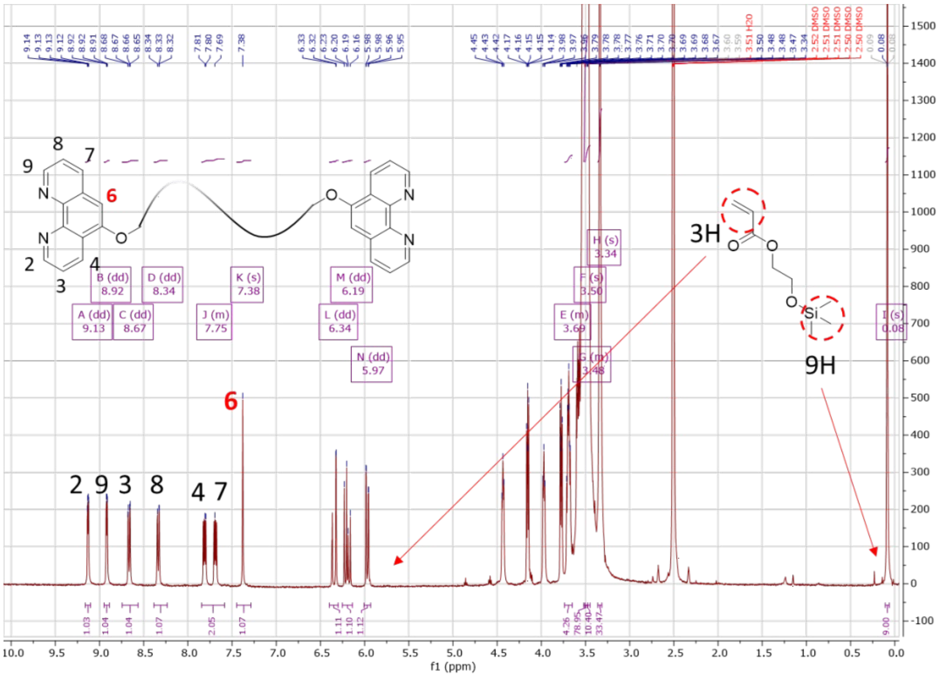

Figure S1. **^{1}H NMR (400 MHz, DMSO) of the LPhen10k in the presence of TMS capped 2-hydroxyethyl acrylate as the external standard.**

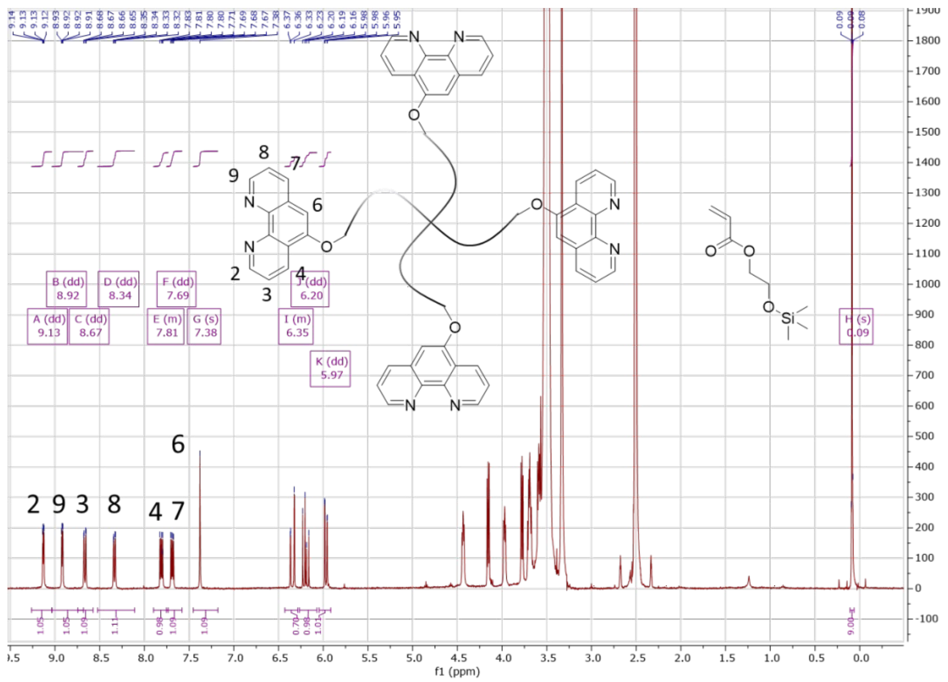

Figure S2. ^{1}**H NMR (400 MHz, DMSO) of the TetraPhen20k in the presence of TMS capped 2-hydroxyethyl acrylate as the external standard.**

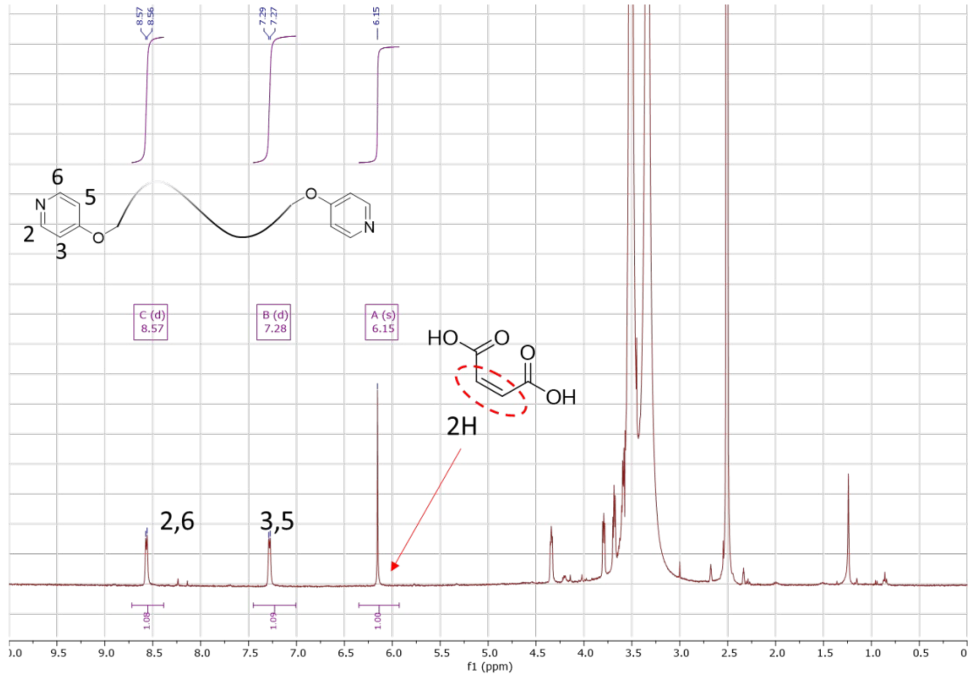

Figure S3. ^{1}**H NMR (400 MHz, DMSO) of the LPy10k in the presence of maleic acid as the external standard.**

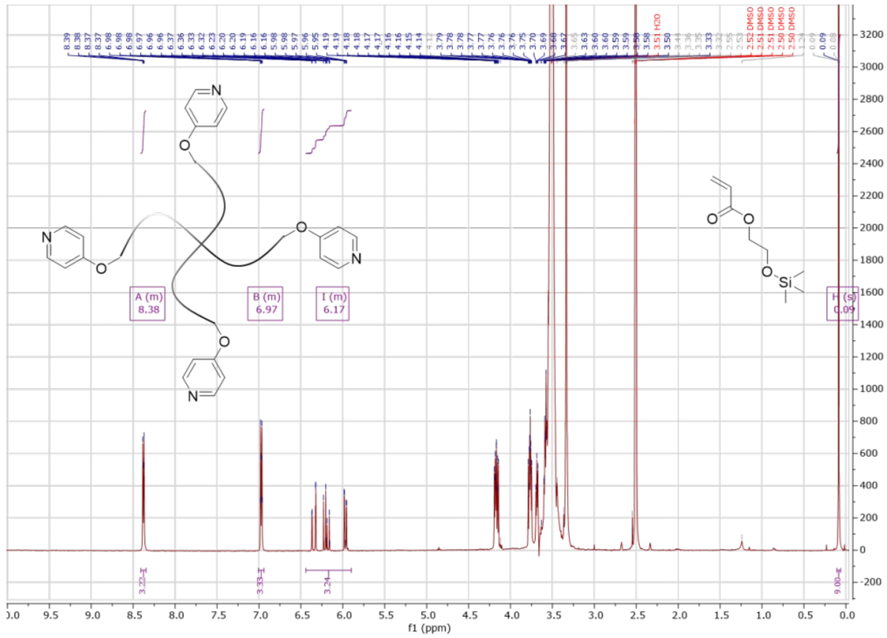

Figure S4. ^{1}H NMR (400 MHz, DMSO) of the TetraPy20k in the presence of TMS capped 2-hydroxyethyl acrylate as the external standard.

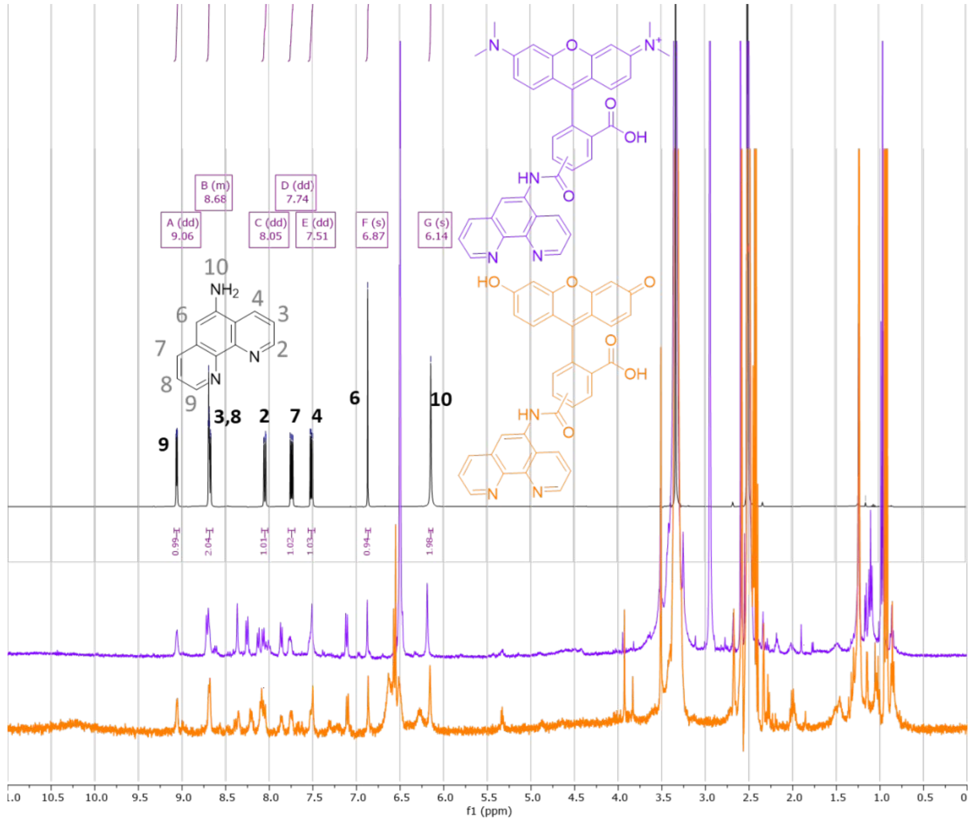

Figure S5. ^{1}H NMR (400 MHz, DMSO) of the Phen-NH_2 before and after functionalization with fluorescein (orange) and rhodamine (purple).

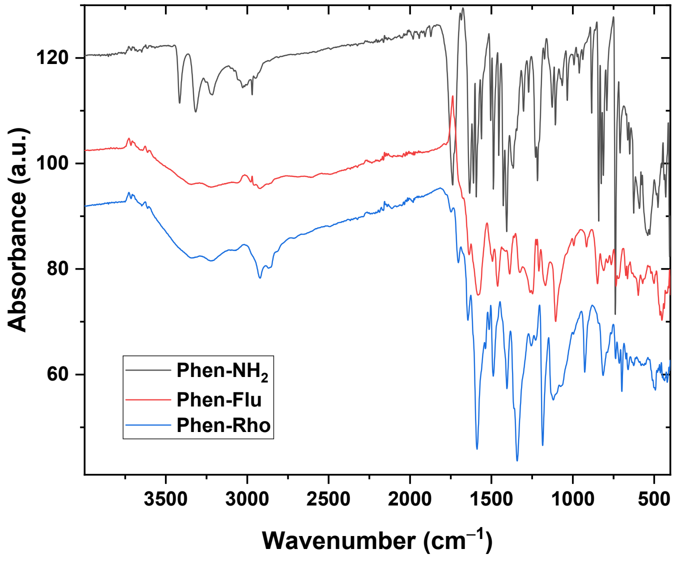

Figure S6. ATR of the Phen-NH_2 before and after functionalization with fluorescein and rhodamine.

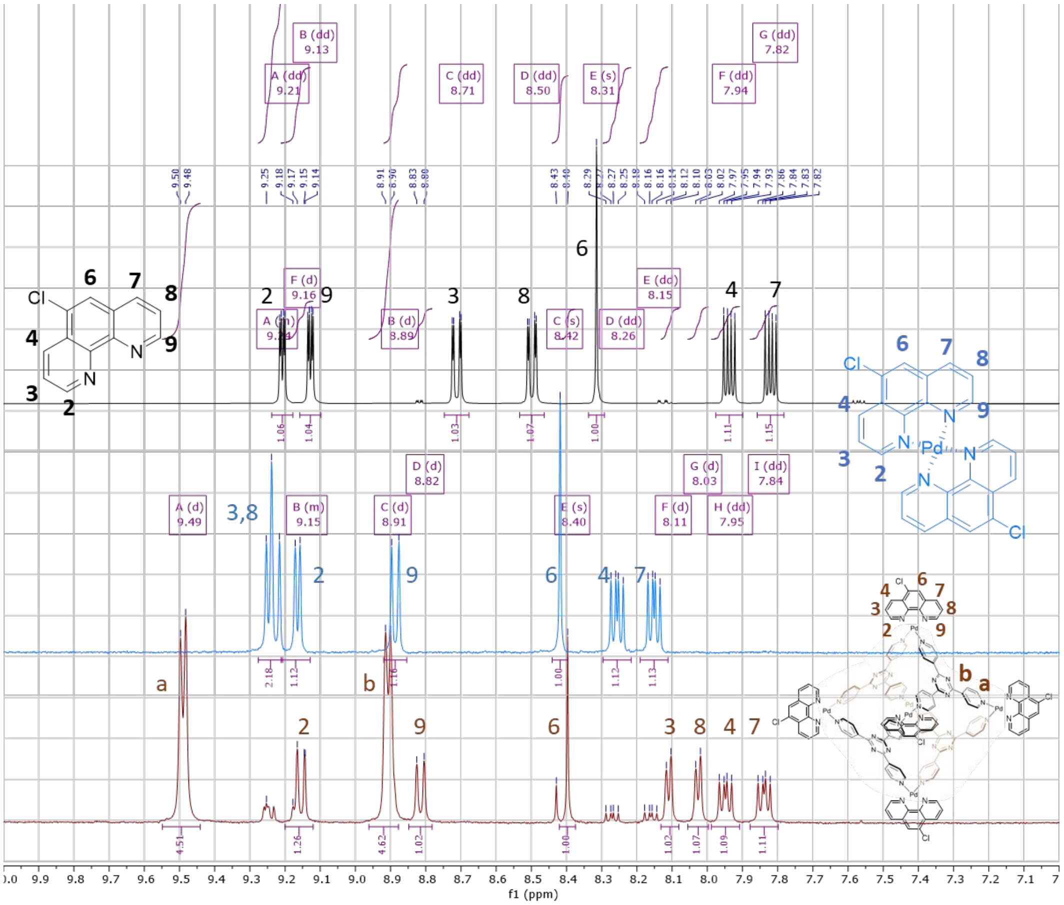

Figure S7. ^{1}H NMR (400 MHz) of the Cl-Phen (in DMSO) and the corresponding bis complex with Pd^{2+} and upon cage assembly (both in D_2O).

Table S1. Assignment of Cl-Phen protons and shifts upon complexation and cage formation.

Likely proton (phen)	Free ligand (ppm)	bis-Pd (ppm)	Cage (ppm)	Δδ (bis – free) (ppm)	Δδ (cage – free) (ppm)
2	9.21	9.16	**9.15**	-0.05	-0.06
9	9.13	8.89	**8.82**	-0.24	-0.31
3	8.71	9.25	**8.11**	0.54	−0.60
8	8.50	9.22	**8.03**	0.72	−0.47
6	8.31	8.42	**8.40**	0.11	0.09
4	7.94	8.26	**7.95**	0.32	0.01
7	7.82	8.15	**7.84**	0.33	0.02

Table S2. Concentration of all components used in preparation of hydrogels.

Polymer			Polymer (g L^{-1})	Phen (polymeryl) (mmol L^{-1})	TPT (mmol L^{-1})	Pd^{2+} (mmol L^{-1})	Considerations		Figure
				6 eq	4 eq	6 eq			
LPhen10k			80	16	10.7	16			3
TetraPhen20k			60	12	8.0	12			5a
TetraPhen20k			80	16	10.7	16			5b
TetraPhen20k			100	20	13.3	20			5c
TetraPhen20k			120	24	16.0	24			5d
	Cage Content (%)						Extra Phen in system (mmol L^{-1})	Added Pd^{2+} (mmol L^{-1})[a]	
							2 eq	1 eq	
TetraPhen20k	100		80	16	10.7	16	0	0	6a
TetraPhen20k	95		80	16	10.1	15.2	0.8	0.4	6b
TetraPhen20k	90		80	16	9.6	14.4	1.6	0.8	6c
TetraPhen20k	80		80	16	8.5	12.8	3.2	1.6	6d
TetraPhen20k	70		80	16	7.5	11.2	4.8	2.4	6e
		Cl-Phen content (%)					Added Cl-Phen (mmol L^{-1})[b]		
TetraPhen20k		0	80	16	10.7	16	0		7a
TetraPhen20k		5	76	15.2	10.7	16	0.8		7b
TetraPhen20k		10	72	14.4	10.7	16	1.6		7c
TetraPhen20k		20	64	12.8	10.7	16	3.2		7d
TetraPhen20k		30	56	11.2	10.7	16	4.8		7e

a. Added on top of the Pd^{2+} listed in 7th column to form bis complexes with the extra Phen (polymeryl), which exists overstoichiometric with respected to TPT.

b. Combined with the polymeryl Phen satisfies stoichiometric TPT:Phen = 4:6 ratio.

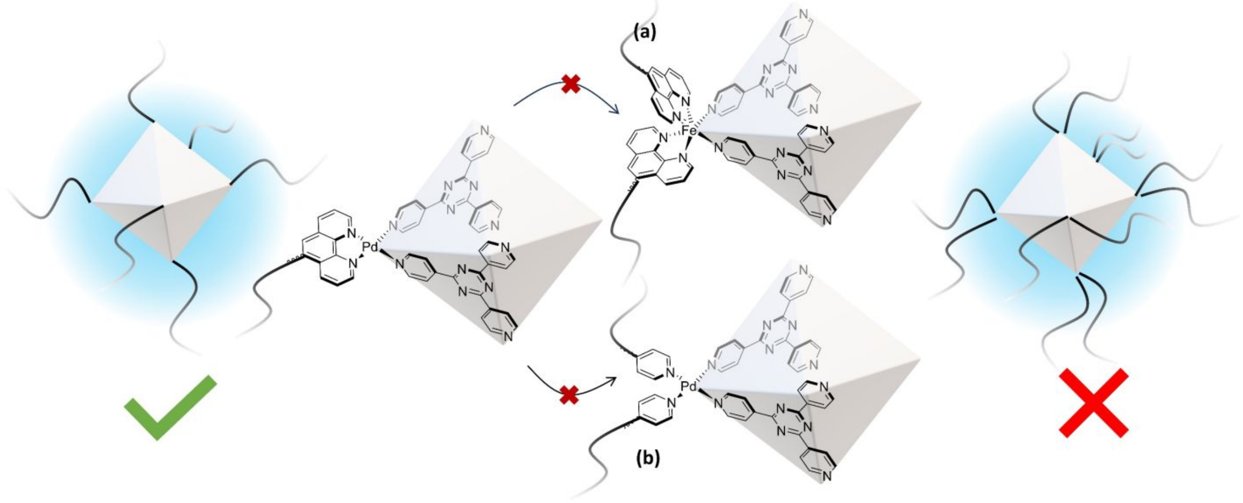

Figure S8. Varying supramolecular parameters, including (a) using metal ions other than Pd^{2+} with different coordination geometry preferences, and (b) using polymer spacers with the monodentate pyridine ligand instead of those with bidentate phenanthroline ligand, was not successful in forming cage-crosslinked polymer networks.

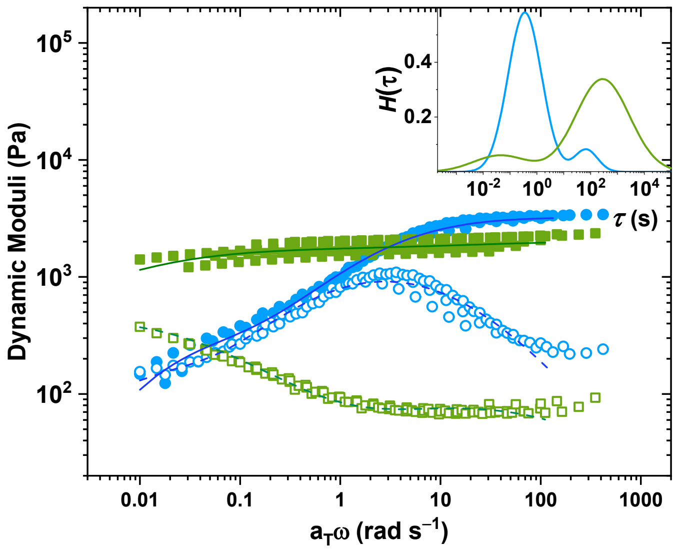

Figure S9. Dynamic moduli master-curves (symbols) and fit of the two-mode generalized Maxwell model (lines) for the cage-crosslinked hydrogels formed by LPhen10k after 5 (blue) and 10 h (green) annealing (main plot) and the corresponding relaxation time spectra (inset).

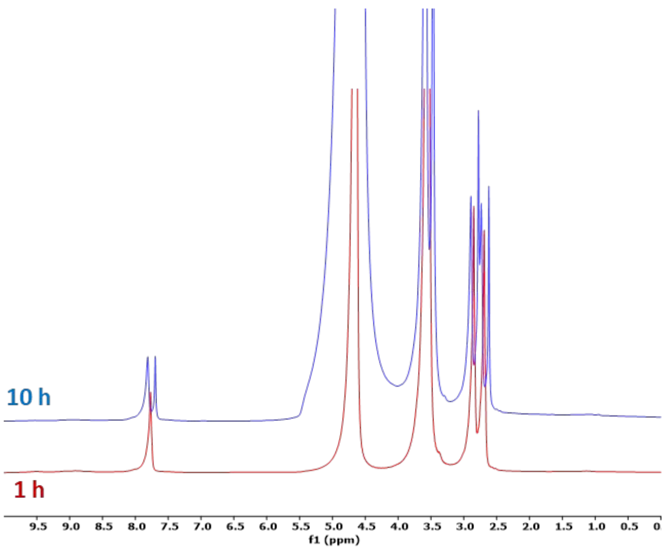

Figure S10. MAS - ^{1}H NMR spectra of hydrogels made by LPhen10k at φ = 80 g L^{-1} after 1 and 10 h annealing time.

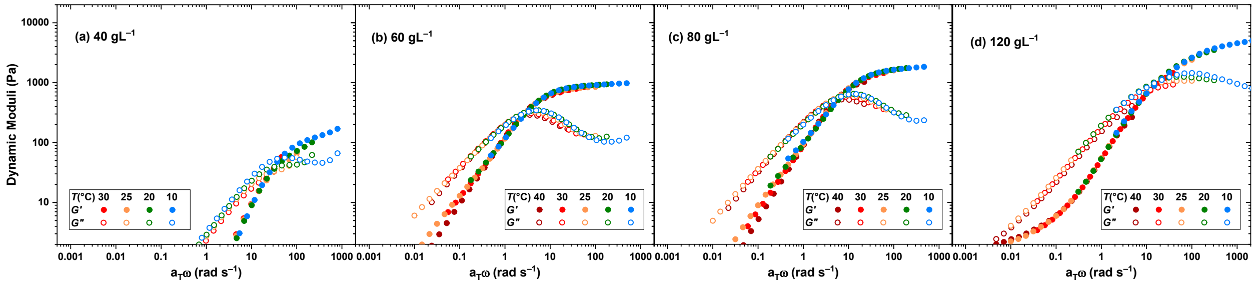

Figure S11. Dynamic moduli master-curves of the cage-crosslinked hydrogels formed by LPhen10k after 1 h annealing, at various concentrations: (a) 40 gL^{-1}, (b) 60 gL^{-1}, (c) 80 gL^{-1}, and (d) 120 gL^{-1}.

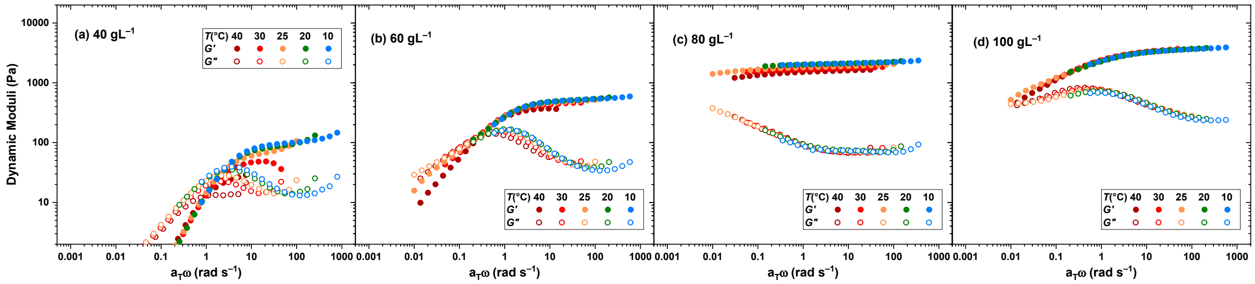

Figure S12. Dynamic moduli master-curves of the cage-crosslinked hydrogels formed by LPhen10k after 10 h annealing, at various concentrations: (a) 40 gL^{-1}, (b) 60 gL^{-1}, (c) 80 gL^{-1}, and (d) 100 gL^{-1}.

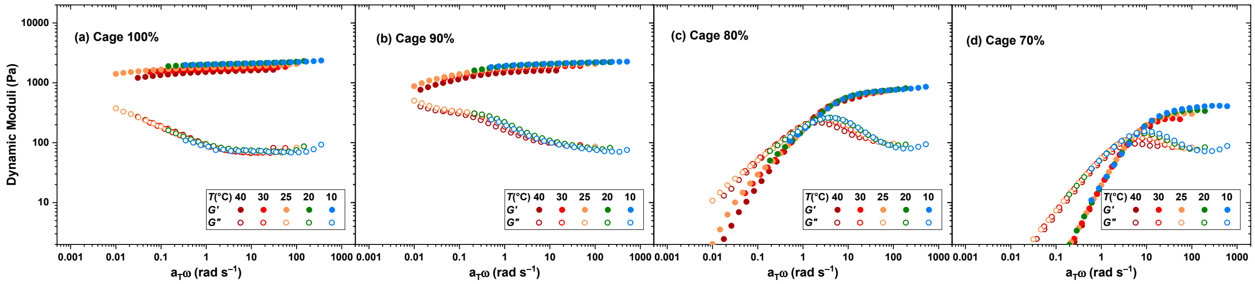

Figure S13. Dynamic moduli master-curves of the cage-crosslinked hydrogels formed by LPhen10k after 10 h annealing, at polymer concentration of φ = 80 gL^{-1}, but various cage contents: (a) 100%, (b) 90%, (c) 80%, and (d) 70%.

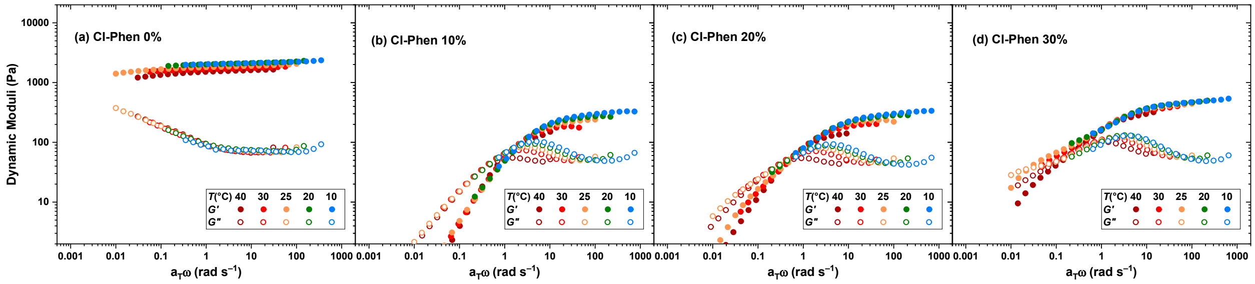

Figure S14. Dynamic moduli master-curves of the cage-crosslinked hydrogels formed by LPhen10k after 10 h annealing, initially at polymer concentration of φ = 80 gL^{-1}, but gradually replacing various fractions of LPhen10k with Cl-Phen: (a) 0%, (b) 10%, (c) 20%, and (d) 30%.

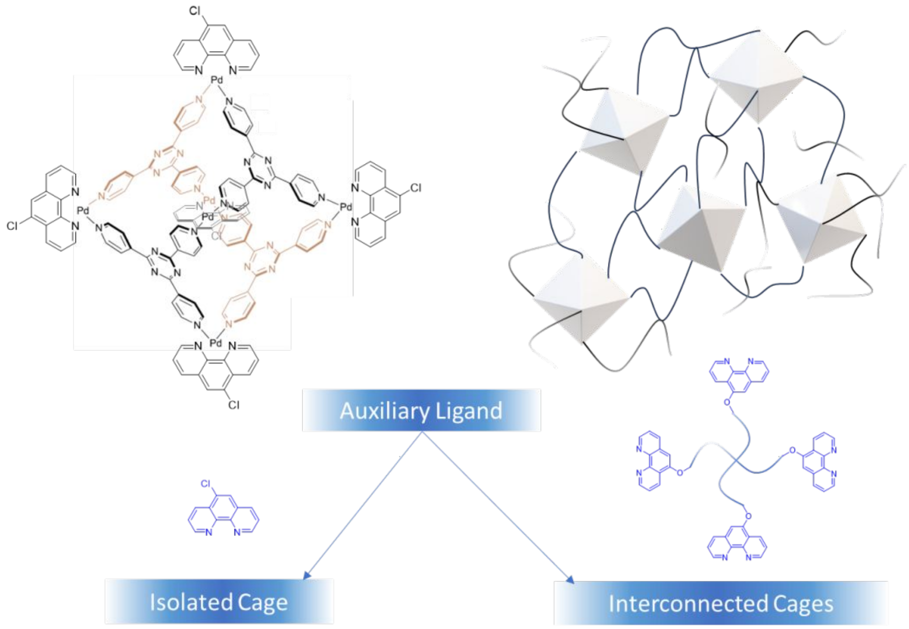

Figure S15. Integration of MOCs as six-fold crosslinks in polymer networks by using polymeric TetraPhen20k ligands instead of Cl-Phen.

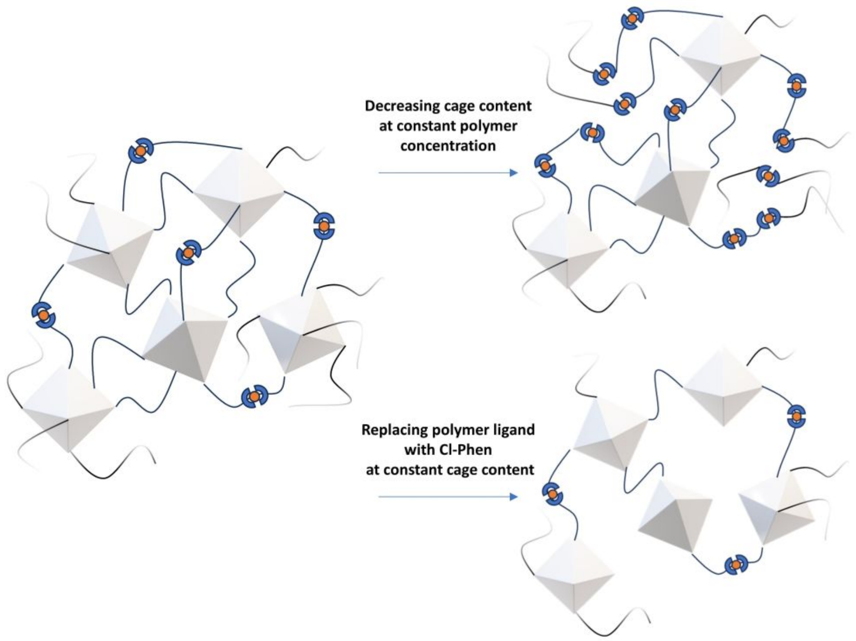

Figure S16. Macromolecular strategies for tuning network connectivity: (a) decreasing cage content at constant polymer concentration, and (b) replacing polymeric ligands with small-molecule ligand at constant cage content.

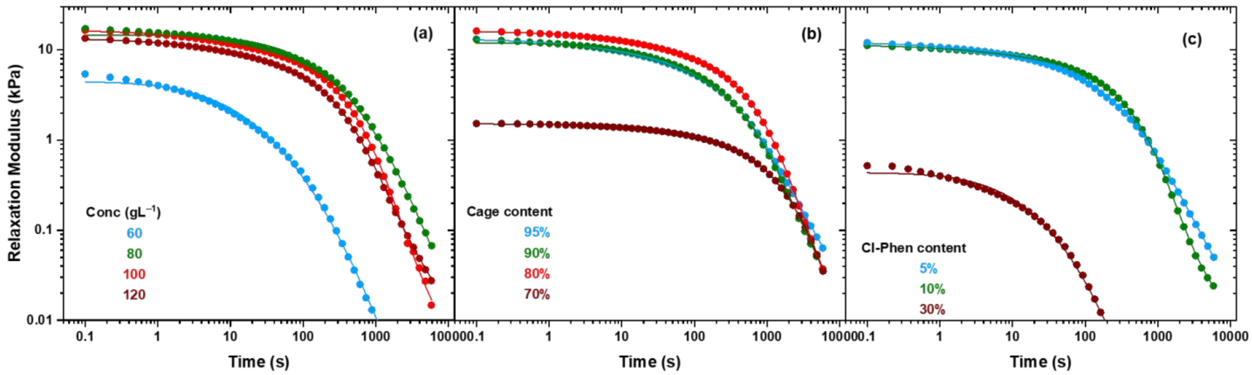

Figure S17. Relaxation modulus (symbols) of the cage-crosslinked hydrogels formed by TetraPhen20k at 25 °C for samples at varying (a) concentration, (b) cage content, and (c) Cl-Phen content. Curves are the fit of generalized Maxwell model.

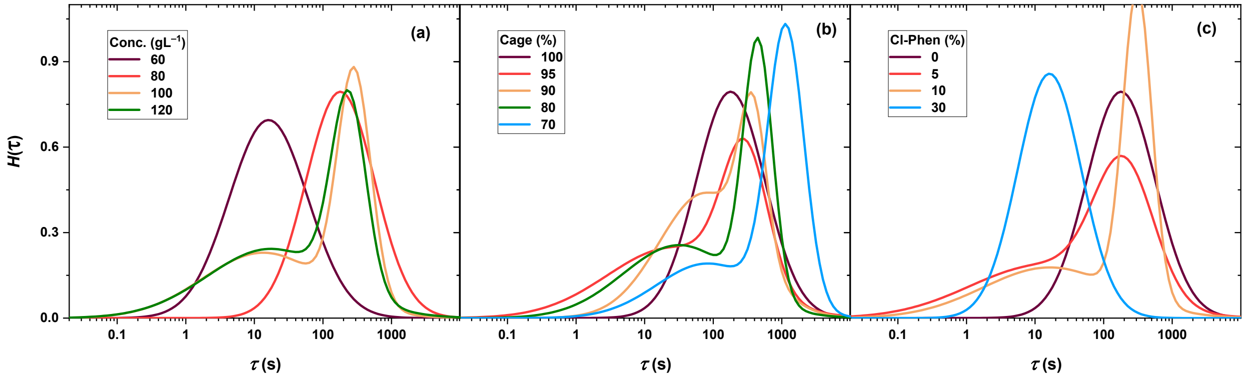

Figure S18. Relaxation time spectra of the cage-crosslinked hydrogels formed by TetraPhen20k at 25 °C for samples at varying (a) concentration, (b) cage content, and (c) Cl-Phen content, obtained from the fit of the generalized Maxwell model on stress-relaxation data shown in Figure S15.

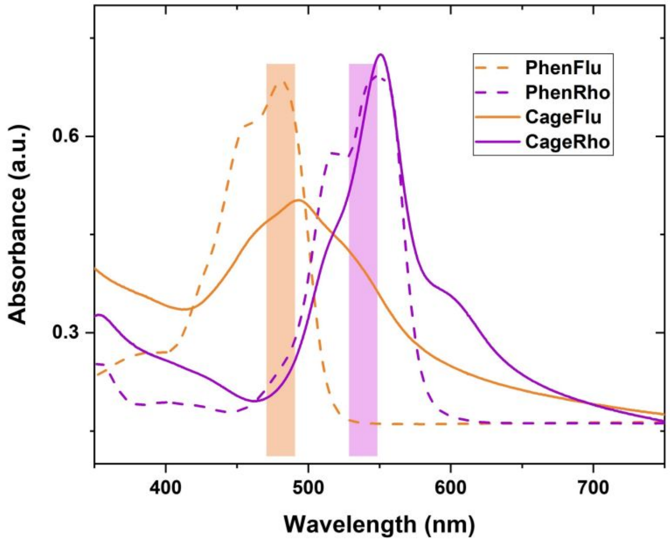

Figure S19. UV-Vis absorption spectra of the dye-labeled ligands and the corresponding dye-labeled cages.

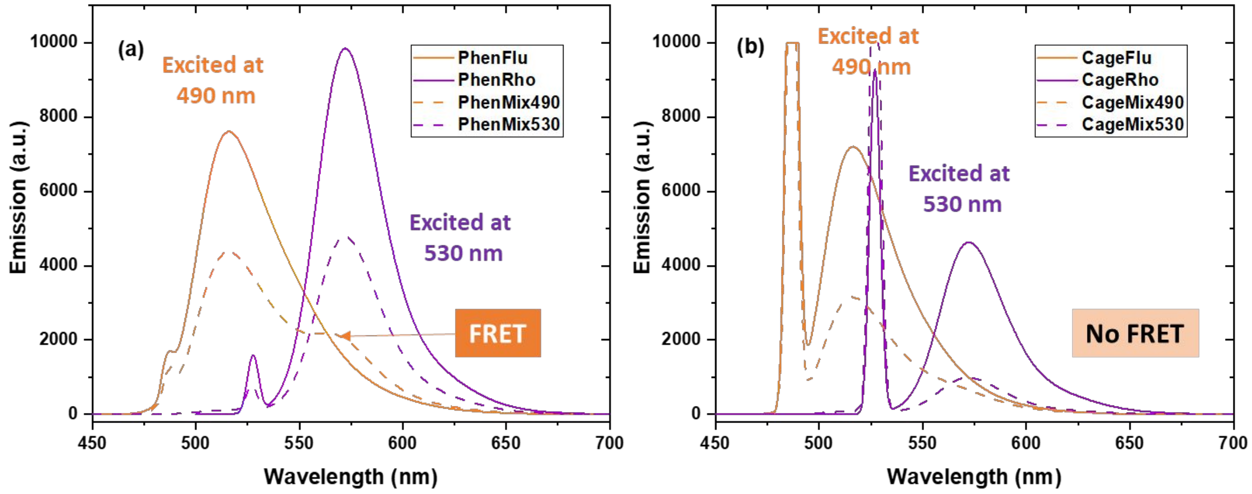

Figure S20. Emission spectra of (a) the dye-labeled ligands (solid lines) and their mixture (dashed lines) excited at various wavelengths, and (b) the corresponding dye-labeled cages (solid lines) and their mixture (dashed lines) excited at various wavelengths. While the mixture of cages does not return FRET upon excitation at 490 nm, the mixture of ligands shows FRET signal at 570 nm, upon excitation at 490 nm.

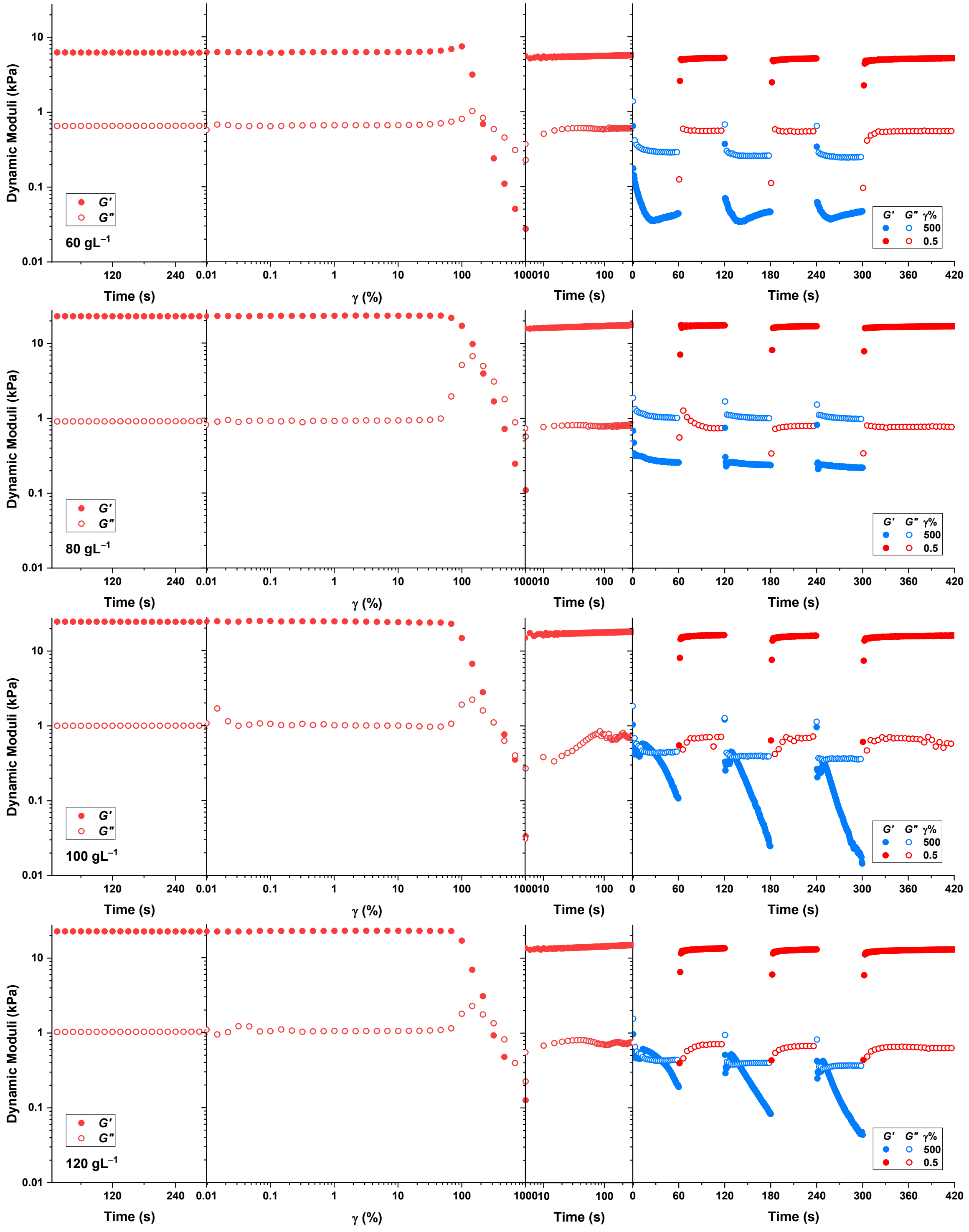

Figure S21. Effect of concentration on self-healing of cage-crosslinked tetraPEG hydrogels(as denoted in legends of the left panel): Amplitude-sweep between two time-sweep measurements at the same oscillation frequency of 10 rad s^{-1}, followed by alternating large- and low-amplitude oscillations for samples made at different polymer concentrations.

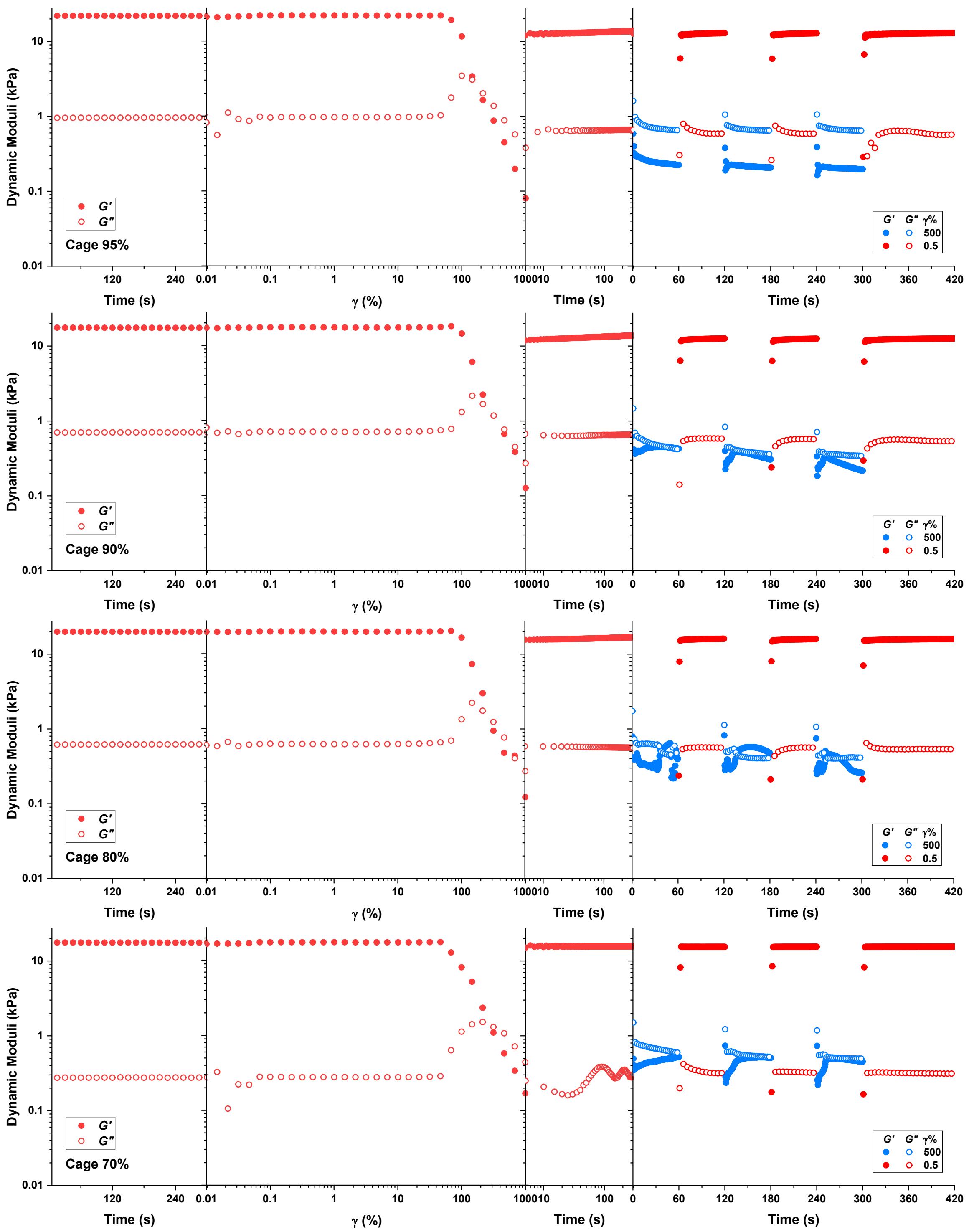

Figure S22. Effect of cage content on self-healing of cage-crosslinked tetraPEG hydrogels (as denoted in legends of the left panel): Amplitude-sweep between two time-sweep measurements at the same oscillation frequency of 10 rad s^{-1}, followed by alternating large- and low-amplitude oscillations for samples made at different cage contents.

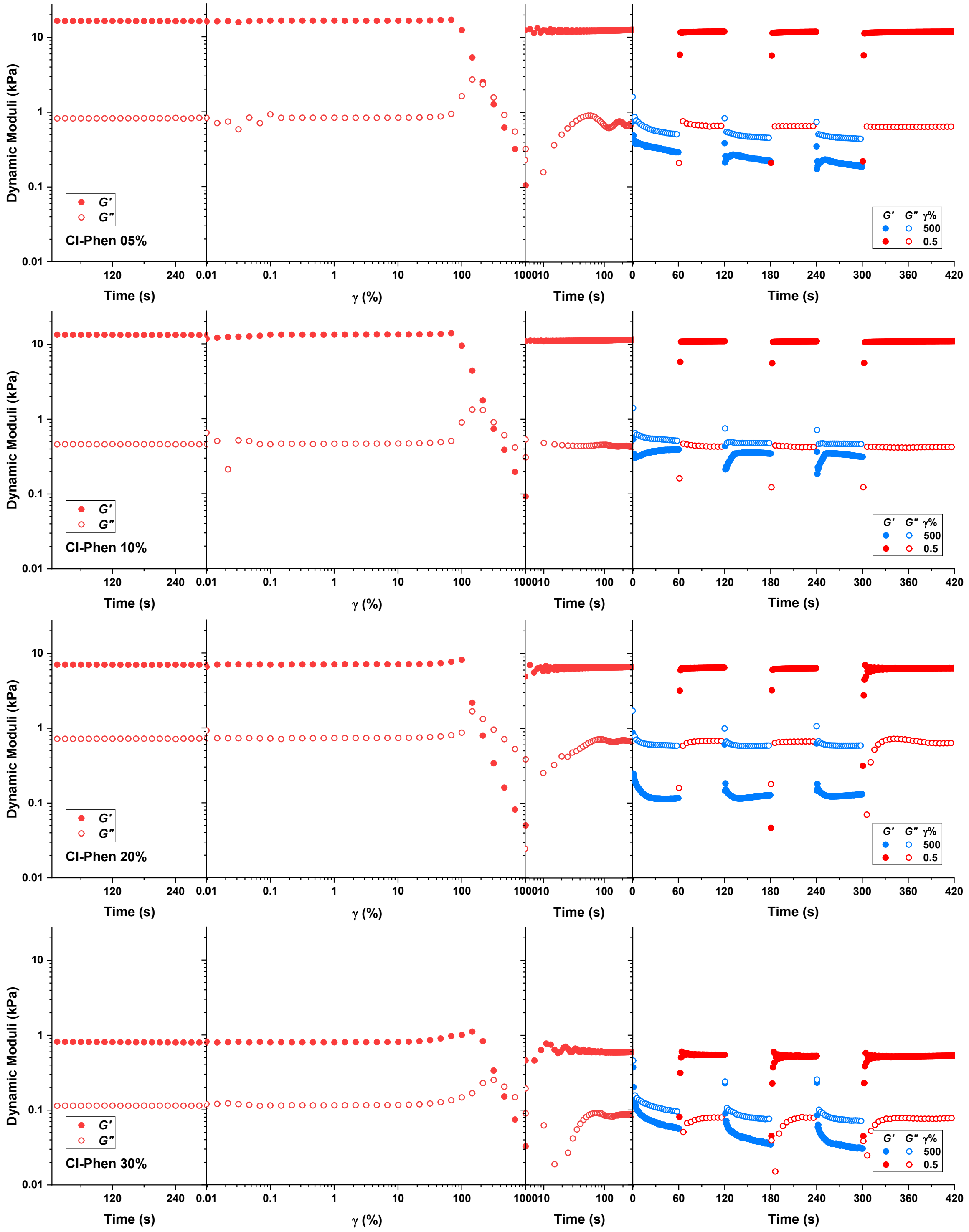

Figure S23. Effect of Cl-Phen content on self-healing of cage-crosslinked tetraPEG hydrogels (as denoted in legends of the left panel): Amplitude-sweep between two time-sweep measurements at the same oscillation frequency of 10 rad s^{-1}, followed by alternating large- and low-amplitude oscillations for samples made at different concentrations of the Cl-Phen ligand.

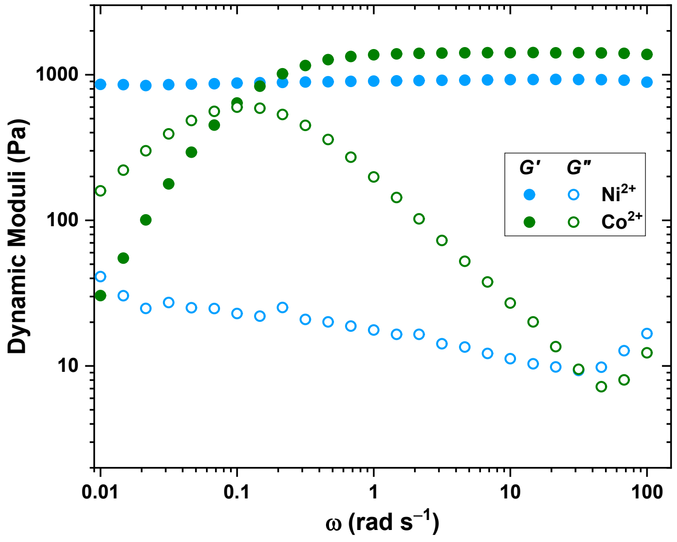

Figure S24. Dynamic storage (filled symbols) and loss (empty symbols) moduli of hydrogels made by tetraPhen20k at φ = 40 g L^{-1} in the presence of Ni^{2+} and Co^{2+} at M^{2+}:Phen ratio of 1:2 at 25 °C.

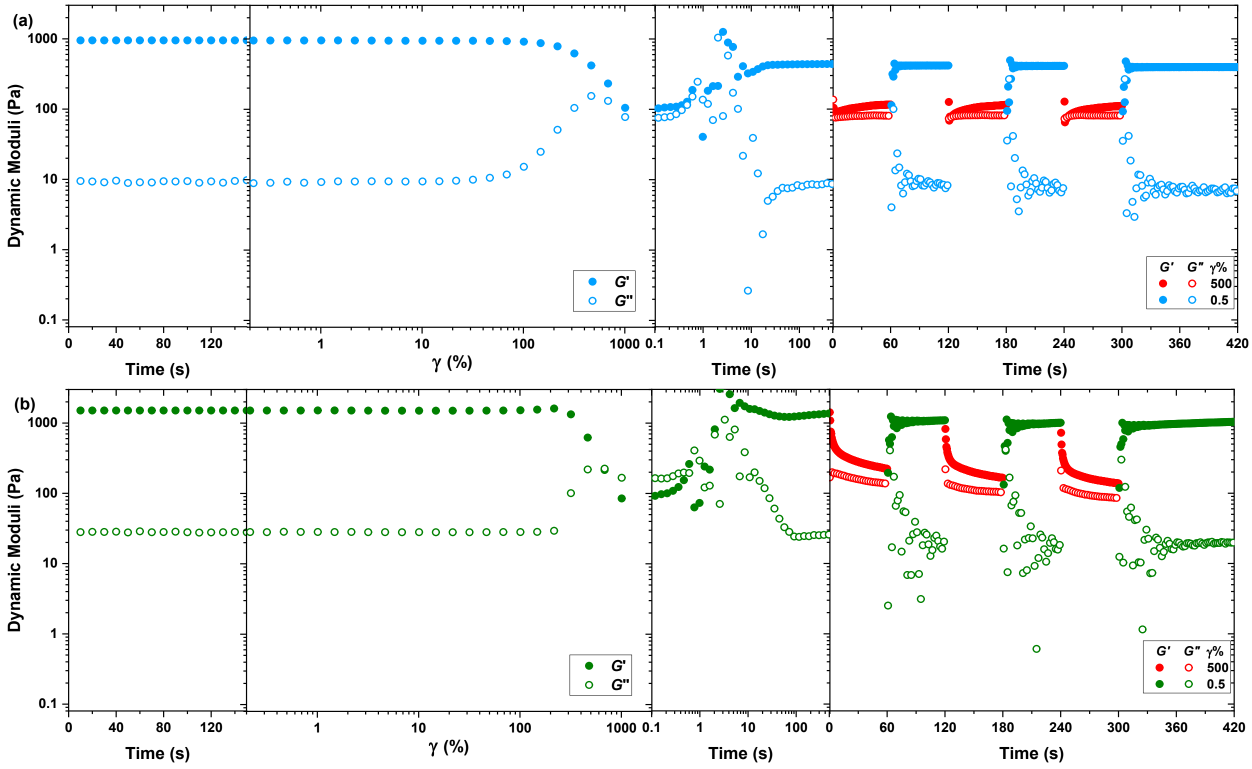

Figure S25. Self-healing of hydrogels made by tetraPhen20k at φ = 40 g L^{-1} in the presence of (a) Ni^{2+} and (b) Co^{2+} at M^{2+}:Phen ratio of 1:2 at 25 °C: Amplitude-sweep between two time-sweep measurements at the same oscillation frequency of 10 rad s^{-1}, followed by alternating large- and low-amplitude oscillations.

Table S3. XYZ coordinates and absolute energies (in a.u.) of all DFT computed species.

20
BPy SCF Done: -495.030324242 A.U.

C	-2.890195	1.153256	-0.000060
C	-3.508223	-0.097727	0.000006
C	-2.683436	-1.229446	0.000064
N	-1.355570	-1.175081	0.000061
C	-0.750542	0.022312	-0.000002
C	-1.496877	1.217406	-0.000065
H	-3.482644	2.071844	-0.000110
H	-4.595706	-0.199980	0.000013
H	-3.129421	-2.232342	0.000115
H	-1.007142	2.191237	-0.000125
C	2.890195	1.153256	0.000061
C	1.496877	1.217406	0.000064
C	0.750542	0.022312	0.000002
N	1.355570	-1.175081	-0.000061
C	2.683436	-1.229446	-0.000065
C	3.508223	-0.097727	-0.000005
H	3.482645	2.071844	0.000111
H	1.007141	2.191237	0.000123
H	3.129421	-2.232342	-0.000116
H	4.595706	-0.199980	-0.000010

270
CAGEBPy+12 SCF Done: -7817.15042306 A.U.

N	0.074988	-5.902506	-1.884423
N	-0.987423	-6.112937	0.214168
C	-1.021692	-5.998460	-1.120460
C	1.251900	-5.930664	-1.244377
N	1.369600	-6.042614	0.085725
C	0.227083	-6.133660	0.779923
C	0.311102	-6.216558	2.277367
C	1.548662	-6.210327	2.941765
C	-0.845327	-6.278050	3.072297
C	1.584506	-6.222214	4.337086
C	-0.728673	-6.287631	4.463186
N	0.465159	-6.239859	5.091300
H	2.482130	-6.197422	2.379143
H	-1.833604	-6.319483	2.614418
H	2.539478	-6.223258	4.868251
H	-1.618680	-6.340888	5.094970
C	2.505903	-5.781324	-2.057688
C	3.772887	-5.764855	-1.451473
C	2.458501	-5.626828	-3.453009
C	4.910776	-5.557852	-2.232910
C	3.640777	-5.424502	-4.166898
N	4.849293	-5.369495	-3.568120
H	3.877304	-5.913119	-0.376679
H	1.507872	-5.664342	-3.984951

H	5.903733	-5.547966	-1.776805
H	3.620790	-5.308209	-5.253263
C	-2.363263	-5.926956	-1.792293
C	-3.555650	-5.988194	-1.052370
C	-2.477000	-5.770779	-3.183590
C	-4.782103	-5.852329	-1.704783
C	-3.739860	-5.642144	-3.764046
N	-4.877420	-5.661522	-3.037660
H	-3.533658	-6.141925	0.026495
H	-1.589142	-5.749950	-3.815520
H	-5.718435	-5.903339	-1.143725
H	-3.844979	-5.525535	-4.845459
Pd	-6.767214	-5.315677	-3.958983
N	-4.794773	1.571909	-3.598040
N	-5.857213	1.361205	-1.499484
C	-5.500905	0.875940	-2.696620
C	-4.448091	2.818680	-3.250287
N	-4.765831	3.375253	-2.073505
C	-5.472918	2.615427	-1.226020
C	-5.812830	3.183895	0.122166
C	-5.413325	4.478883	0.491519
C	-6.521292	2.433154	1.074786
C	-6.760923	2.971202	2.340246
N	-6.336088	4.201603	2.697614
H	-4.889781	5.122439	-0.215446
H	-6.887140	1.434541	0.836048
H	-5.391015	5.954989	2.077052
H	-7.315231	2.401638	3.090208
C	-3.618060	3.619197	-4.212846
C	-3.189159	4.920137	-3.902014
C	-3.217530	3.088338	-5.450093
C	-2.364371	5.607895	-4.793640
C	-2.391770	3.837930	-6.289476
N	-1.952376	5.071479	-5.961795
H	-3.494617	5.399289	-2.971871
H	-3.545741	2.097032	-5.762601
H	-2.027078	6.622630	-4.568587
H	-2.076393	3.441005	-7.257509
C	-5.872792	-0.541362	-3.027160
C	-6.581718	-1.342526	-2.116897
C	-5.503952	-1.128235	-4.248871
C	-6.851478	-2.675124	-2.432737
C	-5.810158	-2.468061	-4.492810
N	-6.455967	-3.239906	-3.593208
H	-6.924588	-0.934319	-1.166175
H	-4.981742	-0.547988	-5.009586
H	-7.406562	-3.309439	-1.737310
H	-5.534697	-2.937245	-5.440480
Pd	-0.577254	6.109634	-7.215219
Pd	0.577104	-6.109543	7.215068
Pd	6.603193	-4.913133	-4.688627
N	1.480298	0.893471	5.949788
N	0.214588	2.836830	5.504454

C	1.381535	2.188382	5.619709
C	0.328908	0.245710	6.173917
N	-0.876685	0.822850	6.079026
C	-0.892005	2.120281	5.744347
C	-2.223793	2.796011	5.583121
C	-2.318030	4.144286	5.200631
C	-3.426593	2.098905	5.783961
C	-3.573525	4.719170	4.996817
C	-4.644680	2.742825	5.560458
N	-4.722046	4.027424	5.153124
H	-1.420681	4.747331	5.062181
H	-3.419163	1.060038	6.113765
H	-3.663350	5.768237	4.704426
H	-5.588813	2.215587	5.717737
C	0.390952	-1.218533	6.503208
C	-0.776208	-1.969835	6.718323
C	1.617441	-1.898439	6.583728
C	-0.681276	-3.341834	6.956980
C	1.631585	-3.272828	6.826835
N	0.501178	-3.991994	6.991331
H	-1.756008	-1.492558	6.701948
H	2.559113	-1.363800	6.459348
H	-1.579721	-3.938822	7.131639
H	2.577863	-3.814706	6.897722
C	2.645180	2.941919	5.316994
C	2.616616	4.291905	4.930004
C	3.902200	2.318775	5.384864
C	3.806065	4.940028	4.593231
C	5.048285	3.033555	5.032907
N	5.004122	4.318880	4.622937
H	1.675068	4.839640	4.890886
H	3.992617	1.282620	5.710881
H	3.800779	5.991450	4.295618
H	6.033790	2.564441	5.086009
Pd	6.767085	5.314746	3.959713
N	5.567650	1.705078	-2.122407
N	4.301577	3.648286	-2.567471
C	5.140834	2.934833	-1.804468
C	5.113090	1.195625	-3.275389
N	4.272850	1.845272	-4.092405
C	3.891849	3.070048	-3.704719
C	2.915753	3.816341	-4.568772
C	2.444225	5.089280	-4.207779
C	2.417443	3.259392	-5.758115
C	-5.690362	4.947887	1.776655
C	1.461796	3.955252	-6.500291
N	0.985653	5.160227	-6.121629
H	2.818281	5.588190	-3.313781
H	2.770033	2.289436	-6.108733
H	1.068783	3.537699	-7.430499
C	5.530840	-0.197970	-3.649099
C	6.382373	-0.951915	-2.824778
C	5.065582	-0.810369	-4.824478

C	6.695185	-2.267058	-3.172418
C	5.422803	-2.130298	-5.104598
N	6.208898	-2.858750	-4.283950
H	6.802690	-0.520511	-1.916326
H	4.428812	-0.265298	-5.521249
H	7.360514	-2.864489	-2.544308
H	5.073297	-2.618617	-6.017554
C	5.590653	3.526803	-0.499278
C	5.157133	4.796806	-0.084323
C	6.441852	2.822599	0.368232
C	5.543679	5.285475	1.164719
C	6.784955	3.377847	1.602044
N	6.328905	4.581852	2.007591
H	4.522475	5.405841	-0.728024
H	6.838435	1.846821	0.087805
H	5.219049	6.273787	1.499328
H	7.450368	2.844677	2.285458
Pd	-6.602970	4.913785	4.687923
C	1.487658	5.723358	-5.002276
H	1.115299	6.716042	-4.737659
C	10.823160	7.729163	2.978068
C	10.175051	7.067118	1.931834
C	9.002440	6.366041	2.201872
N	8.472329	6.305159	3.439006
C	9.092444	6.946562	4.474883
C	10.272238	7.665032	4.260045
H	11.745903	8.289343	2.802581
H	10.571154	7.091270	0.914699
H	8.473940	5.839724	1.407843
H	10.766912	8.176710	5.086475
C	8.198286	7.210332	8.155261
C	8.902031	7.394097	6.962753
C	8.430468	6.815644	5.780687
N	7.284467	6.070236	5.782182
C	6.604447	5.892095	6.931539
C	7.028885	6.445390	8.137016
H	8.561253	7.659894	9.083808
H	9.816314	7.988725	6.961551
H	5.697061	5.291041	6.884464
H	6.448237	6.276681	9.046100
C	0.848184	-6.361443	12.022078
C	0.778349	-5.099186	11.426444
C	0.699680	-5.012446	10.038736
N	0.688758	-6.105826	9.251599
C	0.756525	-7.348935	9.816467
C	0.836830	-7.493966	11.204550
H	0.910832	-6.467306	13.108779
H	0.784469	-4.188001	12.027900
H	0.644008	-4.043230	9.544431
H	0.890761	-8.485677	11.655219
C	0.774238	-10.811931	8.260309
C	0.798241	-9.817301	9.240740
C	0.737907	-8.471406	8.867685

N	0.655451	-8.120015	7.549083
C	0.632148	-9.078153	6.602178
C	0.689645	-10.433359	6.917743
H	0.821139	-11.866881	8.544797
H	0.864055	-10.097630	10.292686
H	0.565712	-8.751009	5.565152
H	0.668149	-11.178033	6.119592
C	-11.073653	-5.075689	-6.113377
C	-10.403252	-6.281415	-5.894569
C	-9.155885	-6.279045	-5.263444
N	-8.585790	-5.104514	-4.857899
C	-9.231076	-3.940663	-5.068286
C	-10.475034	-3.885302	-5.691954
H	-12.049784	-5.069929	-6.606518
H	-10.858042	-7.218439	-6.218081
H	-8.738447	-3.030358	-4.728445
H	-10.964101	-2.920942	-5.843184
C	-7.948710	-9.869830	-5.011647
C	-6.729748	-9.631430	-4.371128
C	-6.376453	-8.320427	-4.061443
N	-7.171582	-7.274319	-4.359168
C	-8.367732	-7.488231	-4.985590
C	-8.771847	-8.784146	-5.319587
H	-8.258394	-10.886409	-5.269941
H	-6.056233	-10.450820	-4.112239
H	-5.433064	-8.101872	-3.562363
H	-9.726055	-8.954791	-5.819451
C	-7.681115	6.736036	9.019360
C	-8.519766	6.873176	7.910782
C	-8.145599	6.319505	6.682893
N	-6.963731	5.643827	6.557868
C	-6.153720	5.510601	7.626201
C	-6.477127	6.041983	8.872189
H	-7.967366	7.166575	9.983076
H	-9.462625	7.412212	8.010528
H	-5.222635	4.964405	7.479352
H	-5.792301	5.911427	9.712435
C	-10.878722	7.077856	4.159149
C	-10.309477	6.451995	3.046846
C	-9.074669	5.823249	3.186070
N	-8.410824	5.798667	4.358204
C	-8.952080	6.405730	5.457069
C	-10.189111	7.051510	5.373576
H	-11.846928	7.581188	4.086445
H	-10.814275	6.448782	2.078748
H	-8.604787	5.326596	2.337931
H	-10.620975	7.535682	6.250257
C	7.934986	-9.390723	-5.881103
C	6.780925	-9.224406	-5.110757
C	6.385187	-7.936207	-4.759484
N	7.078629	-6.844904	-5.138155
C	8.210086	-6.988110	-5.891997
C	8.652768	-8.258263	-6.272796

H	8.275390	-10.387522	-6.175330
H	6.190199	-10.082156	-4.783108
H	5.490395	-7.773518	-4.159734
H	9.555447	-8.372503	-6.874144
C	10.626684	-4.419989	-7.298832
C	10.057889	-5.663319	-7.013015
C	8.889013	-5.734422	-6.249513
N	8.297007	-4.595290	-5.779513
C	8.844346	-3.395260	-6.054363
C	10.007189	-3.266712	-6.809818
H	11.541025	-4.356756	-7.895518
H	10.530170	-6.572080	-7.387978
H	8.337930	-2.515424	-5.659116
H	10.417954	-2.275054	-7.009646
C	-3.719594	8.498698	-9.983271
C	-4.219539	7.660560	-8.982999
C	-3.322509	6.964722	-8.176393
N	-1.988282	7.073183	-8.328924
C	-1.480902	7.888329	-9.302107
C	-2.336069	8.610013	-10.140025
H	-4.394641	9.060273	-10.635189
H	-5.293734	7.544328	-8.826024
H	-3.680826	6.303667	-7.388071
H	-1.931287	9.260281	-10.916431
C	2.096974	8.675700	-10.298714
C	0.700428	8.702465	-10.304673
C	-0.013881	7.932992	-9.381670
N	0.644148	7.153276	-8.471717
C	1.991160	7.126356	-8.464641
C	2.751946	7.872637	-9.361138
H	2.662342	9.275064	-11.017862
H	0.175474	9.324517	-11.030606
H	2.472021	6.490834	-7.721875
H	3.841871	7.822224	-9.321556

318

CAGEBPy+NO3T SCF Done: -11183.4790503 A.U.

N	4.029026	-2.360221	-4.029026
N	2.360221	-4.029026	-4.029026
C	2.935247	-2.935247	-4.541042
C	4.541042	-2.935247	-2.935247
N	4.029026	-4.029026	-2.360221
C	2.935247	-4.541042	-2.935247
C	2.293106	-5.720218	-2.293106
C	2.821216	-6.273692	-1.117695
C	1.117695	-6.273692	-2.821216
C	2.155347	-7.325727	-0.494307
C	0.494307	-7.325727	-2.155347
N	1.009444	-7.819345	-1.009444
H	3.734804	-5.876209	-0.677008
H	0.677008	-5.876209	-3.734804
H	2.509585	-7.770206	0.449034
H	-0.449034	-7.770206	-2.509585
C	5.720218	-2.293106	-2.293106

C	6.273692	-2.821216	-1.117695
C	6.273692	-1.117695	-2.821216
C	7.325727	-2.155347	-0.494307
C	7.325727	-0.494307	-2.155347
N	7.819345	-1.009444	-1.009444
H	5.876209	-3.734804	-0.677008
H	5.876209	-0.677008	-3.734804
H	7.770206	-2.509585	0.449034
H	7.770206	0.449034	-2.509585
C	2.293106	-2.293106	-5.720218
C	1.117695	-2.821216	-6.273692
C	2.821216	-1.117695	-6.273692
C	0.494307	-2.155347	-7.325727
C	2.155347	-0.494307	-7.325727
N	1.009444	-1.009444	-7.819345
H	0.677008	-3.734804	-5.876209
H	3.734804	-0.677008	-5.876209
H	-0.449034	-2.509585	-7.770206
H	2.509585	0.449034	-7.770206
Pd	0.000000	0.000000	-9.297090
N	-2.360221	4.029026	-4.029026
N	-4.029026	2.360221	-4.029026
C	-2.935247	2.935247	-4.541042
C	-2.935247	4.541042	-2.935247
N	-4.029026	4.029026	-2.360221
C	-4.541042	2.935247	-2.935247
C	-5.720218	2.293106	-2.293106
C	-6.273692	2.821216	-1.117695
C	-6.273692	1.117695	-2.821216
C	-7.325727	0.494307	-2.155347
N	-7.819345	1.009444	-1.009444
H	-5.876209	3.734804	-0.677008
H	-5.876209	0.677008	-3.734804
H	-7.770206	2.509585	0.449034
H	-7.770206	-0.449034	-2.509585
C	-2.293106	5.720218	-2.293106
C	-2.821216	6.273692	-1.117695
C	-1.117695	6.273692	-2.821216
C	-2.155347	7.325727	-0.494307
C	-0.494307	7.325727	-2.155347
N	-1.009444	7.819345	-1.009444
H	-3.734804	5.876209	-0.677008
H	-0.677008	5.876209	-3.734804
H	-2.509585	7.770206	0.449034
H	0.449034	7.770206	-2.509585
C	-2.293106	2.293106	-5.720218
C	-2.821216	1.117695	-6.273692
C	-1.117695	2.821216	-6.273692
C	-2.155347	0.494307	-7.325727
C	-0.494307	2.155347	-7.325727
N	-1.009444	1.009444	-7.819345
H	-3.734804	0.677008	-5.876209
H	-0.677008	3.734804	-5.876209

H	-2.509585	-0.449034	-7.770206
H	0.449034	2.509585	-7.770206
Pd	0.000000	9.297090	-0.000000
Pd	-0.000000	-9.297090	0.000000
Pd	9.297090	-0.000000	0.000000
N	-2.360221	-4.029026	4.029026
N	-4.029026	-2.360221	4.029026
C	-2.935247	-2.935247	4.541042
C	-2.935247	-4.541042	2.935247
N	-4.029026	-4.029026	2.360221
C	-4.541042	-2.935247	2.935247
C	-5.720218	-2.293106	2.293106
C	-6.273692	-1.117695	2.821216
C	-6.273692	-2.821216	1.117695
C	-7.325727	-0.494307	2.155347
C	-7.325727	-2.155347	0.494307
N	-7.819345	-1.009444	1.009444
H	-5.876209	-0.677008	3.734804
H	-5.876209	-3.734804	0.677008
H	-7.770206	0.449034	2.509585
H	-7.770206	-2.509585	-0.449034
C	-2.293106	-5.720218	2.293106
C	-2.821216	-6.273692	1.117695
C	-1.117695	-6.273692	2.821216
C	-2.155347	-7.325727	0.494307
C	-0.494307	-7.325727	2.155347
N	-1.009444	-7.819345	1.009444
H	-3.734804	-5.876209	0.677008
H	-0.677008	-5.876209	3.734804
H	-2.509585	-7.770206	-0.449034
H	0.449034	-7.770206	2.509585
C	-2.293106	-2.293106	5.720218
C	-2.821216	-1.117695	6.273692
C	-1.117695	-2.821216	6.273692
C	-2.155347	-0.494307	7.325727
C	-0.494307	-2.155347	7.325727
N	-1.009444	-1.009444	7.819345
H	-3.734804	-0.677008	5.876209
H	-0.677008	-3.734804	5.876209
H	-2.509585	0.449034	7.770206
H	0.449034	-2.509585	7.770206
Pd	0.000000	0.000000	9.297090
N	4.029026	2.360221	4.029026
N	2.360221	4.029026	4.029026
C	2.935247	2.935247	4.541042
C	4.541042	2.935247	2.935247
N	4.029026	4.029026	2.360221
C	2.935247	4.541042	2.935247
C	2.293106	5.720218	2.293106
C	1.117695	6.273692	2.821216
C	2.821216	6.273692	1.117695
C	-7.325727	2.155347	-0.494307
C	2.155347	7.325727	0.494307

N	1.009444	7.819345	1.009444
H	0.677008	5.876209	3.734804
H	3.734804	5.876209	0.677008
H	2.509585	7.770206	-0.449034
C	5.720218	2.293106	2.293106
C	6.273692	1.117695	2.821216
C	6.273692	2.821216	1.117695
C	7.325727	0.494307	2.155347
C	7.325727	2.155347	0.494307
N	7.819345	1.009444	1.009444
H	5.876209	0.677008	3.734804
H	5.876209	3.734804	0.677008
H	7.770206	-0.449034	2.509585
H	7.770206	2.509585	-0.449034
C	2.293106	2.293106	5.720218
C	1.117695	2.821216	6.273692
C	2.821216	1.117695	6.273692
C	0.494307	2.155347	7.325727
C	2.155347	0.494307	7.325727
N	1.009444	1.009444	7.819345
H	0.677008	3.734804	5.876209
H	3.734804	0.677008	5.876209
H	-0.449034	2.509585	7.770206
H	2.509585	-0.449034	7.770206
Pd	-9.297090	0.000000	-0.000000
C	0.494307	7.325727	2.155347
H	-0.449034	7.770206	2.509585
C	2.056645	2.056645	13.112713
C	2.464759	2.464759	11.844389
C	1.873267	1.873267	10.730990
N	0.933515	0.933515	10.846412
C	0.521240	0.521240	12.067498
C	1.074418	1.074418	13.224615
H	2.497115	2.497115	14.009642
H	3.229806	3.229806	11.706657
H	2.160471	2.160471	9.721252
H	0.741430	0.741430	14.206545
C	-2.056645	-2.056645	13.112713
C	-1.074418	-1.074418	13.224615
C	-0.521240	-0.521240	12.067498
N	-0.933515	-0.933515	10.846412
C	-1.873267	-1.873267	10.730990
C	-2.464759	-2.464759	11.844389
H	-2.497115	-2.497115	14.009642
H	-0.741430	-0.741430	14.206545
H	-2.160471	-2.160471	9.721252
H	-3.229806	-3.229806	11.706657
C	-2.056645	-13.112713	2.056645
C	-2.464759	-11.844389	2.464759
C	-1.873267	-10.730990	1.873267
N	-0.933515	-10.846412	0.933515
C	-0.521240	-12.067498	0.521240
C	-1.074418	-13.224615	1.074418

H	-2.497115	-14.009642	2.497115
H	-3.229806	-11.706657	3.229806
H	-2.160471	-9.721252	2.160471
H	-0.741430	-14.206545	0.741430
C	2.056645	-13.112713	-2.056645
C	1.074418	-13.224615	-1.074418
C	0.521240	-12.067498	-0.521240
N	0.933515	-10.846412	-0.933515
C	1.873267	-10.730990	-1.873267
C	2.464759	-11.844389	-2.464759
H	2.497115	-14.009642	-2.497115
H	0.741430	-14.206545	-0.741430
H	2.160471	-9.721252	-2.160471
H	3.229806	-11.706657	-3.229806
C	-2.056645	2.056645	-13.112713
C	-1.074418	1.074418	-13.224615
C	-0.521240	0.521240	-12.067498
N	-0.933515	0.933515	-10.846412
C	-1.873267	1.873267	-10.730990
C	-2.464759	2.464759	-11.844389
H	-2.497115	2.497115	-14.009642
H	-0.741430	0.741430	-14.206545
H	-2.160471	2.160471	-9.721252
H	-3.229806	3.229806	-11.706657
C	2.056645	-2.056645	-13.112713
C	2.464759	-2.464759	-11.844389
C	1.873267	-1.873267	-10.730990
N	0.933515	-0.933515	-10.846412
C	0.521240	-0.521240	-12.067498
C	1.074418	-1.074418	-13.224615
H	2.497115	-2.497115	-14.009642
H	3.229806	-3.229806	-11.706657
H	2.160471	-2.160471	-9.721252
H	0.741430	-0.741430	-14.206545
C	-13.112713	-2.056645	2.056645
C	-13.224615	-1.074418	1.074418
C	-12.067498	-0.521240	0.521240
N	-10.846412	-0.933515	0.933515
C	-10.730990	-1.873267	1.873267
C	-11.844389	-2.464759	2.464759
H	-14.009642	-2.497115	2.497115
H	-14.206545	-0.741430	0.741430
H	-9.721252	-2.160471	2.160471
H	-11.706657	-3.229806	3.229806
C	-13.112713	2.056645	-2.056645
C	-11.844389	2.464759	-2.464759
C	-10.730990	1.873267	-1.873267
N	-10.846412	0.933515	-0.933515
C	-12.067498	0.521240	-0.521240
C	-13.224615	1.074418	-1.074418
H	-14.009642	2.497115	-2.497115
H	-11.706657	3.229806	-3.229806
H	-9.721252	2.160471	-2.160471

H	-14.206545	0.741430	-0.741430
C	13.112713	-2.056645	-2.056645
C	11.844389	-2.464759	-2.464759
C	10.730990	-1.873267	-1.873267
N	10.846412	-0.933515	-0.933515
C	12.067498	-0.521240	-0.521240
C	13.224615	-1.074418	-1.074418
H	14.009642	-2.497115	-2.497115
H	11.706657	-3.229806	-3.229806
H	9.721252	-2.160471	-2.160471
H	14.206545	-0.741430	-0.741430
C	13.112713	2.056645	2.056645
C	13.224615	1.074418	1.074418
C	12.067498	0.521240	0.521240
N	10.846412	0.933515	0.933515
C	10.730990	1.873267	1.873267
C	11.844389	2.464759	2.464759
H	14.009642	2.497115	2.497115
H	14.206545	0.741430	0.741430
H	9.721252	2.160471	2.160471
H	11.706657	3.229806	3.229806
C	-2.056645	13.112713	-2.056645
C	-2.464759	11.844389	-2.464759
C	-1.873267	10.730990	-1.873267
N	-0.933515	10.846412	-0.933515
C	-0.521240	12.067498	-0.521240
C	-1.074418	13.224615	-1.074418
H	-2.497115	14.009642	-2.497115
H	-3.229806	11.706657	-3.229806
H	-2.160471	9.721252	-2.160471
H	-0.741430	14.206545	-0.741430
C	2.056645	13.112713	2.056645
C	1.074418	13.224615	1.074418
C	0.521240	12.067498	0.521240
N	0.933515	10.846412	0.933515
C	1.873267	10.730990	1.873267
C	2.464759	11.844389	2.464759
H	2.497115	14.009642	2.497115
H	0.741430	14.206545	0.741430
H	2.160471	9.721252	2.160471
H	3.229806	11.706657	3.229806
O	1.257532	2.793209	-10.299643
O	-1.257532	-2.793209	-10.299643
O	10.299643	-1.257532	2.793209
O	10.299643	2.793209	-1.257532
O	1.257532	-2.793209	10.299643
O	-2.793209	1.257532	10.299643
O	1.257532	10.299643	-2.793209
O	-2.793209	10.299643	1.257532
O	-2.793209	-10.299643	-1.257532
O	1.257532	-10.299643	2.793209
O	-10.299643	2.793209	1.257532
O	-10.299643	-2.793209	-1.257532

N	-9.707449	2.120592	2.120592
O	-8.457562	2.285193	2.285193
O	-10.299643	1.257532	2.793209
N	-9.707449	-2.120592	-2.120592
O	-8.457562	-2.285193	-2.285193
O	-10.299643	-1.257532	-2.793209
N	-2.120592	2.120592	9.707449
O	-2.285193	2.285193	8.457562
O	-1.257532	2.793209	10.299643
N	2.120592	-2.120592	9.707449
O	2.285193	-2.285193	8.457562
O	2.793209	-1.257532	10.299643
N	-2.120592	-9.707449	-2.120592
O	-1.257532	-10.299643	-2.793209
O	-2.285193	-8.457562	-2.285193
N	2.120592	-9.707449	2.120592
O	2.285193	-8.457562	2.285193
O	2.793209	-10.299643	1.257532
N	9.707449	-2.120592	2.120592
O	10.299643	-2.793209	1.257532
O	8.457562	-2.285193	2.285193
N	9.707449	2.120592	-2.120592
O	8.457562	2.285193	-2.285193
O	10.299643	1.257532	-2.793209
N	2.120592	2.120592	-9.707449
O	2.793209	1.257532	-10.299643
O	2.285193	2.285193	-8.457562
N	-2.120592	-2.120592	-9.707449
O	-2.793209	-1.257532	-10.299643
O	-2.285193	-2.285193	-8.457562
N	-2.120592	9.707449	2.120592
O	-1.257532	10.299643	2.793209
O	-2.285193	8.457562	2.285193
N	2.120592	9.707449	-2.120592
O	2.285193	8.457562	-2.285193
O	2.793209	10.299643	-1.257532

270
CAGEBPyTd+12 SCF Done: -7817.15041772 A.U.

N	-4.046374	-2.376474	4.046374
N	-2.376474	-4.046374	4.046374
C	-2.953741	-2.953741	4.564511
C	-4.564511	-2.953741	2.953741
N	-4.046374	-4.046374	2.376474
C	-2.953741	-4.564511	2.953741
C	-2.314085	-5.763626	2.314085
C	-2.843415	-6.339680	1.147466
C	-1.147466	-6.339680	2.843415
C	-2.183965	-7.412426	0.545268
C	-0.545268	-7.412426	2.183965
N	-1.040683	-7.931590	1.040683
H	-3.764899	-5.958494	0.707428
H	-0.707428	-5.958494	3.764899
H	-2.586335	-7.874764	-0.359416

H	0.359416	-7.874764	2.586335
C	-5.763626	-2.314085	2.314085
C	-6.339680	-2.843415	1.147466
C	-6.339680	-1.147466	2.843415
C	-7.412426	-2.183965	0.545268
C	-7.412426	-0.545268	2.183965
N	-7.931590	-1.040683	1.040683
H	-5.958494	-3.764899	0.707428
H	-5.958494	-0.707428	3.764899
H	-7.874764	-2.586335	-0.359416
H	-7.874764	0.359416	2.586335
C	-2.314085	-2.314085	5.763626
C	-1.147466	-2.843415	6.339680
C	-2.843415	-1.147466	6.339680
C	-0.545268	-2.183965	7.412426
C	-2.183965	-0.545268	7.412426
N	-1.040683	-1.040683	7.931590
H	-0.707428	-3.764899	5.958494
H	-3.764899	-0.707428	5.958494
H	0.359416	-2.586335	7.874764
H	-2.586335	0.359416	7.874764
Pd	0.000000	0.000000	9.472356
N	2.376474	4.046374	4.046374
N	4.046374	2.376474	4.046374
C	2.953741	2.953741	4.564511
C	2.953741	4.564511	2.953741
N	4.046374	4.046374	2.376474
C	4.564511	2.953741	2.953741
C	5.763626	2.314085	2.314085
C	6.339680	2.843415	1.147466
C	6.339680	1.147466	2.843415
C	7.412426	0.545268	2.183965
N	7.931590	1.040683	1.040683
H	5.958494	3.764899	0.707428
H	5.958494	0.707428	3.764899
H	7.874764	2.586335	-0.359416
H	7.874764	-0.359416	2.586335
C	2.314085	5.763626	2.314085
C	2.843415	6.339680	1.147466
C	1.147466	6.339680	2.843415
C	2.183965	7.412426	0.545268
C	0.545268	7.412426	2.183965
N	1.040683	7.931590	1.040683
H	3.764899	5.958494	0.707428
H	0.707428	5.958494	3.764899
H	2.586335	7.874764	-0.359416
H	-0.359416	7.874764	2.586335
C	2.314085	2.314085	5.763626
C	2.843415	1.147466	6.339680
C	1.147466	2.843415	6.339680
C	2.183965	0.545268	7.412426
C	0.545268	2.183965	7.412426
N	1.040683	1.040683	7.931590

H	3.764899	0.707428	5.958494
H	0.707428	3.764899	5.958494
H	2.586335	-0.359416	7.874764
H	-0.359416	2.586335	7.874764
Pd	0.000000	9.472356	-0.000000
Pd	-0.000000	-9.472356	0.000000
Pd	-9.472356	0.000000	-0.000000
N	2.376474	-4.046374	-4.046374
N	4.046374	-2.376474	-4.046374
C	2.953741	-2.953741	-4.564511
C	2.953741	-4.564511	-2.953741
N	4.046374	-4.046374	-2.376474
C	4.564511	-2.953741	-2.953741
C	5.763626	-2.314085	-2.314085
C	6.339680	-1.147466	-2.843415
C	6.339680	-2.843415	-1.147466
C	7.412426	-0.545268	-2.183965
C	7.412426	-2.183965	-0.545268
N	7.931590	-1.040683	-1.040683
H	5.958494	-0.707428	-3.764899
H	5.958494	-3.764899	-0.707428
H	7.874764	0.359416	-2.586335
H	7.874764	-2.586335	0.359416
C	2.314085	-5.763626	-2.314085
C	2.843415	-6.339680	-1.147466
C	1.147466	-6.339680	-2.843415
C	2.183965	-7.412426	-0.545268
C	0.545268	-7.412426	-2.183965
N	1.040683	-7.931590	-1.040683
H	3.764899	-5.958494	-0.707428
H	0.707428	-5.958494	-3.764899
H	2.586335	-7.874764	0.359416
H	-0.359416	-7.874764	-2.586335
C	2.314085	-2.314085	-5.763626
C	2.843415	-1.147466	-6.339680
C	1.147466	-2.843415	-6.339680
C	2.183965	-0.545268	-7.412426
C	0.545268	-2.183965	-7.412426
N	1.040683	-1.040683	-7.931590
H	3.764899	-0.707428	-5.958494
H	0.707428	-3.764899	-5.958494
H	2.586335	0.359416	-7.874764
H	-0.359416	-2.586335	-7.874764
Pd	0.000000	-0.000000	-9.472356
N	-4.046374	2.376474	-4.046374
N	-2.376474	4.046374	-4.046374
C	-2.953741	2.953741	-4.564511
C	-4.564511	2.953741	-2.953741
N	-4.046374	4.046374	-2.376474
C	-2.953741	4.564511	-2.953741
C	-2.314085	5.763626	-2.314085
C	-1.147466	6.339680	-2.843415
C	-2.843415	6.339680	-1.147466

C	7.412426	2.183965	0.545268
C	-2.183965	7.412426	-0.545268
N	-1.040683	7.931590	-1.040683
H	-0.707428	5.958494	-3.764899
H	-3.764899	5.958494	-0.707428
H	-2.586335	7.874764	0.359416
C	-5.763626	2.314085	-2.314085
C	-6.339680	1.147466	-2.843415
C	-6.339680	2.843415	-1.147466
C	-7.412426	0.545268	-2.183965
C	-7.412426	2.183965	-0.545268
N	-7.931590	1.040683	-1.040683
H	-5.958494	0.707428	-3.764899
H	-5.958494	3.764899	-0.707428
H	-7.874764	-0.359416	-2.586335
H	-7.874764	2.586335	0.359416
C	-2.314085	2.314085	-5.763626
C	-1.147466	2.843415	-6.339680
C	-2.843415	1.147466	-6.339680
C	-0.545268	2.183965	-7.412426
C	-2.183965	0.545268	-7.412426
N	-1.040683	1.040683	-7.931590
H	-0.707428	3.764899	-5.958494
H	-3.764899	0.707428	-5.958494
H	0.359416	2.586335	-7.874764
H	-2.586335	-0.359416	-7.874764
Pd	9.472356	-0.000000	0.000000
C	-0.545268	7.412426	-2.183965
H	0.359416	7.874764	-2.586335
C	-2.060484	2.060484	-13.313370
C	-2.469513	2.469513	-12.041207
C	-1.882181	1.882181	-10.923399
N	-0.932361	0.932361	-11.028403
C	-0.519674	0.519674	-12.264632
C	-1.075697	1.075697	-13.420416
H	-2.499965	2.499965	-14.213241
H	-3.236120	3.236120	-11.912073
H	-2.179298	2.179298	-9.918297
H	-0.746335	0.746335	-14.406611
C	2.060484	-2.060484	-13.313370
C	1.075697	-1.075697	-13.420416
C	0.519674	-0.519674	-12.264632
N	0.932361	-0.932361	-11.028403
C	1.882181	-1.882181	-10.923399
C	2.469513	-2.469513	-12.041207
H	2.499965	-2.499965	-14.213241
H	0.746335	-0.746335	-14.406611
H	2.179298	-2.179298	-9.918297
H	3.236120	-3.236120	-11.912073
C	2.060484	-13.313370	-2.060484
C	2.469513	-12.041207	-2.469513
C	1.882181	-10.923399	-1.882181
N	0.932361	-11.028403	-0.932361

C	0.519674	-12.264632	-0.519674
C	1.075697	-13.420416	-1.075697
H	2.499965	-14.213241	-2.499965
H	3.236120	-11.912073	-3.236120
H	2.179298	-9.918297	-2.179298
H	0.746335	-14.406611	-0.746335
C	-2.060484	-13.313370	2.060484
C	-1.075697	-13.420416	1.075697
C	-0.519674	-12.264632	0.519674
N	-0.932361	-11.028403	0.932361
C	-1.882181	-10.923399	1.882181
C	-2.469513	-12.041207	2.469513
H	-2.499965	-14.213241	2.499965
H	-0.746335	-14.406611	0.746335
H	-2.179298	-9.918297	2.179298
H	-3.236120	-11.912073	3.236120
C	2.060484	2.060484	13.313370
C	1.075697	1.075697	13.420416
C	0.519674	0.519674	12.264632
N	0.932361	0.932361	11.028403
C	1.882181	1.882181	10.923399
C	2.469513	2.469513	12.041207
H	2.499965	2.499965	14.213241
H	0.746335	0.746335	14.406611
H	2.179298	2.179298	9.918297
H	3.236120	3.236120	11.912073
C	-2.060484	-2.060484	13.313370
C	-2.469513	-2.469513	12.041207
C	-1.882181	-1.882181	10.923399
N	-0.932361	-0.932361	11.028403
C	-0.519674	-0.519674	12.264632
C	-1.075697	-1.075697	13.420416
H	-2.499965	-2.499965	14.213241
H	-3.236120	-3.236120	11.912073
H	-2.179298	-2.179298	9.918297
H	-0.746335	-0.746335	14.406611
C	13.313370	-2.060484	-2.060484
C	13.420416	-1.075697	-1.075697
C	12.264632	-0.519674	-0.519674
N	11.028403	-0.932361	-0.932361
C	10.923399	-1.882181	-1.882181
C	12.041207	-2.469513	-2.469513
H	14.213241	-2.499965	-2.499965
H	14.406611	-0.746335	-0.746335
H	9.918297	-2.179298	-2.179298
H	11.912073	-3.236120	-3.236120
C	13.313370	2.060484	2.060484
C	12.041207	2.469513	2.469513
C	10.923399	1.882181	1.882181
N	11.028403	0.932361	0.932361
C	12.264632	0.519674	0.519674
C	13.420416	1.075697	1.075697
H	14.213241	2.499965	2.499965

H	11.912073	3.236120	3.236120
H	9.918297	2.179298	2.179298
H	14.406611	0.746335	0.746335
C	-13.313370	-2.060484	2.060484
C	-12.041207	-2.469513	2.469513
C	-10.923399	-1.882181	1.882181
N	-11.028403	-0.932361	0.932361
C	-12.264632	-0.519674	0.519674
C	-13.420416	-1.075697	1.075697
H	-14.213241	-2.499965	2.499965
H	-11.912073	-3.236120	3.236120
H	-9.918297	-2.179298	2.179298
H	-14.406611	-0.746335	0.746335
C	-13.313370	2.060484	-2.060484
C	-13.420416	1.075697	-1.075697
C	-12.264632	0.519674	-0.519674
N	-11.028403	0.932361	-0.932361
C	-10.923399	1.882181	-1.882181
C	-12.041207	2.469513	-2.469513
H	-14.213241	2.499965	-2.499965
H	-14.406611	0.746335	-0.746335
H	-9.918297	2.179298	-2.179298
H	-11.912073	3.236120	-3.236120
C	2.060484	13.313370	2.060484
C	2.469513	12.041207	2.469513
C	1.882181	10.923399	1.882181
N	0.932361	11.028403	0.932361
C	0.519674	12.264632	0.519674
C	1.075697	13.420416	1.075697
H	2.499965	14.213241	2.499965
H	3.236120	11.912073	3.236120
H	2.179298	9.918297	2.179298
H	0.746335	14.406611	0.746335
C	-2.060484	13.313370	-2.060484
C	-1.075697	13.420416	-1.075697
C	-0.519674	12.264632	-0.519674
N	-0.932361	11.028403	-0.932361
C	-1.882181	10.923399	-1.882181
C	-2.469513	12.041207	-2.469513
H	-2.499965	14.213241	-2.499965
H	-0.746335	14.406611	-0.746335
H	-2.179298	9.918297	-2.179298
H	-3.236120	11.912073	-3.236120

318

CAGEBPyTd+NO3c2v SCF Done: -11183.3527194 A.U.

N	-1.180071	4.445179	-3.981537
N	1.180071	4.445179	-3.981537
C	-0.000000	4.079118	-4.494258
C	-1.135630	5.215391	-2.888722
N	-0.000000	5.626587	-2.313665
C	1.135630	5.215391	-2.888722
C	2.423781	5.607631	-2.254880
C	2.442283	6.386114	-1.088106

C	3.647371	5.174907	-2.787162
C	3.660328	6.685499	-0.483165
C	4.833266	5.506475	-2.136745
N	4.821907	6.241479	-1.005815
H	1.513399	6.742213	-0.643800
H	3.675617	4.569153	-3.692306
H	3.746503	7.263445	0.449152
H	5.824520	5.175201	-2.480534
C	-2.423781	5.607631	-2.254880
C	-2.442283	6.386114	-1.088106
C	-3.647371	5.174907	-2.787162
C	-3.660328	6.685499	-0.483165
C	-4.833266	5.506475	-2.136745
N	-4.821907	6.241479	-1.005815
H	-1.513399	6.742213	-0.643800
H	-3.675617	4.569153	-3.692306
H	-3.746503	7.263445	0.449152
H	-5.824520	5.175201	-2.480534
C	-0.000000	3.183556	-5.682895
C	1.204784	2.735708	-6.244717
C	-1.204784	2.735708	-6.244717
C	1.172649	1.849679	-7.318577
C	-1.172649	1.849679	-7.318577
N	-0.000000	1.420305	-7.828520
H	2.161637	3.060997	-5.837717
H	-2.161637	3.060997	-5.837717
H	2.077501	1.434395	-7.786992
H	-2.077501	1.434395	-7.786992
Pd	-0.000000	-0.000000	-9.325937
N	-1.180071	-4.445179	-3.981537
N	1.180071	-4.445179	-3.981537
C	0.000000	-4.079118	-4.494258
C	-1.135630	-5.215391	-2.888722
N	0.000000	-5.626587	-2.313665
C	1.135630	-5.215391	-2.888722
C	2.423781	-5.607631	-2.254880
C	2.442283	-6.386114	-1.088106
C	3.647371	-5.174907	-2.787162
C	4.833266	-5.506475	-2.136745
N	4.821907	-6.241479	-1.005815
H	1.513399	-6.742213	-0.643800
H	3.675617	-4.569153	-3.692306
H	3.746503	-7.263445	0.449152
H	5.824520	-5.175201	-2.480534
C	-2.423781	-5.607631	-2.254880
C	-2.442283	-6.386114	-1.088106
C	-3.647371	-5.174907	-2.787162
C	-3.660328	-6.685499	-0.483165
C	-4.833266	-5.506475	-2.136745
N	-4.821907	-6.241479	-1.005815
H	-1.513399	-6.742213	-0.643800
H	-3.675617	-4.569153	-3.692306
H	-3.746503	-7.263445	0.449152

H	-5.824520	-5.175201	-2.480534
C	0.000000	-3.183556	-5.682895
C	1.204784	-2.735708	-6.244717
C	-1.204784	-2.735708	-6.244717
C	1.172649	-1.849679	-7.318577
C	-1.172649	-1.849679	-7.318577
N	0.000000	-1.420305	-7.828520
H	2.161637	-3.060997	-5.837717
H	-2.161637	-3.060997	-5.837717
H	2.077501	-1.434395	-7.786992
H	-2.077501	-1.434395	-7.786992
Pd	-6.590212	-6.590348	0.000075
Pd	6.590212	6.590348	0.000075
Pd	-6.590212	6.590348	0.000075
N	4.445293	1.180084	3.981639
N	4.445293	-1.180084	3.981639
C	4.079235	0.000000	4.494349
C	5.215462	1.135645	2.888810
N	5.626562	0.000000	2.313704
C	5.215462	-1.135645	2.888810
C	5.607630	-2.423825	2.254982
C	5.175233	-3.647395	2.787555
C	6.385747	-2.442386	1.087959
C	5.506799	-4.833348	2.137226
C	6.685125	-3.660471	0.483115
N	6.241465	-4.822048	1.006063
H	4.569730	-3.675583	3.692883
H	6.741580	-1.513539	0.643394
H	5.175777	-5.824580	2.481327
H	7.262767	-3.746649	-0.449387
C	5.607630	2.423825	2.254982
C	6.385747	2.442386	1.087959
C	5.175233	3.647395	2.787555
C	6.685125	3.660471	0.483115
C	5.506799	4.833348	2.137226
N	6.241465	4.822048	1.006063
H	6.741580	1.513539	0.643394
H	4.569730	3.675583	3.692883
H	7.262767	3.746649	-0.449387
H	5.175777	5.824580	2.481327
C	3.183662	0.000000	5.682982
C	2.735801	-1.204783	6.244793
C	2.735801	1.204783	6.244793
C	1.849724	-1.172649	7.318611
C	1.849724	1.172649	7.318611
N	1.420327	0.000000	7.828537
H	3.061123	-2.161635	5.837815
H	3.061123	2.161635	5.837815
H	1.434384	-2.077511	7.786970
H	1.434384	2.077511	7.786970
Pd	0.000000	0.000000	9.325923
N	-4.445293	1.180084	3.981639
N	-4.445293	-1.180084	3.981639

C	-4.079235	-0.000000	4.494349
C	-5.215462	1.135645	2.888810
N	-5.626562	-0.000000	2.313704
C	-5.215462	-1.135645	2.888810
C	-5.607630	-2.423825	2.254982
C	-5.175233	-3.647395	2.787555
C	-6.385747	-2.442386	1.087959
C	3.660328	-6.685499	-0.483165
C	-6.685125	-3.660471	0.483115
N	-6.241465	-4.822048	1.006063
H	-4.569730	-3.675583	3.692883
H	-6.741580	-1.513539	0.643394
H	-7.262767	-3.746649	-0.449387
C	-5.607630	2.423825	2.254982
C	-5.175233	3.647395	2.787555
C	-6.385747	2.442386	1.087959
C	-5.506799	4.833348	2.137226
C	-6.685125	3.660471	0.483115
N	-6.241465	4.822048	1.006063
H	-4.569730	3.675583	3.692883
H	-6.741580	1.513539	0.643394
H	-5.175777	5.824580	2.481327
H	-7.262767	3.746649	-0.449387
C	-3.183662	-0.000000	5.682982
C	-2.735801	-1.204783	6.244793
C	-2.735801	1.204783	6.244793
C	-1.849724	-1.172649	7.318611
C	-1.849724	1.172649	7.318611
N	-1.420327	-0.000000	7.828537
H	-3.061123	-2.161635	5.837815
H	-3.061123	2.161635	5.837815
H	-1.434384	-2.077511	7.786970
H	-1.434384	2.077511	7.786970
Pd	6.590212	-6.590348	0.000075
C	-5.506799	-4.833348	2.137226
H	-5.175777	-5.824580	2.481327
C	-2.904411	-0.000000	13.153840
C	-3.489781	-0.000000	11.889074
C	-2.657335	-0.000000	10.771143
N	-1.326350	-0.000000	10.876473
C	-0.736936	-0.000000	12.095328
C	-1.514318	-0.000000	13.255602
H	-3.521087	-0.000000	14.055016
H	-4.572736	-0.000000	11.757932
H	-3.068894	-0.000000	9.761946
H	-1.039125	-0.000000	14.235288
C	2.904411	0.000000	13.153840
C	1.514318	0.000000	13.255602
C	0.736936	0.000000	12.095328
N	1.326350	0.000000	10.876473
C	2.657335	0.000000	10.771143
C	3.489781	0.000000	11.889074
H	3.521087	0.000000	14.055016

H	1.039125	0.000000	14.235288
H	3.068894	0.000000	9.761946
H	4.572736	0.000000	11.757932
C	10.748353	7.845930	2.054770
C	10.146438	6.659289	2.469329
C	8.939950	6.284666	1.880458
N	8.349346	7.023945	0.938508
C	8.916670	8.180268	0.521263
C	10.125655	8.612282	1.071069
H	11.693807	8.175039	2.490903
H	10.594799	6.025595	3.235744
H	8.432037	5.365497	2.171962
H	10.581014	9.542316	0.734516
C	7.845649	10.748253	-2.055206
C	8.612067	10.125659	-1.071487
C	8.180101	8.916730	-0.521533
N	7.023751	8.349358	-0.938626
C	6.284409	8.939854	-1.880584
C	6.658994	10.146278	-2.469622
H	8.174726	11.693661	-2.491461
H	9.542111	10.581071	-0.735032
H	5.365215	8.431934	-2.171976
H	6.025251	10.594543	-3.236056
C	-0.000000	-2.904410	-13.153838
C	-0.000000	-1.514317	-13.255599
C	-0.000000	-0.736937	-12.095326
N	-0.000000	-1.326348	-10.876471
C	-0.000000	-2.657332	-10.771141
C	-0.000000	-3.489779	-11.889071
H	-0.000000	-3.521088	-14.055012
H	-0.000000	-1.039122	-14.235283
H	-0.000000	-3.068893	-9.761944
H	0.000000	-4.572734	-11.757930
C	-0.000000	2.904410	-13.153838
C	-0.000000	3.489779	-11.889071
C	-0.000000	2.657332	-10.771141
N	-0.000000	1.326348	-10.876471
C	-0.000000	0.736937	-12.095326
C	-0.000000	1.514317	-13.255599
H	-0.000000	3.521088	-14.055012
H	-0.000000	4.572734	-11.757930
H	-0.000000	3.068893	-9.761944
H	-0.000000	1.039122	-14.235283
C	10.748353	-7.845930	2.054770
C	10.125655	-8.612282	1.071069
C	8.916670	-8.180268	0.521263
N	8.349346	-7.023945	0.938508
C	8.939950	-6.284666	1.880458
C	10.146438	-6.659289	2.469329
H	11.693807	-8.175039	2.490903
H	10.581014	-9.542316	0.734516
H	8.432037	-5.365497	2.171962
H	10.594799	-6.025595	3.235744

C	7.845649	-10.748253	-2.055206
C	6.658994	-10.146278	-2.469622
C	6.284409	-8.939854	-1.880584
N	7.023751	-8.349358	-0.938626
C	8.180101	-8.916730	-0.521533
C	8.612067	-10.125659	-1.071487
H	8.174726	-11.693661	-2.491461
H	6.025251	-10.594543	-3.236056
H	5.365215	-8.431934	-2.171976
H	9.542111	-10.581071	-0.735032
C	-7.845649	10.748253	-2.055206
C	-6.658994	10.146278	-2.469622
C	-6.284409	8.939854	-1.880584
N	-7.023751	8.349358	-0.938626
C	-8.180101	8.916730	-0.521533
C	-8.612067	10.125659	-1.071487
H	-8.174726	11.693661	-2.491461
H	-6.025251	10.594543	-3.236056
H	-5.365215	8.431934	-2.171976
H	-9.542111	10.581071	-0.735032
C	-10.748353	7.845930	2.054770
C	-10.125655	8.612282	1.071069
C	-8.916670	8.180268	0.521263
N	-8.349346	7.023945	0.938508
C	-8.939950	6.284666	1.880458
C	-10.146438	6.659289	2.469329
H	-11.693807	8.175039	2.490903
H	-10.581014	9.542316	0.734516
H	-8.432037	5.365497	2.171962
H	-10.594799	6.025595	3.235744
C	-7.845649	-10.748253	-2.055206
C	-6.658994	-10.146278	-2.469622
C	-6.284409	-8.939854	-1.880584
N	-7.023751	-8.349358	-0.938626
C	-8.180101	-8.916730	-0.521533
C	-8.612067	-10.125659	-1.071487
H	-8.174726	-11.693661	-2.491461
H	-6.025251	-10.594543	-3.236056
H	-5.365215	-8.431934	-2.171976
H	-9.542111	-10.581071	-0.735032
C	-10.748353	-7.845930	2.054770
C	-10.125655	-8.612282	1.071069
C	-8.916670	-8.180268	0.521263
N	-8.349346	-7.023945	0.938508
C	-8.939950	-6.284666	1.880458
C	-10.146438	-6.659289	2.469329
H	-11.693807	-8.175039	2.490903
H	-10.581014	-9.542316	0.734516
H	-8.432037	-5.365497	2.171962
H	-10.594799	-6.025595	3.235744
O	-2.643351	-0.000000	-10.936770
O	2.643351	0.000000	-10.936770
O	-6.408933	9.053196	1.868232

O	-9.051247	6.409061	-1.869250
O	0.000000	2.643710	10.936399
O	0.000000	-2.643710	10.936399
O	-9.051247	-6.409061	-1.869250
O	-6.408933	-9.053196	1.868232
O	9.051247	6.409061	-1.869250
O	6.408933	9.053196	1.868232
O	6.408933	-9.053196	1.868232
O	9.051247	-6.409061	-1.869250
N	5.327251	-8.762577	2.424713
O	4.731246	-7.677697	2.076301
O	4.827411	-9.477265	3.283169
N	8.762497	-5.326240	-2.424533
O	7.677619	-4.729803	-2.076950
O	9.479012	-4.825783	-3.281110
N	0.000000	-3.434108	9.967162
O	0.000000	-2.944810	8.778090
O	0.000000	-4.648202	10.120782
N	-0.000000	3.434108	9.967162
O	-0.000000	2.944810	8.778090
O	-0.000000	4.648202	10.120782
N	8.762497	5.326240	-2.424533
O	9.479012	4.825783	-3.281110
O	7.677619	4.729803	-2.076950
N	5.327251	8.762577	2.424713
O	4.731246	7.677697	2.076301
O	4.827411	9.477265	3.283169
N	-5.327251	8.762577	2.424713
O	-4.827411	9.477265	3.283169
O	-4.731246	7.677697	2.076301
N	-8.762497	5.326240	-2.424533
O	-7.677619	4.729803	-2.076950
O	-9.479012	4.825783	-3.281110
N	-3.433783	-0.000000	-9.967558
O	-4.647879	-0.000000	-10.121203
O	-2.944498	-0.000000	-8.778476
N	3.433783	0.000000	-9.967558
O	4.647879	0.000000	-10.121203
O	2.944498	0.000000	-8.778476
N	-5.327251	-8.762577	2.424713
O	-4.827411	-9.477265	3.283169
O	-4.731246	-7.677697	2.076301
N	-8.762497	-5.326240	-2.424533
O	-7.677619	-4.729803	-2.076950
O	-9.479012	-4.825783	-3.281110

318

CAGEBPyTd+NO3 SCF Done: -11183.3527501 A.U.

N	-1.837988	0.066947	5.798621
N	0.158383	1.318206	5.936601
C	-1.171690	1.226549	5.827290
C	-1.095327	-1.042847	5.877781
N	0.237723	-1.039985	5.989150
C	0.825854	0.161294	6.010563

C	2.311355	0.212890	6.084874
C	3.069720	-0.966431	6.127611
C	2.988798	1.441179	6.074160
C	4.460027	-0.887373	6.134484
C	4.381265	1.455980	6.082490
N	5.086924	0.306628	6.105474
H	2.581265	-1.940258	6.137926
H	2.436075	2.379529	6.042053
H	5.115749	-1.770880	6.135954
H	4.976207	2.380719	6.043980
C	-1.789149	-2.357253	5.801324
C	-1.062444	-3.556397	5.842258
C	-3.181675	-2.426492	5.646990
C	-1.733113	-4.769243	5.706399
C	-3.795799	-3.669503	5.516438
N	-3.071286	-4.807032	5.540987
H	0.020453	-3.545236	5.961622
H	-3.781937	-1.517955	5.611267
H	-1.223375	-5.744263	5.697960
H	-4.877750	-3.795964	5.361598
C	-1.952216	2.486662	5.693864
C	-1.307816	3.732574	5.680011
C	-3.346099	2.455219	5.539442
C	-2.058398	4.890360	5.491813
C	-4.042266	3.647096	5.354961
N	-3.395910	4.830701	5.327551
H	-0.226732	3.799556	5.797507
H	-3.883865	1.507692	5.545319
H	-1.615393	5.896111	5.439008
H	-5.130082	3.693609	5.196662
Pd	-4.448772	6.556385	4.911012
N	-3.956052	4.280501	-1.744299
N	-1.959458	5.531347	-1.606158
C	-3.115203	5.093116	-1.094649
C	-3.580143	3.899665	-2.970306
N	-2.442620	4.291234	-3.555244
C	-1.658720	5.103409	-2.837372
C	-0.360007	5.526403	-3.428423
C	0.029253	5.081018	-4.700368
C	0.521913	6.348103	-2.710956
C	1.756565	6.676701	-3.264959
N	2.113858	6.220056	-4.482861
H	-0.627571	4.441970	-5.289635
H	0.256354	6.715433	-1.720210
H	1.658775	5.104749	-6.176033
H	2.508197	7.289958	-2.745790
C	-4.461174	2.957041	-3.712028
C	-4.104094	2.491835	-4.986316
C	-5.649294	2.481652	-3.137490
C	-4.917854	1.562734	-5.629632
C	-6.421785	1.552828	-3.830141
N	-6.046165	1.107960	-5.046896
H	-3.189528	2.837439	-5.467113

H	-5.961988	2.819189	-2.149908
H	-4.682657	1.132702	-6.614676
H	-7.347102	1.115181	-3.426605
C	-3.468853	5.504377	0.291246
C	-2.610686	6.325098	1.038038
C	-4.649449	5.048639	0.896414
C	-2.938958	6.642737	2.353569
C	-4.923298	5.400347	2.215708
N	-4.072095	6.176469	2.917549
H	-1.684451	6.700145	0.603910
H	-5.342461	4.409910	0.349799
H	-2.297783	7.254337	3.005809
H	-5.813408	5.053375	2.761500
Pd	-7.146472	-0.371828	-5.974112
Pd	7.145609	0.372838	5.975158
Pd	-4.005142	-6.616413	5.203427
N	5.943265	-1.000438	-0.835591
N	5.381081	0.117295	-2.836788
C	5.597142	-0.996257	-2.127734
C	6.062048	0.197515	-0.252276
N	5.864214	1.357775	-0.887962
C	5.520993	1.273151	-2.178107
C	5.243575	2.536814	-2.913705
C	4.844460	2.512358	-4.258265
C	5.336701	3.778823	-2.268352
C	4.532280	3.706900	-4.902364
C	5.011389	4.939568	-2.965569
N	4.612896	4.886521	-4.253149
H	4.760581	1.568039	-4.795165
H	5.643723	3.840360	-1.224808
H	4.183370	3.758494	-5.944563
H	5.031965	5.942329	-2.513195
C	6.398363	0.241019	1.196836
C	6.500918	1.465287	1.873882
C	6.581596	-0.942328	1.927491
C	6.755756	1.472133	3.242920
C	6.834255	-0.871221	3.295108
N	6.909928	0.318921	3.925431
H	6.365927	2.406563	1.342084
H	6.510734	-1.913232	1.438250
H	6.817716	2.393112	3.841730
H	6.957008	-1.758425	3.934152
C	5.406036	-2.307012	-2.806199
C	5.007943	-2.368942	-4.149864
C	5.581393	-3.510183	-2.106651
C	4.777332	-3.609076	-4.739902
C	5.335465	-4.719851	-2.751228
N	4.935921	-4.750719	-4.039203
H	4.861641	-1.457079	-4.727909
H	5.890560	-3.504724	-1.061940
H	4.433888	-3.730067	-5.778162
H	5.422774	-5.697963	-2.255004
Pd	4.448159	-6.556683	-4.911186

N	-1.582952	-5.716060	-1.356838
N	-2.144950	-4.597805	-3.357792
C	-1.309584	-5.323400	-2.606047
C	-2.766180	-5.334068	-0.863408
N	-3.658510	-4.608810	-1.546918
C	-3.307014	-4.257892	-2.788999
C	-4.248000	-3.409970	-3.570333
C	-3.921126	-2.979302	-4.864705
C	-5.465778	-2.989480	-3.015416
C	1.277086	5.443375	-5.201236
C	-6.297523	-2.145901	-3.747416
N	-5.950922	-1.731833	-4.983499
H	-2.985015	-3.284408	-5.331229
H	-5.756501	-3.302675	-2.013209
H	-7.250394	-1.753184	-3.362206
C	-3.093370	-5.706674	0.539811
C	-2.183354	-6.434998	1.320289
C	-4.302246	-5.304168	1.126630
C	-2.491046	-6.715451	2.649167
C	-4.553369	-5.614772	2.460698
N	-3.653103	-6.300893	3.194355
H	-1.233840	-6.766485	0.901274
H	-5.035605	-4.737515	0.553810
H	-1.811385	-7.253961	3.326411
H	-5.465027	-5.303864	2.992514
C	0.015428	-5.684204	-3.179701
C	0.375955	-5.270560	-4.470691
C	0.949280	-6.412412	-2.427796
C	1.645983	-5.570533	-4.956830
C	2.204008	-6.681846	-2.968432
N	2.531765	-6.256881	-4.205963
H	-0.321253	-4.703641	-5.086759
H	0.707421	-6.752362	-1.421333
H	2.005588	-5.250837	-5.946301
H	2.994215	-7.219578	-2.423462
Pd	4.007043	6.615525	-5.203565
C	-4.794137	-2.136055	-5.547385
H	-4.586835	-1.735730	-6.551041
C	3.817875	-10.787918	-7.099332
C	2.719091	-10.208789	-6.467236
C	2.889798	-8.981447	-5.829089
N	4.065994	-8.350078	-5.806244
C	5.146310	-8.894747	-6.413974
C	5.042375	-10.122679	-7.071189
H	3.726294	-11.748479	-7.610550
H	1.740374	-10.690530	-6.461830
H	2.059989	-8.489943	-5.321434
H	5.911870	-10.559773	-7.559557
C	8.731967	-7.709229	-6.757472
C	7.604509	-8.517443	-6.893016
C	6.393162	-8.113565	-6.327276
N	6.310164	-6.944077	-5.650171
C	7.385904	-6.164712	-5.516193

C	8.623640	-6.509677	-6.056334
H	9.683701	-8.016134	-7.196128
H	7.670057	-9.458142	-7.437411
H	7.252553	-5.236944	-4.960003
H	9.477215	-5.843560	-5.923341
C	11.942506	0.538061	6.201507
C	11.348825	0.489490	4.941575
C	9.958028	0.441434	4.863340
N	9.184813	0.440587	5.951783
C	9.740717	0.487272	7.185418
C	11.128625	0.536850	7.333027
H	13.028757	0.576583	6.306308
H	11.943089	0.488347	4.026787
H	9.448752	0.402461	3.900540
H	11.574587	0.574317	8.325647
C	8.215745	0.515800	10.657305
C	9.185529	0.525392	9.656227
C	8.795118	0.481510	8.316000
N	7.482884	0.429904	7.986680
C	6.548297	0.420419	8.940125
C	6.870954	0.462329	10.295438
H	8.510755	0.549829	11.708123
H	10.241153	0.566884	9.919880
H	5.510842	0.377998	8.608819
H	6.075672	0.452776	11.042047
C	-6.967554	10.623803	4.463941
C	-6.685523	10.037015	5.696371
C	-5.946586	8.852874	5.744553
N	-5.504772	8.274834	4.602742
C	-5.771027	8.831113	3.418583
C	-6.502833	10.011567	3.301422
H	-7.544545	11.549629	4.415629
H	-7.040343	10.500984	6.615289
H	-5.387030	8.316330	2.538000
H	-6.697760	10.432200	2.313970
C	-5.586289	7.872611	9.390057
C	-4.843025	6.705977	9.220395
C	-4.506989	6.314111	7.925692
N	-4.873702	7.018555	6.852440
C	-5.596038	8.154845	6.994471
C	-5.965392	8.602537	8.264790
H	-5.870136	8.214259	10.387612
H	-4.522917	6.100818	10.069692
H	-3.927128	5.409472	7.743266
H	-6.546351	9.516479	8.377811
C	8.206882	7.968404	-7.106069
C	7.028280	8.693035	-7.275022
C	5.845837	8.234658	-6.689991
N	5.840086	7.093153	-5.961972
C	6.965156	6.393737	-5.795832
C	8.177816	6.796247	-6.352773
H	9.136597	8.318421	-7.559418
H	7.031709	9.611200	-7.860285

H	6.893201	5.484492	-5.199204
H	9.073677	6.194886	-6.192181
C	3.099096	10.695046	-7.574468
C	2.040576	10.072457	-6.915542
C	2.291851	8.888436	-6.224389
N	3.507423	8.338317	-6.175986
C	4.549817	8.926475	-6.808883
C	4.365134	10.114671	-7.519261
H	2.944372	11.623974	-8.127242
H	1.031966	10.487790	-6.929543
H	1.496005	8.365613	-5.694130
H	5.204177	10.586776	-8.027887
C	-5.055436	-7.805916	9.738850
C	-4.392362	-6.600544	9.515960
C	-4.082390	-6.244866	8.204419
N	-4.399889	-7.019424	7.164291
C	-5.044088	-8.194365	7.358186
C	-5.383498	-8.609024	8.647923
H	-5.316444	-8.121247	10.751198
H	-4.114513	-5.938040	10.336862
H	-3.564656	-5.312230	7.980744
H	-5.901583	-9.554128	8.802662
C	-6.243739	-10.860164	4.942670
C	-6.003036	-10.201508	6.147235
C	-5.345627	-8.969305	6.141242
N	-4.942716	-8.414128	4.973977
C	-5.169854	-9.039134	3.816252
C	-5.820323	-10.270253	3.753143
H	-6.756973	-11.823991	4.936719
H	-6.326620	-10.647021	7.086591
H	-4.820719	-8.539225	2.912892
H	-5.985593	-10.746506	2.785745
C	-10.179958	2.376935	-8.490210
C	-9.229247	3.012520	-7.693693
C	-8.343809	2.225536	-6.959259
N	-8.380144	0.891424	-6.996692
C	-9.295451	0.253600	-7.763807
C	-10.211540	0.983882	-8.524086
H	-10.891932	2.956978	-9.081015
H	-9.164798	4.099850	-7.634106
H	-7.583336	2.677265	-6.322470
H	-10.947247	0.469865	-9.140609
C	-9.985682	-3.427261	-8.359808
C	-10.110196	-2.042361	-8.456151
C	-9.246155	-1.219115	-7.730721
N	-8.291442	-1.759278	-6.937108
C	-8.166155	-3.084960	-6.839829
C	-8.995912	-3.961512	-7.536892
H	-10.656383	-4.079510	-8.922957
H	-10.877626	-1.606832	-9.094071
H	-7.378249	-3.455837	-6.184445
H	-8.859102	-5.038334	-7.428595
O	-7.454504	6.289567	5.604443

O	-2.983853	9.092220	5.914194
O	-2.373100	-9.000803	6.315043
O	-7.022441	-6.521310	5.889487
O	5.847074	-8.942345	-3.519849
O	4.588518	-6.438144	-8.001451
O	-10.076777	-0.447719	-4.977948
O	-6.689913	-0.425082	-9.035641
O	8.292932	3.078391	6.947762
O	8.470590	-2.203061	7.068177
O	4.159426	6.370488	-8.285834
O	5.242117	9.148781	-3.921093
N	3.582373	5.266479	-8.397068
O	3.169985	4.680653	-7.329297
O	3.401063	4.736592	-9.485154
N	4.986543	8.875482	-2.727705
O	4.373621	7.775082	-2.469135
O	5.301460	9.621455	-1.810135
N	3.937379	-5.382331	-8.161162
O	3.486805	-4.777198	-7.119940
O	3.721467	-4.915697	-9.271667
N	5.571645	-8.634632	-2.339331
O	4.887553	-7.566460	-2.128619
O	5.932994	-9.317518	-1.390294
N	7.523255	3.829145	6.308874
O	7.601270	5.048410	6.378780
O	6.626916	3.292498	5.559232
N	7.754437	-3.031211	6.463615
O	6.826868	-2.589524	5.690368
O	7.913451	-4.238225	6.587781
N	-1.261920	-8.682593	5.837339
O	-0.259879	-9.361154	6.019132
O	-1.182153	-7.608358	5.135172
N	-7.301701	-5.462949	5.284425
O	-6.360679	-4.847475	4.660685
O	-8.435949	-5.003713	5.271478
N	-7.659735	5.188381	5.048151
O	-8.759986	4.652706	5.057195
O	-6.677807	4.611288	4.451357
N	-1.852955	8.829725	5.449072
O	-0.899530	9.581957	5.599775
O	-1.699101	7.733175	4.795432
N	-5.440066	-0.382678	-9.020681
O	-4.779053	-0.383823	-10.050558
O	-4.842479	-0.337754	-7.883037
N	-9.839086	-0.412739	-3.750560
O	-8.613333	-0.362753	-3.365303
O	-10.734387	-0.424531	-2.916372

162

CAGEHc3v SCF Done: -4858.76210455 A.U.

N	-1.180708	5.880025	-1.356964
N	0.000000	5.200341	-3.285746
C	-1.136070	5.434648	-2.618230
C	0.000000	6.088949	-0.762487

N	1.180708	5.880025	-1.356964
C	1.136070	5.434648	-2.618230
C	2.422035	5.162353	-3.309538
C	3.649602	5.366248	-2.661973
C	2.448029	4.676168	-4.625138
C	4.829287	5.062870	-3.337250
C	3.676613	4.400860	-5.220505
N	4.849706	4.575586	-4.589193
H	3.676069	5.752430	-1.643330
H	1.518031	4.513074	-5.169170
H	5.802613	5.211049	-2.862702
H	3.732915	4.022315	-6.244242
C	0.000000	6.557914	0.646714
C	1.201447	6.778185	1.336524
C	-1.201447	6.778185	1.336524
C	1.152547	7.183375	2.668101
C	-1.152547	7.183375	2.668101
N	0.000000	7.371466	3.332562
H	2.157814	6.629162	0.835899
H	-2.157814	6.629162	0.835899
H	2.069512	7.365123	3.234357
H	-2.069512	7.365123	3.234357
C	-2.422035	5.162353	-3.309538
C	-2.448029	4.676168	-4.625138
C	-3.649602	5.366248	-2.661973
C	-3.676613	4.400860	-5.220505
C	-4.829287	5.062870	-3.337250
N	-4.849706	4.575586	-4.589193
H	-1.518031	4.513074	-5.169170
H	-3.676069	5.752430	-1.643330
H	-3.732915	4.022315	-6.244242
H	-5.802613	5.211049	-2.862702
Pd	-6.752196	3.898382	-5.515797
N	-5.682605	-1.917490	-1.356964
N	-4.503628	-2.600171	-3.285746
C	-5.274578	-1.733458	-2.618230
C	-5.273184	-3.044474	-0.762487
N	-4.501897	-3.962536	-1.356964
C	-4.138508	-3.701189	-2.618230
C	-3.259712	-4.678721	-3.309538
C	-2.822506	-5.843772	-2.661973
C	-2.825666	-4.458139	-4.625138
C	-1.972949	-5.384470	-5.220505
N	-1.537720	-6.487762	-4.589193
H	-3.143716	-6.059784	-1.643330
H	-3.149421	-3.571190	-5.169170
H	-1.611595	-7.630735	-2.862702
H	-1.616970	-5.243957	-6.244242
C	-5.679321	-3.278957	0.646714
C	-5.269356	-4.429576	1.336524
C	-6.470804	-2.348608	1.336524
C	-5.644712	-4.589822	2.668101
C	-6.797259	-2.593553	2.668101

N	-6.383877	-3.685733	3.332562
H	-4.662115	-5.183303	0.835899
H	-6.819930	-1.445859	0.835899
H	-5.343628	-5.474812	3.234357
H	-7.413140	-1.890312	3.234357
C	-5.681747	-0.483633	-3.309538
C	-5.273694	-0.218029	-4.625138
C	-6.472108	0.477524	-2.661973
C	-5.649563	0.983611	-5.220505
C	-6.799217	1.650850	-3.337250
N	-6.387427	1.912176	-4.589193
H	-4.667452	-0.941884	-5.169170
H	-6.819785	0.307355	-1.643330
H	-5.349885	1.221641	-6.244242
H	-7.414207	2.419685	-2.862702
Pd	-6.751913	-3.898219	5.513204
Pd	6.752196	3.898382	-5.515797
Pd	0.000000	7.796437	5.513204
H	-8.089376	3.575633	-6.221558
H	-7.141277	5.217789	-6.221558
N	5.682605	-1.917490	-1.356964
N	4.501897	-3.962536	-1.356964
C	5.273184	-3.044474	-0.762487
C	5.274578	-1.733458	-2.618230
N	4.503628	-2.600171	-3.285746
C	4.138508	-3.701189	-2.618230
C	3.259712	-4.678721	-3.309538
C	2.822506	-5.843772	-2.661973
C	2.825666	-4.458139	-4.625138
C	1.969930	-6.713720	-3.337250
C	1.972949	-5.384470	-5.220505
N	1.537720	-6.487762	-4.589193
H	3.143716	-6.059784	-1.643330
H	3.149421	-3.571190	-5.169170
H	1.611595	-7.630735	-2.862702
H	1.616970	-5.243957	-6.244242
C	5.681747	-0.483633	-3.309538
C	5.273694	-0.218029	-4.625138
C	6.472108	0.477524	-2.661973
C	5.649563	0.983611	-5.220505
C	6.799217	1.650850	-3.337250
N	6.387427	1.912176	-4.589193
H	4.667452	-0.941884	-5.169170
H	6.819785	0.307355	-1.643330
H	5.349885	1.221641	-6.244242
H	7.414207	2.419685	-2.862702
C	5.679321	-3.278957	0.646714
C	5.269356	-4.429576	1.336524
C	6.470804	-2.348608	1.336524
C	5.644712	-4.589822	2.668101
C	6.797259	-2.593553	2.668101
N	6.383877	-3.685733	3.332562
H	4.662115	-5.183303	0.835899

H	6.819930	-1.445859	0.835899
H	5.343628	-5.474812	3.234357
H	7.413140	-1.890312	3.234357
Pd	6.751913	-3.898219	5.513204
N	1.180703	0.681679	6.003053
N	-0.000000	-1.363359	6.003053
C	1.136065	-0.655907	6.002321
C	0.000000	1.311815	6.002321
N	-1.180703	0.681679	6.003053
C	-1.136065	-0.655907	6.002321
C	-2.422054	-1.398374	5.975360
C	-2.448140	-2.800763	5.953230
C	-3.649602	-0.719770	5.953230
C	-1.969930	-6.713720	-3.337250
C	-4.829343	-1.457357	5.891353
N	-4.849844	-2.800059	5.846918
H	-1.518176	-3.368160	5.979621
H	-3.676000	0.369301	5.979621
H	-5.802663	-0.960524	5.873969
C	0.000000	2.796747	5.975360
C	1.201462	3.520533	5.953230
C	-1.201462	3.520533	5.953230
C	1.152563	4.911012	5.891353
C	-1.152563	4.911012	5.891353
N	0.000000	5.600117	5.846918
H	2.157824	2.998859	5.979621
H	-2.157824	2.998859	5.979621
H	2.069493	5.505516	5.873969
H	-2.069493	5.505516	5.873969
C	2.422054	-1.398374	5.975360
C	2.448140	-2.800763	5.953230
C	3.649602	-0.719770	5.953230
C	3.676780	-3.453655	5.891353
C	4.829343	-1.457357	5.891353
N	4.849844	-2.800059	5.846918
H	1.518176	-3.368160	5.979621
H	3.676000	0.369301	5.979621
H	3.733170	-4.544991	5.873969
H	5.802663	-0.960524	5.873969
Pd	-0.000000	-7.796765	-5.515797
H	7.144155	-4.124680	6.991435
H	8.089397	-4.670415	5.440511
C	-3.676780	-3.453655	5.891353
H	-3.733170	-4.544991	5.873969
H	-7.144155	-4.124680	6.991435
H	0.948099	-8.793421	-6.221558
H	8.089376	3.575633	-6.221558
H	0.000000	9.340831	5.440511
H	-8.089397	-4.670415	5.440511
H	0.000000	8.249359	6.991435
H	-0.948099	-8.793421	-6.221558
H	7.141277	5.217789	-6.221558

162

CAGEH SCF Done: -4858.76210274 A.U.

N	0.957395	1.410237	-5.909118
N	1.414110	3.479614	-4.867325
C	1.801493	2.311065	-5.392280
C	-0.341244	1.732338	-5.878363
N	-0.812828	2.878186	-5.372624
C	0.098266	3.723702	-4.876462
C	-0.380783	4.997005	-4.280701
C	-1.748078	5.307904	-4.239538
C	0.518082	5.920083	-3.725885
C	-2.153607	6.497424	-3.639319
C	0.020332	7.084688	-3.146578
N	-1.291659	7.368533	-3.088435
H	-2.480267	4.624071	-4.668185
H	1.589728	5.723497	-3.745626
H	-3.210673	6.770959	-3.591292
H	0.692764	7.825394	-2.706464
C	-1.317930	0.750985	-6.415724
C	-2.695493	1.015107	-6.395233
C	-0.894045	-0.477296	-6.944379
C	-3.576575	0.050057	-6.876924
C	-1.848436	-1.381532	-7.403908
N	-3.168521	-1.134921	-7.361302
H	-3.067850	1.961141	-6.003025
H	0.167527	-0.719224	-6.989239
H	-4.655600	0.223857	-6.876576
H	-1.552622	-2.346605	-7.822984
C	3.250354	1.984945	-5.379748
C	4.188032	2.874252	-4.834075
C	3.723597	0.770163	-5.897613
C	5.532170	2.510336	-4.811539
C	5.086665	0.491967	-5.831975
N	5.979093	1.336105	-5.287649
H	3.865309	3.833534	-4.430229
H	3.031192	0.054621	-6.340387
H	6.288893	3.179849	-4.394847
H	5.488955	-0.444204	-6.227259
Pd	8.097772	0.727790	-5.009328
N	5.401270	-2.867938	0.652897
N	5.859829	-0.798018	1.692793
C	5.886704	-1.620840	0.637628
C	4.864334	-3.278393	1.808043
N	4.797100	-2.520624	2.909150
C	5.305219	-1.286585	2.808508
C	5.228121	-0.399550	3.997205
C	4.643255	-0.840970	5.193415
C	5.722139	0.912695	3.954562
C	5.595494	1.720201	5.082192
N	5.014035	1.301608	6.218816
H	4.255980	-1.856364	5.273773
H	6.193598	1.293203	3.048801
H	4.112260	-0.273901	7.217388
H	5.970473	2.746793	5.083416

C	4.288902	-4.646408	1.864479
C	3.691599	-5.132861	3.036840
C	4.309861	-5.487609	0.742071
C	3.133275	-6.408763	3.032434
C	3.726521	-6.749139	0.831114
N	3.134883	-7.203064	1.948779
H	3.662528	-4.516757	3.935193
H	4.773066	-5.153941	-0.186134
H	2.661282	-6.818894	3.928826
H	3.726625	-7.430099	-0.023754
C	6.468089	-1.112281	-0.630866
C	6.980793	0.190329	-0.721400
C	6.506607	-1.913100	-1.781984
C	7.483477	0.636254	-1.941286
C	7.028265	-1.381458	-2.958794
N	7.497459	-0.125605	-3.047928
H	6.980293	0.842938	0.151280
H	6.128581	-2.934741	-1.753557
H	7.891793	1.644603	-2.046228
H	7.074199	-1.978214	-3.873279
Pd	2.024387	-9.127490	1.946973
Pd	-2.025381	9.127734	-1.946845
Pd	-4.639029	-2.712914	-7.893733
H	9.601270	0.370656	-4.960168
H	8.664983	1.271760	-6.340814
N	-3.118830	4.201939	3.230190
N	-1.953488	3.080444	4.950826
C	-3.042576	3.282467	4.199664
C	-2.021084	4.940714	3.029490
N	-0.891788	4.803706	3.734493
C	-0.899717	3.861461	4.684893
C	0.340827	3.654988	5.474930
C	0.408274	2.672076	6.473520
C	1.488551	4.425022	5.234862
C	1.603987	2.490232	7.164101
C	2.640291	4.171820	5.975823
N	2.708354	3.214052	6.915668
H	-0.463085	2.058180	6.700090
H	1.477059	5.206435	4.475442
H	1.690624	1.734383	7.948764
H	3.551312	4.753822	5.815139
C	-2.049939	5.955882	1.945816
C	-0.927730	6.750419	1.667953
C	-3.194508	6.139305	1.155772
C	-0.988900	7.664373	0.618847
C	-3.163439	7.078130	0.127525
N	-2.079096	7.821173	-0.150669
H	-0.020986	6.648224	2.263638
H	-4.092098	5.550705	1.343786
H	-0.133365	8.299746	0.376228
H	-4.037850	7.247013	-0.506035
C	-4.227827	2.420553	4.440501
C	-4.210238	1.424681	5.428321

C	-5.395092	2.564497	3.675900
C	-5.332341	0.616615	5.594365
C	-6.468936	1.709986	3.913195
N	-6.440278	0.742131	4.844680
H	-3.327806	1.285119	6.052265
H	-5.455801	3.332200	2.904919
H	-5.352664	-0.167596	6.355355
H	-7.393483	1.795564	3.336652
Pd	-8.098099	-0.727836	5.009637
N	-4.863086	-3.693745	-0.734686
N	-3.697586	-4.815107	0.985911
C	-4.645760	-3.974228	0.555758
C	-4.063736	-4.307832	-1.615076
N	-3.092639	-5.161746	-1.270227
C	-2.942361	-5.386836	0.040454
C	-1.859496	-6.307025	0.472169
C	-1.634693	-6.573650	1.830932
C	-1.017290	-6.924080	-0.464736
C	4.560614	0.037882	6.270667
C	0.007608	-7.751094	-0.011660
N	0.234393	-7.986153	1.291630
H	-2.271746	-6.123681	2.591957
H	-1.162854	-6.752983	-1.531041
H	0.680976	-8.248268	-0.714442
C	-4.249910	-4.006231	-3.057336
C	-5.235634	-3.105562	-3.487423
C	-3.434247	-4.599638	-4.032232
C	-5.349819	-2.826107	-4.847039
C	-3.621691	-4.259331	-5.369654
N	-4.551705	-3.378818	-5.775909
H	-5.898008	-2.629577	-2.764809
H	-2.662775	-5.312948	-3.743301
H	-6.107578	-2.130389	-5.216212
H	-3.004530	-4.703758	-6.154579
C	-5.490725	-3.295002	1.570852
C	-5.302851	-3.526647	2.941586
C	-6.490951	-2.387890	1.190670
C	-6.094761	-2.839821	3.858547
C	-7.234550	-1.747463	2.178884
N	-7.036304	-1.954325	3.491579
H	-4.544024	-4.231302	3.280861
H	-6.677842	-2.186091	0.136245
H	-5.975377	-2.997391	4.933459
H	-8.022068	-1.036125	1.917513
Pd	4.640301	2.714025	7.892411
H	-9.317535	-1.651549	5.233546
H	-8.950137	0.010712	6.067482
C	-0.584726	-7.415023	2.190564
H	-0.382746	-7.644953	3.239779
H	1.389166	-10.536314	1.993244
H	4.523294	3.651191	9.116518
H	-2.526894	10.434578	-1.290304
H	-4.806111	-2.368930	-9.391805

H	3.177112	-10.053755	2.398285
H	-5.658683	-3.751988	-8.414441
H	5.944475	2.472143	8.686794
H	-2.041315	10.155617	-3.101654

43

CAGEmonocomplex+2 SCF Done: -1118.81668078 A.U.

C	-3.589612	3.302659	-0.000470
C	-3.065957	2.821675	-1.204326
C	-3.066054	2.822228	1.203638
C	-2.041106	1.879883	-1.166630
C	-2.041185	1.880422	1.166450
N	-1.538403	1.422091	0.000043
H	-3.443077	3.168525	-2.168238
H	-3.443232	3.169513	2.167371
H	-1.605009	1.482788	-2.086201
H	-1.605156	1.483735	2.086234
Pd	-0.047413	-0.000016	0.000159
C	-3.589626	-3.302644	-0.000064
C	-3.065569	-2.822329	-1.204006
C	-3.066445	-2.821575	1.203958
C	-2.040697	-1.880545	-1.166485
C	-2.041548	-1.879815	1.166599
N	-1.538370	-1.422130	0.000096
H	-3.442382	-3.169686	-2.167856
H	-3.443951	-3.168340	2.167751
H	-1.604278	-1.483966	-2.086129
H	-1.605810	-1.482635	2.086305
C	3.792781	-2.914202	-0.000197
C	2.524769	-3.496791	0.000015
C	1.406459	-2.665238	0.000229
N	1.513711	-1.326014	0.000200
C	2.743536	-0.738401	0.000030
C	3.900805	-1.522059	-0.000170
H	4.691938	-3.534454	-0.000379
H	2.393093	-4.580143	0.000018
H	0.398516	-3.080235	0.000481
H	4.884815	-1.054087	-0.000295
C	3.792723	2.914257	-0.000111
C	3.900774	1.522114	-0.000083
C	2.743521	0.738435	0.000038
N	1.513684	1.326024	0.000147
C	1.406406	2.665242	0.000130
C	2.524701	3.496820	-0.000016
H	4.691869	3.534526	-0.000213
H	4.884793	1.054161	-0.000135
H	0.398455	3.080221	0.000265
H	2.392999	4.580169	-0.000045
H	-4.394471	4.041780	-0.000673
H	-4.394509	-4.041737	-0.000123

43

CAGEmonocomplexc2v+2 SCF Done: -1118.81668073 A.U.

C	0.000000	3.301421	3.590592
C	-1.203966	2.821048	3.066626

C	1.203966	2.821048	3.066626
C	-1.166519	1.879915	2.041175
C	1.166519	1.879915	2.041175
N	0.000000	1.422051	1.538201
H	-2.167816	3.167885	3.443936
H	2.167816	3.167885	3.443936
H	-2.086203	1.483381	1.604786
H	2.086203	1.483381	1.604786
Pd	0.000000	0.000000	0.047131
C	-0.000000	-3.301421	3.590592
C	-1.203966	-2.821048	3.066626
C	1.203966	-2.821048	3.066626
C	-1.166519	-1.879915	2.041175
C	1.166519	-1.879915	2.041175
N	-0.000000	-1.422051	1.538201
H	-2.167816	-3.167885	3.443936
H	2.167816	-3.167885	3.443936
H	-2.086203	-1.483381	1.604786
H	2.086203	-1.483381	1.604786
C	-0.000000	-2.914245	-3.792959
C	-0.000000	-3.496794	-2.524875
C	-0.000000	-2.665265	-1.406639
N	-0.000000	-1.325963	-1.513922
C	-0.000000	-0.738426	-2.743767
C	-0.000000	-1.522141	-3.901031
H	-0.000000	-3.534604	-4.692042
H	-0.000000	-4.580151	-2.393202
H	-0.000000	-3.080071	-0.398608
H	-0.000000	-1.054161	-4.885036
C	0.000000	2.914245	-3.792959
C	0.000000	1.522141	-3.901031
C	0.000000	0.738426	-2.743767
N	0.000000	1.325963	-1.513922
C	0.000000	2.665265	-1.406639
C	0.000000	3.496794	-2.524875
H	0.000000	3.534604	-4.692042
H	0.000000	1.054161	-4.885036
H	0.000000	3.080071	-0.398608
H	0.000000	4.580151	-2.393202
H	0.000000	4.040019	4.395929
H	-0.000000	-4.040019	4.395929

51

CAGEmonocomplexc2v+NO3 SCF Done: -1679.48401589 A.U.

C	-0.000000	3.415681	3.599978
C	1.203661	2.906659	3.105922
C	-1.203661	2.906659	3.105922
C	1.173667	1.909035	2.131967
C	-1.173667	1.909035	2.131967
N	-0.000000	1.437508	1.663177
H	2.167809	3.270359	3.465817
H	-2.167809	3.270359	3.465817
H	2.077385	1.452548	1.702003
H	-2.077385	1.452548	1.702003

Pd	0.000000	0.000000	0.193538
C	-0.000000	-3.415681	3.599978
C	1.203661	-2.906659	3.105922
C	-1.203661	-2.906659	3.105922
C	1.173667	-1.909035	2.131967
C	-1.173667	-1.909035	2.131967
N	-0.000000	-1.437508	1.663177
H	2.167809	-3.270359	3.465817
H	-2.167809	-3.270359	3.465817
H	2.077385	-1.452548	1.702003
H	-2.077385	-1.452548	1.702003
C	0.000000	-2.902588	-3.644371
C	0.000000	-1.512367	-3.742449
C	0.000000	-0.737411	-2.579915
N	0.000000	-1.328972	-1.362278
C	0.000000	-2.660396	-1.261071
C	0.000000	-3.490486	-2.380827
H	0.000000	-3.517095	-4.547086
H	0.000000	-1.035139	-4.721147
H	0.000000	-3.075303	-0.253346
H	0.000000	-4.573722	-2.251636
C	0.000000	2.902588	-3.644371
C	0.000000	3.490486	-2.380827
C	0.000000	2.660396	-1.261071
N	0.000000	1.328972	-1.362278
C	0.000000	0.737411	-2.579915
C	0.000000	1.512367	-3.742449
H	0.000000	3.517095	-4.547086
H	0.000000	4.573722	-2.251636
H	0.000000	3.075303	-0.253346
H	0.000000	1.035139	-4.721147
O	-2.642402	0.000000	-1.353016
O	2.642402	-0.000000	-1.353016
N	-3.467695	0.000000	-0.411744
O	-4.676801	0.000000	-0.614703
O	-3.026568	0.000000	0.792229
N	3.467695	-0.000000	-0.411744
O	4.676801	-0.000000	-0.614703
O	3.026568	-0.000000	0.792229
H	0.000000	-4.198231	4.363284
H	0.000000	4.198231	4.363284

43

CAGEmonocomplexc2v SCF Done: -1119.22366537 A.U.

C	0.000000	2.180366	4.264680
C	-1.199140	2.094354	3.546167
C	1.199140	2.094354	3.546167
C	-1.154171	1.911515	2.167110
C	1.154171	1.911515	2.167110
N	0.000000	1.776592	1.466554
H	-2.166290	2.178183	4.048073
H	2.166290	2.178183	4.048073
H	-2.076715	1.852061	1.581788
H	2.076715	1.852061	1.581788

Pd 0.000000 -0.000000 0.138474
C -0.000000 -2.180366 4.264680
C -1.199140 -2.094354 3.546167
C 1.199140 -2.094354 3.546167
C -1.154171 -1.911515 2.167110
C 1.154171 -1.911515 2.167110
N -0.000000 -1.776592 1.466554
H -2.166290 -2.178183 4.048073
H 2.166290 -2.178183 4.048073
H -2.076715 -1.852061 1.581788
H 2.076715 -1.852061 1.581788
C -0.000000 -2.900562 -4.173918
C -0.000000 -3.506603 -2.914876
C -0.000000 -2.680371 -1.788563
N -0.000000 -1.350087 -1.869829
C -0.000000 -0.748950 -3.071372
C -0.000000 -1.508356 -4.255589
H -0.000000 -3.503905 -5.085215
H -0.000000 -4.593286 -2.804675
H -0.000000 -3.081553 -0.767899
H -0.000000 -1.026477 -5.233472
C 0.000000 2.900562 -4.173918
C 0.000000 1.508356 -4.255589
C 0.000000 0.748950 -3.071372
N 0.000000 1.350087 -1.869829
C 0.000000 2.680371 -1.788563
C 0.000000 3.506603 -2.914876
H 0.000000 3.503905 -5.085215
H 0.000000 1.026477 -5.233472
H 0.000000 3.081553 -0.767899
H 0.000000 4.593286 -2.804675
H 0.000000 2.320984 5.348043
H -0.000000 -2.320984 5.348043
51
CAGEmonocomplex+NO3 SCF Done: -1679.50601192 A.U.
C -3.543549 3.280504 1.063937
C -2.940115 2.512470 2.063891
C -3.177572 3.068846 -0.266561
C -1.988144 1.559914 1.706915
C -2.218046 2.101972 -0.567584
N -1.644940 1.376317 0.414052
H -3.197195 2.640173 3.116838
H -3.620455 3.646144 -1.079907
H -1.484657 0.909827 2.436245
H -1.879253 1.899970 -1.592341
Pd -0.181868 0.000130 0.000923
C -3.534458 -3.288688 -1.063817
C -3.169241 -3.076905 0.266885
C -2.933234 -2.518211 -2.063222
C -2.212516 -2.107506 0.568626
C -1.984044 -1.563140 -1.705555
N -1.641445 -1.379582 -0.412514
H -3.610555 -3.656072 1.079754

H	-3.189949	-2.645896	-3.116258
H	-1.873974	-1.905068	1.593323
H	-1.482773	-0.910452	-2.434202
C	3.644452	-2.862997	-0.468759
C	3.751191	-1.493678	-0.229438
C	2.592581	-0.720829	-0.134156
N	1.373002	-1.290972	-0.283420
C	1.263415	-2.605531	-0.492092
C	2.380307	-3.431174	-0.601678
H	4.543688	-3.478605	-0.540941
H	4.730182	-1.033182	-0.106332
H	0.256626	-3.009524	-0.570987
H	2.245312	-4.499731	-0.772588
C	3.637381	2.873402	0.466016
C	2.371902	3.438008	0.601692
C	1.257134	2.609328	0.493723
N	1.369919	1.295195	0.284085
C	2.590786	0.728454	0.132431
C	3.747435	1.504512	0.225846
H	4.535049	3.491460	0.536750
H	2.234275	4.506088	0.773497
H	0.249358	3.010510	0.574611
H	4.727456	1.046769	0.100682
O	1.354259	0.679429	-2.713364
O	1.361517	-0.682368	2.712059
N	0.339117	1.395747	-2.665743
O	0.396610	2.573854	-2.261591
O	-0.789141	0.915002	-2.993958
N	0.345865	-1.397895	2.664549
O	0.402628	-2.576522	2.261585
O	-0.782189	-0.915943	2.991540
H	-4.280003	-4.045800	-1.320661
H	-4.291311	4.035617	1.320209

45

CAGEmonocomplexPHEN+2Ni SCF Done: -2575.25641807 A.U.

C	3.712146	3.383092	-0.256354
C	3.087821	2.890571	-1.406980
C	3.367121	2.847286	0.987282
C	2.138016	1.880748	-1.276909
C	2.406033	1.839722	1.044821
N	1.811660	1.377298	-0.070112
H	3.329466	3.279895	-2.398085
H	3.831342	3.201409	1.910047
H	1.625754	1.451399	-2.141886
H	2.093565	1.390735	1.990734
Ni	0.414363	-0.000033	-0.000191
C	3.712215	-3.383015	0.256825
C	3.367213	-2.847546	-0.986960
C	3.087763	-2.890278	1.407292
C	2.406045	-1.840073	-1.044803
C	2.137880	-1.880571	1.276914
N	1.811567	-1.377424	0.069977
H	3.831522	-3.201848	-1.909613

H	3.329360	-3.279357	2.398505
H	2.093598	-1.391353	-1.990849
H	1.625485	-1.451114	2.141758
C	-3.410611	-2.830528	-0.253677
C	-3.500424	-1.421981	-0.121561
C	-2.281933	-0.710346	-0.048371
N	-1.066583	-1.319690	-0.066022
C	-1.002178	-2.640223	-0.206533
C	-2.164194	-3.433258	-0.303236
H	-4.323398	-3.428181	-0.318644
H	-0.010016	-3.091927	-0.235240
H	-2.061039	-4.513843	-0.415500
C	-3.410478	2.830596	0.253851
C	-2.164035	3.433306	0.302990
C	-1.002064	2.640241	0.205992
N	-1.066532	1.319702	0.065574
C	-2.281901	0.710384	0.048287
C	-3.500356	1.422045	0.121817
H	-4.323234	3.428267	0.319079
H	-2.060824	4.513895	0.415156
H	-0.009885	3.091930	0.234365
H	4.460737	-4.175724	0.329964
H	4.460595	4.175891	-0.329253
C	-4.733367	-0.681641	-0.061902
C	-4.733331	0.681729	0.062540
H	-5.675165	-1.232396	-0.114525
H	-5.675102	1.232505	0.115460

45
CAGEmonocomplexPHEN+2 SCF Done: -1195.00096196 A.U.

C	-3.861442	-3.346581	0.000029
C	-3.345535	-2.857684	-1.203931
C	-3.345020	-2.858173	1.203971
C	-2.335277	-1.900214	-1.166574
C	-2.334781	-1.900690	1.166576
N	-1.840010	-1.434435	-0.000014
H	-3.717535	-3.210224	-2.167757
H	-3.716610	-3.211112	2.167810
H	-1.905542	-1.496211	-2.086087
H	-1.904660	-1.497047	2.086067
Pd	-0.364882	-0.000004	0.000000
C	-3.861418	3.346601	-0.000018
C	-3.345019	2.858171	-1.203960
C	-3.345505	2.857710	1.203942
C	-2.334796	1.900670	-1.166564
C	-2.335257	1.900229	1.166585
N	-1.840014	1.434426	0.000023
H	-3.716614	3.211105	-2.167798
H	-3.717491	3.210263	2.167769
H	-1.904692	1.497013	-2.086057
H	-1.905521	1.496224	2.086097
C	3.555013	2.840851	0.000101
C	3.629585	1.426108	0.000061
C	2.406147	0.714738	0.000046

N	1.192669	1.343910	0.000063
C	1.147081	2.675890	0.000109
C	2.316019	3.459745	0.000127
H	4.473563	3.433035	0.000128
H	0.160440	3.141670	0.000151
H	2.226533	4.547346	0.000159
C	3.555018	-2.840849	-0.000143
C	2.316026	-3.459746	-0.000193
C	1.147087	-2.675894	-0.000163
N	1.192672	-1.343913	-0.000067
C	2.406148	-0.714739	-0.000017
C	3.629588	-1.426106	-0.000074
H	4.473570	-3.433032	-0.000151
H	2.226543	-4.547347	-0.000252
H	0.160446	-3.141675	-0.000221
H	-4.654907	4.097898	-0.000036
H	-4.654943	-4.097865	0.000049
C	4.861336	0.683862	0.000078
C	4.861338	-0.683859	0.000003
H	5.803366	1.236542	0.000125
H	5.803369	-1.236537	0.000000

53

CAGEmonocomplexPHEN+NO3Ni SCF Done: -3135.95890098 A.U.

C	3.886899	-3.052429	1.147026
C	3.288304	-2.251302	2.124204
C	3.457770	-2.938560	-0.176370
C	2.284144	-1.362243	1.747490
C	2.448155	-2.028167	-0.489339
N	1.886035	-1.264014	0.464865
H	3.588386	-2.309683	3.171986
H	3.886941	-3.551819	-0.970581
H	1.766098	-0.711880	2.456827
H	2.033739	-1.907353	-1.494463
Ni	0.507218	0.000178	0.002391
C	3.884348	3.051859	-1.150991
C	3.463095	2.933884	0.174608
C	3.278232	2.255678	-2.127560
C	2.453832	2.024070	0.490171
C	2.274872	1.366872	-1.748085
N	1.884899	1.264232	-0.463295
H	3.897988	3.543707	0.968347
H	3.571842	2.317700	-3.176961
H	2.044511	1.901660	1.496875
H	1.750437	0.720289	-2.456242
C	-3.276199	2.738391	-0.735693
C	-3.366432	1.373633	-0.382622
C	-2.150800	0.681676	-0.202792
N	-0.932164	1.250696	-0.382606
C	-0.871985	2.540111	-0.692338
C	-2.029202	3.318365	-0.880640
H	-4.187624	3.324295	-0.878088
H	0.117255	2.981414	-0.792218
H	-1.920265	4.374674	-1.129542

C -3.275698 -2.739634 0.737835
C -2.028621 -3.319251 0.883207
C -0.871547 -2.540918 0.694152
N -0.931957 -1.251887 0.383284
C -2.150686 -0.683047 0.203624
C -3.366183 -1.375072 0.383920
H -4.187023 -3.325597 0.880641
H -1.919475 -4.375354 1.132881
H 0.117802 -2.981878 0.794572
O -1.613171 -1.497348 -2.538989
O -1.615412 1.514493 2.538564
N -0.421920 -1.781937 -2.374182
O -0.053376 -2.940044 -2.101338
O 0.458579 -0.858922 -2.434543
N -0.421102 1.783874 2.371606
O -0.037425 2.938559 2.105282
O 0.446637 0.847891 2.422119
H 4.672407 3.758934 -1.422726
H 4.675353 -3.759876 1.416639
C -4.596785 0.658142 -0.180847
C -4.596657 -0.659771 0.182211
H -5.538523 1.193040 -0.324142
H -5.538305 -1.194770 0.325734
53
CAGEmonocomplexPHEN+NO3 SCF Done: -1755.68748460 A.U.
C -3.729961 -3.270251 -1.227278
C -3.108870 -2.465879 -2.187251
C -3.400157 -3.096630 0.117835
C -2.175340 -1.517134 -1.776480
C -2.457118 -2.131761 0.473231
N -1.865664 -1.371496 -0.470136
H -3.339000 -2.561785 -3.249751
H -3.857983 -3.702575 0.901508
H -1.664997 -0.836847 -2.471935
H -2.145023 -1.959530 1.511350
Pd -0.417958 0.000103 -0.000096
C -3.730242 3.269682 1.228326
C -3.400564 3.096533 -0.116875
C -3.108829 2.465179 2.187983
C -2.457364 2.131964 -0.472677
C -2.175149 1.516762 1.776805
N -1.865633 1.371538 0.470374
H -3.858629 3.702598 -0.900314
H -3.338805 2.560752 3.250547
H -2.145377 1.960133 -1.510885
H -1.664504 0.836479 2.472024
C 3.481514 2.794772 0.494846
C 3.560830 1.402242 0.255752
C 2.341566 0.700039 0.142526
N 1.133899 1.305421 0.296573
C 1.077670 2.613848 0.496799
C 2.243586 3.396996 0.605403
H 4.399749 3.380976 0.581778

H	0.089811	3.063352	0.578434
H	2.145619	4.470677	0.769958
C	3.481681	-2.794384	-0.494819
C	2.243794	-3.396613	-0.605752
C	1.077839	-2.613492	-0.497367
N	1.133986	-1.305085	-0.297022
C	2.341608	-0.699690	-0.142689
C	3.560909	-1.401865	-0.255637
H	4.399948	-3.380568	-0.581536
H	2.145885	-4.470281	-0.770419
H	0.090011	-3.063005	-0.579308
O	1.131871	-0.926226	2.658821
O	1.130659	0.925604	-2.659566
N	0.081935	-1.586922	2.593085
O	0.079957	-2.757080	2.158871
O	-1.020969	-1.058503	2.932421
N	0.080894	1.586546	-2.593617
O	0.079333	2.756820	-2.159717
O	-1.022259	1.058267	-2.932411
H	-4.464348	4.022486	1.527271
H	-4.463930	-4.023312	-1.525908
C	4.791872	0.673281	0.118234
C	4.791904	-0.672891	-0.117795
H	5.732932	1.220276	0.211242
H	5.732994	-1.219884	-0.210535

45

CAGEmonocomplexpy+py+2 SCF Done: -1120.01173729 A.U.

C	3.546634	-3.305049	-0.000405
C	3.485545	-2.327493	0.997033
C	2.566905	-3.312765	-0.997562
C	2.456024	-1.391204	0.963430
C	1.560386	-2.351819	-0.963409
N	1.509088	-1.406418	0.000162
H	4.227380	-2.282861	1.796583
H	2.574446	-4.055701	-1.797300
H	2.382234	-0.606497	1.718960
H	0.772226	-2.333351	-1.718716
Pd	-0.000008	-0.000036	0.000123
C	3.305113	3.546411	-0.000025
C	2.327734	3.485050	0.997573
C	3.312596	2.567023	-0.997516
C	1.391362	2.455609	0.963783
C	2.351596	1.560542	-0.963525
N	1.406367	1.509003	0.000184
H	2.283301	4.226619	1.797380
H	4.055389	2.574789	-1.797384
H	0.606774	2.381599	1.719415
H	2.332936	0.772620	-1.719080
C	-3.546448	3.305187	-0.000321
C	-2.566851	3.312799	-0.997591
C	-1.560397	2.351769	-0.963537
N	-1.509058	1.406385	0.000026
C	-2.455894	1.391265	0.963428

C	-3.485319	2.327637	0.997136
H	-4.345820	4.050172	-0.000438
H	-2.382053	0.606559	1.718951
H	-4.227068	2.283100	1.796770
C	-3.305319	-3.546317	0.000101
C	-2.327830	-3.485018	0.997601
C	-1.391364	-2.455673	0.963681
N	-1.406370	-1.509095	0.000038
C	-2.351674	-1.560608	-0.963594
C	-3.312781	-2.566992	-0.997442
H	-4.050382	-4.345617	0.000143
H	-2.283396	-4.226569	1.797426
H	-0.606670	-2.381729	1.719206
H	4.050090	4.345791	-0.000100
H	4.346054	-4.049983	-0.000614
H	-2.332982	-0.772744	-1.719204
H	-4.055638	-2.574730	-1.797252
H	-0.772340	2.333228	-1.718952
H	-2.574415	4.055711	-1.797352

53

CAGEmonocomplexpy+py+NO3 SCF Done: -1680.71582103 A.U.

C	3.428287	3.368751	0.373972
C	3.120817	2.559502	1.470976
C	2.707782	3.201149	-0.807992
C	2.114093	1.606401	1.347718
C	1.710404	2.228522	-0.868544
N	1.434217	1.447696	0.194725
H	3.644299	2.661995	2.422985
H	2.910481	3.803828	-1.694547
H	1.798105	0.986771	2.188772
H	1.132278	2.054689	-1.780550
Pd	0.000001	-0.023512	0.000247
C	-3.430123	3.366297	-0.378404
C	-2.709424	3.201001	0.803769
C	-3.122357	2.555405	-1.474106
C	-1.711548	2.228984	0.865827
C	-2.115129	1.603022	-1.349377
N	-1.435055	1.446574	-0.196199
H	-2.912351	3.805039	1.689346
H	-3.645994	2.656053	-2.426225
H	-1.133265	2.056936	1.778070
H	-1.798939	0.982136	-2.189429
C	-3.483129	-3.291531	-0.783418
C	-2.763739	-2.729598	-1.840207
C	-1.744233	-1.821310	-1.563414
N	-1.441066	-1.473588	-0.294542
C	-2.112677	-2.022006	0.738095
C	-3.144453	-2.935047	0.522269
H	-4.290415	-4.003166	-0.975050
H	-1.812967	-1.741939	1.752018
H	-3.667013	-3.351264	1.385075
C	3.484505	-3.289356	0.786930
C	2.766289	-2.725241	1.843358

C	1.746453	-1.817557	1.565812
N	1.441864	-1.472470	0.296568
C	2.112363	-2.022983	-0.735667
C	3.144388	-2.935574	-0.519110
H	4.292004	-4.000591	0.979143
H	2.977498	-2.981648	2.882947
H	1.125925	-1.391503	2.364450
O	0.629624	-1.209185	-3.337346
O	-0.628481	-1.204828	3.337632
N	0.986374	-0.011845	-3.192560
O	2.182916	0.267394	-3.025098
O	0.110026	0.892774	-3.174906
N	-0.986011	-0.007815	3.192445
O	-2.182801	0.270571	3.025013
O	-0.110294	0.897392	3.174273
H	-4.219132	4.119429	-0.448829
H	4.216911	4.122394	0.443242
H	1.811641	-1.744922	-1.749849
H	3.666014	-3.353535	-1.381638
H	-1.122942	-1.396810	-2.362322
H	-2.973784	-2.988132	-2.879506

43

CAGEmonocomplex SCF Done: -1119.23551278 A.U.

C	4.210589	2.321204	0.465165
C	3.153492	2.570498	1.351433
C	3.934582	1.606698	-0.703613
C	1.885852	2.088417	1.043091
C	2.633040	1.166079	-0.938398
N	1.616785	1.377987	-0.075674
H	3.307148	3.138068	2.272023
H	4.715548	1.394212	-1.436704
H	1.039815	2.259309	1.715589
H	2.387452	0.604715	-1.843201
Pd	-0.029604	-0.070593	0.035531
C	4.102968	-2.160107	-0.525246
C	3.722438	-1.749745	0.756178
C	3.089071	-2.417854	-1.463498
C	2.369364	-1.586317	1.044837
C	1.765309	-2.203085	-1.102272
N	1.389047	-1.753781	0.121014
H	4.466043	-1.566621	1.535292
H	3.324740	-2.782078	-2.466293
H	2.040982	-1.290649	2.044823
H	0.952965	-2.372231	-1.816717
C	-4.160763	-2.656783	0.656098
C	-2.956459	-3.358455	0.442986
C	-1.823610	-2.640736	0.089940
N	-1.821207	-1.308016	-0.112081
C	-2.984397	-0.616323	0.094738
C	-4.164334	-1.279441	0.492449
H	-5.066069	-3.182025	0.969714
H	-2.904363	-4.444062	0.548713
H	-0.857078	-3.134194	-0.046552

H	-5.064660	-0.698106	0.701090
C	-3.860492	2.955075	-0.770868
C	-4.000584	1.588011	-0.581571
C	-2.911510	0.828704	-0.105151
N	-1.702283	1.412574	0.163575
C	-1.573569	2.735159	-0.063146
C	-2.609269	3.546357	-0.502333
H	-4.694113	3.553946	-1.145140
H	-4.937696	1.086815	-0.832045
H	-0.582112	3.153033	0.132169
H	-2.448378	4.619292	-0.626699
H	5.219386	2.681881	0.679204
H	5.155449	-2.299532	-0.782467

282

CAGEphen+12Ni SCF Done: -16555.8549627 A.U.

N	-2.096666	1.561647	5.526702
N	-4.115962	1.268167	4.337913
C	-3.154673	0.825695	5.159916
C	-2.032535	2.803783	5.028177
N	-2.952720	3.318835	4.201414
C	-3.980492	2.520746	3.881578
C	-5.010652	3.041018	2.919847
C	-4.913483	4.331733	2.373070
C	-6.094378	2.246174	2.509874
C	-5.850891	4.752553	1.428748
C	-6.990683	2.739433	1.560713
N	-6.860672	3.963392	1.011846
H	-4.114582	5.006843	2.679818
H	-6.241918	1.249672	2.926210
H	-5.799004	5.754799	0.996296
H	-7.844716	2.141511	1.233117
C	-0.838322	3.646794	5.374646
C	-0.685379	4.944306	4.857524
C	0.183827	3.158595	6.206038
C	0.472203	5.668827	5.145064
C	1.311207	3.945151	6.446664
N	1.464932	5.171173	5.908643
H	-1.461626	5.390045	4.235538
H	0.104213	2.173105	6.664940
H	0.608611	6.682388	4.759973
H	2.114472	3.588623	7.096172
C	-3.242032	-0.589035	5.657681
C	-4.303416	-1.430612	5.284086
C	-2.250982	-1.131885	6.492828
C	-4.311758	-2.759700	5.709596
C	-2.330707	-2.471324	6.876369
N	-3.331704	-3.279408	6.474859
H	-5.119658	-1.055468	4.666727
H	-1.422250	-0.517284	6.844368
H	-5.131109	-3.429643	5.437473
H	-1.575346	-2.912065	7.531641
Ni	-3.323515	-5.221655	6.940957
N	1.259004	-5.758248	1.627787

N	-0.760908	-6.051566	0.439986
C	-0.072822	-5.897752	1.579471
C	1.904230	-5.783224	0.453649
N	1.288622	-5.932571	-0.727143
C	-0.044239	-6.066009	-0.692148
C	-0.779801	-6.188178	-1.996331
C	-0.096988	-6.171355	-3.224260
C	-2.180187	-6.295616	-2.037842
C	-2.832130	-6.335216	-3.271134
N	-2.167462	-6.274117	-4.441926
H	0.991625	-6.125338	-3.254521
H	-2.761279	-6.349152	-1.117200
H	-0.310569	-6.210880	-5.382377
H	-3.919697	-6.426310	-3.326933
C	3.393060	-5.582694	0.457548
C	4.130136	-5.560017	-0.738508
C	4.099901	-5.382502	1.655305
C	5.500704	-5.300183	-0.698635
C	5.471540	-5.128910	1.611997
N	6.158491	-5.066669	0.454212
H	3.642413	-5.744153	-1.695763
H	3.587954	-5.424395	2.616697
H	6.094766	-5.285525	-1.615746
H	6.042430	-4.978107	2.531539
C	-0.841052	-5.827412	2.868556
C	-2.242422	-5.927643	2.890028
C	-0.189039	-5.630471	4.097548
C	-2.925096	-5.785700	4.098903
C	-0.943078	-5.498845	5.264468
N	-2.289657	-5.553276	5.264420
H	-2.800526	-6.115795	1.972842
H	0.898645	-5.580501	4.148177
H	-4.014039	-5.867666	4.140029
H	-0.456593	-5.352924	6.232068
Ni	8.091796	-4.564971	0.441200
Ni	-8.091291	4.564937	-0.441850
Ni	3.158434	6.196362	6.174769
N	-2.838909	2.656124	-4.718427
N	-1.953385	0.723920	-5.747710
C	-1.899219	2.026138	-5.436531
C	-3.876259	1.912394	-4.310611
N	-4.003002	0.606041	-4.580635
C	-3.022047	0.048545	-5.303447
C	-3.099894	-1.424334	-5.588778
C	-2.089768	-2.083044	-6.309956
C	-4.171741	-2.203180	-5.120957
C	-2.162671	-3.464634	-6.492967
C	-4.172201	-3.580601	-5.345139
N	-3.174717	-4.206144	-6.000843
H	-1.251490	-1.525799	-6.728147
H	-5.002123	-1.742210	-4.586087
H	-1.392416	-3.996151	-7.057241
H	-4.999160	-4.204331	-4.996854

C	-4.929342	2.567275	-3.462675
C	-6.026174	1.840511	-2.969627
C	-4.841929	3.924072	-3.107980
C	-6.945095	2.466863	-2.126521
C	-5.802037	4.478029	-2.260096
N	-6.825051	3.757886	-1.758997
H	-6.166443	0.793878	-3.239817
H	-4.032965	4.547380	-3.489002
H	-7.809535	1.922990	-1.737735
H	-5.757896	5.532733	-1.977522
C	-0.694927	2.810741	-5.873483
C	0.347774	2.206300	-6.595803
C	-0.553384	4.171197	-5.551855
C	1.482338	2.950689	-6.921654
C	0.612509	4.847337	-5.914009
N	1.624042	4.243573	-6.568644
H	0.278537	1.163196	-6.904440
H	-1.344866	4.702969	-5.023756
H	2.301678	2.503052	-7.489552
H	0.740440	5.907323	-5.681044
Ni	3.325358	5.221752	-6.940383
N	4.812211	3.765070	-0.218266
N	5.697561	1.832904	-1.247733
C	5.128231	3.044663	-1.303015
C	5.099660	3.213387	0.968560
N	5.667968	2.007816	1.107153
C	5.953883	1.349651	-0.024500
C	6.548269	-0.025699	0.085000
C	6.838179	-0.789143	-1.058429
C	6.810161	-0.610832	1.335355
C	-0.821332	-6.215212	-4.416303
C	7.295751	-1.917759	1.396534
N	7.528247	-2.654486	0.292264
H	6.689093	-0.375367	-2.055764
H	6.638724	-0.054100	2.256641
H	7.513679	-2.389768	2.357743
C	4.718540	3.965651	2.211825
C	4.087087	5.218968	2.142433
C	4.953819	3.431255	3.489869
C	3.684129	5.853761	3.318130
C	4.520584	4.128081	4.618721
N	3.876948	5.309216	4.535731
H	3.910049	5.699887	1.180404
H	5.471498	2.479310	3.607823
H	3.198419	6.832228	3.288037
H	4.699705	3.734837	5.622456
C	4.779983	3.604360	-2.652941
C	5.048546	2.888515	-3.831825
C	4.147459	4.852057	-2.785262
C	4.645621	3.410222	-5.061882
C	3.775785	5.305498	-4.051688
N	4.000435	4.588444	-5.170442
H	5.568755	1.931370	-3.794827

H	3.945417	5.468687	-1.909439
H	4.851107	2.874257	-5.991788
H	3.289713	6.275977	-4.178624
Ni	-3.158713	-6.195934	-6.174211
C	7.322764	-2.089876	-0.914059
H	7.562104	-2.698673	-1.789443
C	7.115315	7.713304	-8.353578
C	7.181773	7.084855	-7.117265
C	6.088698	6.334575	-6.650393
N	4.961689	6.195298	-7.359127
C	4.888161	6.811878	-8.579180
C	5.937005	7.586752	-9.130252
H	7.959297	8.300068	-8.728064
H	8.076864	7.165111	-6.497449
H	6.137059	5.839835	-5.680684
C	2.258104	7.008282	-11.210906
C	3.498809	7.232893	-10.564504
C	3.674656	6.635790	-9.293002
N	2.721553	5.870234	-8.676868
C	1.560402	5.677215	-9.314318
C	1.296876	6.230547	-10.579257
H	2.065100	7.444522	-12.195426
H	0.808533	5.066349	-8.815329
H	0.332109	6.040755	-11.053602
C	-11.368922	6.222837	-3.454621
C	-10.271799	5.564846	-3.994250
C	-9.272202	5.054306	-3.148164
N	-9.331136	5.176331	-1.816440
C	-10.409756	5.823710	-1.276452
C	-11.463289	6.369743	-2.048505
H	-12.153575	6.624636	-4.102474
H	-10.172852	5.436890	-5.073897
H	-8.409671	4.537284	-3.567900
C	-11.443650	6.639470	2.208883
C	-11.500869	6.578817	0.794414
C	-10.428454	5.927790	0.138468
N	-9.365604	5.368512	0.795470
C	-9.341809	5.442820	2.131794
C	-10.362280	6.069710	2.867558
H	-12.244273	7.130176	2.770174
H	-8.491582	4.994410	2.645110
H	-10.291919	6.101326	3.956526
C	-3.620862	-9.682782	8.546640
C	-4.182713	-8.574553	9.227667
C	-4.014423	-7.303238	8.627989
N	-3.348997	-7.103795	7.448347
C	-2.830045	-8.171631	6.830066
C	-2.947647	-9.471917	7.350757
H	-3.719206	-10.689961	8.962278
H	-2.302411	-8.002087	5.891823
H	-2.504820	-10.306940	6.804458
C	-5.751882	-5.035591	11.019800
C	-5.529414	-3.841412	10.347292

C	-4.816584	-3.839086	9.135862
N	-4.331772	-4.960556	8.588940
C	-4.546838	-6.142230	9.245833
C	-5.252458	-6.241808	10.469098
H	-6.305216	-5.050618	11.963426
H	-5.902485	-2.896949	10.748051
H	-4.639903	-2.903888	8.605162
C	-5.486227	-6.619544	-10.293903
C	-5.002125	-7.730113	-9.559106
C	-4.326503	-7.449457	-8.347109
N	-4.125909	-6.183266	-7.866853
C	-4.595748	-5.155927	-8.584967
C	-5.278713	-5.338670	-9.799815
H	-6.016291	-6.774964	-11.238209
H	-4.430981	-4.152250	-8.193925
H	-5.640651	-4.464569	-10.344562
C	-3.423337	-10.845943	-7.110168
C	-2.779253	-10.459331	-5.942471
C	-2.672524	-9.096159	-5.616797
N	-3.174634	-8.132455	-6.398475
C	-3.811164	-8.505346	-7.551684
C	-3.966660	-9.851641	-7.960944
H	-3.512984	-11.903671	-7.374764
H	-2.351165	-11.203569	-5.268192
H	-2.167733	-8.788800	-4.701282
C	1.986683	8.594501	10.104680
C	1.040283	7.729909	9.571166
C	1.334765	6.995558	8.409398
N	2.512090	7.094030	7.780059
C	3.450720	7.944278	8.299821
C	3.243853	8.723002	9.463846
H	1.769598	9.171597	11.008379
H	0.063422	7.610785	10.043770
H	0.594656	6.315973	7.987680
C	6.916655	8.875907	7.299230
C	5.718559	8.864276	8.055604
C	4.682368	8.014606	7.598923
N	4.785630	7.223932	6.486148
C	5.930749	7.258126	5.793770
C	7.013272	8.071004	6.172008
H	7.752157	9.512981	7.604041
H	6.002767	6.625027	4.909783
H	7.924138	8.059654	5.570348
C	10.005048	-8.899525	0.787066
C	8.619903	-8.806619	0.763239
C	8.001387	-7.548485	0.662118
N	8.702481	-6.410709	0.586009
C	10.068748	-6.493716	0.608852
C	10.778210	-7.714753	0.708378
H	10.498386	-9.872819	0.865507
H	7.999028	-9.702622	0.822352
H	6.914579	-7.471475	0.643211
C	12.804967	-3.969595	0.454837

C	12.183685	-5.240080	0.541604
C	10.768256	-5.262061	0.525856
N	9.993769	-4.137057	0.432787
C	10.611718	-2.952352	0.352412
C	12.012258	-2.833551	0.360753
H	13.896070	-3.890382	0.462336
H	9.986197	-2.063117	0.278754
H	12.463509	-1.841816	0.292668
C	-4.655347	-10.122583	-9.195004
C	-5.153006	-9.102927	-9.963128
C	-12.546901	7.028940	-1.368910
C	-12.564980	7.129413	-0.002539
C	-5.416086	-7.540114	11.067626
C	-4.901941	-8.661290	10.470967
C	4.572974	8.017886	-11.112744
C	5.744839	8.187952	-10.423408
C	12.216622	-7.677836	0.723285
C	12.892127	-6.488447	0.643119
C	4.305375	9.582451	9.916764
C	5.494789	9.650325	9.239957
H	12.766225	-8.619589	0.800062
H	13.985052	-6.473555	0.655408
H	6.553515	8.786625	-10.850595
H	4.439113	8.479737	-12.094391
H	-13.361300	7.451217	-1.963296
H	-13.393913	7.632508	0.502060
H	6.293620	10.307040	9.594148
H	4.147547	10.184572	10.815318
H	-5.960604	-7.619069	12.012148
H	-5.032935	-9.642020	10.935580
H	-5.674350	-9.321679	-10.898652
H	-4.776403	-11.161457	-9.512719

282

CAGEphen+12Pd SCF Done: -8274.28465729 A.U.

N	4.768573	2.299169	3.281092
N	5.883069	0.731360	1.911140
C	5.475388	1.977065	2.189300
C	4.475789	1.295592	4.119224
N	4.846684	0.024824	3.912035
C	5.550666	-0.216612	2.797824
C	5.945982	-1.634799	2.501877
C	5.599780	-2.685718	3.367039
C	6.652580	-1.968250	1.334687
C	5.922580	-3.999213	3.022827
C	6.939860	-3.305952	1.059002
N	6.563155	-4.311640	1.876605
H	5.081050	-2.486364	4.304698
H	6.978777	-1.193095	0.641317
H	5.663775	-4.827610	3.686728
H	7.492090	-3.581681	0.157341
C	3.644553	1.603309	5.331440
C	3.268309	0.595629	6.234663
C	3.188190	2.905587	5.593366

C	2.436120	0.907527	7.310839
C	2.358668	3.139579	6.691142
N	1.967747	2.154750	7.527247
H	3.619211	-0.427986	6.103856
H	3.474796	3.735809	4.947909
H	2.137554	0.136028	8.024722
H	1.998247	4.147441	6.910901
C	5.785009	3.062587	1.198895
C	6.489770	2.792023	0.014453
C	5.357371	4.384380	1.405737
C	6.696428	3.812251	-0.915278
C	5.602204	5.350905	0.429089
N	6.242059	5.068947	-0.725408
H	6.876912	1.793173	-0.186668
H	4.835661	4.663436	2.321148
H	7.246084	3.619153	-1.839709
H	5.279503	6.384424	0.576354
Pd	6.451573	6.543036	-2.242004
N	-0.336390	4.892843	-3.838306
N	0.777745	3.324681	-5.208139
C	0.777627	4.363552	-4.361912
C	-1.494592	4.328918	-4.206965
N	-1.578047	3.288736	-5.047443
C	-0.419985	2.816419	-5.528238
C	-0.463696	1.621211	-6.436357
C	-1.680463	1.012428	-6.785153
C	0.712310	1.049438	-6.949083
C	0.635960	-0.103485	-7.731973
N	-0.536482	-0.705035	-8.023998
H	-2.628580	1.429817	-6.446197
H	1.684486	1.496522	-6.741675
H	-2.614201	-0.622681	-7.856890
H	1.541063	-0.558403	-8.141630
C	-2.764751	4.859978	-3.607143
C	-4.012213	4.294357	-3.918121
C	-2.751119	5.924413	-2.690858
C	-5.162911	4.768486	-3.286416
C	-3.944356	6.343627	-2.100589
N	-5.131625	5.762851	-2.374260
H	-4.091222	3.488396	-4.647650
H	-1.818067	6.426614	-2.435441
H	-6.141392	4.341890	-3.520026
H	-3.951399	7.172758	-1.388915
C	2.100757	4.934063	-3.938874
C	3.311022	4.405835	-4.417432
C	2.179379	5.999452	-3.026972
C	4.521909	4.915872	-3.946722
C	3.428438	6.455846	-2.603249
N	4.584613	5.910754	-3.036751
H	3.314676	3.601136	-5.152602
H	1.274843	6.473670	-2.646234
H	5.472073	4.518583	-4.311870
H	3.506927	7.286234	-1.897359

Pd	-6.891520	6.339786	-1.331769
Pd	6.892250	-6.339333	1.331577
Pd	0.581768	2.541190	9.091397
N	0.041884	-6.180259	-0.765579
N	-1.277448	-5.510130	-2.606014
C	-1.143058	-5.961473	-1.351585
C	1.129158	-5.926711	-1.506468
N	1.078329	-5.474181	-2.766622
C	-0.143375	-5.280307	-3.281585
C	-0.246736	-4.728695	-4.674481
C	-1.493310	-4.465382	-5.265683
C	0.899529	-4.428750	-5.428737
C	-1.548081	-3.886257	-6.534370
C	0.764064	-3.850868	-6.691937
N	-0.438845	-3.561647	-7.231499
H	-2.418886	-4.708281	-4.743824
H	1.894297	-4.642222	-5.037716
H	-2.509766	-3.678681	-7.009823
H	1.645599	-3.615068	-7.293005
C	2.478176	-6.112856	-0.873527
C	3.660746	-5.832472	-1.577589
C	2.608217	-6.547794	0.455653
C	4.893177	-5.949609	-0.933114
C	3.876150	-6.640755	1.031582
N	5.002927	-6.326232	0.358352
H	3.626246	-5.523953	-2.622411
H	1.728996	-6.813335	1.042585
H	5.822505	-5.739035	-1.467900
H	3.994715	-6.981182	2.063103
C	-2.387446	-6.187283	-0.541879
C	-3.662482	-5.942914	-1.077945
C	-2.322776	-6.624681	0.791279
C	-4.791910	-6.096019	-0.272411
C	-3.497410	-6.754820	1.533775
N	-4.713995	-6.474526	1.020812
H	-3.779368	-5.634505	-2.116811
H	-1.364503	-6.863372	1.252554
H	-5.791106	-5.913711	-0.675347
H	-3.464645	-7.097690	2.570766
Pd	-6.452396	-6.542502	2.241569
N	-4.268363	-0.114150	4.533541
N	-5.587766	0.556540	2.693366
C	-5.109522	-0.379084	3.524775
C	-3.911382	1.167688	4.691031
N	-4.345519	2.160199	3.902494
C	-5.183980	1.814589	2.916146
C	-5.657931	2.888182	1.979200
C	-6.508923	2.594055	0.901193
C	-5.246035	4.222662	2.128531
C	-1.676114	-0.139265	-7.573539
C	-5.650572	5.179345	1.196294
N	-6.432938	4.875865	0.139148
H	-6.889600	1.583587	0.752638

H	-4.613198	4.519219	2.964951
H	-5.342068	6.222408	1.300351
C	-2.932964	1.502992	5.779779
C	-2.406959	0.509049	6.621323
C	-2.485175	2.819101	5.980112
C	-1.446195	0.848343	7.575153
C	-1.521798	3.080494	6.955575
N	-0.991089	2.109719	7.728697
H	-2.740996	-0.525054	6.537184
H	-2.881974	3.638894	5.381345
H	-1.029969	0.087716	8.240122
H	-1.165823	4.099324	7.126609
C	-5.498278	-1.809190	3.282322
C	-6.346439	-2.166225	2.221218
C	-5.006179	-2.847393	4.090261
C	-6.627920	-3.512555	1.984303
C	-5.332824	-4.170758	3.790297
N	-6.113380	-4.504725	2.741031
H	-6.787067	-1.402704	1.580193
H	-4.371206	-2.630534	4.949220
H	-7.288904	-3.806708	1.165429
H	-4.961355	-4.989634	4.411132
Pd	-0.581178	-2.542517	-9.091069
C	-6.870895	3.605667	0.010354
H	-7.535256	3.394199	-0.831009
C	-10.467296	-7.367487	4.816334
C	-9.909345	-6.098543	4.878375
C	-8.743333	-5.810275	4.148326
N	-8.142240	-6.727839	3.384156
C	-8.678573	-7.986751	3.310254
C	-9.851125	-8.359828	4.014162
H	-11.374498	-7.605770	5.379264
H	-10.363715	-5.315587	5.488523
H	-8.294721	-4.817131	4.188385
C	-7.787479	-11.139671	1.524896
C	-8.506835	-10.252065	2.363101
C	-8.007776	-8.930966	2.486351
N	-6.880589	-8.503761	1.834475
C	-6.220586	-9.361416	1.049615
C	-6.648526	-10.688598	0.873212
H	-8.131982	-12.170002	1.396754
H	-5.327235	-8.994389	0.543256
H	-6.075793	-11.351434	0.221767
C	8.255008	-10.895614	0.430160
C	7.025040	-10.480219	-0.059745
C	6.585407	-9.166138	0.176012
N	7.319918	-8.287539	0.865742
C	8.537501	-8.679443	1.357560
C	9.054614	-9.985189	1.164780
H	8.609675	-11.915381	0.254172
H	6.389242	-11.161233	-0.628723
H	5.620906	-8.827061	-0.203418
C	11.242542	-7.038352	3.335465

C	10.553243	-8.050271	2.622168
C	9.285293	-7.713936	2.084849
N	8.726374	-6.471586	2.233720
C	9.397823	-5.534932	2.911222
C	10.660311	-5.786644	3.475527
H	12.224529	-7.248194	3.769463
H	8.929168	-4.555652	3.014048
H	11.169592	-4.989236	4.020071
C	7.538332	10.061606	-5.383354
C	8.380464	9.712602	-4.298500
C	7.943964	8.662357	-3.452331
N	6.763069	7.996176	-3.651541
C	5.987139	8.345170	-4.682713
C	6.346610	9.375438	-5.568710
H	7.830626	10.865587	-6.065164
H	5.053424	7.798093	-4.817210
H	5.679501	9.623986	-6.396324
C	10.711182	8.450191	-0.958322
C	10.207376	7.414678	-0.184195
C	8.974045	6.828253	-0.516857
N	8.256842	7.237566	-1.568171
C	8.738164	8.259019	-2.344669
C	9.972049	8.904280	-2.078764
H	11.669698	8.915794	-0.711042
H	10.756343	7.045621	0.684243
H	8.566918	6.013792	0.083227
C	-0.727709	-6.330685	-12.100659
C	-0.779525	-4.988315	-12.551514
C	-0.727970	-3.971849	-11.564760
N	-0.632121	-4.247044	-10.225706
C	-0.584949	-5.522208	-9.827064
C	-0.630730	-6.589256	-10.740835
H	-0.764259	-7.151384	-12.823018
H	-0.508786	-5.711694	-8.755844
H	-0.589140	-7.613942	-10.366612
C	-0.917145	-0.861987	-13.617938
C	-0.861263	0.065164	-12.587091
C	-0.763335	-0.373926	-11.255447
N	-0.721352	-1.672357	-10.940031
C	-0.775395	-2.602960	-11.944547
C	-0.874536	-2.245053	-13.312618
H	-0.993452	-0.534427	-14.658883
H	-0.892283	1.136524	-12.794406
H	-0.718633	0.344292	-10.436083
C	3.654385	3.637759	12.667191
C	4.192284	3.313077	11.430008
C	3.340655	2.987353	10.360291
N	2.010388	2.978571	10.492565
C	1.460595	3.296512	11.706913
C	2.247471	3.636491	12.835975
H	4.305297	3.893274	13.508447
H	5.272545	3.306370	11.272702
H	3.750539	2.730383	9.382970

C	-2.010282	3.551172	13.052535
C	-0.594089	3.593067	13.029281
C	0.042655	3.274837	11.803380
N	-0.656562	2.937767	10.674016
C	-1.992132	2.905775	10.723079
C	-2.700607	3.207678	11.898912
H	-2.548772	3.788531	13.974692
H	-2.522507	2.634349	9.809715
H	-3.791510	3.167770	11.889306
C	-8.502910	9.815785	-4.289838
C	-7.326869	9.166162	-4.636678
C	-6.818706	8.149644	-3.809563
N	-7.436149	7.779468	-2.683102
C	-8.598951	8.409499	-2.324025
C	-9.178571	9.443742	-3.101154
H	-8.909811	10.608710	-4.924179
H	-6.786428	9.433085	-5.546859
H	-5.895515	7.631243	-4.070678
C	-10.993193	8.118925	0.523021
C	-10.427768	8.592542	-0.686888
C	-9.222315	7.984735	-1.119313
N	-8.608646	6.980498	-0.417234
C	-9.163119	6.552169	0.721307
C	-10.357103	7.101394	1.219723
H	-11.922966	8.555580	0.899237
H	-8.653284	5.752124	1.259071
H	-10.771127	6.717974	2.154243
C	-0.925698	-3.288927	-14.300723
C	-0.880026	-4.607637	-13.934852
C	10.342538	-10.309643	1.716705
C	11.062957	-9.379499	2.417262
C	10.399812	9.967988	-2.947202
C	9.634718	10.356561	-4.014243
C	-9.697532	-10.613838	3.084219
C	-10.343756	-9.704215	3.877885
C	-10.401738	10.049562	-2.647858
C	-11.002243	9.640381	-1.487297
C	1.585273	3.956624	14.071955
C	0.219309	3.935751	14.164879
H	-10.847162	10.846914	-3.248355
H	-11.931742	10.107889	-1.152278
H	-11.247974	-9.990085	4.421428
H	-10.080834	-11.632949	2.987983
H	10.739657	-11.316951	1.567050
H	12.040799	-9.637032	2.832316
H	-0.273412	4.180851	15.109294
H	2.193646	4.218553	14.941463
H	11.351469	10.464401	-2.740583
H	9.969634	11.166209	-4.667749
H	-0.919670	-5.392767	-14.694305
H	-1.002167	-3.011058	-15.355099

282

CAGEphen+12Zn SCF Done: -18181.9995400 A.U.

N	5.943104	-2.514132	-0.578837
N	4.851511	-4.245297	0.598780
C	5.289300	-3.682762	-0.535511
C	6.156511	-1.906672	0.596033
N	5.753391	-2.405467	1.772407
C	5.105113	-3.577151	1.732060
C	4.574959	-4.139165	3.020598
C	4.802721	-3.495458	4.248254
C	3.796300	-5.308351	3.039271
C	4.208555	-3.997323	5.407081
C	3.236467	-5.740662	4.241569
N	3.410334	-5.083681	5.405778
H	5.435433	-2.609416	4.301867
H	3.622348	-5.873539	2.123694
H	4.377418	-3.513196	6.372375
H	2.626086	-6.646696	4.275305
C	6.824218	-0.560794	0.587487
C	7.004987	0.170934	1.773018
C	7.254626	0.033853	-0.610237
C	7.535353	1.459766	1.709770
C	7.777463	1.327406	-0.585110
N	7.889388	2.047201	0.549208
H	6.729590	-0.257360	2.736743
H	7.180274	-0.504168	-1.555318
H	7.682963	2.044502	2.621316
H	8.120083	1.805938	-1.506111
C	4.971022	-4.366495	-1.834960
C	4.269836	-5.583347	-1.867829
C	5.328125	-3.793664	-3.066974
C	3.907730	-6.133819	-3.097905
C	4.925608	-4.409416	-4.252409
N	4.199930	-5.545280	-4.275028
H	4.005200	-6.096801	-0.943435
H	5.910318	-2.872897	-3.102738
H	3.364313	-7.081033	-3.144012
H	5.193957	-3.978566	-5.220380
Zn	3.405949	-6.294008	-6.097314
N	-1.805609	-1.395749	-6.068481
N	-2.888986	-3.126846	-4.882950
C	-1.864286	-2.650596	-5.602769
C	-2.846556	-0.603480	-5.779258
N	-3.907044	-1.004225	-5.065308
C	-3.892475	-2.273759	-4.637640
C	-5.031854	-2.746229	-3.779744
C	-6.095471	-1.892296	-3.443628
C	-5.055658	-4.046754	-3.249084
C	-6.080523	-4.413079	-2.376026
N	-7.064486	-3.567447	-2.009865
H	-6.153079	-0.886870	-3.860657
H	-4.280245	-4.766952	-3.510091
H	-7.918002	-1.689019	-2.291360
H	-6.116522	-5.420699	-1.954092
C	-2.790753	0.831156	-6.222077

C	-3.811665	1.737099	-5.889537
C	-1.697010	1.329531	-6.949272
C	-3.673218	3.082633	-6.231277
C	-1.637592	2.690140	-7.253308
N	-2.591551	3.564508	-6.875555
H	-4.704642	1.398311	-5.364328
H	-0.896305	0.664653	-7.273233
H	-4.456767	3.802285	-5.980751
H	-0.796094	3.096158	-7.820462
C	-0.686550	-3.552081	-5.842731
C	-0.656473	-4.863175	-5.339112
C	0.445246	-3.103614	-6.543709
C	0.497104	-5.632207	-5.495790
C	1.557979	-3.937925	-6.656204
N	1.602175	-5.172212	-6.115935
H	-1.522409	-5.280207	-4.824998
H	0.461851	-2.111247	-6.994304
H	0.539296	-6.654477	-5.111331
H	2.447271	-3.607210	-7.198807
Zn	-2.327361	5.655523	-7.140609
Zn	2.327546	-5.655610	7.141679
Zn	8.450298	4.095213	0.493299
N	-2.765672	-0.818292	5.793496
N	-4.683491	-0.545132	4.443542
C	-3.744310	-0.067347	5.270969
C	-2.762338	-2.114176	5.453399
N	-3.666315	-2.667729	4.634124
C	-4.610787	-1.850192	4.149572
C	-5.601258	-2.412444	3.169737
C	-6.598499	-1.606647	2.595614
C	-5.540401	-3.754766	2.759835
C	-7.433129	-2.142395	1.614086
C	-6.413443	-4.210725	1.771694
N	-7.327129	-3.414652	1.181152
H	-6.721817	-0.569431	2.907229
H	-4.815522	-4.437820	3.202613
H	-8.216045	-1.531767	1.157130
H	-6.381326	-5.251642	1.439922
C	-1.645232	-2.978642	5.965308
C	-1.573423	-4.345355	5.648650
C	-0.608719	-2.441600	6.746857
C	-0.466934	-5.088552	6.062325
C	0.463272	-3.253953	7.116670
N	0.554653	-4.550204	6.758011
H	-2.370746	-4.827014	5.082501
H	-0.633637	-1.397760	7.059537
H	-0.394943	-6.154016	5.829333
H	1.279787	-2.853299	7.722778
C	-3.749535	1.403611	5.576928
C	-4.705562	2.263347	5.010866
C	-2.774909	1.979903	6.408465
C	-4.614856	3.636584	5.240314
C	-2.755994	3.363691	6.586718

N	-3.639744	4.188398	5.989929
H	-5.511161	1.867260	4.392663
H	-2.034587	1.356867	6.910363
H	-5.349659	4.321202	4.809048
H	-2.006757	3.830347	7.231300
Zn	-3.406217	6.297216	6.094603
N	1.631291	6.189489	1.020447
N	-0.281558	6.467694	-0.335940
C	0.319311	6.408502	0.860093
C	2.349446	6.026501	-0.098756
N	1.822696	6.068262	-1.329864
C	0.504347	6.293904	-1.406921
C	-0.138541	6.285069	-2.764863
C	-1.519755	6.488305	-2.920996
C	0.612128	6.032868	-3.925208
C	-7.082017	-2.338410	-2.563501
C	-0.038636	5.932464	-5.155136
N	-1.373229	6.068433	-5.288108
H	-2.145709	6.731159	-2.062303
H	1.693849	5.910727	-3.870597
H	0.528569	5.736579	-6.068655
C	3.812531	5.712654	0.036352
C	4.422785	5.604451	1.297045
C	4.615375	5.476145	-1.091944
C	5.759420	5.212957	1.381196
C	5.944695	5.089031	-0.919414
N	6.505934	4.927501	0.295565
H	3.860765	5.819053	2.205999
H	4.207718	5.588414	-2.096570
H	6.251826	5.124668	2.352972
H	6.585131	4.901568	-1.785054
C	-0.535633	6.526319	2.089912
C	-1.926956	6.697166	1.997711
C	0.018902	6.427761	3.376877
C	-2.696723	6.698822	3.161385
C	-0.822850	6.439715	4.489624
N	-2.163338	6.541496	4.389422
H	-2.407563	6.822725	1.027492
H	1.096831	6.337896	3.511407
H	-3.780463	6.831021	3.109868
H	-0.410038	6.365338	5.498927
Zn	-8.449350	-4.096920	-0.488427
C	-2.091532	6.373044	-4.188721
H	-3.163793	6.530445	-4.330637
C	-7.109520	9.232177	6.990245
C	-7.189529	8.488213	5.821895
C	-6.143289	7.611553	5.499041
N	-5.062637	7.459130	6.273326
C	-4.965755	8.184954	7.430853
C	-5.979101	9.096028	7.834659
H	-7.911459	9.923363	7.265854
H	-8.050668	8.576981	5.156896
H	-6.185099	7.014874	4.583288

C	-2.515041	8.557401	10.245483
C	-3.680164	8.758360	9.463482
C	-3.797107	8.013318	8.258771
N	-2.830093	7.131300	7.854924
C	-1.742577	6.965396	8.616825
C	-1.546758	7.659540	9.819810
H	-2.387390	9.111968	11.179811
H	-0.992555	6.252819	8.261712
H	-0.641815	7.489023	10.406042
C	4.369127	-5.776648	11.494729
C	4.311123	-4.532929	10.882590
C	3.728696	-4.425469	9.611125
N	3.222507	-5.477032	8.956404
C	3.269990	-6.712953	9.544929
C	3.842104	-6.912655	10.830552
H	4.818022	-5.888555	12.486081
H	4.710332	-3.644614	11.375736
H	3.671416	-3.455016	9.109822
C	2.210624	-10.207659	8.689679
C	2.761754	-9.129753	9.427094
C	2.720344	-7.839852	8.831771
N	2.171863	-7.629629	7.594384
C	1.660596	-8.669310	6.924864
C	1.660290	-9.974890	7.437551
H	2.223235	-11.216250	9.113227
H	1.232690	-8.462429	5.939744
H	1.230724	-10.789940	6.852035
C	6.116634	-6.835110	-10.033044
C	5.479739	-7.902736	-9.351880
C	4.710959	-7.579567	-8.200998
N	4.580527	-6.291279	-7.754397
C	5.195102	-5.307007	-8.420752
C	5.972826	-5.536927	-9.565164
H	6.717578	-7.042387	-10.923383
H	5.068075	-4.291886	-8.033828
H	6.454407	-4.699597	-10.073715
C	3.498116	-10.978947	-7.181309
C	2.756666	-10.626208	-6.062933
C	2.687019	-9.276056	-5.689376
N	3.308490	-8.304862	-6.368511
C	4.045074	-8.633574	-7.475562
C	4.169592	-9.976218	-7.924952
H	3.569177	-12.024672	-7.494690
H	2.231487	-11.381751	-5.475573
H	2.108452	-8.974688	-4.811384
C	-10.729687	-8.331395	-0.474765
C	-11.380236	-7.077081	-0.588584
C	-10.565036	-5.912911	-0.576612
N	-9.202068	-5.983277	-0.460473
C	-8.619846	-7.183426	-0.354625
C	-9.348173	-8.382136	-0.357666
H	-11.321873	-9.251227	-0.479884
H	-7.530003	-7.199352	-0.263255

H	-8.826309	-9.336806	-0.268589
C	-13.160053	-3.226808	-0.919067
C	-12.332944	-2.112973	-0.903971
C	-10.947560	-2.294347	-0.780880
N	-10.382939	-3.503047	-0.676445
C	-11.183198	-4.614579	-0.689555
C	-12.596359	-4.522901	-0.810680
H	-14.244031	-3.113751	-1.013997
H	-12.745286	-1.105563	-0.986176
H	-10.276271	-1.430878	-0.766855
C	11.719962	5.931143	3.504941
C	10.566209	5.429116	4.089482
C	9.570416	4.878712	3.269209
N	9.683876	4.815382	1.937402
C	10.815777	5.305806	1.342065
C	11.874125	5.880609	2.096710
H	12.512289	6.365882	4.121402
H	10.426240	5.457573	5.171734
H	8.652744	4.476889	3.708258
C	12.164119	5.643881	-2.141389
C	12.096462	5.736832	-0.728533
C	10.928862	5.232573	-0.094065
N	9.900015	4.675256	-0.806101
C	9.996067	4.603115	-2.138871
C	11.111791	5.076101	-2.845099
H	13.046194	6.020747	-2.667430
H	9.154617	4.152142	-2.672734
H	11.142527	4.994366	-3.933234
C	-5.811603	8.467960	-8.895920
C	-6.085743	7.837484	-7.690828
C	-5.113148	7.002269	-7.121764
N	-3.923006	6.784321	-7.693361
C	-3.634262	7.397646	-8.883589
C	-4.561045	8.259714	-9.530080
H	-6.552429	9.124597	-9.361547
H	-7.042150	7.983121	-7.185118
H	-5.307539	6.494662	-6.172582
C	-0.758399	7.517410	-11.288158
C	-2.032637	7.783955	-10.727201
C	-2.349072	7.155719	-9.492209
N	-1.467873	6.322157	-8.856002
C	-0.273002	6.092232	-9.412810
C	0.120612	6.670555	-10.628524
H	-0.476781	7.981610	-12.237902
H	0.403625	5.421791	-8.875152
H	1.106851	6.451371	-11.041994
C	-13.392073	-5.719372	-0.819496
C	-12.806185	-6.949865	-0.712590
C	3.867975	-8.229731	11.404545
C	3.347664	-9.297836	10.728356
C	4.954332	-10.269327	-9.092457
C	5.585506	-9.270398	-9.779889
C	-4.722674	9.671499	9.843194

C	-5.830207	9.834168	9.058507
C	-4.210084	8.879430	-10.778142
C	-2.992042	8.650196	-11.354885
C	13.041304	6.381335	1.424546
C	13.148390	6.312172	0.063455
H	-4.935417	9.539338	-11.260766
H	-2.731326	9.124531	-12.304380
H	-6.619578	10.530360	9.352982
H	-4.615472	10.235987	10.772905
H	4.312025	-8.364302	12.394097
H	3.370635	-10.297095	11.170469
H	14.038829	6.694868	-0.441686
H	13.845062	6.820007	2.021248
H	5.038819	-11.307578	-9.423221
H	6.180965	-9.499983	-10.667152
H	-13.417086	-7.856098	-0.719955
H	-14.477244	-5.629480	-0.913454

330

CAGEphen+NO3Cl SCF Done: -14397.3751597 A.U.

N	2.395304	1.830781	5.411482
N	0.722174	3.450425	5.020439
C	1.159792	2.315772	5.578545
C	3.196863	2.528365	4.598732
N	2.833720	3.661435	3.988663
C	1.592388	4.093827	4.234394
C	1.143723	5.343612	3.562289
C	1.956006	5.969083	2.606961
C	-0.112056	5.904407	3.842370
C	1.491880	7.102209	1.943124
C	-0.517249	7.044824	3.155681
N	0.275161	7.612633	2.221264
H	2.940566	5.569404	2.367306
H	-0.772272	5.450135	4.580569
H	2.079731	7.617524	1.171888
H	-1.496337	7.517561	3.308969
C	4.555085	1.989079	4.318738
C	5.386772	2.606894	3.375254
C	5.014792	0.825778	4.955145
C	6.620801	2.038640	3.067925
C	6.261342	0.310184	4.614845
N	7.029326	0.908497	3.679208
H	5.070292	3.516501	2.866346
H	4.398987	0.316027	5.695275
H	7.300785	2.469253	2.321052
H	6.663938	-0.613624	5.051336
C	0.200165	1.517637	6.389001
C	-1.149478	1.886207	6.465418
C	0.612027	0.347711	7.046175
C	-2.047407	1.073762	7.154276
C	-0.328291	-0.418702	7.726964
N	-1.628611	-0.056079	7.759098
H	-1.507193	2.790054	5.973611
H	1.652556	0.027077	7.011050

H	-3.118470	1.304239	7.227832
H	-0.077149	-1.361158	8.231871
Pd	-2.947373	-1.332761	8.678612
N	-3.317304	-4.544489	2.403159
N	-4.977638	-2.885863	2.144065
C	-4.065926	-3.539393	2.872416
C	-3.515733	-4.877944	1.123157
N	-4.405727	-4.276737	0.325724
C	-5.112364	-3.282782	0.873721
C	-6.069035	-2.545531	0.005046
C	-6.338029	-2.982742	-1.301258
C	-6.684033	-1.370361	0.456469
C	-7.510427	-0.651635	-0.404384
N	-7.736310	-1.083563	-1.661596
H	-5.882312	-3.893543	-1.688307
H	-6.504985	-1.001952	1.465985
H	-7.405719	-2.498509	-3.152240
H	-7.998769	0.286754	-0.110030
C	-2.665836	-5.948434	0.535182
C	-2.677730	-6.186037	-0.845669
C	-1.802954	-6.710616	1.337932
C	-1.817903	-7.137828	-1.388374
C	-0.976454	-7.657513	0.740687
N	-0.986671	-7.844858	-0.596630
H	-3.337412	-5.618761	-1.501220
H	-1.766261	-6.558593	2.416293
H	-1.769891	-7.345863	-2.465514
H	-0.260699	-8.267560	1.307721
C	-3.840372	-3.089735	4.272972
C	-4.669240	-2.120332	4.858999
C	-2.766691	-3.590747	5.020904
C	-4.390647	-1.673808	6.146736
C	-2.530152	-3.096837	6.302009
N	-3.332474	-2.153331	6.835121
H	-5.515905	-1.707691	4.311353
H	-2.103962	-4.347868	4.603135
H	-4.982216	-0.896991	6.649600
H	-1.692305	-3.439917	6.923113
Pd	0.372031	-9.166384	-1.387203
Pd	-0.469042	9.221263	1.183598
Pd	8.779414	-0.023548	3.142506
N	-1.155155	4.558538	-4.022752
N	-2.604257	2.834973	-4.732329
C	-1.452041	3.510799	-4.800501
C	-2.081482	4.902312	-3.120795
N	-3.264980	4.291420	-2.998270
C	-3.488099	3.264600	-3.825201
C	-4.779113	2.535154	-3.700407
C	-5.015074	1.365802	-4.435313
C	-5.773697	2.981737	-2.816357
C	-6.204043	0.663771	-4.249344
C	-6.945056	2.244065	-2.679620
N	-7.135055	1.105034	-3.379538

H	-4.271888	0.991749	-5.138515
H	-5.630304	3.888581	-2.229849
H	-6.431634	-0.267417	-4.784693
H	-7.746870	2.522249	-1.982873
C	-1.758145	5.991872	-2.160372
C	-2.612156	6.265590	-1.083675
C	-0.568970	6.728277	-2.274616
C	-2.247685	7.224603	-0.141878
C	-0.263384	7.682838	-1.309457
N	-1.089162	7.903333	-0.264027
H	-3.546985	5.719503	-0.963464
H	0.121558	6.546082	-3.097376
H	-2.868183	7.460470	0.732631
H	0.665813	8.268454	-1.322344
C	-0.422859	3.040293	-5.767356
C	-0.624559	1.869337	-6.510137
C	0.788789	3.731100	-5.924753
C	0.382743	1.406147	-7.353600
C	1.753880	3.226246	-6.790409
N	1.544001	2.080525	-7.473748
H	-1.554474	1.308887	-6.422335
H	0.984467	4.645245	-5.365343
H	0.283723	0.485012	-7.942886
H	2.732323	3.703016	-6.936397
Pd	3.082698	1.366028	-8.630672
N	5.235932	-1.734000	-2.676664
N	3.768357	-3.386630	-3.508023
C	4.423943	-2.225618	-3.619434
C	5.352097	-2.461779	-1.560732
N	4.707215	-3.614817	-1.354603
C	3.928206	-4.043611	-2.353357
C	3.160830	-5.301708	-2.146077
C	2.350355	-5.830612	-3.162570
C	3.197183	-5.961408	-0.910355
C	-7.181263	-2.229283	-2.111440
C	2.411824	-7.094149	-0.710675
N	1.627639	-7.570857	-1.698412
H	2.294697	-5.347667	-4.137564
H	3.820671	-5.587833	-0.099079
H	2.388215	-7.636730	0.243415
C	6.218549	-1.940400	-0.469119
C	6.998409	-0.788434	-0.655210
C	6.246373	-2.570982	0.781930
C	7.753732	-0.298610	0.405644
C	7.012576	-2.027390	1.810323
N	7.739870	-0.909911	1.609527
H	7.007478	-0.271352	-1.614214
H	5.662149	-3.472340	0.963129
H	8.363097	0.612120	0.334234
H	7.053438	-2.468512	2.814996
C	4.195887	-1.405765	-4.840258
C	3.426880	-1.896290	-5.906841
C	4.699224	-0.100784	-4.925157

C	3.169522	-1.071422	-6.997336
C	4.398297	0.682714	-6.036784
N	3.641195	0.193223	-7.039276
H	3.017677	-2.905611	-5.879775
H	5.304862	0.314315	-4.120408
H	2.549491	-1.383947	-7.848353
H	4.748115	1.717870	-6.142514
Pd	-8.838990	0.025795	-2.992634
C	1.595689	-6.971606	-2.908101
H	0.918711	-7.416946	-3.649482
C	6.682233	0.403227	-11.685508
C	6.760967	-0.120434	-10.410436
C	5.716640	0.111798	-9.496088
N	4.648025	0.820164	-9.829728
C	4.566917	1.383621	-11.064981
C	5.559280	1.182821	-12.049782
H	7.475440	0.231903	-12.414189
H	7.622610	-0.708658	-10.093157
H	5.753039	-0.289189	-8.485149
C	2.062621	3.520355	-12.816481
C	3.240947	2.763461	-12.608743
C	3.423793	2.196937	-11.331061
N	2.543307	2.390861	-10.314280
C	1.433863	3.082640	-10.530117
C	1.161118	3.669823	-11.781287
H	1.874327	3.971130	-13.793795
H	0.739259	3.182651	-9.698371
H	0.234228	4.229487	-11.911530
C	-2.324883	13.314661	-0.551795
C	-2.637833	12.137753	-1.202418
C	-2.093880	10.923022	-0.745243
N	-1.277803	10.877949	0.297260
C	-0.989151	12.017037	0.981857
C	-1.474941	13.280392	0.578573
H	-2.728786	14.269490	-0.890410
H	-3.305809	12.128693	-2.064281
H	-2.324131	9.980933	-1.238815
C	1.078406	12.826772	3.977683
C	0.224988	13.027697	2.866130
C	-0.165058	11.883931	2.140424
N	0.213754	10.628920	2.498089
C	1.030374	10.458437	3.527694
C	1.483835	11.546373	4.298995
H	1.413531	13.685185	4.564721
H	1.336829	9.439578	3.757022
H	2.155750	11.356710	5.136773
C	-5.560764	-4.271675	11.462406
C	-4.912239	-3.119432	11.965570
C	-4.184958	-2.334427	11.043652
N	-4.138385	-2.632276	9.717302
C	-4.731820	-3.727091	9.265385
C	-5.460873	-4.575518	10.119282
H	-6.125389	-4.909806	12.143207

H	-4.637210	-3.942908	8.202994
H	-5.932868	-5.466849	9.704740
C	-2.808450	0.402245	13.169903
C	-2.114030	1.089945	12.194500
C	-2.101726	0.601626	10.873092
N	-2.737200	-0.511819	10.537940
C	-3.460526	-1.181393	11.473536
C	-3.512640	-0.775907	12.822465
H	-2.827750	0.759577	14.202248
H	-1.576705	2.011175	12.422383
H	-1.564485	1.125237	10.084748
C	-11.835891	1.965199	-6.225686
C	-12.256853	1.023852	-5.255554
C	-11.280191	0.544644	-4.359319
N	-9.994400	0.983809	-4.378418
C	-9.609699	1.847966	-5.306452
C	-10.514961	2.366761	-6.252961
H	-12.556533	2.358021	-6.946912
H	-8.562724	2.144914	-5.307020
H	-10.151985	3.075422	-6.998182
C	-13.213994	-1.844887	-2.235568
C	-12.183533	-2.262573	-1.416983
C	-10.884043	-1.762995	-1.622503
N	-10.618780	-0.899952	-2.592020
C	-11.624231	-0.440003	-3.384056
C	-12.952098	-0.904989	-3.259933
H	-14.228281	-2.222300	-2.100380
H	-12.357351	-2.970906	-0.606335
H	-10.053699	-2.075147	-0.992208
C	11.826785	2.023667	6.262632
C	10.527802	2.490547	6.228986
C	9.597759	1.900472	5.353065
N	9.935615	0.896641	4.557306
C	11.219362	0.447989	4.539815
C	12.210006	0.968964	5.401205
H	12.564271	2.460233	6.937182
H	10.206972	3.315303	6.866125
H	8.567814	2.248597	5.305930
C	13.052060	-2.189820	2.645637
C	12.825506	-1.140224	3.568375
C	11.530874	-0.584014	3.603389
N	10.539086	-0.991449	2.768682
C	10.759367	-1.990028	1.925863
C	12.018271	-2.615656	1.835214
H	14.038072	-2.657132	2.589057
H	9.926438	-2.307718	1.301604
H	12.151961	-3.433970	1.126832
C	-2.229092	-13.219119	-1.300816
C	-2.857494	-12.045126	-0.935039
C	-2.127152	-10.840741	-0.917111
N	-0.841815	-10.802906	-1.237444
C	-0.214620	-11.939415	-1.638980
C	-0.862320	-13.191001	-1.668759

H	-2.774489	-14.165731	-1.317115
H	-3.913723	-12.026871	-0.664159
H	-2.598141	-9.900943	-0.635491
C	3.252334	-12.780647	-2.749193
C	1.891708	-12.973400	-2.413464
C	1.151644	-11.829961	-2.039649
N	1.694899	-10.583220	-2.041569
C	2.976417	-10.421887	-2.336150
C	3.790723	-11.510087	-2.700699
H	3.861294	-13.639222	-3.034713
H	3.374745	-9.410240	-2.289474
H	4.840777	-11.331872	-2.934600
O	-2.134987	-4.747454	8.899655
O	-4.069437	1.937688	9.351618
O	10.896920	2.178463	2.076192
O	9.076841	-3.496523	3.624239
O	5.343683	3.612854	-9.163972
O	0.107544	0.597083	-10.343833
O	1.634122	-11.121422	0.862204
O	-2.534931	-9.451856	-3.349366
O	-3.957634	9.602373	0.890747
O	1.946790	11.069252	0.122464
O	-9.656473	-1.683169	-5.611334
O	-11.048909	1.755386	-1.380758
N	-8.481302	-1.303970	-5.733430
O	-7.553577	-1.899184	-5.102638
O	-8.189241	-0.312557	-6.433618
N	-10.189176	1.358757	-0.579097
O	-9.060101	1.936881	-0.520879
O	-10.391585	0.365028	0.149611
N	1.006577	-0.257032	-10.200837
O	1.026693	-0.948696	-9.135838
O	1.895415	-0.406461	-11.053863
N	5.515362	3.137430	-8.030858
O	4.767926	3.499128	-7.070257
O	6.386268	2.268076	-7.817859
N	-3.409378	9.520076	2.009969
O	-3.050466	10.534632	2.627185
O	-3.179820	8.368336	2.492606
N	2.416053	9.972028	0.463631
O	2.289040	8.963498	-0.297918
O	2.976811	9.821460	1.568274
N	9.763048	2.682216	2.082263
O	9.393941	3.436640	3.006297
O	8.936011	2.396151	1.162129
N	8.788869	-2.737947	4.572814
O	7.571895	-2.422313	4.753018
O	9.668651	-2.258179	5.304684
N	-1.363774	-3.872991	9.346754
O	-1.431344	-3.497382	10.527434
O	-0.530456	-3.332929	8.555314
N	-4.861356	1.047373	8.979185
O	-5.471745	0.343627	9.798873

O	-5.000287	0.836874	7.734406
N	-1.408721	-9.352346	-3.878323
O	-0.742917	-10.357640	-4.171767
O	-0.926920	-8.193311	-4.072656
N	2.206333	-10.021888	0.916426
O	1.631783	-9.033601	1.469520
O	3.327186	-9.848151	0.394406
C	-13.933540	-0.380346	-4.181727
C	-13.599904	0.535017	-5.142123
C	-1.071315	14.431032	1.354110
C	-0.250961	14.312073	2.442382
C	-4.268948	-1.561696	13.753077
C	-4.930165	-2.687463	13.344435
C	4.238514	2.540787	-13.614130
C	5.351407	1.792710	-13.343220
C	-0.115620	-14.347625	-2.069268
C	1.203569	-14.244200	-2.416319
C	13.531742	0.389333	5.328438
C	13.824368	-0.625401	4.458828
H	-0.609316	-15.320078	-2.091546
H	4.105436	2.978939	-14.604188
H	0.044180	15.201396	3.000856
H	14.829385	-1.048823	4.435460
H	-4.310970	-1.255797	14.799289
H	-14.361855	0.903786	-5.830131
Cl	-5.826440	-3.625581	14.510577
Cl	14.779092	0.998893	6.384900
Cl	2.081819	-15.677634	-2.882460
Cl	-15.582208	-0.938996	-4.066221
Cl	-1.642408	16.009818	0.880154
Cl	6.550181	1.558060	-14.588703

330

CAGEphen+NO3Co3 SCF Done: -19169.1685340 A.U.

N	-3.878965	3.265898	-3.310190
N	-3.658037	4.590270	-1.368614
C	-3.326101	4.274538	-2.626392
C	-4.851029	2.592877	-2.683910
N	-5.284105	2.881257	-1.451107
C	-4.636360	3.864415	-0.816889
C	-4.991973	4.127715	0.602340
C	-6.123244	3.541244	1.187437
C	-4.172031	4.927394	1.408995
C	-6.383202	3.757738	2.537133
C	-4.482056	5.075343	2.757335
N	-5.562729	4.496574	3.312547
H	-6.788917	2.912660	0.596554
H	-3.289069	5.414131	0.996130
H	-7.234920	3.316764	3.061810
H	-3.841257	5.674154	3.402861
C	-5.453596	1.421081	-3.370674
C	-6.598623	0.794127	-2.858517
C	-4.858325	0.874924	-4.515235
C	-7.088064	-0.348414	-3.483076

C	-5.390370	-0.286303	-5.067938
N	-6.477836	-0.891733	-4.556621
H	-7.095409	1.186909	-1.971849
H	-3.975040	1.334046	-4.958150
H	-7.960733	-0.900435	-3.124818
H	-4.922654	-0.742431	-5.939241
C	-2.268466	5.081253	-3.290933
C	-1.658418	6.152298	-2.620315
C	-1.845840	4.791093	-4.596502
C	-0.647227	6.865810	-3.255554
C	-0.829429	5.552076	-5.170181
N	-0.234704	6.559216	-4.500863
H	-1.966548	6.419510	-1.609890
H	-2.300237	3.975912	-5.158965
H	-0.125360	7.706400	-2.788947
H	-0.461299	5.372739	-6.184601
Co	1.331139	7.493932	-5.284264
N	4.782938	1.691076	-3.314828
N	5.150382	3.115031	-1.466794
C	4.685418	2.874338	-2.698150
C	5.379932	0.713775	-2.622510
N	5.818242	0.853524	-1.365315
C	5.677621	2.068013	-0.823440
C	6.081233	2.257290	0.594796
C	6.363128	1.155309	1.412767
C	6.135070	3.535844	1.168271
C	6.447486	3.666002	2.518043
N	6.676039	2.594064	3.304147
H	6.345531	0.143168	1.009907
H	5.923049	4.420689	0.568770
H	6.836214	0.519760	3.418955
H	6.485072	4.628126	3.035234
C	5.549067	-0.611050	-3.276178
C	6.171921	-1.669539	-2.597547
C	5.084523	-0.842492	-4.579216
C	6.283466	-2.907145	-3.222975
C	5.233631	-2.107958	-5.142981
N	5.809601	-3.121042	-4.465673
H	6.558641	-1.527854	-1.588731
H	4.605605	-0.045509	-5.147459
H	6.751279	-3.775961	-2.750234
H	4.891951	-2.344442	-6.155328
C	3.972509	3.977400	-3.392995
C	4.000946	5.284809	-2.887133
C	3.202578	3.729078	-4.536869
C	3.256104	6.276231	-3.516844
C	2.462513	4.767515	-5.095349
N	2.481523	6.014351	-4.589981
H	4.588044	5.523004	-2.000672
H	3.158620	2.732153	-4.974354
H	3.210963	7.309283	-3.162567
H	1.833359	4.584201	-5.965237
Co	5.839685	-4.949806	-5.236455

Co	-5.976908	4.619799	5.262667
Co	-7.183204	-2.630329	-5.246137
N	-1.254249	-0.480320	5.873397
N	1.084796	-0.796994	5.869539
C	-0.169424	-1.263143	5.871109
C	-1.030842	0.838979	5.864902
N	0.189525	1.387290	5.861883
C	1.220302	0.533935	5.860342
C	2.595575	1.097718	5.810837
C	3.719868	0.260108	5.775458
C	2.802995	2.484518	5.764261
C	4.989792	0.822710	5.670825
C	4.100306	2.975244	5.654510
N	5.168421	2.156468	5.594341
H	3.606009	-0.822609	5.817494
H	1.957911	3.171421	5.797856
H	5.899676	0.215303	5.640729
H	4.333607	4.042613	5.600244
C	-2.208538	1.746044	5.820674
C	-2.049468	3.138719	5.775783
C	-3.512222	1.228893	5.788977
C	-3.175118	3.952967	5.674143
C	-4.589352	2.103220	5.682844
N	-4.418578	3.437455	5.610284
H	-1.055874	3.584628	5.806704
H	-3.681449	0.153400	5.831518
H	-3.107139	5.044035	5.634078
H	-5.629451	1.766620	5.642838
C	-0.367765	-2.736544	5.833979
C	0.730906	-3.608526	5.800334
C	-1.654706	-3.292728	5.798585
C	0.509523	-4.978662	5.703050
C	-1.799887	-4.674774	5.705708
N	-0.732867	-5.495702	5.640962
H	1.747544	-3.218232	5.835158
H	-2.536255	-2.653350	5.831448
H	1.317026	-5.715341	5.660161
H	-2.777595	-5.164450	5.675506
Co	-0.987624	-7.438698	5.318577
N	-2.161773	-5.475823	-1.326095
N	0.130808	-6.030505	-1.413131
C	-1.042023	-5.956243	-0.774987
C	-2.058724	-5.039617	-2.587534
N	-0.911530	-5.020061	-3.276076
C	0.159484	-5.521458	-2.650302
C	1.472966	-5.463261	-3.342447
C	2.591311	-6.133073	-2.825570
C	1.643438	-4.688737	-4.497409
C	6.640709	1.363886	2.760117
C	2.913328	-4.573685	-5.055536
N	3.983907	-5.204296	-4.539470
H	2.503423	-6.748852	-1.930948
H	0.802018	-4.160039	-4.944284

H	3.071894	-3.951342	-5.934753
C	-3.287942	-4.529995	-3.251011
C	-4.518415	-4.532799	-2.576863
C	-3.251854	-4.025679	-4.559228
C	-5.643983	-4.017651	-3.211709
C	-4.420550	-3.528573	-5.132255
N	-5.588693	-3.514049	-4.460259
H	-4.592794	-4.926798	-1.563695
H	-2.320410	-4.015099	-5.124679
H	-6.629884	-3.983305	-2.738864
H	-4.450348	-3.126732	-6.149307
C	-1.086217	-6.385829	0.647598
C	-0.008016	-7.064491	1.233705
C	-2.186140	-6.072629	1.457015
C	-0.057991	-7.385446	2.586750
C	-2.151421	-6.403597	2.808269
N	-1.104903	-7.040664	3.364251
H	0.867646	-7.328970	0.641625
H	-3.052586	-5.556766	1.044443
H	0.754022	-7.893196	3.113979
H	-2.985537	-6.144003	3.458351
Co	6.975853	2.905692	5.255276
C	3.823240	-5.991047	-3.455729
H	4.740038	-6.464264	-3.094361
C	-1.603073	-12.156322	5.143792
C	-1.844446	-11.432735	3.990675
C	-1.678725	-10.034109	3.998759
N	-1.293684	-9.374483	5.080224
C	-1.093490	-10.067052	6.228674
C	-1.218251	-11.471615	6.319262
H	-1.712106	-13.243566	5.159167
H	-2.159933	-11.924597	3.069389
H	-1.871129	-9.444735	3.103947
C	-0.125919	-9.117881	9.704890
C	-0.482832	-9.943531	8.615142
C	-0.744884	-9.299499	7.383899
N	-0.680454	-7.954638	7.218325
C	-0.342886	-7.202810	8.254643
C	-0.056851	-7.748601	9.520864
H	0.094665	-9.566269	10.676742
H	-0.292485	-6.126945	8.085370
H	0.219960	-7.081350	10.338635
C	-7.900542	4.753615	9.631372
C	-6.741978	4.015931	9.466395
C	-6.114838	3.980578	8.206041
N	-6.592829	4.633087	7.157737
C	-7.732464	5.353922	7.304590
C	-8.432830	5.458518	8.528500
H	-8.407882	4.794611	10.598404
H	-6.305401	3.455947	10.294907
H	-5.202025	3.404768	8.051580
C	-9.773035	7.483538	5.020594
C	-9.378450	6.824508	6.207446

C	-8.217557	6.022071	6.136936
N	-7.505967	5.840788	4.996964
C	-7.878926	6.489534	3.904734
C	-9.013239	7.323970	3.876600
H	-10.664917	8.115024	5.020373
H	-7.262490	6.356674	3.017483
H	-9.276329	7.830915	2.947072
C	4.695162	10.128019	-7.384396
C	3.364111	10.572155	-7.211741
C	2.467922	9.676589	-6.586121
N	2.834735	8.453328	-6.130434
C	4.080422	8.049447	-6.325081
C	5.045079	8.862710	-6.950829
H	5.428845	10.782340	-7.861692
H	4.333421	7.047655	-5.982605
H	6.058060	8.479468	-7.082035
C	-0.729806	11.599082	-6.592951
C	-1.532493	10.644014	-5.995260
C	-0.978100	9.403025	-5.626841
N	0.296352	9.107547	-5.830528
C	1.095798	10.038387	-6.408118
C	0.634251	11.309513	-6.821217
H	-1.135417	12.570571	-6.886098
H	-2.589034	10.834739	-5.800458
H	-1.591262	8.634493	-5.155524
C	8.030303	4.507398	9.629082
C	8.916387	4.606679	8.532864
C	8.480751	4.054129	7.306383
N	7.282581	3.437349	7.151108
C	6.469949	3.357655	8.193472
C	6.808418	3.882538	9.455616
H	8.315363	4.925583	10.597626
H	5.512529	2.861649	8.032973
H	6.097977	3.791292	10.278749
C	11.369116	4.728512	5.044003
C	10.854553	4.154068	3.896446
C	9.560154	3.599147	3.915641
N	8.803781	3.607833	5.002238
C	9.311876	4.131315	6.145188
C	10.591903	4.724337	6.224984
H	12.365762	5.176708	5.050892
H	11.431883	4.122673	2.971277
H	9.139638	3.133662	3.025955
C	-11.143817	-1.045225	-7.357986
C	-10.228422	-0.106920	-6.918569
C	-9.043065	-0.532632	-6.288324
N	-8.766376	-1.812628	-6.094331
C	-9.636970	-2.744315	-6.555310
C	-10.858659	-2.419287	-7.186546
H	-12.076180	-0.739595	-7.839164
H	-10.406196	0.961553	-7.048828
H	-8.305389	0.189093	-5.942142
C	-9.691217	-6.475178	-6.565604

C	-10.125481	-5.150205	-6.794923
C	-9.260654	-4.112553	-6.376823
N	-8.057382	-4.336367	-5.792465
C	-7.673251	-5.586727	-5.587611
C	-8.465263	-6.689539	-5.961619
H	-10.325578	-7.313899	-6.862888
H	-6.703458	-5.730936	-5.110745
H	-8.100116	-7.698935	-5.765675
C	10.419850	-5.205898	-6.566474
C	9.993210	-4.032753	-5.970448
C	8.643236	-3.896733	-5.593162
N	7.752625	-4.856752	-5.788165
C	8.159810	-6.014892	-6.364607
C	9.489856	-6.247220	-6.784478
H	11.462593	-5.337330	-6.865896
H	10.684561	-3.209693	-5.782834
H	8.284251	-2.981859	-5.121051
C	6.446221	-9.189812	-7.313564
C	7.492766	-8.252905	-7.153501
C	7.163669	-7.027339	-6.531976
N	5.922553	-6.737039	-6.069622
C	4.953232	-7.620066	-6.252049
C	5.176985	-8.864514	-6.872381
H	6.647604	-10.153878	-7.787129
H	3.960159	-7.341333	-5.903707
H	4.341285	-9.555273	-6.993508
O	2.696608	6.011798	-8.168765
O	1.205348	10.729689	-3.468552
O	-9.922875	-4.351696	-3.355004
O	-6.311553	-2.677279	-7.237064
O	-3.090208	-7.307077	5.907700
O	2.173808	-8.724298	6.766865
O	5.433930	-4.180198	-7.237980
O	8.719854	-6.407407	-3.382474
O	-4.819417	6.406943	5.813112
O	-8.700797	2.523125	6.693800
O	9.427772	0.561326	4.318163
O	6.511018	6.316867	6.662932
N	8.425761	0.228178	4.950726
O	7.835792	-0.849227	4.747110
O	7.930043	1.027309	5.827122
N	6.774044	5.932280	5.524640
O	6.082202	4.951775	5.027820
O	7.678289	6.410543	4.835783
N	1.718040	-8.774622	5.625551
O	1.227979	-7.688990	5.107231
O	1.675754	-9.808401	4.954368
N	-4.026370	-7.354395	5.027810
O	-4.663466	-6.308340	4.802868
O	-4.235000	-8.399995	4.413062
N	-4.374871	7.222828	4.924206
O	-5.163939	7.917358	4.284008
O	-3.147154	7.245789	4.719309

N	-8.494694	2.924208	5.549542
O	-7.296471	2.810910	5.060523
O	-9.360816	3.448687	4.845400
N	-9.560990	-3.176994	-3.385971
O	-10.337937	-2.228131	-3.520579
O	-8.291550	-2.917439	-3.297563
N	-5.833734	-1.619039	-7.790894
O	-4.598105	-1.535503	-7.914554
O	-6.594146	-0.715099	-8.136369
N	1.532724	5.808249	-7.824102
O	0.853074	6.757007	-7.283345
O	0.984977	4.697101	-7.945815
N	2.041194	9.827912	-3.475420
O	3.252168	10.022353	-3.608545
O	1.630178	8.601330	-3.358140
N	7.526433	-6.703279	-3.403260
O	7.113078	-7.857698	-3.536173
O	6.649977	-5.749827	-3.299554
N	4.276157	-4.303586	-7.784612
O	3.580007	-3.279020	-7.906957
O	3.877334	-5.417066	-8.124331
C	11.016538	5.277302	7.481936
C	10.211898	5.226492	8.586324
C	-9.624579	6.260515	8.572550
C	-10.073899	6.919280	7.461815
C	1.567465	12.213766	-7.435194
C	2.873608	11.857822	-7.627285
C	-0.592098	-11.375070	8.679496
C	-0.950123	-12.106048	7.580833
C	9.807998	-7.508787	-7.394976
C	8.849710	-8.467061	-7.576553
C	-11.721283	-3.489330	-7.607384
C	-11.373184	-4.797550	-7.414916
H	10.837444	-7.687377	-7.714266
H	9.103036	-9.420551	-8.046074
H	-1.034621	-13.193544	7.643675
H	-0.383576	-11.870556	9.630592
H	-10.166983	6.334931	9.517974
H	-10.978496	7.530168	7.509286
H	-12.041702	-5.601108	-7.732679
H	-12.670567	-3.234095	-8.084332
H	1.209502	13.197170	-7.748852
H	3.572303	12.552269	-8.099882
H	10.543720	5.657496	9.533790
H	12.001603	5.746769	7.536537

330

CAGEphen+NO3Co5 SCF Done: -19169.1729655 A.U.

N	-4.671251	-2.062098	-3.268530
N	-5.728364	-1.356319	-1.279622
C	-5.331087	-1.153806	-2.541513
C	-4.477053	-3.252380	-2.688412
N	-4.910387	-3.564116	-1.461347
C	-5.493615	-2.573841	-0.777142

C	-5.856713	-2.832542	0.640585
C	-5.886087	-4.134759	1.155169
C	-6.124612	-1.773983	1.516479
C	-6.162709	-4.329423	2.504754
C	-6.361150	-2.042245	2.861920
N	-6.374691	-3.297876	3.350653
H	-5.683953	-4.990821	0.511939
H	-6.128953	-0.744867	1.158112
H	-6.193750	-5.323939	2.953933
H	-6.529335	-1.203912	3.542363
C	-3.689644	-4.274031	-3.424331
C	-3.658020	-5.608658	-3.001772
C	-2.906745	-3.914927	-4.527936
C	-2.842686	-6.517052	-3.670172
C	-2.089419	-4.869409	-5.125525
N	-2.048517	-6.147515	-4.698045
H	-4.251151	-5.935455	-2.148028
H	-2.912772	-2.892425	-4.904503
H	-2.780139	-7.565885	-3.374469
H	-1.457822	-4.565261	-5.963308
C	-5.631127	0.162894	-3.164554
C	-6.309499	1.157117	-2.444641
C	-5.253412	0.444704	-4.486288
C	-6.561096	2.383456	-3.051469
C	-5.542758	1.695815	-5.026567
N	-6.170911	2.649369	-4.312823
H	-6.634265	0.974890	-1.420510
H	-4.739667	-0.304741	-5.087991
H	-7.085109	3.202707	-2.553230
H	-5.287641	1.964870	-6.054034
Co	-6.406013	4.487725	-5.042888
N	0.339077	5.130656	-3.195646
N	-0.751285	6.037120	-1.305763
C	-0.758604	5.540377	-2.548503
C	1.490338	5.230003	-2.522192
N	1.575823	5.641952	-1.251753
C	0.431008	6.032675	-0.680603
C	0.465382	6.442408	0.747378
C	1.574133	6.149272	1.549418
C	-0.625031	7.088031	1.344558
C	-0.575236	7.401766	2.699104
N	0.481489	7.073326	3.474210
H	2.452734	5.662701	1.126750
H	-1.510641	7.336185	0.760324
H	2.396728	6.195746	3.528506
H	-1.400845	7.901702	3.208354
C	2.748811	4.849026	-3.217282
C	3.983208	4.926235	-2.555237
C	2.741426	4.411236	-4.549565
C	5.141155	4.551909	-3.228047
C	3.943576	4.051658	-5.156404
N	5.116982	4.109140	-4.501397
H	4.037399	5.273918	-1.524000

H	1.807917	4.350409	-5.108753
H	6.133820	4.594158	-2.773532
H	3.994744	3.709033	-6.191146
C	-2.071084	5.383839	-3.225276
C	-3.220795	6.019200	-2.737867
C	-2.202233	4.545551	-4.339024
C	-4.445324	5.782994	-3.356422
C	-3.463906	4.329913	-4.882998
N	-4.566627	4.931233	-4.396362
H	-3.165722	6.683835	-1.876194
H	-1.333358	4.043342	-4.763664
H	-5.371236	6.251116	-3.014368
H	-3.574262	3.647876	-5.727441
Co	6.800072	3.416791	-5.321078
Co	-6.538282	-3.720481	5.297388
Co	-0.744594	-7.488080	-5.401121
N	0.089981	-1.533751	5.784514
N	1.336777	0.471150	5.813656
C	1.247899	-0.863692	5.778394
C	-1.021525	-0.789688	5.823294
N	-1.021433	0.547427	5.864644
C	0.178641	1.138906	5.853220
C	0.226177	2.625032	5.851844
C	1.448289	3.311699	5.852921
C	-0.954031	3.382793	5.821801
C	1.450725	4.703821	5.794200
C	-0.871523	4.770349	5.766907
N	0.311716	5.416284	5.735441
H	2.391359	2.766996	5.887775
H	-1.927281	2.893159	5.824835
H	2.376170	5.281614	5.792851
H	-1.750613	5.418472	5.737083
C	-2.333246	-1.489086	5.788274
C	-3.536922	-0.773284	5.864630
C	-2.403070	-2.883333	5.650870
C	-4.745797	-1.459323	5.777670
C	-3.648576	-3.498108	5.567571
N	-4.796782	-2.793345	5.615643
H	-3.532609	0.309750	5.981010
H	-1.492897	-3.480146	5.594517
H	-5.709956	-0.951287	5.835749
H	-3.781691	-4.577791	5.454609
C	2.510230	-1.645031	5.696703
C	3.751912	-0.997616	5.611271
C	2.497514	-3.046647	5.675363
C	4.909159	-1.757509	5.479035
C	3.699486	-3.739313	5.540495
N	4.878514	-3.104916	5.425829
H	3.812636	0.090036	5.632124
H	1.559202	-3.595097	5.752177
H	5.905630	-1.315136	5.404735
H	3.736782	-4.829252	5.516736
Co	6.564489	-4.095779	5.019917

N	4.023550	-4.158969	-1.534741
N	5.522996	-2.338695	-1.643175
C	4.968325	-3.374584	-1.004229
C	3.632377	-3.852969	-2.777169
N	4.080634	-2.792642	-3.458110
C	5.021056	-2.058444	-2.851704
C	5.504102	-0.830548	-3.533298
C	6.643771	-0.152991	-3.083578
C	4.796377	-0.281915	-4.609147
C	1.539375	6.461430	2.905174
C	5.211351	0.929440	-5.155325
N	6.298525	1.586115	-4.701769
H	7.228399	-0.542358	-2.250546
H	3.910931	-0.779846	-5.003408
H	4.621128	1.360525	-5.968010
C	2.631789	-4.739259	-3.429315
C	2.109760	-5.848802	-2.748833
C	2.189915	-4.498917	-4.739113
C	1.165599	-6.651558	-3.380590
C	1.243462	-5.349350	-5.306339
N	0.732499	-6.395484	-4.630146
H	2.435030	-6.078779	-1.734532
H	2.580959	-3.657556	-5.310637
H	0.718997	-7.532716	-2.913935
H	0.872136	-5.219216	-6.324922
C	5.394485	-3.640123	0.394462
C	6.528226	-3.024161	0.939357
C	4.642622	-4.479578	1.224430
C	6.856440	-3.252767	2.272245
C	5.012397	-4.635831	2.557136
N	6.098279	-4.028047	3.077075
H	7.147319	-2.364627	0.331931
H	3.761431	-4.993991	0.841853
H	7.729738	-2.794008	2.740303
H	4.389840	-5.263546	3.199220
Co	0.331601	7.393001	5.442921
C	7.017856	1.037868	-3.697951
H	7.894032	1.604005	-3.375639
C	10.826631	-6.196142	4.612109
C	9.988071	-6.226400	3.513175
C	8.707076	-5.645961	3.596200
N	8.270084	-5.060522	4.700305
C	9.062398	-5.069368	5.800418
C	10.366691	-5.611438	5.814441
H	11.830511	-6.625525	4.565929
H	10.300941	-6.688151	2.575511
H	8.023696	-5.670515	2.749902
C	8.645819	-3.873550	9.309222
C	9.265745	-4.452539	8.180179
C	8.506260	-4.499794	6.988246
N	7.237312	-4.032077	6.893287
C	6.676233	-3.496142	7.968200
C	7.349706	-3.399371	9.200612

H	9.191969	-3.803918	10.253201
H	5.654851	-3.130803	7.856689
H	6.840479	-2.945980	10.052595
C	-7.121487	-5.750803	9.578736
C	-6.058079	-4.878382	9.422994
C	-5.880285	-4.212778	8.195377
N	-6.700206	-4.386752	7.168148
C	-7.745208	-5.238897	7.309723
C	-8.012736	-5.951291	8.501689
H	-7.277469	-6.283891	10.519860
H	-5.350429	-4.699148	10.234087
H	-5.049443	-3.522302	8.047216
C	-10.554865	-6.281368	5.090643
C	-9.734137	-6.225793	6.240481
C	-8.601205	-5.384994	6.174061
N	-8.264182	-4.672566	5.070715
C	-9.067244	-4.711174	4.018421
C	-10.226734	-5.510943	3.990463
H	-11.441349	-6.920425	5.086113
H	-8.799655	-4.080947	3.173150
H	-10.848581	-5.511390	3.094000
C	-7.611233	8.776126	-6.732549
C	-8.538289	7.719966	-6.578706
C	-8.028178	6.488033	-6.113485
N	-6.726283	6.295037	-5.787601
C	-5.869278	7.287384	-5.973288
C	-6.277540	8.549909	-6.445796
H	-7.953455	9.752520	-7.084450
H	-4.823574	7.079975	-5.756452
H	-5.530669	9.334901	-6.573268
C	-11.054029	4.306807	-6.108662
C	-10.451112	3.138658	-5.674459
C	-9.070287	3.130858	-5.400148
N	-8.312217	4.209136	-5.544163
C	-8.892306	5.358177	-5.969125
C	-10.268483	5.468687	-6.273940
H	-12.125183	4.339877	-6.322749
H	-11.027230	2.222251	-5.536587
H	-8.572051	2.223835	-5.056846
C	-0.862008	8.830375	9.835183
C	-0.673829	9.721982	8.756317
C	-0.262765	9.158646	7.525842
N	-0.039209	7.832785	7.349694
C	-0.218830	7.017285	8.379487
C	-0.630748	7.479038	9.643859
H	-1.188566	9.212454	10.805523
H	-0.031019	5.957681	8.203833
H	-0.766433	6.761988	10.455154
C	0.040776	12.152677	5.323388
C	0.474189	11.507232	4.180100
C	0.606656	10.104548	4.177727
N	0.324187	9.368717	5.241649
C	-0.053336	9.993809	6.384336

C	-0.229582	11.391788	6.483809
H	-0.084146	13.238149	5.344000
H	0.713442	12.063535	3.272584
H	0.964401	9.576697	3.296234
C	-3.857098	-10.617522	-7.201772
C	-4.329575	-9.361758	-6.867550
C	-3.440738	-8.394125	-6.359824
N	-2.152537	-8.644628	-6.184715
C	-1.667325	-9.855183	-6.555821
C	-2.478507	-10.896121	-7.058834
H	-4.530748	-11.389468	-7.582038
H	-5.383186	-9.104080	-6.984861
H	-3.785775	-7.394441	-6.104025
C	1.736310	-11.380770	-6.612313
C	0.336803	-11.277206	-6.770910
C	-0.256243	-10.042240	-6.421049
N	0.448872	-8.979599	-5.961552
C	1.762404	-9.099775	-5.826917
C	2.446550	-10.288755	-6.143653
H	2.243632	-12.316447	-6.860067
H	2.298888	-8.225281	-5.457858
H	3.528789	-10.332539	-6.011073
C	8.912388	7.570419	-6.350253
C	7.614648	7.613205	-5.870289
C	6.932642	6.410384	-5.605512
N	7.487523	5.222313	-5.800956
C	8.758459	5.168609	-6.269298
C	9.525880	6.317503	-6.570240
H	9.464555	8.490138	-6.558771
H	7.109838	8.563440	-5.688801
H	5.909823	6.416591	-5.227719
C	11.066632	2.385278	-7.181288
C	10.613063	3.708971	-6.978930
C	9.307688	3.862734	-6.462186
N	8.508732	2.818506	-6.131621
C	8.940356	1.588752	-6.362967
C	10.221894	1.330785	-6.887937
H	12.071250	2.209307	-7.573828
H	8.251139	0.776997	-6.140439
H	10.529634	0.296959	-7.051568
O	-4.530641	5.357204	-7.738187
O	-9.248578	5.998189	-3.113299
O	-0.599371	-10.783106	-3.575852
O	-0.463436	-6.642340	-7.341594
O	5.638300	-5.966168	5.483491
O	9.261862	-1.736781	6.195880
O	5.883709	3.338556	-7.259911
O	9.579642	5.124029	-3.405344
O	-7.637215	-1.975428	5.895553
O	-5.832223	-7.281799	6.317276
O	3.252452	8.764062	4.369610
O	-3.042523	8.497135	6.715330
N	3.302184	7.706923	5.000843

O	4.146702	6.830866	4.766503
O	2.413126	7.457846	5.900278
N	-2.556164	8.433984	5.587259
O	-1.915940	7.364087	5.242148
O	-2.627587	9.357125	4.767101
N	8.908067	-2.105555	5.076835
O	7.644379	-2.133201	4.799195
O	9.701655	-2.480593	4.205852
N	5.335003	-6.837630	4.585041
O	4.138269	-7.120185	4.434271
O	6.224359	-7.335674	3.891601
N	-8.284372	-1.257553	5.043744
O	-9.194226	-1.765787	4.384056
O	-7.939937	-0.076693	4.900921
N	-6.026429	-6.742469	5.229098
O	-5.411804	-5.636584	4.961653
O	-6.810295	-7.191104	4.382912
N	-1.371223	-9.827878	-3.510599
O	-2.602757	-9.953894	-3.531826
O	-0.883820	-8.632014	-3.451830
N	-1.301946	-5.816783	-7.868473
O	-0.911454	-4.666365	-8.107609
O	-2.460065	-6.180738	-8.084355
N	-4.792919	4.164492	-7.559661
O	-5.903438	3.835871	-6.994722
O	-4.004346	3.257895	-7.859616
N	-8.032059	6.177074	-3.076896
O	-7.516131	7.302610	-3.096062
O	-7.249047	5.149313	-3.053787
N	9.158142	3.968710	-3.413726
O	9.898497	2.980470	-3.499715
O	7.882144	3.767889	-3.361146
N	5.595684	2.229484	-7.848026
O	4.402422	1.986383	-8.074016
O	6.495473	1.436386	-8.133407
C	-0.659908	11.942756	7.739911
C	-0.875879	11.142905	8.827621
C	-9.154351	-6.823519	8.538429
C	-9.980312	-6.952335	7.456399
C	-10.772603	6.738817	-6.719661
C	-9.943551	7.815838	-6.867866
C	10.601205	-4.983229	8.164769
C	11.127082	-5.540889	7.032438
C	10.862150	6.138942	-7.068435
C	11.381201	4.889688	-7.267087
C	-1.858267	-12.149140	-7.394076
C	-0.510723	-12.332952	-7.253460
H	11.457973	7.027662	-7.289261
H	12.396872	4.767745	-7.650881
H	12.142131	-5.945060	7.035257
H	11.191344	-4.933651	9.082828
H	-9.352026	-7.383140	9.455663
H	-10.847911	-7.615044	7.498891

H	-0.052867	-13.291019	-7.510473
H	-2.490604	-12.958360	-7.766959
H	-11.838706	6.826149	-6.941930
H	-10.338806	8.774649	-7.211659
H	-1.205782	11.574513	9.775489
H	-0.812428	13.022426	7.808685

330

CAGEphen+NO3Cu3 SCF Done: -20715.0786138 A.U.

N	2.110345	-3.279386	-4.741017
N	2.165955	-4.833876	-2.963808
C	1.576909	-4.280226	-4.030295
C	3.298759	-2.829687	-4.323515
N	3.957604	-3.331816	-3.273500
C	3.356681	-4.331531	-2.620182
C	4.046691	-4.900271	-1.430759
C	5.298833	-4.412561	-1.029411
C	3.452828	-5.916807	-0.668792
C	5.907720	-4.945743	0.103890
C	4.116416	-6.396350	0.458966
N	5.319533	-5.917781	0.824656
H	5.789869	-3.618155	-1.590244
H	2.478793	-6.320994	-0.942960
H	6.875127	-4.594582	0.475329
H	3.674198	-7.169921	1.100189
C	3.917228	-1.684265	-5.044549
C	5.128065	-1.135674	-4.595864
C	3.296550	-1.106112	-6.161146
C	5.650264	-0.022368	-5.246710
C	3.884128	0.003648	-6.767891
N	5.026903	0.535927	-6.299505
H	5.644034	-1.562608	-3.736698
H	2.358919	-1.507101	-6.545273
H	6.576654	0.471056	-4.936597
H	3.441069	0.502158	-7.635748
C	0.240792	-4.795458	-4.434743
C	-0.385156	-5.814632	-3.702138
C	-0.435013	-4.258961	-5.540271
C	-1.650961	-6.250093	-4.087710
C	-1.695256	-4.751356	-5.873184
N	-2.280546	-5.727765	-5.155758
H	0.102861	-6.255006	-2.833317
H	0.013404	-3.460266	-6.130428
H	-2.188433	-7.022077	-3.522360
H	-2.278151	-4.369833	-6.717196
Cu	-4.232234	-6.215429	-5.628963
N	-5.685826	-0.462396	-1.788756
N	-5.588783	-2.092031	-0.083274
C	-5.540114	-1.724098	-1.368719
C	-5.882462	0.457448	-0.837899
N	-5.933586	0.176599	0.468651
C	-5.782735	-1.109411	0.803580
C	-5.817258	-1.460900	2.249131
C	-5.972471	-0.463962	3.223363

C	-5.673432	-2.789834	2.673351
C	-5.688340	-3.074697	4.037164
N	-5.833515	-2.103247	4.956835
H	-6.083064	0.581189	2.936479
H	-5.543609	-3.592559	1.948024
H	-6.051411	-0.068810	5.363404
H	-5.571975	-4.087602	4.435239
C	-6.040610	1.876926	-1.254514
C	-6.179039	2.893079	-0.297753
C	-6.036720	2.234356	-2.610588
C	-6.296048	4.214722	-0.722247
C	-6.170058	3.575918	-2.961855
N	-6.297157	4.536455	-2.028335
H	-6.182725	2.658606	0.766182
H	-5.924951	1.475197	-3.384147
H	-6.373807	5.043515	-0.006529
H	-6.163111	3.920168	-4.000581
C	-5.292809	-2.776094	-2.391909
C	-5.173706	-4.124579	-2.025756
C	-5.150396	-2.438824	-3.745816
C	-4.924604	-5.078666	-3.010069
C	-4.889102	-3.444899	-4.673332
N	-4.788965	-4.734667	-4.303874
H	-5.269465	-4.426611	-0.983234
H	-5.229212	-1.402212	-4.071799
H	-4.815241	-6.144613	-2.788302
H	-4.737562	-3.219432	-5.736748
Cu	-6.246562	6.494683	-2.667495
Cu	6.223383	-6.450464	2.597219
Cu	5.718106	2.339263	-7.004307
N	2.940707	-0.622408	4.952752
N	0.810850	0.027564	5.737631
C	2.071648	0.300250	5.381875
C	2.483429	-1.877757	4.887550
N	1.239133	-2.234886	5.222601
C	0.434291	-1.252266	5.641433
C	-0.963872	-1.605842	6.007855
C	-1.846145	-0.638264	6.510024
C	-1.436817	-2.915493	5.839842
C	-3.152500	-1.006244	6.825629
C	-2.759536	-3.207643	6.164388
N	-3.588237	-2.267529	6.653088
H	-1.519280	0.391910	6.648837
H	-0.785608	-3.695711	5.447306
H	-3.891570	-0.298352	7.213382
H	-3.178753	-4.211051	6.014944
C	3.405943	-2.940139	4.402758
C	2.994026	-4.279859	4.356236
C	4.700413	-2.626726	3.963883
C	3.875997	-5.248212	3.882894
C	5.523985	-3.647112	3.491917
N	5.114031	-4.928422	3.463361
H	1.994042	-4.564215	4.682075

H	5.058044	-1.597532	3.977580
H	3.614660	-6.309123	3.818100
H	6.529463	-3.438207	3.106118
C	2.529977	1.714229	5.457183
C	1.644173	2.737342	5.825690
C	3.852974	2.059833	5.145385
C	2.102635	4.052568	5.864158
C	4.244294	3.395103	5.217299
N	3.379250	4.362157	5.573678
H	0.606066	2.513318	6.069140
H	4.569974	1.295722	4.846707
H	1.435114	4.886187	6.118174
H	5.259396	3.727323	4.979619
Cu	4.037378	6.314112	5.471379
N	2.582454	5.296353	-1.369670
N	0.462697	5.968524	-0.576267
C	1.770492	5.745090	-0.405674
C	2.002830	5.011801	-2.541165
N	0.698325	5.176301	-2.786976
C	-0.031436	5.679492	-1.785431
C	-1.479286	5.920328	-2.030292
C	-2.307814	6.424315	-1.017306
C	-2.050534	5.638641	-3.279907
C	-5.967220	-0.821919	4.569491
C	-3.413100	5.859089	-3.469472
N	-4.190547	6.350483	-2.487483
H	-1.903259	6.649728	-0.031009
H	-1.441176	5.245345	-4.093188
H	-3.905669	5.622080	-4.421090
C	2.853109	4.454619	-3.625350
C	4.248278	4.580494	-3.589904
C	2.274618	3.768973	-4.701976
C	5.009613	4.018336	-4.613891
C	3.105493	3.205316	-5.665858
N	4.442728	3.325791	-5.618479
H	4.738127	5.112460	-2.774285
H	1.193040	3.661280	-4.779391
H	6.099658	4.102988	-4.639397
H	2.675843	2.641109	-6.494179
C	2.350969	5.971970	0.945632
C	1.561296	6.458435	1.997353
C	3.691960	5.657474	1.209886
C	2.126517	6.604630	3.262128
C	4.185926	5.819338	2.502428
N	3.412448	6.286435	3.499396
H	0.513359	6.708360	1.834189
H	4.336713	5.270100	0.421406
H	1.562634	6.966730	4.127452
H	5.217112	5.546607	2.759504
Cu	-5.598583	-2.629734	6.941723
C	-3.659973	6.628630	-1.282524
H	-4.362229	7.013346	-0.536263
C	6.500602	10.461244	5.682730

C	6.193390	9.973533	4.427278
C	5.449802	8.781248	4.321032
N	5.042204	8.108813	5.381814
C	5.358993	8.549288	6.621080
C	6.079231	9.750060	6.831529
H	7.068283	11.387729	5.800855
H	6.514980	10.489301	3.521383
H	5.178947	8.371372	3.345995
C	4.725309	7.414286	10.129750
C	5.192734	8.218022	9.062996
C	4.936998	7.760487	7.747764
N	4.308928	6.587558	7.502788
C	3.877182	5.852347	8.511029
C	4.062654	6.233985	9.854707
H	4.891015	7.737420	11.160601
H	3.364273	4.922609	8.256040
H	3.678274	5.597264	10.652790
C	9.222788	-7.660394	6.181561
C	8.545516	-6.462492	6.303404
C	7.640435	-6.083616	5.291884
N	7.422333	-6.831856	4.226008
C	8.098882	-7.992848	4.068365
C	9.008943	-8.471694	5.041684
H	9.924944	-7.988410	6.952261
H	8.697912	-5.806697	7.161773
H	7.079375	-5.149543	5.359496
C	8.218910	-10.715956	1.505080
C	8.509039	-10.002550	2.692216
C	7.869037	-8.752071	2.868267
N	7.042929	-8.228061	1.933932
C	6.782025	-8.909362	0.833359
C	7.351656	-10.172494	0.577440
H	8.682050	-11.691324	1.335761
H	6.097367	-8.445194	0.120303
H	7.096803	-10.701359	-0.342045
C	-8.395497	-7.178197	-7.886888
C	-7.251647	-8.006357	-7.978945
C	-6.104753	-7.609150	-7.249868
N	-6.098289	-6.509804	-6.461428
C	-7.173485	-5.746493	-6.391363
C	-8.355299	-6.044738	-7.098422
H	-9.298270	-7.442506	-8.443378
H	-7.108040	-4.863877	-5.751786
H	-9.214535	-5.377696	-7.015626
C	-3.679189	-10.328134	-8.102146
C	-2.586237	-9.867395	-7.394060
C	-2.693715	-8.653973	-6.685752
N	-3.806849	-7.944007	-6.669222
C	-4.899502	-8.390574	-7.330186
C	-4.883218	-9.584437	-8.091278
H	-3.627649	-11.261573	-8.668364
H	-1.647640	-10.422876	-7.370211
H	-1.847958	-8.253932	-6.122776

C	-5.643944	-4.888285	11.214619
C	-6.808851	-4.383463	10.589547
C	-6.643170	-3.727716	9.345706
N	-5.425963	-3.547046	8.783717
C	-4.350424	-4.028173	9.379730
C	-4.415046	-4.716281	10.607670
H	-5.729059	-5.411829	12.170233
H	-3.395845	-3.866050	8.874698
H	-3.499186	-5.103953	11.056016
C	-10.164754	-2.780670	8.506661
C	-9.937640	-2.181030	7.283034
C	-8.624199	-2.144275	6.774002
N	-7.599823	-2.657450	7.430399
C	-7.793038	-3.221947	8.644485
C	-9.078433	-3.330279	9.228237
H	-11.172337	-2.833343	8.926860
H	-10.750271	-1.735535	6.707488
H	-8.407678	-1.687616	5.806045
C	9.851861	0.895177	-9.150584
C	8.997890	-0.098289	-8.703377
C	7.752514	0.263693	-8.154211
N	7.365598	1.523864	-8.051240
C	8.191601	2.507058	-8.473373
C	9.460512	2.248353	-9.047458
H	10.826807	0.645562	-9.576814
H	9.273330	-1.152396	-8.765941
H	7.053163	-0.492590	-7.790139
C	8.017424	6.238847	-8.622666
C	8.544086	4.934741	-8.770623
C	7.737404	3.863450	-8.320748
N	6.531330	4.058054	-7.739475
C	6.038893	5.280953	-7.635116
C	6.759808	6.409165	-8.072951
H	8.606371	7.098903	-8.951364
H	5.039711	5.366624	-7.209184
H	6.318482	7.400924	-7.963352
C	-10.882042	7.824628	-2.338166
C	-10.530626	6.621906	-1.756689
C	-9.198257	6.175799	-1.862799
N	-8.270036	6.871547	-2.493428
C	-8.584807	8.064842	-3.047251
C	-9.900616	8.586546	-3.015483
H	-11.907855	8.197237	-2.279756
H	-11.259495	6.016051	-1.216503
H	-8.885192	5.226698	-1.422424
C	-6.767531	10.693375	-4.994255
C	-7.834651	10.028904	-4.344073
C	-7.535602	8.807613	-3.693142
N	-6.280520	8.304517	-3.649388
C	-5.298258	8.934492	-4.267421
C	-5.500764	10.143184	-4.962950
H	-6.957642	11.633662	-5.517988
H	-4.309258	8.475028	-4.212889

H	-4.659157	10.621904	-5.465552
O	-5.674939	-4.484489	-8.719406
O	-4.042280	-9.746397	-4.188247
O	8.231653	4.283437	-5.251412
O	3.367251	4.127291	-8.681936
O	6.221321	5.600575	5.654646
O	1.517618	7.708532	8.009641
O	-6.934633	5.836110	-4.766479
O	-8.107915	7.971867	0.343699
O	4.912818	-8.017719	3.703728
O	9.548632	-4.764326	3.331471
O	-7.636092	0.283185	8.325816
O	-5.225531	-6.375894	7.645659
N	-6.489568	0.188289	7.893761
O	-6.027951	0.987941	7.049338
O	-5.739257	-0.768678	8.292690
N	-5.194280	-5.707952	6.614212
O	-4.288796	-5.841397	5.761847
O	-6.097873	-4.826453	6.403795
N	1.425624	7.369337	6.831521
O	0.510863	6.620101	6.424158
O	2.301338	7.768628	5.988414
N	6.970426	5.794118	4.636024
O	6.895042	4.982633	3.685287
O	7.716701	6.769781	4.604122
N	4.240622	-8.842969	2.993263
O	4.690584	-9.966978	2.779686
O	3.148689	-8.461683	2.518347
N	8.711836	-4.627209	2.442294
O	8.266878	-3.503100	2.114932
O	8.250406	-5.656669	1.839362
N	8.358634	3.063772	-5.454062
O	9.418981	2.563422	-5.830327
O	7.330073	2.310543	-5.298150
N	3.302601	2.895091	-8.707956
O	2.220974	2.287276	-8.662650
O	4.384583	2.202389	-8.735133
N	-4.646159	-4.322785	-8.066580
O	-3.930962	-5.335653	-7.750607
O	-4.278192	-3.193816	-7.672453
N	-4.190779	-8.636336	-3.682143
O	-5.042065	-7.821346	-4.179922
O	-3.514867	-8.257210	-2.699300
N	-6.961877	7.555222	0.187519
O	-6.291813	7.919257	-0.840503
O	-6.426692	6.761902	0.992047
N	-6.103801	6.014102	-5.723215
O	-5.191370	5.171151	-5.877077
O	-6.205453	7.005782	-6.441677
C	-9.214891	-3.983918	10.500937
C	-8.127959	-4.499083	11.148289
C	9.657561	-9.736578	4.829172
C	9.409061	-10.475956	3.707793

C	-6.068067	-9.974220	-8.805275
C	-7.200266	-9.211372	-8.760520
C	5.905151	9.452511	9.247489
C	6.340308	10.181470	8.177402
C	-10.171104	9.839979	-3.664541
C	-9.182785	10.526069	-4.311393
C	10.270380	3.356224	-9.472033
C	9.829438	4.642998	-9.343545
H	-11.190737	10.231589	-3.635516
H	-9.400725	11.472173	-4.812642
H	6.891963	11.112584	8.327592
H	6.098336	9.794338	10.267134
H	10.353013	-10.098995	5.589994
H	9.898357	-11.441710	3.560371
H	10.453063	5.477609	-9.672521
H	11.252863	3.147807	-9.902054
H	-6.042325	-10.895145	-9.392707
H	-8.092552	-9.509581	-9.316158
H	-8.243148	-5.004619	12.110059
H	-10.212293	-4.065952	10.939461

330

CAGEphen+NO3Cu5 SCF Done: -20715.0786021 A.U.

N	-2.092981	-3.341809	4.698980
N	-2.187106	-4.861971	2.893900
C	-1.579942	-4.335938	3.964200
C	-3.279426	-2.868842	4.302146
N	-3.955192	-3.342285	3.249540
C	-3.374334	-4.337600	2.571816
C	-4.083985	-4.875190	1.379475
C	-5.333447	-4.363634	1.000192
C	-3.511943	-5.885523	0.593011
C	-5.961254	-4.868373	-0.135743
C	-4.193785	-6.335803	-0.535989
N	-5.394330	-5.835185	-0.880239
H	-5.807889	-3.573084	1.580429
H	-2.540513	-6.307319	0.849331
H	-6.928651	-4.499808	-0.489925
H	-3.768035	-7.102072	-1.196711
C	-3.875753	-1.729595	5.051105
C	-5.082562	-1.155566	4.623811
C	-3.238027	-1.182628	6.173743
C	-5.584031	-0.048758	5.301400
C	-3.805416	-0.077675	6.807970
N	-4.944685	0.479318	6.360183
H	-5.611370	-1.557950	3.760622
H	-2.302800	-1.603933	6.541652
H	-6.506173	0.462777	5.008848
H	-3.348155	0.396843	7.681978
C	-0.246543	-4.875725	4.344847
C	0.357511	-5.890438	3.588066
C	0.448766	-4.366888	5.451326
C	1.621607	-6.348971	3.951774
C	1.706060	-4.881263	5.761466

N	2.270072	-5.853111	5.021183
H	-0.145944	-6.309467	2.717505
H	0.017815	-3.572315	6.059775
H	2.142715	-7.117765	3.367052
H	2.303546	-4.520662	6.604397
Cu	4.218974	-6.376916	5.464558
N	5.708192	-0.575715	1.711172
N	5.571856	-2.173019	-0.021915
C	5.541921	-1.827730	1.270356
C	5.905671	0.358645	0.774762
N	5.938988	0.100785	-0.537091
C	5.768437	-1.177078	-0.893199
C	5.782508	-1.502965	-2.345102
C	5.938303	-0.490481	-3.303178
C	5.618062	-2.822397	-2.790955
C	5.614083	-3.083474	-4.159653
N	5.760478	-2.097093	-5.062958
H	6.064522	0.548008	-2.999076
H	5.486643	-3.636230	-2.078423
H	5.999472	-0.058812	-5.437195
H	5.478056	-4.088057	-4.571816
C	6.085305	1.768370	1.215080
C	6.225934	2.800160	0.275582
C	6.099039	2.101255	2.577303
C	6.362339	4.112467	0.722750
C	6.251453	3.434573	2.951329
N	6.380372	4.410765	2.034346
H	6.215948	2.585209	-0.792437
H	5.986031	1.329603	3.338205
H	6.439900	4.953203	0.021246
H	6.259365	3.759739	3.996169
C	5.292330	-2.894703	2.277357
C	5.149810	-4.234502	1.888522
C	5.170658	-2.580111	3.638745
C	4.898483	-5.202744	2.858399
C	4.906044	-3.599106	4.551030
N	4.782983	-4.880462	4.159735
H	5.228862	-4.518918	0.839660
H	5.267839	-1.550778	3.982344
H	4.770504	-6.262602	2.618176
H	4.770078	-3.390756	5.619952
Cu	6.361634	6.357972	2.714667
Cu	-6.330117	-6.322599	-2.653516
Cu	-5.603760	2.277988	7.105406
N	-2.994424	-0.503784	-4.938163
N	-0.864677	0.132206	-5.734682
C	-2.117698	0.415018	-5.359861
C	-2.553970	-1.766328	-4.902656
N	-1.318641	-2.133846	-5.258966
C	-0.504703	-1.154213	-5.667088
C	0.884886	-1.519282	-6.054240
C	1.776268	-0.553512	-6.543519
C	1.341054	-2.838585	-5.918622

C	3.074526	-0.932811	-6.878347
C	2.656713	-3.141848	-6.261264
N	3.494777	-2.203306	-6.737080
H	1.462658	0.483804	-6.657544
H	0.682530	-3.617903	-5.536629
H	3.820737	-0.228781	-7.259811
H	3.063535	-4.153197	-6.134718
C	-3.485601	-2.825146	-4.427557
C	-3.093962	-4.171708	-4.417538
C	-4.768041	-2.501722	-3.961328
C	-3.983318	-5.137085	-3.951937
C	-5.599795	-3.519618	-3.498545
N	-5.209070	-4.807229	-3.505195
H	-2.103634	-4.463752	-4.765305
H	-5.109722	-1.467134	-3.946734
H	-3.735688	-6.202382	-3.913852
H	-6.596643	-3.305442	-3.092931
C	-2.557742	1.836101	-5.403980
C	-1.662887	2.853971	-5.765012
C	-3.872029	2.193588	-5.069722
C	-2.103956	4.175674	-5.774131
C	-4.245781	3.535094	-5.112652
N	-3.372564	4.497365	-5.462180
H	-0.630902	2.620612	-6.025140
H	-4.595767	1.433806	-4.776264
H	-1.427946	5.004796	-6.019667
H	-5.253639	3.877505	-4.858960
Cu	-4.010675	6.457530	-5.309504
N	-2.491764	5.293835	1.488240
N	-0.373050	5.958083	0.685401
C	-1.684845	5.751782	0.524329
C	-1.903450	4.982181	2.648443
N	-0.594749	5.127905	2.883577
C	0.130344	5.640990	1.883656
C	1.583587	5.859659	2.117105
C	2.407651	6.370946	1.104161
C	2.164533	5.547852	3.355036
C	5.913609	-0.824368	-4.655209
C	3.531938	5.746564	3.533104
N	4.305015	6.245342	2.551387
H	1.995504	6.618983	0.126481
H	1.558992	5.147767	4.167851
H	4.032030	5.485134	4.474369
C	-2.748808	4.415604	3.731601
C	-4.142777	4.557458	3.712954
C	-2.167099	3.705355	4.790379
C	-4.899728	3.986834	4.735492
C	-2.994166	3.134962	5.753616
N	-4.330362	3.271496	5.722644
H	-4.635044	5.108497	2.911568
H	-1.086089	3.584117	4.854541
H	-5.988395	4.083596	4.774142
H	-2.561511	2.552410	6.567588

C	-2.276538	6.009831	-0.816483
C	-1.493432	6.512199	-1.865634
C	-3.622152	5.709504	-1.073975
C	-2.069447	6.688260	-3.121764
C	-4.127135	5.900667	-2.358148
N	-3.359676	6.383239	-3.352387
H	-0.442239	6.751449	-1.707540
H	-4.262031	5.310692	-0.287226
H	-1.509708	7.062261	-3.984495
H	-5.162931	5.641361	-2.611318
Cu	5.504982	-2.577767	-7.051245
C	3.765349	6.552549	1.357562
H	4.463227	6.939212	0.608468
C	-6.509708	10.591048	-5.388504
C	-6.157302	10.092000	-4.149571
C	-5.398340	8.906630	-4.081643
N	-5.017560	8.251042	-5.162688
C	-5.376712	8.704037	-6.385734
C	-6.116273	9.898973	-6.558621
H	-7.091446	11.512063	-5.477265
H	-6.455755	10.593125	-3.227678
H	-5.093319	8.487536	-3.120594
C	-4.829104	7.625974	-9.926857
C	-5.277300	8.408117	-8.835925
C	-4.978513	7.935776	-7.535037
N	-4.328789	6.767794	-7.325076
C	-3.913804	6.053668	-8.355284
C	-4.141725	6.451989	-9.687578
H	-5.028719	7.960888	-10.947906
H	-3.381464	5.127725	-8.128209
H	-3.770613	5.832417	-10.505193
C	-9.463998	-7.364664	-6.178342
C	-8.739911	-6.195072	-6.305027
C	-7.791589	-5.868316	-5.315216
N	-7.575536	-6.638922	-4.264958
C	-8.295000	-7.773209	-4.103369
C	-9.251715	-8.200109	-5.055817
H	-10.201582	-7.651949	-6.932004
H	-8.888767	-5.521717	-7.150321
H	-7.193814	-4.957473	-5.387278
C	-8.448189	-10.530172	-1.578068
C	-8.745767	-9.786110	-2.744497
C	-8.061136	-8.559596	-2.921652
N	-7.186682	-8.084610	-2.005255
C	-6.918304	-8.794478	-0.924731
C	-7.530277	-10.038078	-0.670663
H	-8.945695	-11.488488	-1.408782
H	-6.193964	-8.369987	-0.226457
H	-7.268246	-10.592245	0.231740
C	8.390002	-7.439663	7.661493
C	7.235886	-8.253986	7.749027
C	6.087447	-7.827329	7.039300
N	6.088301	-6.712838	6.272319

C	7.173070	-5.962841	6.206224
C	8.357566	-6.290665	6.895508
H	9.294478	-7.726898	8.203671
H	7.113534	-5.067211	5.584435
H	9.224962	-5.633753	6.816937
C	3.633668	-10.529835	7.862739
C	2.540298	-10.040889	7.174525
C	2.657283	-8.815314	6.488974
N	3.779625	-8.119981	6.475285
C	4.872530	-8.593938	7.116529
C	4.847464	-9.802116	7.854429
H	3.575019	-11.473406	8.411191
H	1.594130	-10.583301	7.149077
H	1.811568	-8.393064	5.942389
C	5.492329	-4.634671	-11.420710
C	6.664633	-4.146540	-10.795897
C	6.518742	-3.558610	-9.516143
N	5.311840	-3.423387	-8.920005
C	4.229456	-3.890194	-9.515054
C	4.275550	-4.513595	-10.777957
H	5.562422	-5.105828	-12.404413
H	3.284602	-3.767751	-8.981659
H	3.355064	-4.891479	-11.225175
C	10.048159	-2.622633	-8.698619
C	9.841613	-2.096083	-7.438356
C	8.538562	-2.099448	-6.902150
N	7.504399	-2.582527	-7.565987
C	7.677970	-3.077849	-8.812760
C	8.951768	-3.141417	-9.427531
H	11.047064	-2.641351	-9.141753
H	10.662422	-1.676702	-6.854839
H	8.338411	-1.699331	-5.905921
C	-9.726837	0.848882	9.282497
C	-8.892498	-0.147382	8.805371
C	-7.650260	0.208265	8.245051
N	-7.248240	1.464974	8.159368
C	-8.054966	2.451018	8.610830
C	-9.319095	2.198506	9.198089
H	-10.698946	0.604297	9.718032
H	-9.181031	-1.198770	8.852966
H	-6.966139	-0.550214	7.857346
C	-7.829192	6.176952	8.824363
C	-8.371003	4.877343	8.956388
C	-7.584860	3.803917	8.476333
N	-6.384500	3.993384	7.881656
C	-5.877376	5.211436	7.792224
C	-6.577103	6.340836	8.260256
H	-8.402148	7.038513	9.176546
H	-4.883216	5.291771	7.353650
H	-6.124257	7.328597	8.162175
C	11.017060	7.614713	2.400325
C	10.639722	6.439803	1.779575
C	9.299490	6.015778	1.877589

N	8.388067	6.707027	2.536974
C	8.727935	7.875094	3.128682
C	10.053546	8.372026	3.108019
H	12.049721	7.969129	2.349740
H	11.354186	5.838966	1.215095
H	8.966375	5.088158	1.407322
C	6.969527	10.474037	5.167264
C	8.021220	9.809762	4.492196
C	7.695828	8.616591	3.802959
N	6.430829	8.140177	3.747318
C	5.463185	8.769780	4.388250
C	5.691796	9.951039	5.121831
H	7.180081	11.392593	5.720880
H	4.464887	8.332362	4.322160
H	4.861298	10.430010	5.642349
O	5.714995	-4.716914	8.567800
O	3.962843	-9.876913	3.955757
O	-8.104501	4.291829	5.415452
O	-3.208833	3.999866	8.788246
O	-6.206692	5.754548	-5.463751
O	-1.501893	7.830112	-7.851142
O	7.060771	5.640058	4.796202
O	8.202935	7.837947	-0.299864
O	-5.083210	-7.896527	-3.810238
O	-9.670345	-4.594076	-3.293709
O	7.555136	0.342825	-8.443658
O	5.032193	-6.283384	-7.817055
N	6.426108	0.264407	-7.963910
O	6.019287	1.056383	-7.085901
O	5.638596	-0.668046	-8.349391
N	5.069457	-5.636613	-6.772698
O	4.202336	-5.764927	-5.879121
O	6.004052	-4.782704	-6.588598
N	-1.413469	7.502473	-6.669745
O	-0.504170	6.750003	-6.254075
O	-2.286502	7.915687	-5.830799
N	-6.953919	5.954767	-4.444820
O	-6.884103	5.145130	-3.492893
O	-7.694015	6.935516	-4.415665
N	-4.394120	-8.721466	-3.115751
O	-4.841721	-9.842203	-2.882655
O	-3.286664	-8.341890	-2.674998
N	-8.804307	-4.464744	-2.431538
O	-8.338474	-3.345267	-2.118880
O	-8.334799	-5.498821	-1.843239
N	-8.251754	3.072108	5.603493
O	-9.316188	2.586881	5.988157
O	-7.240160	2.301743	5.422894
N	-3.164101	2.766604	8.791901
O	-2.093253	2.142209	8.719660
O	-4.256780	2.091066	8.822726
N	4.679841	-4.535522	7.930414
O	3.951591	-5.536392	7.606777

O	4.317387	-3.396870	7.559151
N	4.125217	-8.758909	3.471934
O	4.994101	-7.968637	3.978636
O	3.446625	-8.347878	2.503630
N	7.046336	7.462621	-0.119770
O	6.417065	7.833013	0.930830
O	6.460626	6.702882	-0.922774
N	6.246371	5.813593	5.767540
O	5.318646	4.986500	5.915216
O	6.378310	6.786753	6.506374
C	9.067403	-3.722747	-10.736814
C	7.972362	-4.215181	-11.388191
C	-9.946049	-9.440089	-4.841150
C	-9.695043	-10.206252	-3.738461
C	6.033765	-10.221525	8.548945
C	7.175728	-9.473164	8.507665
C	-6.010795	9.635345	-8.982523
C	-6.423066	10.344370	-7.890197
C	10.351215	9.597369	3.797558
C	9.378906	10.280814	4.470941
C	-10.108266	3.309016	9.653643
C	-9.652152	4.592052	9.541887
H	11.378327	9.969509	3.777414
H	9.617293	11.205157	5.002562
H	-6.990536	11.270187	-8.011410
H	-6.238438	9.988093	-9.991280
H	-10.678482	-9.761878	-5.585356
H	-10.219429	-11.153223	-3.589807
H	-10.260088	5.428651	9.894339
H	-11.087399	3.105720	10.093645
H	6.001248	-11.153292	9.118676
H	8.069234	-9.794049	9.048513
H	8.072116	-4.666541	-12.378228
H	10.055841	-3.768962	-11.200159

330

CAGEphen+NO3Fe SCF Done: -18454.9195882 A.U.

N	1.760509	-4.563741	3.292059
N	3.815961	-3.402320	3.332836
C	2.633805	-3.728972	3.867963
C	2.134067	-5.094711	2.121853
N	3.281724	-4.796920	1.501115
C	4.087101	-3.934686	2.134875
C	5.336340	-3.512526	1.453623
C	5.569440	-3.834802	0.108685
C	6.303356	-2.743905	2.116272
C	6.714336	-3.355049	-0.517489
C	7.433863	-2.315899	1.424987
N	7.631835	-2.598400	0.119973
H	4.850908	-4.437099	-0.447029
H	6.172434	-2.474823	3.164133
H	6.930866	-3.566975	-1.565527
H	8.209734	-1.723445	1.911574
C	1.214795	-6.066130	1.476705

C	1.597219	-6.793149	0.340824
C	-0.070648	-6.291002	1.990778
C	0.703726	-7.697116	-0.229507
C	-0.909735	-7.200155	1.357193
N	-0.536960	-7.897007	0.263864
H	2.588107	-6.659369	-0.093050
H	-0.412010	-5.754546	2.875566
H	0.971631	-8.283144	-1.110244
H	-1.917515	-7.404085	1.721601
C	2.255183	-3.111025	5.163270
C	3.162388	-2.332819	5.895391
C	0.962970	-3.269054	5.685089
C	2.753404	-1.739051	7.087200
C	0.625388	-2.631706	6.872965
N	1.497786	-1.869882	7.564952
H	4.180843	-2.186827	5.536039
H	0.225255	-3.873150	5.157779
H	3.434940	-1.132950	7.685760
H	-0.370112	-2.716799	7.310975
Fe	0.847846	-0.819926	9.171807
N	-3.081737	2.599425	4.406810
N	-1.094971	3.868725	4.293060
C	-1.834700	2.877516	4.803230
C	-3.590292	3.395353	3.457589
N	-2.938571	4.439591	2.931018
C	-1.687318	4.631702	3.366215
C	-0.897405	5.739748	2.773134
C	-1.480082	6.673340	1.905100
C	0.470649	5.875059	3.051762
C	1.192380	6.895418	2.444319
N	0.629176	7.783680	1.599298
H	-2.541187	6.615883	1.663047
H	0.967225	5.179498	3.727575
H	-1.115239	8.427776	0.676531
H	2.259450	7.033651	2.624199
C	-4.941939	3.075519	2.934095
C	-5.598826	3.925185	2.033234
C	-5.589160	1.885323	3.297245
C	-6.842027	3.557405	1.524001
C	-6.823520	1.581740	2.734942
N	-7.443456	2.394843	1.854822
H	-5.140512	4.864511	1.724476
H	-5.121176	1.195092	3.998706
H	-7.379335	4.195006	0.820091
H	-7.356841	0.661844	2.978714
C	-1.230357	2.032629	5.864089
C	0.120866	2.169891	6.213348
C	-1.984998	1.070550	6.549709
C	0.658322	1.347578	7.197002
C	-1.376943	0.292730	7.531943
N	-0.070811	0.418776	7.849353
H	0.747014	2.908567	5.713795
H	-3.041183	0.930526	6.320290

H	1.704926	1.416538	7.496910
H	-1.935177	-0.465194	8.084025
Fe	-9.153200	1.768522	0.960864
Fe	9.161090	-1.775886	-0.927238
Fe	-1.885107	-9.112337	-0.646811
N	3.980656	1.790474	-4.107463
N	2.683117	3.743688	-3.831108
C	2.985920	2.608601	-4.471154
C	4.686140	2.161583	-3.032177
N	4.448424	3.278166	-2.332167
C	3.435723	4.039195	-2.764542
C	3.107102	5.271973	-2.005635
C	1.941904	5.999675	-2.288299
C	3.930904	5.737897	-0.971654
C	1.638141	7.123263	-1.528269
C	3.563985	6.879738	-0.263521
N	2.427448	7.559889	-0.525185
H	1.272180	5.679696	-3.086121
H	4.851622	5.212279	-0.718891
H	0.738275	7.713594	-1.706882
H	4.179962	7.267559	0.549503
C	5.781709	1.268833	-2.577474
C	6.429660	1.482718	-1.352282
C	6.190595	0.170438	-3.346850
C	7.420731	0.597597	-0.941537
C	7.199906	-0.663073	-2.872579
N	7.802634	-0.464319	-1.681031
H	6.150554	2.325774	-0.720490
H	5.723489	-0.033950	-4.309983
H	7.945550	0.720100	0.007006
H	7.540879	-1.526926	-3.445521
C	2.151470	2.222663	-5.636156
C	1.202304	3.102868	-6.174096
C	2.267432	0.952852	-6.219722
C	0.408478	2.688936	-7.240280
C	1.428754	0.608761	-7.274042
N	0.505536	1.454180	-7.777231
H	1.080753	4.104171	-5.761522
H	2.995161	0.235730	-5.840371
H	-0.341974	3.347976	-7.679711
H	1.474459	-0.371504	-7.750628
Fe	-0.786246	0.792807	-9.196510
N	-3.375268	-3.082015	-3.920417
N	-4.591124	-1.089431	-3.568738
C	-3.713016	-1.831416	-4.253757
C	-3.986868	-3.592391	-2.844739
N	-4.918185	-2.941269	-2.137321
C	-5.180297	-1.686825	-2.524966
C	-6.166890	-0.902307	-1.740329
C	-6.360182	0.464876	-1.987027
C	-6.924748	-1.492447	-0.719306
C	-0.692978	7.678851	1.347988
C	-7.821463	-0.714291	0.009322

N	-7.983143	0.606325	-0.219067
H	-5.800606	0.967299	-2.775523
H	-6.815440	-2.552797	-0.492547
H	-8.432866	-1.143523	0.804585
C	-3.574440	-4.943685	-2.390640
C	-2.451347	-5.573077	-2.947610
C	-4.258416	-5.616007	-1.368690
C	-2.041703	-6.802852	-2.446858
C	-3.795731	-6.855512	-0.932647
N	-2.691054	-7.435644	-1.447805
H	-1.891549	-5.092978	-3.749872
H	-5.142134	-5.172679	-0.910034
H	-1.164753	-7.321409	-2.836952
H	-4.306170	-7.409454	-0.143473
C	-3.066966	-1.231756	-5.448268
C	-3.204151	0.133007	-5.739854
C	-2.296636	-2.010171	-6.323318
C	-2.560677	0.659158	-6.854485
C	-1.697614	-1.413113	-7.429540
N	-1.814699	-0.093805	-7.689449
H	-3.800222	0.778038	-5.094673
H	-2.168501	-3.077685	-6.145225
H	-2.632063	1.715662	-7.116905
H	-1.102150	-1.992085	-8.136800
Fe	1.804863	9.116516	0.623328
C	-7.262782	1.177666	-1.206455
H	-7.439602	2.243447	-1.357871
C	-3.486877	-0.461780	-12.940218
C	-3.899481	-0.868742	-11.682431
C	-3.127482	-0.529572	-10.555741
N	-2.008166	0.178959	-10.647416
C	-1.602894	0.592792	-11.873342
C	-2.299868	0.294123	-13.064809
H	-4.064288	-0.718971	-13.831733
H	-4.809972	-1.455057	-11.548252
H	-3.425283	-0.845047	-9.555305
C	1.318055	2.554307	-13.113643
C	0.117167	1.811166	-13.151755
C	-0.396218	1.360360	-11.916011
N	0.193562	1.617754	-10.723429
C	1.324682	2.312568	-10.713392
C	1.921060	2.800143	-11.891534
H	1.757840	2.926082	-14.042466
H	1.777588	2.494819	-9.738077
H	2.849795	3.369060	-11.823427
C	13.130152	-0.118255	-3.021610
C	11.937153	0.527966	-3.298291
C	10.743854	0.060275	-2.716155
N	10.711983	-0.988282	-1.902930
C	11.876728	-1.618484	-1.615591
C	13.124139	-1.232419	-2.153061
H	14.069711	0.221822	-3.464239
H	11.903316	1.392785	-3.962988

H	9.790931	0.550226	-2.919829
C	12.782589	-4.552564	0.504009
C	12.949729	-3.463184	-0.379895
C	11.790855	-2.731296	-0.720533
N	10.558221	-3.014549	-0.231366
C	10.426497	-4.043150	0.598035
C	11.517634	-4.841700	0.987839
H	13.647603	-5.152116	0.798327
H	9.422186	-4.242658	0.972845
H	11.351211	-5.673942	1.673704
C	-0.280900	1.376444	13.269356
C	0.487250	0.192058	13.215344
C	0.734699	-0.352775	11.936304
N	0.264973	0.192593	10.788084
C	-0.448654	1.309352	10.863715
C	-0.744614	1.934292	12.089824
H	-0.500877	1.839030	14.234741
H	-0.804232	1.727250	9.921106
H	-1.339538	2.849134	12.092445
C	2.778961	-3.372771	12.725364
C	2.977384	-3.818226	11.429190
C	2.433923	-3.090075	10.354595
N	1.723183	-1.982725	10.533016
C	1.513019	-1.545407	11.799164
C	2.026199	-2.197054	12.942212
H	3.197539	-3.915587	13.576531
H	3.555198	-4.720819	11.223764
H	2.583736	-3.413309	9.324022
C	4.233463	12.679995	-1.446543
C	3.375858	12.871954	-0.340426
C	2.731760	11.726673	0.176575
N	2.909492	10.479722	-0.323253
C	3.714811	10.325309	-1.367073
C	4.397785	11.404304	-1.959456
H	4.755818	13.533949	-1.885000
H	3.828970	9.310667	-1.750583
H	5.050541	11.218054	-2.813911
C	0.722199	13.129372	2.990135
C	0.129228	11.951353	3.412664
C	0.414027	10.750181	2.737129
N	1.244253	10.695749	1.702275
C	1.840105	11.841765	1.289472
C	1.609814	13.098464	1.891419
H	0.510647	14.076741	3.492212
H	-0.562910	11.937646	4.256170
H	-0.047454	9.810689	3.042695
C	-0.466393	-13.347332	1.070797
C	0.230036	-12.221199	1.476300
C	-0.145593	-10.958029	0.981804
N	-1.152806	-10.797180	0.132007
C	-1.849083	-11.892096	-0.259328
C	-1.546750	-13.202315	0.172199
H	-0.191329	-14.340141	1.435523

H	1.069260	-12.294536	2.169964
H	0.389045	-10.056370	1.282756
C	-4.755242	-12.440210	-2.534259
C	-3.703941	-12.746358	-1.641532
C	-2.935666	-11.662938	-1.161546
N	-3.171372	-10.371828	-1.501600
C	-4.165002	-10.109054	-2.342317
C	-4.980302	-11.119560	-2.885053
H	-5.379179	-13.242598	-2.935775
H	-4.326691	-9.060616	-2.594645
H	-5.784241	-10.845616	-3.570164
C	-12.372568	4.220427	3.512025
C	-11.035826	4.378829	3.837080
C	-10.056302	3.686017	3.101156
N	-10.362955	2.874845	2.095798
C	-11.669614	2.703005	1.779328
C	-12.725536	3.358498	2.449828
H	-13.152098	4.751537	4.063926
H	-10.725795	5.035645	4.651499
H	-8.996663	3.796833	3.333967
C	-13.471596	0.689750	-0.793628
C	-13.278450	1.582761	0.283866
C	-11.947342	1.806434	0.699391
N	-10.875669	1.201310	0.130652
C	-11.082539	0.367283	-0.881633
C	-12.369741	0.086338	-1.376324
H	-14.482241	0.482652	-1.154261
H	-10.199401	-0.100815	-1.317502
H	-12.480753	-0.609793	-2.209297
O	-1.268161	-2.334126	11.270729
O	3.324046	0.702013	11.032652
O	-0.666843	-9.209919	-2.226812
O	-3.945873	-10.828580	1.507704
O	0.349438	-0.859337	-9.376544
O	-2.316407	3.261471	-10.871288
O	-9.244043	0.279705	2.307400
O	-10.888153	4.221615	-0.585598
O	9.488356	-0.476787	0.579775
O	10.634614	-3.638183	-3.271127
O	0.110035	11.561543	-1.133334
O	4.280059	10.568103	2.328622
N	-0.377940	10.541995	-0.665817
O	-1.499574	10.484989	-0.168423
O	0.322681	9.431834	-0.705557
N	4.358037	9.370780	2.081412
O	5.413240	8.752009	1.998771
O	3.256604	8.677919	1.913574
N	-1.956225	3.446910	-9.715335
O	-1.666287	4.545199	-9.253051
O	-1.891362	2.425904	-8.893815
N	0.180381	-1.687168	-10.381749
O	-0.377022	-2.755361	-10.144005
O	0.594862	-1.364554	-11.486688

N	10.519940	-0.634944	1.376991
O	11.623123	-0.278362	0.987163
O	10.309765	-1.124593	2.483772
N	9.481367	-3.267292	-3.453550
O	8.969132	-3.128684	-4.558653
O	8.716316	-3.018860	-2.416835
N	0.054439	-10.263706	-2.529780
O	-0.510701	-11.321796	-2.776335
O	1.270724	-10.109265	-2.567039
N	-4.120169	-9.678234	1.129545
O	-5.224929	-9.190416	0.906245
O	-3.073718	-8.902247	0.964537
N	-1.656917	-2.033188	10.148692
O	-0.787863	-1.957078	9.168631
O	-2.828700	-1.814141	9.861910
N	3.459379	0.224570	9.914548
O	2.479962	0.355682	9.049720
O	4.469880	-0.364030	9.538799
N	-9.684560	4.319356	-0.383153
O	-8.947630	3.233076	-0.387146
O	-9.104393	5.383716	-0.193867
N	-10.331435	-0.443048	2.448550
O	-10.296752	-1.597259	2.031460
O	-11.305054	0.056741	2.995760
C	2.281204	14.251990	1.353408
C	3.122807	14.144113	0.282670
C	14.293915	-1.987049	-1.788689
C	14.209967	-3.057155	-0.943587
C	1.756893	-1.629038	14.236686
C	1.024113	-0.483428	14.366943
C	-0.604554	1.491003	-14.354800
C	-1.763458	0.768829	-14.312913
C	-14.074937	3.112351	2.014947
C	-14.339256	2.266030	0.975623
C	-2.345269	-14.291806	-0.323801
C	-3.374695	-14.074180	-1.195169
H	-14.889738	3.622045	2.534726
H	-15.368216	2.089840	0.653050
H	-2.303517	0.534713	-15.233444
H	-0.205428	1.842042	-15.309436
H	15.257603	-1.686218	-2.206630
H	15.105409	-3.623561	-0.676721
H	-3.971742	-14.910455	-1.566698
H	-2.108125	-15.304532	0.010878
H	2.156794	-2.133893	15.119325
H	0.830252	-0.059853	15.355308
H	3.623079	15.028449	-0.119252
H	2.099549	15.223516	1.819225

330

CAGEphen+NO3NiISOMER SCF Done: -19922.2005251 A.U.

N	3.158056	-3.965668	-3.352620
N	2.674566	-5.188196	-1.392684
C	2.417599	-4.835943	-2.657400

C	4.222265	-3.459552	-2.719368
N	4.576054	-3.792150	-1.473136
C	3.762688	-4.642853	-0.837920
C	4.060348	-4.962819	0.584232
C	5.234062	-4.503324	1.197162
C	3.146066	-5.688790	1.360646
C	5.449673	-4.763499	2.548062
C	3.410009	-5.895769	2.711035
N	4.539250	-5.434432	3.284196
H	5.969909	-3.930481	0.633778
H	2.224444	-6.072017	0.924165
H	6.334626	-4.385406	3.075176
H	2.697263	-6.434854	3.343095
C	5.028605	-2.424513	-3.419915
C	6.224449	-1.944963	-2.868509
C	4.576698	-1.859517	-4.620852
C	6.912717	-0.918965	-3.510859
C	5.297881	-0.820043	-5.199936
N	6.437770	-0.362842	-4.644874
H	6.610745	-2.351564	-1.934436
H	3.656041	-2.206572	-5.088706
H	7.828909	-0.486115	-3.089406
H	4.946801	-0.340723	-6.119401
C	1.220453	-5.423116	-3.318163
C	0.344185	-6.253798	-2.604380
C	0.925998	-5.139810	-4.659944
C	-0.797520	-6.743958	-3.231558
C	-0.226703	-5.669872	-5.234793
N	-1.065947	-6.442319	-4.517810
H	0.540385	-6.504747	-1.562528
H	1.583559	-4.503289	-5.251288
H	-1.533057	-7.378532	-2.711037
H	-0.508689	-5.462347	-6.273620
Ni	-2.716698	-7.018996	-5.309012
N	-5.009735	-0.751746	-3.353253
N	-5.569091	-2.067033	-1.474327
C	-5.104039	-1.926603	-2.720477
C	-5.394101	0.324419	-2.658141
N	-5.828627	0.277595	-1.393794
C	-5.899843	-0.937514	-0.839094
C	-6.325264	-1.035507	0.583202
C	-6.502496	0.119492	1.357930
C	-6.507684	-2.281835	1.198004
C	-6.840292	-2.338199	2.549043
N	-6.972919	-1.213663	3.283154
H	-6.378058	1.109215	0.920060
H	-6.374604	-3.205597	0.635876
H	-6.928964	0.882148	3.339037
H	-6.948302	-3.293317	3.079288
C	-5.303214	1.654900	-3.318545
C	-5.587975	2.828960	-2.605826
C	-4.906346	1.768307	-4.659061
C	-5.441223	4.062780	-3.232938

C	-4.788361	3.031571	-5.233837
N	-5.041731	4.144325	-4.517947
H	-5.906748	2.784310	-1.564998
H	-4.681180	0.880609	-5.249405
H	-5.626262	5.017486	-2.714319
H	-4.464089	3.172165	-6.271669
C	-4.611771	-3.142403	-3.421670
C	-4.797636	-4.418155	-2.872205
C	-3.894709	-3.033336	-4.621508
C	-4.254349	-5.527369	-3.515214
C	-3.356134	-4.177864	-5.201068
N	-3.532911	-5.393988	-4.647663
H	-5.344763	-4.549708	-1.939273
H	-3.732783	-2.062242	-5.088102
H	-4.341318	-6.537414	-3.095570
H	-2.763919	-4.113439	-6.119517
Ni	-4.716832	5.861919	-5.309283
Ni	4.940201	-5.585203	5.171744
Ni	7.437263	1.155772	-5.307309
N	1.332604	0.276019	5.780687
N	-0.909377	1.013876	5.781770
C	0.408661	1.243025	5.778060
C	0.872072	-0.980039	5.777784
N	-0.427358	-1.296819	5.781694
C	-1.284814	-0.269852	5.778448
C	-2.740596	-0.573414	5.726624
C	-3.693964	0.452514	5.802379
C	-3.190685	-1.891126	5.557806
C	-5.046374	0.140248	5.687004
C	-4.555122	-2.137699	5.436035
N	-5.449380	-1.130486	5.492916
H	-3.386391	1.489056	5.936886
H	-2.482842	-2.717272	5.498514
H	-5.827193	0.909074	5.721127
H	-4.963655	-3.147882	5.270519
C	1.863323	-2.088509	5.725127
C	1.453043	-3.427404	5.803673
C	3.228943	-1.818455	5.553310
C	2.400643	-4.441683	5.688880
C	4.125621	-2.876047	5.432041
N	3.702154	-4.154465	5.492381
H	0.402076	-3.680244	5.940393
H	3.589484	-0.792185	5.491510
H	2.126381	-5.502438	5.725781
H	5.204114	-2.722670	5.264176
C	0.873686	2.655511	5.726062
C	-0.042413	3.704159	5.557093
C	2.238832	2.968153	5.802196
C	0.426321	5.009107	5.435929
C	2.644657	4.295552	5.687599
N	1.745651	5.279968	5.493735
H	-1.111772	3.504239	5.497370
H	2.982660	2.183449	5.936681

H	-0.244262	5.867986	5.270307
H	3.700879	4.587393	5.722620
Ni	2.366357	7.066989	5.173703
N	3.157700	4.912934	-1.392571
N	0.996883	5.859228	-1.472612
C	2.140486	5.580971	-0.837412
C	2.981846	4.515078	-2.657641
N	1.858302	4.721666	-3.353334
C	0.886965	5.388491	-2.719677
C	-0.412163	5.569695	-3.420923
C	-1.428781	6.358513	-2.865564
C	-0.672042	4.902392	-4.626249
C	-6.813805	-0.005042	2.708369
C	-1.932612	5.006429	-5.206083
N	-2.901911	5.757506	-4.646735
H	-1.272505	6.891204	-1.928088
H	0.091369	4.283954	-5.097064
H	-2.169711	4.467072	-6.128807
C	4.088798	3.771319	-3.317983
C	5.246575	3.428378	-2.604272
C	3.989820	3.372688	-4.659101
C	6.241303	2.683355	-3.230846
C	5.024551	2.638057	-5.233362
N	6.113400	2.298235	-4.516469
H	5.366385	3.725184	-1.562885
H	3.109554	3.623261	-5.250329
H	7.159077	2.364131	-2.710830
H	4.984932	2.288275	-6.271611
C	2.267859	5.997112	0.585317
C	1.278999	6.776731	1.200875
C	3.357012	5.573271	1.359762
C	1.395986	7.091456	2.552314
C	3.404324	5.903610	2.710594
N	2.436358	6.644079	3.286168
H	0.412247	7.123343	0.639063
H	4.152460	4.971730	0.921396
H	0.621965	7.660952	3.083081
H	4.230482	5.559637	3.340856
Ni	-7.307238	-1.485344	5.170046
C	-2.661440	6.440464	-3.508060
H	-3.498136	7.009946	-3.082808
C	3.532251	11.614734	4.805456
C	3.918266	10.768288	3.780894
C	3.599635	9.398081	3.848522
N	2.933272	8.887413	4.874506
C	2.596560	9.695282	5.908802
C	2.853779	11.081680	5.925068
H	3.754917	12.683837	4.763104
H	4.465213	11.141111	2.913868
H	3.900678	8.710779	3.060875
C	0.970201	9.107173	9.204664
C	1.569064	9.816934	8.141025
C	1.979963	9.060188	7.024393

N	1.833264	7.714065	6.936504
C	1.284580	7.067375	7.956056
C	0.839592	7.733113	9.112171
H	0.618748	9.645448	10.088234
H	1.184105	5.986787	7.860786
H	0.383931	7.152903	9.915730
C	7.416814	-5.385122	9.195071
C	6.290590	-4.586964	9.104563
C	5.487773	-4.643270	7.951228
N	5.771384	-5.443485	6.932401
C	6.865664	-6.241339	7.018258
C	7.730002	-6.260359	8.132220
H	8.061486	-5.347050	10.076493
H	6.017473	-3.900983	9.907566
H	4.600740	-4.017745	7.857556
C	8.298131	-8.865756	4.800281
C	8.178386	-8.009297	5.918441
C	7.105153	-7.094724	5.903649
N	6.233690	-6.985766	4.871925
C	6.340215	-7.820328	3.847456
C	7.368523	-8.780336	3.778667
H	9.113416	-9.592245	4.756852
H	5.591499	-7.740196	3.062330
H	7.415810	-9.442363	2.913007
C	-6.617430	-8.960414	-7.093711
C	-5.471008	-9.712442	-6.749477
C	-4.366284	-8.991059	-6.251878
N	-4.376810	-7.649375	-6.063433
C	-5.447639	-6.956162	-6.423885
C	-6.594647	-7.584583	-6.946725
H	-7.504178	-9.467978	-7.481557
H	-5.401214	-5.875483	-6.308165
H	-7.452065	-6.970788	-7.225965
C	-1.792141	-11.634310	-5.761695
C	-0.751914	-10.817059	-5.357224
C	-0.956013	-9.429386	-5.253782
N	-2.123143	-8.865057	-5.533196
C	-3.145475	-9.656810	-5.944761
C	-3.039422	-11.056010	-6.083781
H	-1.658555	-12.716233	-5.836255
H	0.225386	-11.229455	-5.102709
H	-0.151505	-8.769631	-4.930720
C	-8.386584	-3.709290	9.200950
C	-9.298927	-3.544315	8.135962
C	-8.845776	-2.812261	7.019209
N	-7.605244	-2.269275	6.932498
C	-6.772445	-2.422418	7.953114
C	-7.129718	-3.138737	9.109439
H	-8.679600	-4.281189	10.084710
H	-5.785091	-1.971844	7.858530
H	-6.400651	-3.244387	9.913973
C	-11.830558	-2.741704	4.795450
C	-11.287179	-1.986998	3.770619

C	-9.940484	-1.580516	3.839926
N	-9.167476	-1.902093	4.867757
C	-9.701933	-2.595084	5.902132
C	-11.032209	-3.062696	5.916890
H	-12.868403	-3.081316	4.751902
H	-11.881400	-1.699910	2.902080
H	-9.493294	-0.978368	3.051992
C	11.063649	-1.252724	-7.102048
C	9.861637	-1.921260	-6.949703
C	8.745620	-1.241987	-6.423417
N	8.811229	0.032400	-6.064611
C	9.966955	0.712596	-6.258126
C	11.142404	0.116495	-6.759546
H	11.945336	-1.766900	-7.492705
H	9.758158	-2.971063	-7.227348
H	7.787289	-1.742617	-6.303421
C	10.970123	4.264102	-5.774339
C	11.091977	2.894581	-6.095985
C	9.933962	2.102968	-5.951982
N	8.738626	2.592537	-5.536146
C	8.644597	3.885607	-5.257200
C	9.743741	4.756339	-5.365401
H	11.839864	4.920883	-5.852767
H	7.672223	4.252516	-4.930526
H	9.613005	5.809036	-5.111032
C	-9.177883	7.355670	-5.785902
C	-8.988441	6.048141	-5.376040
C	-7.683643	5.535277	-5.264533
N	-6.612222	6.266045	-5.541362
C	-6.788329	7.545190	-5.958500
C	-8.054176	8.148812	-6.105446
H	-10.182480	7.777832	-5.866683
H	-9.833575	5.406409	-5.123286
H	-7.513095	4.510366	-4.936971
C	-4.451652	10.207442	-7.103738
C	-5.675879	9.587622	-6.764190
C	-5.601944	8.271922	-6.262285
N	-4.433665	7.614364	-6.065812
C	-3.297990	8.198100	-6.421700
C	-3.270038	9.504199	-6.948089
H	-4.449126	11.228017	-7.494610
H	-2.383790	7.621388	-6.299062
H	-2.309666	9.942205	-7.223244
O	-3.669277	-5.427650	-8.256572
O	-2.905380	-10.032883	-2.982105
O	10.147080	2.502386	-2.987874
O	6.838788	1.573518	-7.566546
O	4.521233	6.775701	6.128905
O	-0.913493	8.544000	6.437503
O	-4.784444	5.134372	-7.569502
O	-7.247381	7.528494	-2.994884
O	3.611374	-7.303825	6.123297
O	7.860798	-3.476351	6.421798

O	-9.312688	1.266742	4.452963
O	-6.952910	-5.064238	6.436617
N	-8.294430	1.325724	5.151653
O	-7.368424	2.129910	4.877453
O	-8.133432	0.526897	6.123467
N	-7.119261	-4.772987	5.243489
O	-6.117130	-4.484487	4.518506
O	-8.258602	-4.720899	4.736623
N	-0.577661	8.545794	5.244528
O	-0.826722	7.534759	4.517242
O	0.035726	9.508591	4.740085
N	5.294771	6.517662	5.157531
O	5.528870	5.314250	4.881255
O	5.752637	7.430365	4.460505
N	2.999302	-7.846282	5.153677
O	3.560054	-8.699331	4.456353
O	1.839164	-7.448675	4.880002
N	7.686070	-3.763410	5.228853
O	6.936923	-3.035750	4.507040
O	8.203269	-4.779344	4.719435
N	9.656594	1.369369	-2.881218
O	10.161524	0.382686	-3.456439
O	8.596301	1.200554	-2.203575
N	6.068947	0.621963	-7.898946
O	4.827973	0.791043	-7.794484
O	6.534343	-0.466900	-8.253932
N	-2.493768	-5.567593	-7.900455
O	-2.054246	-6.709237	-7.565192
O	-1.727488	-4.576913	-7.797648
N	-3.640310	-9.040782	-2.875695
O	-4.745861	-8.982599	-3.453991
O	-3.257086	-8.039821	-2.195631
N	-6.021861	7.679194	-2.889527
O	-5.427114	8.614401	-3.464019
O	-5.339116	6.848575	-2.213652
N	-3.575022	4.948044	-7.902222
O	-3.095598	3.791047	-7.795273
O	-2.868961	5.897641	-8.259967
C	-11.474570	-3.819798	7.056646
C	-10.642012	-4.057533	8.115255
C	8.849338	-7.162794	8.111175
C	9.059190	-8.006124	7.055176
C	-4.190328	-11.784369	-6.544966
C	-5.351726	-11.140320	-6.871741
C	1.792816	11.237246	8.121629
C	2.415437	11.841561	7.064532
C	-8.111293	9.507994	-6.571380
C	-6.973260	10.194018	-6.894827
C	12.318634	0.933936	-6.887003
C	12.296603	2.262023	-6.561261
H	-9.090265	9.982306	-6.671236
H	-7.032343	11.221742	-7.260706
H	2.591185	12.919827	7.069204

H	1.460916	11.827268	8.978970
H	9.528927	-7.167725	8.966427
H	9.906003	-8.696389	7.058699
H	13.198631	2.870439	-6.658765
H	13.237504	0.469467	-7.252414
H	-4.114853	-12.870133	-6.638524
H	-6.213959	-11.704975	-7.234091
H	-10.989651	-4.638170	8.972766
H	-12.497136	-4.204394	7.060267

330

CAGEphen+NO3Ni SCF Done: -19922.2005024 A.U.

N	3.854140	-3.297151	-3.345170
N	3.609769	-4.584607	-1.382599
C	3.291389	-4.290297	-2.648111
C	4.803120	-2.597412	-2.713062
N	5.213120	-2.854368	-1.466157
C	4.574867	-3.842066	-0.829051
C	4.926737	-4.097225	0.593800
C	5.992260	-3.423661	1.206417
C	4.165134	-4.981513	1.370860
C	6.252825	-3.637294	2.557657
C	4.462819	-5.133589	2.721389
N	5.485151	-4.467648	3.293989
H	6.606996	-2.722914	0.642390
H	3.332441	-5.531959	0.934569
H	7.050305	-3.098268	3.084872
H	3.864674	-5.796793	3.354126
C	5.398997	-1.430075	-3.416284
C	6.482508	-0.731299	-2.866847
C	4.847500	-0.963570	-4.617895
C	6.963172	0.405476	-3.511720
C	5.357695	0.192910	-5.199301
N	6.390213	0.859394	-4.645903
H	6.939536	-1.055420	-1.932444
H	4.009387	-1.480155	-5.084401
H	7.780826	1.005320	-3.092261
H	4.921386	0.595473	-6.119073
C	2.227547	-5.095145	-3.307669
C	1.524499	-6.075718	-2.592430
C	1.885492	-4.875617	-4.650048
C	0.497340	-6.775409	-3.218962
C	0.855164	-5.616492	-5.224194
N	0.177761	-6.533274	-4.506152
H	1.763937	-6.282645	-1.549940
H	2.410557	-4.127001	-5.242579
H	-0.104584	-7.537025	-2.696991
H	0.540268	-5.468809	-6.263777
Ni	-1.331724	-7.415843	-5.297634
N	-4.778961	-1.697715	-3.355192
N	-5.077690	-3.091620	-1.473338
C	-4.648049	-2.867832	-2.720015
C	-5.360399	-0.712752	-2.661901
N	-5.777396	-0.838680	-1.397090

C	-5.616361	-2.044060	-0.839934
C	-6.014239	-2.217495	0.583175
C	-6.407445	-1.114994	1.354983
C	-5.955594	-3.473844	1.201761
C	-6.271422	-3.588534	2.553234
N	-6.615867	-2.507742	3.284155
H	-6.473627	-0.120926	0.914369
H	-5.649298	-4.357130	0.642224
H	-6.969703	-0.441394	3.334175
H	-6.196358	-4.545326	3.086340
C	-5.524031	0.609575	-3.324689
C	-6.028441	1.708815	-2.614283
C	-5.153485	0.794747	-4.664756
C	-6.117926	2.947265	-3.243072
C	-5.276632	2.056747	-5.241187
N	-5.738469	3.101807	-4.527430
H	-6.334497	1.605594	-1.573794
H	-4.762145	-0.034590	-5.253193
H	-6.481170	3.850161	-2.726037
H	-4.982194	2.255805	-6.278367
C	-3.933459	-3.969132	-3.419098
C	-3.873449	-5.255935	-2.867330
C	-3.249150	-3.727405	-4.618713
C	-3.128157	-6.242501	-3.507863
C	-2.501685	-4.749278	-5.195655
N	-2.444159	-5.975857	-4.640032
H	-4.386208	-5.487441	-1.934289
H	-3.274446	-2.743934	-5.086700
H	-3.020911	-7.249830	-3.086212
H	-1.930953	-4.575044	-6.113430
Ni	-5.741976	4.849473	-5.319333
Ni	5.907904	-4.539953	5.181236
Ni	7.081043	2.539648	-5.311366
N	1.255556	0.532500	5.788102
N	-1.085637	0.832208	5.784356
C	0.165124	1.306928	5.782262
C	1.041233	-0.788065	5.786158
N	-0.174691	-1.345267	5.788720
C	-1.211156	-0.499354	5.782814
C	-2.583142	-1.072886	5.729551
C	-3.713441	-0.245588	5.801846
C	-2.775735	-2.452250	5.563058
C	-4.982295	-0.808137	5.686084
C	-4.068836	-2.952527	5.440716
N	-5.137492	-2.132652	5.494939
H	-3.607540	0.830707	5.934264
H	-1.924437	-3.129819	5.506227
H	-5.894421	-0.200709	5.718395
H	-4.278583	-4.022106	5.277604
C	2.224486	-1.688883	5.735469
C	2.075161	-3.081190	5.814995
C	3.514257	-1.165292	5.563436
C	3.197619	-3.897864	5.700167

C	4.594812	-2.034077	5.441986
N	4.421138	-3.369598	5.502741
H	1.091051	-3.528332	5.951802
H	3.673939	-0.089353	5.500860
H	3.128983	-4.991385	5.737111
H	5.624488	-1.679427	5.273134
C	0.353587	2.781942	5.728257
C	-0.744990	3.636835	5.556122
C	1.634230	3.348822	5.805406
C	-0.532932	5.006883	5.432825
C	1.780195	4.729065	5.688394
N	0.710632	5.523999	5.491435
H	-1.756735	3.237088	5.495710
H	2.513615	2.720306	5.942377
H	-1.354498	5.722126	5.265335
H	2.761604	5.216528	5.723586
Ni	0.980135	7.395639	5.166756
N	2.166808	5.417269	-1.395521
N	-0.135226	5.932886	-1.475317
C	1.040799	5.879336	-0.840608
C	2.069565	4.991057	-2.659853
N	0.926954	4.978146	-3.354939
C	-0.153634	5.448270	-2.721778
C	-1.463195	5.377613	-3.423379
C	-2.611986	5.958613	-2.869448
C	-1.589807	4.672554	-4.628564
C	-6.688869	-1.292509	2.705972
C	-2.846250	4.535133	-5.210250
N	-3.941220	5.089229	-4.653032
H	-2.561141	6.511172	-1.931826
H	-0.722080	4.210335	-5.097943
H	-2.974889	3.960782	-6.133453
C	3.297600	4.471292	-3.320425
C	4.500234	4.356497	-2.607817
C	3.275114	4.059368	-4.661021
C	5.618201	3.814156	-3.234948
C	4.430231	3.534601	-5.235772
N	5.564587	3.409509	-4.519799
H	4.562368	4.672197	-1.566943
H	2.362614	4.136789	-5.251398
H	6.580736	3.676763	-2.716003
H	4.456754	3.182414	-6.273675
C	1.086723	6.314657	0.581342
C	-0.032528	6.892898	1.195679
C	2.236741	6.107819	1.356150
C	0.022636	7.227704	2.546219
C	2.220461	6.444513	2.706165
N	1.129396	6.988874	3.280415
H	-0.949516	7.066677	0.633571
H	3.132279	5.668115	0.918608
H	-0.845408	7.641314	3.076052
H	3.097092	6.265712	3.336601
Ni	-6.893850	-2.833602	5.171886

C	-3.836778	5.805099	-3.513971
H	-4.767179	6.205646	-3.090650
C	1.260303	12.081257	4.787055
C	1.797926	11.321161	3.763193
C	1.745465	9.915509	3.834226
N	1.190200	9.289930	4.862634
C	0.707889	10.021586	5.896016
C	0.697504	11.431622	5.909233
H	1.276013	13.173091	4.742275
H	2.262334	11.789038	2.894117
H	2.170462	9.296206	3.047275
C	-0.773920	9.142948	9.195409
C	-0.321276	9.951163	8.129714
C	0.223973	9.283608	7.013704
N	0.334197	7.933889	6.928195
C	-0.081329	7.197004	7.949470
C	-0.642776	7.768799	9.105197
H	-1.219550	9.606765	10.078726
H	0.023927	6.116733	7.855954
H	-0.979658	7.114388	9.910328
C	8.304750	-3.875366	9.203149
C	7.047309	-3.305651	9.113981
C	6.268848	-3.512778	7.961165
N	6.698559	-4.243830	6.941543
C	7.924528	-4.819358	7.026074
C	8.777707	-4.674434	8.139398
H	8.931108	-3.716099	10.084210
H	6.649457	-2.684653	9.917760
H	5.279049	-3.067132	7.868577
C	9.827422	-7.123053	4.805507
C	9.548327	-6.305182	5.924111
C	8.320790	-5.611309	5.910807
N	7.443369	-5.669705	4.880056
C	7.705251	-6.468970	3.855324
C	8.897195	-7.215982	3.785096
H	10.765928	-7.681233	4.760838
H	6.953904	-6.532877	3.071214
H	9.068383	-7.856994	2.919253
C	-4.786224	-10.068718	-7.086520
C	-3.517464	-10.587197	-6.740433
C	-2.572070	-9.667530	-6.241576
N	-2.839663	-8.352705	-6.053492
C	-4.023090	-7.877537	-6.415256
C	-5.027746	-8.714046	-6.939528
H	-5.558766	-10.736765	-7.475440
H	-4.184873	-6.808038	-6.299364
H	-5.986568	-8.275903	-7.219844
C	0.459221	-11.769464	-5.744392
C	1.323129	-10.768191	-5.338666
C	0.857230	-9.445083	-5.237074
N	-0.395673	-9.114520	-5.519263
C	-1.246967	-10.087364	-5.931915
C	-0.874957	-11.440501	-6.069511

H	0.797380	-12.805916	-5.817733
H	2.360676	-10.985959	-5.081706
H	1.520079	-8.643443	-4.913257
C	-7.534678	-5.215296	9.206427
C	-8.460488	-5.228842	8.140425
C	-8.153717	-4.425715	7.022698
N	-7.039383	-3.656202	6.936052
C	-6.193844	-3.646283	7.957729
C	-6.409375	-4.415900	9.114884
H	-7.714348	-5.831100	10.090993
H	-5.310620	-3.015493	7.863506
H	-5.674436	-4.379517	9.920196
C	-11.094470	-4.929041	4.796160
C	-10.703936	-4.085763	3.770738
C	-9.459613	-3.429719	3.840571
N	-8.640461	-3.596970	4.869366
C	-9.034125	-4.377687	5.904376
C	-10.250871	-5.090326	5.918804
H	-12.048467	-5.460369	4.752204
H	-11.341123	-3.918365	2.901342
H	-9.134775	-2.754077	3.052297
C	11.099141	0.864955	-7.106141
C	10.047096	-0.020621	-6.951133
C	8.822341	0.433969	-6.424728
N	8.643494	1.698199	-6.068616
C	9.647987	2.586163	-6.264160
C	10.915297	2.224636	-6.765860
H	12.062405	0.527900	-7.497134
H	10.145863	-1.071385	-7.226853
H	7.977325	-0.240147	-6.302457
C	9.954814	6.264675	-5.786869
C	10.335771	4.943061	-6.106286
C	9.350288	3.945166	-5.960070
N	8.083679	4.198173	-5.544108
C	7.744494	5.450051	-5.267631
C	8.657089	6.514451	-5.378087
H	10.683122	7.075303	-5.866924
H	6.719817	5.625181	-4.941716
H	8.327783	7.523304	-5.125619
C	-10.403474	5.476273	-5.807605
C	-9.972076	4.227930	-5.396230
C	-8.594369	3.969949	-5.281206
N	-7.679012	4.889516	-5.555645
C	-8.091987	6.112512	-5.974009
C	-9.448517	6.466838	-6.124672
H	-11.469420	5.701482	-5.891225
H	-10.681904	3.438512	-5.145361
H	-8.234679	2.995408	-4.953001
C	-6.295532	9.166965	-7.115379
C	-7.382038	8.327925	-6.778216
C	-7.062943	7.049802	-6.275182
N	-5.792315	6.623885	-6.075670
C	-4.785890	7.410804	-6.429359

C	-5.003032	8.698662	-6.956588
H	-6.484139	10.169654	-7.507042
H	-3.779816	7.016360	-6.304093
H	-4.141635	9.309535	-7.229921
O	-2.565896	-6.043427	-8.250183
O	-0.940560	-10.403515	-2.964055
O	9.487574	4.380973	-2.998916
O	6.412840	2.834556	-7.570348
O	3.150387	7.523951	6.121030
O	-2.521197	8.222319	6.431938
O	-5.666240	4.123463	-7.580693
O	-8.546986	6.011001	-3.011410
O	4.930309	-6.478339	6.131913
O	8.373628	-1.916104	6.432096
O	-9.383133	-0.514592	4.446755
O	-5.874172	-6.281660	6.443116
N	-8.395909	-0.261160	5.146601
O	-7.639551	0.703980	4.871588
O	-8.087023	-1.012924	6.120175
N	-6.089142	-6.028157	5.249162
O	-5.157609	-5.555479	4.526633
O	-7.216127	-6.193456	4.739028
N	-2.191602	8.288090	5.238901
O	-2.243411	7.247942	4.511767
O	-1.772871	9.350001	4.734644
N	3.958601	7.413836	5.149832
O	4.418811	6.276157	4.879540
O	4.232447	8.393707	4.447346
N	4.430644	-7.126567	5.162854
O	5.141862	-7.857540	4.464133
O	3.215734	-6.955877	4.891148
N	8.258357	-2.231393	5.239069
O	7.385382	-1.659826	4.515853
O	8.960230	-3.130295	4.730701
N	9.222821	3.175294	-2.889591
O	9.906570	2.302171	-3.463718
O	8.215012	2.808464	-2.210185
N	5.838899	1.753275	-7.901466
O	4.588600	1.681742	-7.795665
O	6.503683	0.773214	-8.256468
N	-1.385903	-5.955404	-7.892136
O	-0.737052	-6.991290	-7.553569
O	-0.823329	-4.836246	-7.790857
N	-1.852932	-9.571284	-2.861100
O	-2.947734	-9.727945	-3.441535
O	-1.671119	-8.514140	-2.182296
N	-7.372117	6.390092	-2.903278
O	-6.963105	7.420861	-3.476340
O	-6.546691	5.702980	-2.225276
N	-4.442667	4.168866	-7.910598
O	-3.753558	3.123333	-7.800898
O	-3.928029	5.234521	-8.268068
C	-10.542291	-5.915905	7.059579

C	-9.681111	-5.988672	8.119472
C	10.048203	-5.347532	8.116768
C	10.413602	-6.134989	7.060003
C	-1.864373	-12.375727	-6.532297
C	-3.126870	-11.965855	-6.861727
C	-0.370299	11.388096	8.107562
C	0.124999	12.097148	7.048192
C	-9.759349	7.790867	-6.591659
C	-8.770088	8.678970	-6.912541
C	11.913677	3.251439	-6.895708
C	11.638683	4.551392	-6.571835
H	-10.809863	8.072298	-6.694360
H	-9.020820	9.677066	-7.279309
H	0.093399	13.189193	7.050635
H	-0.806498	11.906423	8.964527
H	10.717143	-5.223616	8.971453
H	11.376237	-6.651613	7.062340
H	12.407847	5.320705	-6.671181
H	12.904030	2.970417	-7.261504
H	-1.582366	-13.427009	-6.625096
H	-3.864417	-12.685161	-7.225329
H	-9.912786	-6.623400	8.977822
H	-11.472849	-6.488282	7.062968

330

CAGEphen+NO3Pd SCF Done: -11640.5697178 A.U.

N	4.406290	1.569853	-4.082227
N	4.721636	-0.765660	-3.956946
C	4.206600	0.326313	-4.532713
C	5.159895	1.685559	-2.983182
N	5.712337	0.646907	-2.346679
C	5.463328	-0.561795	-2.862633
C	5.999266	-1.752282	-2.147948
C	6.741306	-1.606355	-0.966864
C	5.729123	-3.048550	-2.610057
C	7.155970	-2.739345	-0.271789
C	6.168918	-4.145697	-1.874097
N	6.852946	-3.974815	-0.723078
H	6.977158	-0.616989	-0.576542
H	5.160535	-3.205573	-3.525961
H	7.707671	-2.678121	0.679357
H	5.949952	-5.182449	-2.174023
C	5.351361	3.043597	-2.404977
C	6.087935	3.223862	-1.225238
C	4.754860	4.166322	-2.997038
C	6.177528	4.492525	-0.658477
C	4.877713	5.411641	-2.386412
N	5.565174	5.549383	-1.232965
H	6.571168	2.378715	-0.736307
H	4.178558	4.070313	-3.916532
H	6.711133	4.679454	0.286678
H	4.396861	6.316276	-2.790653
C	3.317298	0.142974	-5.712038
C	3.027764	-1.139543	-6.200024

C	2.706927	1.244803	-6.328737
C	2.124666	-1.287514	-7.249477
C	1.811863	1.037609	-7.375003
N	1.527152	-0.210772	-7.802141
H	3.484897	-2.021422	-5.752636
H	2.908928	2.258159	-5.983604
H	1.835699	-2.274387	-7.643699
H	1.278221	1.865904	-7.866965
Pd	0.121239	-0.478304	-9.270877
N	-4.617509	0.346991	-4.137921
N	-4.299935	-1.988229	-4.012653
C	-4.088260	-0.797544	-4.584170
C	-5.386637	0.255861	-3.047365
N	-5.648865	-0.893466	-2.415308
C	-5.081054	-1.991223	-2.926845
C	-5.287948	-3.282764	-2.216664
C	-6.054673	-3.342198	-1.043858
C	-4.677147	-4.459197	-2.674657
C	-4.816772	-5.635453	-1.942779
N	-5.534075	-5.655325	-0.799499
H	-6.549774	-2.452230	-0.657025
H	-4.077350	-4.457048	-3.584094
H	-6.718202	-4.636264	0.592756
H	-4.326609	-6.576022	-2.239411
C	-5.939851	1.512730	-2.473375
C	-6.708417	1.488376	-1.300573
C	-5.660317	2.754628	-3.062030
C	-7.139460	2.686305	-0.736696
C	-6.117653	3.921073	-2.454571
N	-6.827227	3.868900	-1.307476
H	-6.952301	0.544354	-0.814616
H	-5.071126	2.817109	-3.976241
H	-7.712569	2.722551	0.202904
H	-5.892544	4.921983	-2.855354
C	-3.168672	-0.735332	-5.752834
C	-2.543760	-1.893893	-6.236661
C	-2.864755	0.490603	-6.362390
C	-1.621278	-1.794218	-7.274850
C	-1.934206	0.531099	-7.397393
N	-1.323843	-0.596020	-7.820549
H	-2.755941	-2.866614	-5.794367
H	-3.332139	1.413219	-6.020129
H	-1.075655	-2.667904	-7.664517
H	-1.633284	1.472753	-7.882810
Pd	-7.384802	5.614945	-0.387511
Pd	7.386176	-5.614043	0.387890
Pd	5.628231	7.378268	-0.306926
N	1.726324	-4.136922	4.294982
N	-0.612060	-4.455377	4.279986
C	0.500890	-3.906379	4.779076
C	1.800111	-4.959901	3.243077
N	0.738106	-5.550285	2.683797
C	-0.450018	-5.266304	3.228646

C	-1.666990	-5.844472	2.596227
C	-2.944336	-5.542995	3.090514
C	-1.566894	-6.660149	1.459767
C	-4.069189	-6.026245	2.427308
C	-2.726015	-7.115538	0.836945
N	-3.943025	-6.781910	1.316015
H	-3.065682	-4.917178	3.973937
H	-0.593407	-6.922373	1.046977
H	-5.093591	-5.786704	2.753016
H	-2.702078	-7.726334	-0.079128
C	3.135044	-5.191084	2.626852
C	3.270251	-6.003110	1.491400
C	4.279395	-4.559812	3.135595
C	4.516417	-6.132261	0.883817
C	5.500473	-4.724694	2.487101
N	5.594194	-5.486063	1.376484
H	2.407218	-6.515499	1.067777
H	4.218491	-3.924906	4.018776
H	4.667770	-6.726875	-0.030794
H	6.419720	-4.220488	2.824233
C	0.362106	-2.942590	5.904740
C	-0.901285	-2.619350	6.420670
C	1.486158	-2.296074	6.438990
C	-1.010077	-1.649852	7.414136
C	1.318126	-1.334825	7.432259
N	0.086523	-1.020250	7.886346
H	-1.799219	-3.102372	6.036977
H	2.485902	-2.522201	6.069932
H	-1.981713	-1.333440	7.824857
H	2.164106	-0.772391	7.857609
Pd	-0.126072	0.478594	9.269583
N	0.506731	4.871374	3.811806
N	-1.832005	4.555367	3.797135
C	-0.620469	4.376630	4.335088
C	0.374660	5.568804	2.678084
N	-0.797831	5.794132	2.075008
C	-1.875787	5.264625	2.663906
C	-3.193390	5.429989	1.991718
C	-4.352816	4.857294	2.534583
C	-3.296161	6.116624	0.773196
C	-6.160022	-4.546173	-0.352373
C	-4.525610	6.181280	0.122752
N	-5.618076	5.592156	0.653020
H	-4.317097	4.319908	3.481565
H	-2.420620	6.580440	0.320296
H	-4.650930	6.675870	-0.853167
C	1.609213	6.079550	2.022148
C	2.872543	5.830961	2.578113
C	1.540607	6.774829	0.806106
C	4.015399	6.244935	1.898964
C	2.716538	7.165318	0.170904
N	3.919835	6.883321	0.713480
H	2.969425	5.298796	3.523757

H	0.578809	6.992902	0.343272
H	5.030516	6.041011	2.274341
H	2.717912	7.679427	-0.803040
C	-0.514446	3.534518	5.557725
C	-1.653993	2.944812	6.123873
C	0.733460	3.268046	6.139615
C	-1.515396	2.091098	7.215073
C	0.812735	2.406294	7.230435
N	-0.297608	1.826906	7.733779
H	-2.642610	3.130061	5.705359
H	1.642558	3.710346	5.733738
H	-2.373810	1.573829	7.671588
H	1.771750	2.135257	7.699084
Pd	-5.624487	-7.380286	0.305820
C	-5.555983	4.953315	1.840444
H	-6.485731	4.488501	2.204621
C	-0.554326	3.489182	13.006793
C	-0.619506	4.030321	11.736352
C	-0.499674	3.187173	10.615621
N	-0.325244	1.881923	10.742206
C	-0.258131	1.328223	11.979589
C	-0.368213	2.095690	13.160370
H	-0.644716	4.123857	13.891688
H	-0.762171	5.100686	11.582117
H	-0.547043	3.586551	9.603136
C	0.197136	-2.129871	13.307649
C	0.009410	-0.728172	13.311571
C	-0.068574	-0.086897	12.055368
N	0.029916	-0.765362	10.883942
C	0.205353	-2.076548	10.897454
C	0.294418	-2.799305	12.102027
H	0.263406	-2.669641	14.255424
H	0.278547	-2.578315	9.933227
H	0.439247	-3.879731	12.062957
C	9.240776	-9.458267	2.623295
C	7.987156	-9.029542	3.017957
C	7.407237	-7.910226	2.391762
N	8.030511	-7.250747	1.429073
C	9.262124	-7.653314	1.025091
C	9.921275	-8.764743	1.595716
H	9.711020	-10.324621	3.094802
H	7.434872	-9.541267	3.807225
H	6.420979	-7.550411	2.682858
C	11.724630	-6.478226	-1.521317
C	11.169525	-7.267169	-0.487185
C	9.887659	-6.902845	-0.018729
N	9.200778	-5.846708	-0.523650
C	9.734030	-5.118609	-1.490958
C	11.006159	-5.407432	-2.019676
H	12.714030	-6.721720	-1.916057
H	9.145919	-4.281103	-1.864639
H	11.404594	-4.778532	-2.816893
C	-2.640018	-1.052460	-13.177235

C	-1.239541	-0.868574	-13.246791
C	-0.550498	-0.712567	-12.023594
N	-1.183484	-0.734822	-10.823165
C	-2.494189	-0.907868	-10.775208
C	-3.262634	-1.071599	-11.943095
H	-3.215791	-1.177450	-14.097498
H	-2.958492	-0.918698	-9.789731
H	-4.340732	-1.211255	-11.853531
C	2.986460	-0.297798	-13.140281
C	3.576106	-0.154115	-11.898175
C	2.776645	-0.200568	-10.740591
N	1.467372	-0.378800	-10.805764
C	0.866488	-0.522361	-12.014287
C	1.588050	-0.489400	-13.228224
H	3.586675	-0.265216	-14.052805
H	4.651670	-0.005013	-11.794460
H	3.214655	-0.090531	-9.749157
C	-6.417208	-11.583704	2.523194
C	-7.245740	-11.093449	1.487261
C	-6.898793	-9.845736	0.923163
N	-5.822720	-9.131652	1.340725
C	-5.057581	-9.604222	2.310996
C	-5.326777	-10.838556	2.931678
H	-6.646031	-12.544736	2.990185
H	-4.205357	-8.996373	2.612840
H	-4.667372	-11.187092	3.727621
C	-9.556053	-9.360923	-1.652299
C	-9.141928	-8.136865	-2.143520
C	-7.998178	-7.521841	-1.600513
N	-7.301725	-8.084264	-0.626439
C	-7.689358	-9.285887	-0.128364
C	-8.823220	-9.976337	-0.611058
H	-10.440860	-9.857421	-2.058039
H	-9.683924	-7.634632	-2.945906
H	-7.649299	-6.557570	-1.968344
C	9.613995	9.155652	-2.348550
C	9.210817	7.888099	-2.725234
C	8.051706	7.329900	-2.154110
N	7.330541	7.986828	-1.260516
C	7.706862	9.232779	-0.875651
C	8.854565	9.872128	-1.394652
H	10.510353	9.609518	-2.778168
H	9.773133	7.308204	-3.458272
H	7.710776	6.333354	-2.432553
C	6.367570	11.784974	1.495343
C	7.223033	11.193530	0.537038
C	6.889216	9.895007	0.092341
N	5.800912	9.225695	0.550354
C	5.010477	9.793005	1.446780
C	5.265008	11.083868	1.946964
H	6.585429	12.788371	1.869178
H	4.149115	9.218003	1.784896
H	4.584808	11.510337	2.685390

C	-11.677829	6.265417	-2.475454
C	-10.950220	5.147681	-2.839336
C	-9.690669	4.918500	-2.254302
N	-9.177702	5.747241	-1.359773
C	-9.874110	6.850858	-0.986709
C	-11.144610	7.161604	-1.520195
H	-12.657821	6.464160	-2.916066
H	-11.331975	4.436033	-3.572576
H	-9.096067	4.045688	-2.521787
C	-9.282436	9.676061	1.379117
C	-9.940829	8.875578	0.416977
C	-9.271129	7.709455	-0.015468
N	-8.049906	7.353145	0.457679
C	-7.447139	8.113007	1.357409
C	-8.038930	9.292957	1.846086
H	-9.761533	10.587954	1.743725
H	-6.468080	7.787629	1.707365
H	-7.503518	9.887191	2.587735
O	-1.333794	2.157259	-10.435570
O	1.597740	-3.217185	-10.122885
O	7.204720	7.566701	2.501104
O	4.036055	9.808408	-1.709291
O	2.839688	-0.151892	10.365345
O	-2.833517	-0.935548	10.314381
O	-7.399603	7.101027	-3.244192
O	-9.899112	4.046235	0.904494
O	6.525199	-8.509412	-0.735306
O	8.916205	-5.083105	3.177807
O	-8.477743	-6.431305	1.478742
O	-4.000378	-9.939560	-0.798807
N	-7.593693	-6.325272	2.347741
O	-6.965587	-5.225726	2.462319
O	-7.276769	-7.283694	3.074986
N	-4.167458	-9.105847	-1.706983
O	-3.279626	-8.217302	-1.903941
O	-5.203564	-9.091329	-2.395566
N	-3.101196	0.093357	9.668108
O	-3.311044	0.002667	8.417404
O	-3.121213	1.214587	10.206990
N	2.835548	0.907553	9.713190
O	3.080451	0.869122	8.466184
O	2.550441	1.997076	10.242002
N	6.467756	-7.659957	-1.642586
O	7.467004	-7.364146	-2.322195
O	5.374321	-7.044346	-1.847697
N	8.977310	-4.075329	2.450672
O	8.079222	-3.181193	2.553688
O	9.868807	-3.944112	1.592785
N	7.540946	6.540438	1.883336
O	8.448377	6.560229	1.032351
O	6.909838	5.456558	2.091665
N	4.226743	8.886729	-2.523042
O	3.343280	7.981520	-2.652737

O	5.281477	8.803392	-3.177649
N	-0.279932	2.464191	-9.849819
O	0.818626	2.446943	-10.433589
O	-0.320557	2.755944	-8.612982
N	0.530234	-3.464807	-9.533748
O	-0.554283	-3.509528	-10.141773
O	0.541322	-3.627962	-8.272879
N	-9.038427	4.285866	1.770384
O	-9.011957	5.369990	2.380097
O	-8.135569	3.421303	2.002500
N	-6.405581	7.451482	-2.583208
O	-5.317564	6.805734	-2.709300
O	-6.463576	8.388464	-1.766647
C	-9.158965	-11.244465	-0.023485
C	-8.403198	-11.779679	0.981777
C	11.225011	-9.118773	1.104725
C	11.823056	-8.401281	0.106825
C	0.867969	-0.650139	-14.461825
C	-0.486697	-0.831731	-14.470723
C	-0.104995	0.070966	14.500863
C	-0.285718	1.423861	14.428419
C	-11.809285	8.352678	-1.066646
C	-11.232461	9.173956	-0.138727
C	9.176664	11.193277	-0.928876
C	8.395059	11.826319	-0.003386
H	-12.791564	8.589454	-1.481819
H	-11.747048	10.076627	0.198451
H	-0.371294	2.021005	15.339137
H	-0.044044	-0.428790	15.470317
H	11.731303	-9.978937	1.548676
H	12.814232	-8.679717	-0.258342
H	8.649830	12.830408	0.343314
H	10.065146	11.684111	-1.332593
H	1.429698	-0.623987	-15.398350
H	-1.023343	-0.952844	-15.414464
H	-8.668092	-12.744251	1.420792
H	-10.036622	-11.775104	-0.399574

330

CAGEphen+NO3Zn SCF Done: -21548.5317263 A.U.

N	2.232503	-4.187429	3.811362
N	4.027897	-2.657578	3.884157
C	2.893033	-3.168993	4.373606
C	2.774593	-4.703637	2.702698
N	3.896908	-4.242763	2.139368
C	4.487631	-3.213080	2.757151
C	5.712061	-2.634909	2.141286
C	6.165980	-3.081948	0.891291
C	6.428035	-1.613563	2.781660
C	7.288098	-2.479691	0.327204
C	7.544972	-1.067180	2.149899
N	7.954102	-1.487984	0.941276
H	5.640725	-3.879345	0.366201
H	6.113647	-1.246655	3.758340

H	7.683274	-2.775858	-0.647560
H	8.147202	-0.278540	2.608803
C	2.081697	-5.850127	2.055366
C	2.646810	-6.508370	0.954015
C	0.841705	-6.299957	2.533353
C	1.956813	-7.570319	0.368953
C	0.216328	-7.364466	1.889049
N	0.761839	-7.979524	0.826938
H	3.614143	-6.196701	0.560719
H	0.374581	-5.819865	3.392674
H	2.358983	-8.130885	-0.479167
H	-0.751421	-7.756479	2.211242
C	2.320539	-2.559007	5.603737
C	2.994277	-1.534609	6.283703
C	1.081999	-2.986778	6.105103
C	2.406092	-0.968147	7.414641
C	0.562497	-2.365789	7.238048
N	1.208868	-1.372897	7.870735
H	3.963485	-1.182056	5.931834
H	0.532897	-3.786955	5.609802
H	2.892645	-0.176657	7.991035
H	-0.400533	-2.648022	7.670407
Zn	0.170728	-0.261881	9.459099
N	-3.863451	2.400072	4.128576
N	-2.098977	3.966237	4.105097
C	-2.719861	2.896031	4.613204
C	-4.390354	3.045300	3.081361
N	-3.849266	4.138232	2.530860
C	-2.699520	4.561399	3.068180
C	-2.038551	5.752020	2.469157
C	-2.642642	6.466954	1.425349
C	-0.784433	6.180820	2.929408
C	-0.182692	7.279727	2.321742
N	-0.763729	7.946952	1.311223
H	-3.621004	6.171782	1.046926
H	-0.287448	5.656683	3.745099
H	-2.404141	8.161967	0.069003
H	0.795509	7.656252	2.630552
C	-5.636046	2.498846	2.479325
C	-6.288026	3.167903	1.433825
C	-6.175211	1.284472	2.929970
C	-7.428526	2.597341	0.868600
C	-7.315205	0.781200	2.308178
N	-7.919708	1.421215	1.293787
H	-5.906558	4.119022	1.063221
H	-5.701347	0.739129	3.745669
H	-7.984056	3.081200	0.060782
H	-7.775184	-0.164132	2.606198
C	-2.095574	2.212359	5.777740
C	-0.837980	2.611939	6.254165
C	-2.744801	1.152065	6.425757
C	-0.278606	1.932113	7.333277
C	-2.117186	0.527274	7.503441

N	-0.904224	0.908653	7.937478
H	-0.306282	3.437902	5.782716
H	-3.728230	0.820588	6.093370
H	0.699294	2.192388	7.745604
H	-2.584908	-0.291965	8.056105
Zn	-9.518742	0.397390	0.182165
Zn	9.518946	-0.399101	-0.156293
Zn	-0.413472	-9.486801	-0.265368
N	4.069622	2.283749	-3.999643
N	2.472382	4.019725	-3.933576
C	2.996237	2.918742	-4.483408
C	4.620194	2.809002	-2.899178
N	4.158977	3.905400	-2.286201
C	3.082425	4.479367	-2.835301
C	2.517656	5.688384	-2.177952
C	1.308503	6.244782	-2.621517
C	3.169420	6.293599	-1.094194
C	0.794537	7.356242	-1.958175
C	2.589335	7.408302	-0.488664
N	1.419692	7.917641	-0.910310
H	0.776502	5.807120	-3.465765
H	4.117018	5.898577	-0.728684
H	-0.146512	7.828580	-2.250401
H	3.061368	7.929598	0.348406
C	5.803067	2.122575	-2.313822
C	6.334649	2.531602	-1.081604
C	6.407738	1.042471	-2.972334
C	7.421819	1.838957	-0.553499
C	7.496977	0.406637	-2.377142
N	7.982615	0.795246	-1.186222
H	5.897049	3.372002	-0.543304
H	6.031959	0.703288	-3.937296
H	7.874917	2.104005	0.404906
H	8.016936	-0.428544	-2.853840
C	2.333975	2.357699	-5.691360
C	1.280302	3.039015	-6.316926
C	2.738212	1.124710	-6.224646
C	0.662621	2.463475	-7.426864
C	2.064770	0.617090	-7.333249
N	1.044367	1.270490	-7.912838
H	0.946555	4.005537	-5.940295
H	3.558861	0.569498	-5.771007
H	-0.152832	2.957041	-7.962262
H	2.325284	-0.341937	-7.787644
Zn	-0.145734	0.257393	-9.459670
N	-2.602457	-3.803372	-4.058696
N	-4.159394	-2.034221	-3.945161
C	-3.115692	-2.653160	-4.508090
C	-3.199957	-4.339164	-2.988251
N	-4.264733	-3.801040	-2.382817
C	-4.708809	-2.644730	-2.888657
C	-5.870511	-1.989562	-2.229333
C	-6.317328	-0.728882	-2.653043

C	-6.540421	-2.607603	-1.163967
C	-1.973779	7.559998	0.873852
C	-7.608536	-1.946461	-0.557214
N	-8.013268	-0.730575	-0.960987
H	-5.828367	-0.220754	-3.483624
H	-6.230376	-3.591289	-0.812239
H	-8.176466	-2.388294	0.265925
C	-2.628325	-5.591740	-2.425316
C	-1.440422	-6.130019	-2.942652
C	-3.244636	-6.250986	-1.352389
C	-0.908786	-7.276141	-2.357146
C	-2.648566	-7.397372	-0.826642
N	-1.497049	-7.887337	-1.315802
H	-0.936583	-5.650476	-3.781357
H	-4.174334	-5.870363	-0.930095
H	0.019081	-7.735241	-2.706882
H	-3.091461	-7.958594	0.000497
C	-2.481123	-2.021057	-5.695975
C	-2.890833	-0.755754	-6.142316
C	-1.455811	-2.672161	-6.396351
C	-2.253867	-0.191137	-7.244862
C	-0.873442	-2.039078	-7.494146
N	-1.262988	-0.818781	-7.899285
H	-3.690889	-0.222201	-5.629914
H	-1.118195	-3.661338	-6.088287
H	-2.523447	0.792946	-7.635938
H	-0.082911	-2.507900	-8.086188
Zn	0.388772	9.484690	0.240109
C	-7.388937	-0.135434	-1.990563
H	-7.778139	0.846683	-2.269820
C	-2.127076	-1.775219	-13.539723
C	-2.518663	-2.276374	-12.311515
C	-1.971961	-1.711389	-11.143337
N	-1.101544	-0.716034	-11.176551
C	-0.698517	-0.219505	-12.365206
C	-1.190490	-0.717443	-13.598065
H	-2.528454	-2.190092	-14.468028
H	-3.231746	-3.098625	-12.233713
H	-2.247099	-2.083148	-10.153557
C	1.653144	2.451586	-13.539243
C	0.713971	1.396114	-13.598166
C	0.275438	0.850117	-12.365522
N	0.730150	1.300200	-11.177065
C	1.601978	2.294286	-11.142668
C	2.097996	2.904830	-12.310555
H	2.014440	2.902541	-14.467186
H	1.919933	2.627153	-10.151889
H	2.814635	3.723884	-12.231746
C	13.610543	1.340632	-2.380201
C	12.384680	1.786420	-2.840083
C	11.213691	1.306781	-2.222409
N	11.242424	0.449510	-1.215594
C	12.428045	-0.006019	-0.758867

C	13.663490	0.416713	-1.311056
H	14.540769	1.689451	-2.836256
H	12.310363	2.491394	-3.669621
H	10.225089	1.626190	-2.560258
C	13.587069	-2.377271	1.901435
C	13.651380	-1.456602	0.830100
C	12.421695	-0.964233	0.324834
N	11.230539	-1.352516	0.827008
C	11.191719	-2.207968	1.835053
C	12.356413	-2.753723	2.408099
H	14.512806	-2.778368	2.322278
H	10.200140	-2.471150	2.210588
H	12.274039	-3.454159	3.240701
C	-1.912236	1.537726	13.598231
C	-0.845364	0.610148	13.627885
C	-0.334548	0.170750	12.380507
N	-0.827189	0.614458	11.204792
C	-1.831128	1.475581	11.198143
C	-2.409365	1.971201	12.382524
H	-2.337139	1.899068	14.538373
H	-2.199137	1.785044	10.217144
H	-3.238312	2.678551	12.326465
C	2.353013	-2.197244	13.481926
C	2.818592	-2.590270	12.240262
C	2.210181	-2.056396	11.088065
N	1.206835	-1.196694	11.148977
C	0.744735	-0.792039	12.350867
C	1.287465	-1.271783	13.569523
H	2.802042	-2.588856	14.398434
H	3.645833	-3.294670	12.139674
H	2.552737	-2.332794	10.088169
C	2.730719	13.519294	-1.473199
C	1.641215	13.599638	-0.575392
C	1.055073	12.378224	-0.157684
N	1.499284	11.180773	-0.594237
C	2.525379	11.126255	-1.427045
C	3.176983	12.281582	-1.899693
H	3.213012	14.437965	-1.817314
H	2.851110	10.128904	-1.731473
H	4.021359	12.186166	-2.584209
C	-1.638471	13.605530	2.140478
C	-2.178130	12.387657	2.512631
C	-1.614800	11.208168	1.988972
N	-0.585655	11.221240	1.158222
C	-0.051138	12.399670	0.774207
C	-0.544030	13.642447	1.245852
H	-2.050831	14.542106	2.524873
H	-3.029129	12.326494	3.192844
H	-2.016122	10.226045	2.249573
C	1.703375	-13.657400	1.419777
C	2.249450	-12.449568	1.814645
C	1.663486	-11.256466	1.349979
N	0.607231	-11.247551	0.553947

C	0.065581	-12.415548	0.148583
C	0.579875	-13.670615	0.561267
H	2.132591	-14.603942	1.758619
H	3.122240	-12.406352	2.468110
H	2.068956	-10.281452	1.630123
C	-2.785839	-13.475369	-2.039949
C	-1.665891	-13.579478	-1.182957
C	-1.071870	-12.369408	-0.744008
N	-1.536998	-11.160623	-1.123768
C	-2.591410	-11.084065	-1.918609
C	-3.252794	-12.226667	-2.408273
H	-3.275090	-14.384765	-2.398567
H	-2.932623	-10.078872	-2.176725
H	-4.120750	-12.113250	-3.059741
C	-13.507572	2.695305	2.054118
C	-12.258905	3.140985	2.447999
C	-11.116389	2.500493	1.931091
N	-11.192928	1.485364	1.086638
C	-12.401678	1.042134	0.681361
C	-13.611686	1.617533	1.144660
H	-14.416910	3.169244	2.432885
H	-12.145394	3.976687	3.140344
H	-10.111275	2.826332	2.208666
C	-13.689816	-1.625308	-1.613968
C	-13.702921	-0.543195	-0.703827
C	-12.447888	-0.051440	-0.264477
N	-11.279967	-0.575547	-0.692461
C	-11.289000	-1.593310	-1.537168
C	-12.482108	-2.154660	-2.031285
H	-14.636373	-2.036411	-1.974531
H	-10.314069	-1.986943	-1.833960
H	-12.438952	-2.996134	-2.724607
O	-1.988127	-2.225461	11.384295
O	2.375832	1.637039	11.402998
O	0.972486	-9.436241	-1.823840
O	-2.527338	-11.384686	1.781535
O	1.310224	-1.237281	-9.452670
O	-2.098499	2.496346	-11.304799
O	-9.480999	-1.061212	1.672662
O	-11.397321	2.613478	-1.766853
O	9.528505	1.082201	1.312819
O	11.342038	-2.396742	-2.366018
O	-1.655770	11.550293	-1.706735
O	2.587597	11.283613	2.278062
N	-1.944650	10.452721	-1.248684
O	-3.038981	10.190190	-0.744254
O	-1.046166	9.508126	-1.273773
N	2.788964	10.188422	1.770247
O	3.860601	9.861948	1.254339
O	1.817485	9.318954	1.752389
N	-1.584072	2.698864	-10.212915
O	-1.027620	3.755942	-9.906120
O	-1.604001	1.744057	-9.325206

N	1.252231	-2.152221	-10.379743
O	0.709720	-3.222851	-10.095791
O	1.718864	-1.901116	-11.482956
N	10.455667	1.028515	2.228045
O	11.561651	1.485351	1.971012
O	10.168986	0.499868	3.304754
N	10.255389	-1.875632	-2.579155
O	9.955550	-1.333793	-3.645688
O	9.365616	-1.871579	-1.625874
N	1.877255	-10.374186	-1.866611
O	1.579024	-11.456191	-2.354579
O	2.987598	-10.121832	-1.392738
N	-2.750355	-10.273211	1.320161
O	-3.838829	-9.935729	0.848703
O	-1.784664	-9.397208	1.304957
N	-2.233616	-1.717732	10.298109
O	-1.309694	-1.730481	9.378266
O	-3.306828	-1.174401	10.025659
N	2.591833	1.165882	10.294257
O	1.644969	1.213379	9.399148
O	3.655353	0.628287	9.976539
N	-10.287800	2.813120	-1.290332
O	-9.413161	1.846402	-1.315354
O	-9.950999	3.878141	-0.767626
N	-10.422802	-1.963074	1.667701
O	-10.173541	-3.049575	1.140237
O	-11.505187	-1.684564	2.166448
C	0.070954	14.862295	0.798754
C	1.119481	14.841751	-0.074290
C	14.891782	-0.105585	-0.777521
C	14.885986	-1.003608	0.249871
C	0.748701	-0.802017	14.816594
C	-0.274773	0.100052	14.844590
C	0.191575	0.870365	-14.829767
C	-0.721387	-0.143790	-14.829734
C	-14.866811	1.097425	0.675313
C	-14.910575	0.060587	-0.210730
C	-0.044049	-14.878353	0.094097
C	-1.121464	-14.834637	-0.742141
H	-15.789347	1.552325	1.044129
H	-15.868787	-0.330537	-0.561277
H	-1.112388	-0.539293	-15.770330
H	0.541949	1.302383	-15.770312
H	15.834516	0.232591	-1.214468
H	15.824095	-1.394626	0.651228
H	-1.591302	-15.757242	-1.091542
H	0.365028	-15.836583	0.423463
H	1.178479	-1.183575	15.745965
H	-0.680142	0.450927	15.796739
H	1.582710	15.773397	-0.408181
H	-0.321289	15.810786	1.173636

162

CAGEphen+phen+12CoDividedBy3big SCF Done: -7091.28497986 a.u.

Co	-8.930822	0.009524	-0.731852
N	-2.120130	0.328180	1.056228
N	-0.762100	1.559599	2.558469
C	-0.937615	0.649826	1.600244
C	-3.168406	1.008123	1.550151
N	-3.084925	1.924044	2.516019
C	-1.863813	2.184708	3.007570
C	-1.725496	3.193131	4.076934
C	-0.472508	3.477674	4.644976
C	-2.843106	3.896595	4.555615
C	-0.399874	4.441458	5.654179
C	-2.654520	4.840633	5.569085
N	-1.463119	5.108752	6.107999
H	0.422338	2.955975	4.305964
H	-3.834782	3.711324	4.143339
H	0.563533	4.684421	6.116043
H	-3.507473	5.405195	5.961479
C	-4.527482	0.751474	0.993779
C	-5.646510	1.393749	1.549757
C	-4.748246	-0.108426	-0.094320
C	-6.907594	1.158765	1.016717
C	-6.043908	-0.286442	-0.570960
N	-7.115018	0.334150	-0.029617
H	-5.523152	2.073849	2.392033
H	-3.913844	-0.632110	-0.561209
H	-7.790581	1.648304	1.429615
H	-6.248254	-0.946531	-1.416541
C	0.293512	-0.037757	1.113065
C	1.558328	0.402833	1.540203
C	0.258235	-1.126280	0.227243
C	2.699241	-0.233439	1.071567
C	1.454490	-1.712581	-0.180534
N	2.668245	-1.276621	0.217289
H	1.637344	1.248726	2.222915
H	-0.696345	-1.504929	-0.140091
H	3.684793	0.112585	1.382683
H	1.443485	-2.562504	-0.864994
Co	4.458254	-2.494790	0.026126
N	7.988428	3.402409	-1.345810
N	9.633796	2.230095	-0.105558
C	8.426360	2.360065	-0.645871
C	8.879813	4.404130	-1.504288
N	10.115630	4.359660	-0.996922
C	10.470849	3.271137	-0.304954
C	11.833126	3.204988	0.263874
C	12.257793	2.089184	1.004115
C	12.741212	4.259763	0.078423
C	14.018047	4.147317	0.637551
N	14.420108	3.086171	1.341152
H	11.586713	1.247083	1.173514
H	12.454839	5.145036	-0.489287
H	14.746709	4.954962	0.508274
C	8.470605	5.600551	-2.271438

C	7.185548	5.700174	-2.829402
C	9.358378	6.670947	-2.462538
C	6.855207	6.853991	-3.543231
C	8.920779	7.779002	-3.195271
N	7.698504	7.872741	-3.724790
H	6.463909	4.892464	-2.706504
H	10.365762	6.636378	-2.047697
H	5.859727	6.958651	-3.989961
H	9.593104	8.628108	-3.359325
C	7.471618	1.227827	-0.449213
C	7.842358	0.081690	0.269352
C	6.173007	1.270560	-0.978007
C	6.927469	-0.951410	0.423419
C	5.318778	0.194708	-0.776697
N	5.676052	-0.907679	-0.084011
H	8.843963	0.010694	0.695101
H	5.848564	2.145461	-1.542377
H	7.201762	-1.850427	0.972714
H	4.306505	0.208965	-1.177457
C	13.556698	2.083437	1.517538
H	13.915283	1.226568	2.099189
C	4.376490	-3.260560	4.757601
C	5.000127	-2.106602	4.309884
C	5.032530	-1.823756	2.931454
N	4.477112	-2.624032	2.021727
C	3.843100	-3.751601	2.452378
C	3.772613	-4.128444	3.816747
H	4.348996	-3.504681	5.822579
H	5.472820	-1.412611	5.007177
H	5.526033	-0.922784	2.566583
C	2.085734	-6.578287	0.757430
C	2.617576	-5.794557	1.808902
C	3.252761	-4.579999	1.449267
N	3.357599	-4.149560	0.162759
C	2.857553	-4.911045	-0.809028
C	2.210581	-6.133474	-0.549811
H	1.589748	-7.526171	0.980783
H	2.969235	-4.545415	-1.830657
H	1.818010	-6.716828	-1.384498
C	-10.625098	-1.343141	3.494731
C	-9.741179	-2.187582	2.840409
C	-9.195671	-1.793928	1.603810
N	-9.496407	-0.628498	1.035291
C	-10.347782	0.221247	1.674062
C	-10.954690	-0.093777	2.915668
H	-11.070377	-1.633134	4.449781
H	-9.469494	-3.156303	3.263049
H	-8.509587	-2.442631	1.054081
C	-11.770076	3.599363	0.932379
C	-11.531625	2.388432	1.628343
C	-10.629028	1.473928	1.031609
N	-9.990600	1.719818	-0.141330
C	-10.231758	2.860812	-0.775167

C	-11.120896	3.831856	-0.269465
H	-12.461914	4.337472	1.345827
H	-9.704151	3.019801	-1.720044
H	-11.285365	4.754468	-0.829249
C	-11.863530	0.854391	3.503033
C	-12.143546	2.041611	2.883658
C	2.559823	-6.158946	3.199330
C	3.109775	-5.357909	4.162147
H	3.060820	-5.646397	5.214594
H	2.069099	-7.095262	3.474472
H	-12.333514	0.605341	4.457100
H	-12.840568	2.749266	3.338461
C	-8.381298	1.523138	-5.202988
C	-9.439764	0.676109	-4.802769
C	-9.456018	0.261110	-3.448217
N	-8.502325	0.619915	-2.538572
C	-7.532196	1.447571	-2.941283
C	-7.437142	1.914247	-4.263070
H	-8.322264	1.873039	-6.236406
H	-6.804477	1.753497	-2.188810
H	-6.618980	2.583720	-4.533879
C	-12.591600	-1.749047	-3.253336
C	-12.538637	-2.053941	-1.900809
C	-11.470417	-1.582861	-1.118388
N	-10.472037	-0.851098	-1.623539
C	-10.521210	-0.544669	-2.953383
C	-11.561048	-0.963244	-3.818300
H	-13.416519	-2.102453	-3.876543
H	-13.317217	-2.656103	-1.429291
H	-11.425483	-1.814344	-0.054123
C	-11.512506	-0.556996	-5.198290
C	-10.498583	0.231104	-5.669603
H	-12.312628	-0.883989	-5.866220
H	-10.482279	0.542178	-6.716506
C	8.180116	-5.461796	-1.000748
C	7.413145	-4.792965	-1.985936
C	6.350614	-3.972698	-1.528611
N	6.066793	-3.803993	-0.213486
C	6.791677	-4.451655	0.692508
C	7.862612	-5.295468	0.337796
H	9.011073	-6.104908	-1.301710
H	6.527493	-4.297893	1.741660
H	8.429881	-5.802817	1.120344
C	4.903946	-2.720054	-4.741450
C	3.871589	-1.957284	-4.219934
C	3.698363	-1.889610	-2.825274
N	4.492555	-2.536172	-1.974344
C	5.514704	-3.287495	-2.470889
C	5.764719	-3.418469	-3.861027
H	5.060070	-2.786672	-5.821172
H	3.192399	-1.405024	-4.871523
H	2.898586	-1.289111	-2.390535
C	6.859712	-4.241719	-4.299386

C	7.649081	-4.903764	-3.400189
H	7.048381	-4.332116	-5.371478
H	8.474375	-5.530424	-3.745945

126

CAGEphen+phen+12CoDividedBy3 SCF Done: -6070.28093753 A.U.

N	0.000006	1.211488	0.000107
N	-1.175456	3.266082	0.006265
C	-1.128772	1.937397	0.007629
C	1.128785	1.937395	-0.007446
N	1.175469	3.266081	-0.006162
C	0.000007	3.919644	0.000032
C	0.000009	5.393101	-0.000031
C	1.204814	6.115844	-0.037600
C	-1.204795	6.115853	0.037476
C	1.145737	7.512148	-0.036611
C	-1.145711	7.512155	0.036365
N	0.000014	8.195997	-0.000153
H	2.163848	5.599196	-0.068999
H	-2.163831	5.599211	0.068918
H	2.067719	8.103058	-0.066840
H	-2.067691	8.103072	0.066541
C	2.446643	1.227647	-0.019202
C	3.641054	1.964794	0.014421
C	2.545046	-0.170883	-0.067226
C	4.860100	1.296062	0.003570
C	3.805763	-0.763977	-0.082424
N	4.947943	-0.046487	-0.046693
H	3.609839	3.053540	0.049771
H	1.646133	-0.787084	-0.094797
H	5.802239	1.844585	0.031829
H	3.914838	-1.850260	-0.130837
C	-2.446632	1.227649	0.019388
C	-3.641040	1.964795	-0.014273
C	-2.545036	-0.170880	0.067425
C	-4.860089	1.296064	-0.003444
C	-3.805755	-0.763972	0.082596
N	-4.947933	-0.046482	0.046828
H	-3.609825	3.053540	-0.049645
H	-1.646124	-0.787082	0.095018
H	-5.802224	1.844589	-0.031731
H	-3.914829	-1.850255	0.131006
Co	-6.679211	-1.001723	-0.044776
Co	6.679257	-1.001677	0.044836
C	-10.129710	-4.302624	-0.038248
C	-9.321925	-4.018008	1.088542
C	-8.318759	-3.033071	0.927477
N	-8.105456	-2.357174	-0.238641
C	-8.891650	-2.640146	-1.280627
C	-9.908501	-3.609850	-1.218997
H	-10.917635	-5.056889	0.029074
H	-8.713317	-2.086817	-2.202753
H	-10.512892	-3.803593	-2.106897
C	-6.782687	-2.879482	4.324013

C	-5.851053	-1.882650	4.070977
C	-5.756797	-1.325211	2.783703
N	-6.533351	-1.724616	1.772283
C	-7.470451	-2.686201	2.019066
C	-7.636452	-3.308218	3.280312
H	-6.868533	-3.325880	5.317823
H	-5.187695	-1.520667	4.858275
H	-5.035711	-0.535621	2.569381
C	7.601361	0.685735	4.386673
C	6.643517	-0.309773	4.264355
C	6.325547	-0.812186	2.988054
N	6.914755	-0.358551	1.883818
C	7.845496	0.630429	1.984524
C	8.237698	1.189659	3.225714
H	7.870997	1.081427	5.369236
H	6.139017	-0.717473	5.142021
H	5.586493	-1.607202	2.860923
C	9.996752	2.486351	-0.445019
C	9.443487	2.092852	0.799185
C	8.444336	1.089809	0.765683
N	8.002461	0.523286	-0.387410
C	8.536682	0.905517	-1.541770
C	9.543514	1.890490	-1.611521
H	10.775279	3.252869	-0.476576
H	8.158844	0.420615	-2.445776
H	9.952365	2.172159	-2.583743
C	-8.656375	-4.312811	3.423236
C	-9.462369	-4.654622	2.371514
C	9.250283	2.211979	3.234237
C	9.831091	2.641585	2.071849
H	10.603416	3.413903	2.094796
H	9.554063	2.638708	4.192800
H	-8.779889	-4.798445	4.393777
H	-10.234417	-5.417663	2.493830
C	-7.600907	0.685235	-4.386859
C	-8.237307	1.189329	-3.226006
C	-7.845222	0.630226	-1.984722
N	-6.914521	-0.358772	-1.883824
C	-6.325259	-0.812567	-2.987963
C	-6.643119	-0.310304	-4.264350
H	-7.870451	1.080824	-5.369489
H	-5.586244	-1.607595	-2.860683
H	-6.138577	-0.718140	-5.141929
C	-9.996607	2.486489	0.444445
C	-9.543497	1.890722	1.611046
C	-8.536704	0.905694	1.541479
N	-8.002404	0.523332	0.387200
C	-8.444149	1.089763	-0.765985
C	-9.443252	2.092844	-0.799674
H	-10.775102	3.253045	0.475859
H	-9.952423	2.172501	2.583204
H	-8.158969	0.420855	2.445561
C	-9.830727	2.641458	-2.072429

C	-9.249845	2.211693	-3.234723
H	-10.603015	3.413808	-2.095525
H	-9.553527	2.638331	-4.193358
C	6.782226	-2.879965	-4.323725
C	7.636019	-3.308668	-3.280034
C	7.470189	-2.686469	-2.018854
N	6.533227	-1.724734	-1.772124
C	5.756643	-1.325369	-2.783538
C	5.850732	-1.882989	-4.070747
H	6.867943	-3.326502	-5.317484
H	5.035668	-0.535665	-2.569267
H	5.187359	-1.521025	-4.858042
C	10.129426	-4.302933	0.038460
C	9.908391	-3.609974	1.219135
C	8.891659	-2.640143	1.280717
N	8.105409	-2.357226	0.238756
C	8.318544	-3.033301	-0.927288
C	9.321587	-4.018372	-1.088304
H	10.917262	-5.057294	-0.028825
H	10.512832	-3.803666	2.107012
H	8.713470	-2.086662	2.202780
C	9.461853	-4.655167	-2.371205
C	8.655815	-4.313399	-3.422906
H	10.233805	-5.418311	-2.493486
H	8.779199	-4.799168	-4.393396

126

CAGEphen+phen+12CuDividedBy3 SCF Done: -6585.62270783 A.U.

N	0.000062	1.311448	-0.000180
N	-1.175315	3.365226	0.001653
C	-1.129197	2.035846	0.003412
C	1.129334	2.035735	-0.003903
N	1.175541	3.365021	-0.002232
C	0.000296	4.016736	-0.000302
C	0.000306	5.492385	-0.000217
C	1.205404	6.213944	-0.039009
C	-1.205107	6.213365	0.038682
C	1.147640	7.610661	-0.037955
C	-1.147946	7.610173	0.037812
N	-0.000337	8.287478	-0.000034
H	2.164298	5.697176	-0.071717
H	-2.163717	5.696115	0.071337
H	2.068162	8.203778	-0.069282
H	-2.068787	8.202805	0.069227
C	2.447359	1.325039	-0.010852
C	3.640561	2.064061	0.040446
C	2.542188	-0.073089	-0.071946
C	4.858316	1.391184	0.036015
C	3.804399	-0.667892	-0.083051
N	4.939094	0.050936	-0.025962
H	3.608924	3.152504	0.085141
H	1.641474	-0.686133	-0.114303
H	5.805236	1.934559	0.082260
H	3.916641	-1.753511	-0.143317

C	-2.447146	1.325091	0.010312
C	-3.640318	2.064079	-0.041906
C	-2.542039	-0.073054	0.072256
C	-4.858042	1.391142	-0.037547
C	-3.804175	-0.667844	0.083295
N	-4.938924	0.050945	0.025220
H	-3.608629	3.152436	-0.087298
H	-1.641314	-0.686015	0.115271
H	-5.804938	1.934500	-0.084527
H	-3.916530	-1.753418	0.144219
Cu	-6.859090	-0.801137	-0.002050
Cu	6.859109	-0.801189	0.001883
C	-9.549934	-4.869623	-0.439991
C	-8.874465	-4.497167	0.748575
C	-8.089342	-3.317794	0.702284
N	-7.972288	-2.558374	-0.418398
C	-8.621206	-2.922985	-1.517334
C	-9.422308	-4.081873	-1.571601
H	-10.168085	-5.770922	-0.454080
H	-8.510467	-2.280504	-2.394217
H	-9.931483	-4.340794	-2.501725
C	-6.794610	-3.121642	4.210080
C	-6.060555	-1.950655	4.100646
C	-6.014602	-1.282178	2.862406
N	-6.656953	-1.739233	1.792669
C	-7.389812	-2.880872	1.877706
C	-7.489123	-3.623862	3.082819
H	-6.846774	-3.658649	5.160725
H	-5.520700	-1.539036	4.955232
H	-5.445836	-0.357509	2.735960
C	8.264126	1.211550	4.143885
C	7.164334	0.367638	4.158744
C	6.713107	-0.186960	2.946862
N	7.310339	0.073501	1.788194
C	8.384718	0.905469	1.743084
C	8.909505	1.507328	2.918811
H	8.639318	1.651651	5.071502
H	6.647549	0.125330	5.089021
H	5.850635	-0.858582	2.917259
C	10.691046	2.240857	-0.887092
C	10.127354	2.021969	0.394725
C	8.995361	1.169693	0.465279
N	8.449649	0.581686	-0.629880
C	8.998454	0.801205	-1.817344
C	10.126368	1.629535	-1.993533
H	11.565733	2.887876	-0.991723
H	8.533975	0.302999	-2.673678
H	10.538880	1.777789	-2.993211
C	-8.287645	-4.819725	3.099094
C	-8.950653	-5.238573	1.979172
C	10.056285	2.367969	2.811761
C	10.641448	2.612839	1.601101
H	11.514258	3.266290	1.532343

H	10.455081	2.822228	3.721632
H	-8.359687	-5.387657	4.029406
H	-9.556886	-6.147041	2.004284
C	-8.265881	1.211758	-4.143522
C	-8.910859	1.507360	-2.918194
C	-8.385564	0.905474	-1.742710
N	-7.311115	0.073634	-1.788263
C	-6.714256	-0.186650	-2.947156
C	-7.165980	0.367996	-4.158837
H	-8.641467	1.651873	-5.070973
H	-5.851687	-0.858164	-2.917902
H	-6.649492	0.125834	-5.089316
C	-10.691025	2.240414	0.888447
C	-10.125837	1.629092	1.994628
C	-8.997887	0.800917	1.817949
N	-8.449518	0.581545	0.630257
C	-8.995738	1.169543	-0.464654
C	-10.127807	2.021675	-0.393602
H	-11.565751	2.887317	0.993461
H	-10.537970	1.777232	2.994479
H	-8.533010	0.302723	2.674073
C	-10.642438	2.612558	-1.599744
C	-10.057705	2.367851	-2.810646
H	-11.515303	3.265895	-1.530605
H	-10.456905	2.822128	-3.720331
C	6.795351	-3.121548	-4.210300
C	7.489823	-3.623703	-3.082985
C	7.390310	-2.880747	-1.877870
N	6.657290	-1.739203	-1.792864
C	6.014996	-1.282199	-2.862665
C	6.061151	-1.950647	-4.100912
H	6.847662	-3.658532	-5.160950
H	5.446108	-0.357602	-2.736267
H	5.521329	-1.539075	-4.955542
C	9.550513	-4.869224	0.439987
C	9.422704	-4.081486	1.571589
C	8.621460	-2.922701	1.517260
N	7.972576	-2.558188	0.418269
C	8.089806	-3.317591	-0.702402
C	8.875087	-4.496860	-0.748634
H	10.168782	-5.770440	0.454127
H	9.931847	-4.340342	2.501748
H	8.510567	-2.280216	2.394123
C	8.951465	-5.238247	-1.979231
C	8.288497	-4.819467	-3.099203
H	9.557816	-6.146637	-2.004307
H	8.360691	-5.387379	-4.029515

162

CAGEphen+phen+12FeDividedBy3big SCF Done: -6853.26953186 A.U.

Fe	8.902955	-0.060129	0.641920
N	2.046269	0.409896	-0.975342
N	0.641937	1.768491	-2.311042
C	0.848169	0.749250	-1.480239

C	3.060980	1.190965	-1.375432
N	2.944377	2.224632	-2.203995
C	1.711631	2.499235	-2.664860
C	1.528653	3.636820	-3.585289
C	0.260501	3.950200	-4.102934
C	2.616643	4.438845	-3.969245
C	0.144304	5.039265	-4.971106
C	2.385018	5.504322	-4.843445
N	1.179461	5.799649	-5.334440
H	-0.612764	3.356394	-3.833668
H	3.618797	4.235347	-3.592435
H	-0.831354	5.307718	-5.390907
H	3.213362	6.147667	-5.159993
C	4.440878	0.908991	-0.868541
C	5.518861	1.700459	-1.296447
C	4.715013	-0.126212	0.037535
C	6.797954	1.436607	-0.821005
C	6.024459	-0.315792	0.473678
N	7.057172	0.447292	0.056130
H	5.349339	2.516011	-1.999035
H	3.911881	-0.768227	0.400324
H	7.649077	2.035888	-1.145271
H	6.259752	-1.104046	1.195981
C	-0.359049	-0.047910	-1.103881
C	-1.622594	0.329435	-1.583112
C	-0.298074	-1.181557	-0.281260
C	-2.741868	-0.412829	-1.226947
C	-1.472368	-1.870750	0.020174
N	-2.688795	-1.500986	-0.432200
H	-1.725177	1.201021	-2.228669
H	0.657424	-1.525108	0.116017
H	-3.726754	-0.123079	-1.588154
H	-1.441153	-2.760638	0.648863
Fe	-4.385950	-2.606159	-0.098252
N	-7.813955	3.399545	1.331392
N	-9.500712	2.259289	0.120704
C	-8.280057	2.363276	0.638422
C	-8.683711	4.415176	1.505428
N	-9.929185	4.396599	1.021280
C	-10.313980	3.313862	0.335625
C	-11.688058	3.271351	-0.209345
C	-12.144520	2.163035	-0.941329
C	-12.574850	4.341066	-0.008609
C	-13.862824	4.250333	-0.545347
N	-14.295634	3.196366	-1.241591
H	-11.490396	1.309867	-1.121295
H	-12.263282	5.221108	0.553972
H	-14.575169	5.070410	-0.403484
C	-8.237457	5.604183	2.266430
C	-6.943718	5.674740	2.807671
C	-9.097052	6.695047	2.467736
C	-6.577348	6.821409	3.515325
C	-8.624225	7.793797	3.192497

N	-7.393300	7.860602	3.706130
H	-6.244021	4.849168	2.676735
H	-10.110321	6.682645	2.066104
H	-5.573999	6.902511	3.949463
H	-9.274751	8.658430	3.363539
C	-7.349520	1.214854	0.423534
C	-7.750848	0.077092	-0.290199
C	-6.042139	1.232067	0.929881
C	-6.854279	-0.970744	-0.461416
C	-5.211797	0.140478	0.708851
N	-5.593461	-0.957351	0.022234
H	-8.760220	0.021973	-0.699134
H	-5.692029	2.097850	1.492585
H	-7.157447	-1.864053	-1.005611
H	-4.194156	0.138848	1.097737
C	-13.452161	2.179252	-1.432074
H	-13.834916	1.328114	-2.006740
C	-4.555118	-3.571354	-4.827983
C	-5.130060	-2.387922	-4.396748
C	-5.098319	-2.060200	-3.027481
N	-4.530329	-2.840163	-2.108047
C	-3.938722	-3.994867	-2.527898
C	-3.933512	-4.419527	-3.880417
H	-4.579337	-3.855700	-5.882890
H	-5.617907	-1.708083	-5.097500
H	-5.561879	-1.138114	-2.674910
C	-2.181649	-6.819999	-0.820702
C	-2.738014	-6.051124	-1.870586
C	-3.323097	-4.807850	-1.521620
N	-3.353138	-4.330753	-0.247943
C	-2.827897	-5.079488	0.720042
C	-2.232486	-6.331562	0.474497
H	-1.727424	-7.790048	-1.037227
H	-2.881959	-4.677927	1.733597
H	-1.824026	-6.903023	1.309801
C	10.228898	-1.093793	-3.834887
C	9.451756	-2.030655	-3.169576
C	9.016221	-1.767277	-1.855973
N	9.324310	-0.639151	-1.218870
C	10.076033	0.291836	-1.867182
C	10.565777	0.118273	-3.183518
H	10.583433	-1.283662	-4.851118
H	9.177925	-2.973889	-3.645448
H	8.411834	-2.495572	-1.307757
C	11.403069	3.655560	-0.935678
C	11.157697	2.503359	-1.722109
C	10.369833	1.484464	-1.136975
N	9.845010	1.570296	0.119256
C	10.090539	2.665700	0.837930
C	10.866895	3.730365	0.340464
H	12.009367	4.471973	-1.335953
H	9.662057	2.703423	1.840026
H	11.037851	4.603937	0.972087

C	11.367021	1.164963	-3.761033
C	11.651797	2.305953	-3.059872
C	-2.750555	-6.462190	-3.248686
C	-3.318629	-5.676810	-4.213081
H	-3.323524	-5.999744	-5.256617
H	-2.298756	-7.421098	-3.512510
H	11.750248	1.031931	-4.775282
H	12.264085	3.090374	-3.510572
C	8.602191	1.048049	5.309747
C	9.679314	0.312475	4.758709
C	9.629423	0.043906	3.368797
N	8.605480	0.450232	2.563576
C	7.609509	1.149041	3.111784
C	7.572105	1.466860	4.482056
H	8.594144	1.284897	6.376693
H	6.815449	1.479493	2.440276
H	6.733580	2.044216	4.875046
C	12.820562	-1.816848	2.808468
C	12.697728	-2.015922	1.443227
C	11.563979	-1.524481	0.768529
N	10.583908	-0.869651	1.392299
C	10.696451	-0.669910	2.736643
C	11.798768	-1.121591	3.500763
H	13.693646	-2.187059	3.351754
H	13.466926	-2.546360	0.879193
H	11.455021	-1.668588	-0.307059
C	11.822728	-0.848599	4.913104
C	10.806928	-0.159256	5.516729
H	12.675082	-1.200405	5.498747
H	10.839954	0.046527	6.588930
C	-8.113605	-5.383119	1.182556
C	-7.282594	-4.722746	2.118646
C	-6.217971	-3.942005	1.602352
N	-5.983684	-3.789981	0.271543
C	-6.775912	-4.432353	-0.585057
C	-7.851130	-5.240705	-0.169754
H	-8.948036	-5.996928	1.530641
H	-6.564326	-4.299439	-1.647144
H	-8.467333	-5.738945	-0.920221
C	-4.609229	-2.663222	4.724182
C	-3.582349	-1.941859	4.138597
C	-3.463029	-1.919744	2.736381
N	-4.301180	-2.565691	1.926860
C	-5.322798	-3.268018	2.494257
C	-5.520605	-3.359468	3.895788
H	-4.723586	-2.698090	5.810395
H	-2.864560	-1.388161	4.746052
H	-2.660394	-1.352626	2.263207
C	-6.618135	-4.142785	4.395957
C	-7.460387	-4.800983	3.543620
H	-6.765639	-4.205431	5.476420
H	-8.287515	-5.397487	3.935122

126

CAGEphen+phen+12FeDividedBy3 SCF Done: -5832.24405953 A.U.

N	-0.000043	1.211163	-0.000024
N	-1.175100	3.266369	0.011022
C	-1.129032	1.937414	0.011418
C	1.128951	1.937407	-0.011477
N	1.175027	3.266362	-0.011085
C	-0.000034	3.919632	-0.000032
C	-0.000030	5.393420	-0.000022
C	1.204494	6.116180	-0.042515
C	-1.204550	6.116186	0.042483
C	1.145457	7.512490	-0.041257
C	-1.145503	7.512497	0.041247
N	-0.000021	8.196466	0.000001
H	2.163347	5.599442	-0.077825
H	-2.163406	5.599454	0.077785
H	2.067359	8.103335	-0.075331
H	-2.067402	8.103346	0.075330
C	2.448527	1.231583	-0.026272
C	3.640370	1.973678	-0.006424
C	2.554936	-0.166709	-0.063378
C	4.863069	1.312388	-0.019504
C	3.819988	-0.749813	-0.086403
N	4.962400	-0.030586	-0.063268
H	3.603678	3.062449	0.021300
H	1.659561	-0.788519	-0.081009
H	5.799794	1.869888	0.001961
H	3.928024	-1.838022	-0.135543
C	-2.448612	1.231599	0.026205
C	-3.640449	1.973698	0.006273
C	-2.555033	-0.166692	0.063374
C	-4.863156	1.312416	0.019342
C	-3.820091	-0.749781	0.086373
N	-4.962497	-0.030552	0.063168
H	-3.603753	3.062467	-0.021511
H	-1.659665	-0.788510	0.081069
H	-5.799873	1.869926	-0.002188
H	-3.928123	-1.837991	0.135551
Fe	-6.697257	-1.020926	-0.077870
Fe	6.697197	-1.020838	0.078056
C	-10.273510	-4.236213	-0.082722
C	-9.437814	-4.008983	1.038309
C	-8.418053	-3.038216	0.894386
N	-8.215407	-2.327931	-0.252089
C	-9.021175	-2.561980	-1.288708
C	-10.060490	-3.510545	-1.243174
H	-11.076249	-4.975610	-0.024378
H	-8.843927	-1.979433	-2.193520
H	-10.686575	-3.659815	-2.124599
C	-6.809714	-3.076207	4.266298
C	-5.852100	-2.099282	4.042097
C	-5.771699	-1.482627	2.779906
N	-6.586109	-1.800919	1.771744
C	-7.539786	-2.752773	1.987411

C	-7.696568	-3.431018	3.221091
H	-6.890558	-3.567590	5.239208
H	-5.161369	-1.796738	4.830930
H	-5.033624	-0.702724	2.585759
C	7.611081	0.830304	4.391875
C	6.693853	-0.208378	4.327125
C	6.371542	-0.775645	3.078538
N	6.919136	-0.348357	1.942232
C	7.811360	0.678065	1.993515
C	8.203972	1.309311	3.197733
H	7.880431	1.277491	5.352067
H	6.222613	-0.599409	5.230553
H	5.659765	-1.602753	3.004181
C	9.808226	2.505415	-0.578789
C	9.309853	2.146792	0.697650
C	8.362442	1.097189	0.743509
N	7.916866	0.439892	-0.365741
C	8.401593	0.797463	-1.554853
C	9.350152	1.828626	-1.698510
H	10.545804	3.306366	-0.672691
H	8.030837	0.250580	-2.422430
H	9.714926	2.080019	-2.696037
C	-8.736580	-4.416861	3.343930
C	-9.570703	-4.694946	2.295924
C	9.170068	2.373895	3.131031
C	9.700688	2.773104	1.933658
H	10.435736	3.580497	1.899415
H	9.478411	2.860126	4.059349
H	-8.851657	-4.940693	4.295460
H	-10.358313	-5.444474	2.401449
C	-7.610330	0.827611	-4.393002
C	-8.203316	1.307444	-3.199237
C	-7.810966	0.676882	-1.994576
N	-6.918904	-0.349645	-1.942534
C	-6.371228	-0.777724	-3.078503
C	-6.693277	-0.211174	-4.327484
H	-7.879475	1.274250	-5.353507
H	-5.659595	-1.604901	-3.003542
H	-6.221977	-0.602842	-5.230605
C	-9.808048	2.506005	0.576310
C	-9.350265	1.829809	1.696508
C	-8.401795	0.798455	1.553622
N	-7.916890	0.440154	0.364804
C	-8.362194	1.096843	-0.744911
C	-9.309489	2.146584	-0.699833
H	-10.545549	3.307098	0.669613
H	-9.715202	2.081818	2.693819
H	-8.031254	0.252028	2.421579
C	-9.700037	2.772203	-1.936281
C	-9.169275	2.372201	-3.133326
H	-10.434986	3.579713	-1.902648
H	-9.477406	2.857914	-4.061986
C	6.809416	-3.078658	-4.264896

C	7.696253	-3.432941	-3.219496
C	7.539526	-2.753991	-1.986195
N	6.585899	-1.801971	-1.771056
C	5.771511	-1.484182	-2.779392
C	5.851869	-2.101542	-4.041241
H	6.890228	-3.570589	-5.237532
H	5.033486	-0.704123	-2.585676
H	5.161162	-1.799388	-4.830245
C	10.273209	-4.236414	0.084721
C	10.060272	-3.510064	1.244761
C	9.021035	-2.561388	1.289770
N	8.215259	-2.327873	0.253039
C	8.417808	-3.038849	-0.893028
C	9.437498	-4.009769	-1.036417
H	11.075893	-4.975902	0.026779
H	10.686364	-3.658874	2.126258
H	8.843861	-1.978301	2.194248
C	9.570317	-4.696463	-2.293640
C	8.736199	-4.418920	-3.341793
H	10.357876	-5.446104	-2.398748
H	8.851230	-4.943298	-4.293028

162

CAGEphen+phen+12NiDividedBy3big SCF Done: -7342.40448367 A.U.

Ni	8.899680	1.168594	0.595971
N	2.525446	-1.521076	-0.323860
N	1.527006	-3.379705	-1.388779
C	1.458066	-2.266803	-0.659169
C	3.697693	-1.976350	-0.779543
N	3.857759	-3.074915	-1.512023
C	2.742419	-3.766603	-1.806263
C	2.859676	-4.995013	-2.617595
C	1.723887	-5.755115	-2.942079
C	4.107033	-5.437553	-3.087339
C	1.890739	-6.908758	-3.712342
C	4.157197	-6.607098	-3.851363
N	3.076770	-7.328255	-4.158374
H	0.736127	-5.448610	-2.598268
H	5.016016	-4.880391	-2.861369
H	1.023377	-7.522079	-3.980903
H	5.116172	-6.975862	-4.231664
C	4.938906	-1.204754	-0.452820
C	6.190087	-1.652895	-0.905036
C	4.899198	-0.023364	0.303128
C	7.332781	-0.924339	-0.592849
C	6.086379	0.653055	0.570862
N	7.281077	0.210254	0.129966
H	6.261163	-2.565911	-1.495973
H	3.948183	0.360284	0.673256
H	8.323168	-1.238052	-0.925965
H	6.090014	1.581684	1.146085
C	0.105337	-1.838129	-0.195751
C	-1.034840	-2.584652	-0.537602
C	-0.075110	-0.689526	0.587304

C	-2.282356	-2.152570	-0.086129
C	-1.368554	-0.343735	0.988511
N	-2.454997	-1.055888	0.662884
H	-0.937226	-3.483765	-1.145830
H	0.780364	-0.078997	0.876918
H	-3.189892	-2.713145	-0.338332
H	-1.532433	0.549681	1.600326
Ni	-6.226103	-2.090036	0.357290
N	-5.892863	4.751842	-0.489678
N	-8.244555	4.474575	-0.476746
C	-6.998936	4.015622	-0.407730
C	-6.092906	6.074147	-0.656177
N	-7.307389	6.623610	-0.734829
C	-8.365638	5.807993	-0.643235
C	-9.720672	6.391740	-0.728117
C	-10.865909	5.583120	-0.641594
C	-9.898762	7.774178	-0.898090
C	-11.201991	8.275013	-0.972226
N	-12.289969	7.505375	-0.889951
H	-10.775733	4.504921	-0.509611
H	-9.038416	8.439460	-0.970150
H	-11.370272	9.349396	-1.104433
C	-4.907123	6.956942	-0.756673
C	-3.607109	6.432023	-0.683200
C	-5.049867	8.341941	-0.928538
C	-2.526303	7.309571	-0.784265
C	-3.895846	9.127447	-1.017627
N	-2.658990	8.627784	-0.947718
H	-3.451263	5.361356	-0.550445
H	-6.040698	8.792149	-0.990871
H	-1.500795	6.925205	-0.730179
H	-3.980454	10.211311	-1.151871
C	-6.822519	2.543041	-0.221880
C	-7.931563	1.689385	-0.122758
C	-5.542411	1.974102	-0.138581
C	-7.726382	0.325721	0.048473
C	-5.412348	0.601268	0.034358
N	-6.489988	-0.205459	0.120404
H	-8.939886	2.100448	-0.179809
H	-4.662292	2.613285	-0.209240
H	-8.558207	-0.374470	0.130098
H	-4.432096	0.122403	0.118204
C	-12.120616	6.191012	-0.728591
H	-13.031379	5.584912	-0.664586
C	-6.249805	-3.421733	-4.187604
C	-6.435906	-2.076163	-3.914092
C	-6.392338	-1.619280	-2.582575
N	-6.170064	-2.440720	-1.556441
C	-5.978519	-3.765632	-1.816242
C	-6.010560	-4.318008	-3.117567
H	-6.288876	-3.794274	-5.214309
H	-6.622792	-1.358652	-4.714888
H	-6.543843	-0.565039	-2.350663

C	-5.323143	-6.748241	0.327370
C	-5.545424	-5.991154	-0.848635
C	-5.744981	-4.600995	-0.683860
N	-5.734112	-3.978405	0.527254
C	-5.520133	-4.712982	1.616940
C	-5.308015	-6.104049	1.554154
H	-5.168728	-7.828118	0.260879
H	-5.518786	-4.190992	2.574122
H	-5.139472	-6.657933	2.479255
C	9.980776	-2.007571	3.902978
C	8.940631	-1.114769	4.103432
C	8.623050	-0.204944	3.083145
N	9.281957	-0.174809	1.924254
C	10.298336	-1.055279	1.688487
C	10.692647	-1.997713	2.680019
H	10.261584	-2.719389	4.683468
H	8.376313	-1.097963	5.037169
H	7.821217	0.525579	3.211849
C	12.760970	-1.802942	-1.038858
C	12.091105	-1.895979	0.206961
C	10.997159	-1.013946	0.422793
N	10.574804	-0.131114	-0.513443
C	11.219385	-0.066513	-1.669155
C	12.329131	-0.883142	-1.977702
H	13.611285	-2.457683	-1.245932
H	10.849012	0.661022	-2.399343
H	12.825647	-0.785872	-2.945049
C	11.792565	-2.884047	2.416204
C	12.469541	-2.829629	1.231496
C	-5.587243	-6.537108	-2.179534
C	-5.809205	-5.735340	-3.266489
H	-5.842344	-6.164326	-4.270525
H	-5.441993	-7.612114	-2.307952
H	12.082615	-3.599043	3.189247
H	13.311333	-3.500466	1.044531
C	8.610473	4.732595	-2.505447
C	9.530719	4.717831	-1.429911
C	9.482712	3.601059	-0.562695
N	8.600860	2.571477	-0.712254
C	7.753979	2.605477	-1.741741
C	7.730214	3.672819	-2.658792
H	8.603702	5.571048	-3.206436
H	7.070464	1.763203	-1.849874
H	7.014587	3.650153	-3.482301
C	12.210540	4.337812	1.871933
C	12.090352	3.195754	2.647048
C	11.115766	2.231208	2.326142
N	10.285535	2.373750	1.293655
C	10.392944	3.496297	0.528949
C	11.342921	4.516268	0.767110
H	12.963110	5.095348	2.104701
H	12.740718	3.027415	3.507152
H	11.014938	1.327940	2.927602

C	11.369570	5.651200	-0.117324
C	10.502524	5.747011	-1.171801
H	12.102619	6.439729	0.067149
H	10.537758	6.611625	-1.838291
C	-10.829596	-3.329802	2.366954
C	-9.703045	-2.826407	3.064358
C	-8.537486	-2.542746	2.299677
N	-8.486103	-2.721494	0.958646
C	-9.553487	-3.195695	0.333458
C	-10.755413	-3.520558	0.998997
H	-11.745619	-3.561947	2.916117
H	-9.471355	-3.326006	-0.751140
H	-11.605145	-3.907986	0.433749
C	-6.216342	-1.346011	5.004586
C	-5.083231	-1.104477	4.246717
C	-5.122472	-1.344698	2.863536
N	-6.222104	-1.798197	2.259265
C	-7.357832	-2.043474	2.974560
C	-7.391117	-1.830007	4.381469
H	-6.215130	-1.169089	6.083153
H	-4.163623	-0.733915	4.701962
H	-4.247413	-1.173303	2.227384
C	-8.593607	-2.114616	5.114467
C	-9.703136	-2.597646	4.482519
H	-8.600366	-1.940193	6.192641
H	-10.611361	-2.816618	5.048809

126

CAGEphen+phen+12NiDividedBy3 SCF Done: -6321.40510227 A.U.

N	-0.000020	1.084707	-0.000128
N	-1.175057	3.139458	0.021622
C	-1.128379	1.811317	0.020724
C	1.128366	1.811275	-0.020984
N	1.175095	3.139413	-0.021916
C	0.000031	3.794075	-0.000177
C	0.000058	5.266843	-0.000251
C	1.205091	5.989544	-0.038340
C	-1.204951	5.989589	0.037762
C	1.145986	7.385835	-0.037039
C	-1.145799	7.385878	0.036312
N	0.000105	8.069435	-0.000399
H	2.164324	5.473261	-0.069300
H	-2.164201	5.473342	0.068778
H	2.067891	7.976834	-0.067088
H	-2.067683	7.976912	0.066301
C	2.447993	1.102790	-0.045577
C	3.640586	1.844080	-0.053848
C	2.546952	-0.296522	-0.062820
C	4.861804	1.179138	-0.075825
C	3.807682	-0.889555	-0.089015
N	4.943840	-0.164305	-0.095328
H	3.606928	2.933238	-0.042611
H	1.648253	-0.913637	-0.058467
H	5.809551	1.719541	-0.084274

H	3.920339	-1.975773	-0.114315
C	-2.448033	1.102886	0.045414
C	-3.640595	1.844222	0.053741
C	-2.547048	-0.296423	0.062713
C	-4.861839	1.179328	0.075805
C	-3.807801	-0.889400	0.088999
N	-4.943935	-0.164113	0.095358
H	-3.606898	2.933379	0.042464
H	-1.648373	-0.913571	0.058321
H	-5.809568	1.719763	0.084284
H	-3.920489	-1.975613	0.114377
Ni	-6.673545	-1.048908	0.059632
Ni	6.673377	-1.049075	-0.059584
C	-10.365831	-4.034777	0.356411
C	-9.568091	-3.665533	1.466694
C	-8.487261	-2.789346	1.214392
N	-8.194116	-2.290619	-0.019357
C	-8.962495	-2.653757	-1.045938
C	-10.056396	-3.527730	-0.895283
H	-11.213347	-4.711359	0.492123
H	-8.713491	-2.246830	-2.025794
H	-10.648282	-3.793381	-1.772924
C	-7.002302	-2.327470	4.604641
C	-5.981964	-1.457399	4.252773
C	-5.819498	-1.077679	2.907494
N	-6.618317	-1.529003	1.939699
C	-7.636389	-2.371186	2.278254
C	-7.875039	-2.812538	3.601265
H	-7.141344	-2.634928	5.644174
H	-5.298762	-1.058374	5.004325
H	-5.024416	-0.391641	2.614852
C	7.244654	-0.093068	4.516903
C	6.326189	-1.047916	4.111513
C	6.152620	-1.280795	2.738563
N	6.834325	-0.609317	1.809640
C	7.731681	0.352374	2.176650
C	7.977874	0.635237	3.550252
H	7.413556	0.101104	5.579226
H	5.750076	-1.626932	4.835012
H	5.452579	-2.039592	2.381616
C	10.138146	2.698570	0.513584
C	9.433015	2.033196	1.547887
C	8.457924	1.075521	1.156360
N	8.174691	0.809562	-0.141359
C	8.849406	1.446958	-1.087049
C	9.851123	2.401774	-0.806863
H	10.901206	3.438512	0.768285
H	8.593865	1.202897	-2.123770
H	10.378741	2.895022	-1.625474
C	-8.978245	-3.704836	3.839724
C	-9.788085	-4.115622	2.815819
C	8.958661	1.623884	3.904551
C	9.663733	2.289374	2.942801

H	10.415499	3.031182	3.222433
H	9.136035	1.826267	4.963109
H	-9.160951	-4.049453	4.859964
H	-10.622453	-4.793171	3.010697
C	-7.244746	-0.094297	-4.517133
C	-7.977973	0.634274	-3.550689
C	-7.731723	0.351844	-2.177020
N	-6.834360	-0.609724	-1.809704
C	-6.152661	-1.281480	-2.738436
C	-6.326259	-1.049001	-4.111454
H	-7.413660	0.099542	-5.579515
H	-5.452612	-2.040168	-2.381280
H	-5.750155	-1.628237	-4.834785
C	-10.138396	2.698194	-0.514484
C	-9.851219	2.401802	0.806022
C	-8.849275	1.447283	1.086412
N	-8.174528	0.809744	0.140829
C	-8.457967	1.075263	-1.156925
C	-9.433216	2.032675	-1.548659
H	-10.901607	3.437933	-0.769322
H	-10.378889	2.895162	1.624534
H	-8.593553	1.203628	2.123179
C	-9.663989	2.288426	-2.943648
C	-8.958854	1.622750	-3.905223
H	-10.415846	3.030065	-3.223488
H	-9.136268	1.824843	-4.963832
C	7.002439	-2.329026	-4.604123
C	7.875220	-2.813633	-3.600556
C	7.636480	-2.371863	-2.277702
N	6.618291	-1.529675	-1.939486
C	5.819441	-1.078782	-2.907451
C	5.981979	-1.458964	-4.252591
H	7.141542	-2.636838	-5.643543
H	5.024275	-0.392729	-2.615064
H	5.298749	-1.060288	-5.004304
C	10.366085	-4.034487	-0.355242
C	10.056522	-3.527139	0.896295
C	8.962480	-2.653286	1.046658
N	8.194117	-2.290529	0.019940
C	8.487375	-2.789567	-1.213667
C	9.568332	-3.665683	-1.465666
H	11.213704	-4.710986	-0.490728
H	10.648396	-3.792471	1.774040
H	8.713352	-2.246143	2.026393
C	9.788433	-4.116179	-2.814637
C	8.978555	-3.705854	-3.838696
H	10.622897	-4.793682	-3.009264
H	9.161325	-4.050786	-4.858819

414

CAGEphen+phen+12Ni SCF Done: -19983.8354166 A.U.

N	-2.099502	-4.115135	-2.874952
N	-4.366938	-4.225979	-2.244300
C	-3.387960	-3.795206	-3.044072

C	-1.809942	-4.870268	-1.805860
N	-2.720599	-5.275087	-0.908622
C	-3.997647	-4.953859	-1.179621
C	-5.091661	-5.391070	-0.253847
C	-4.845255	-5.772396	1.075187
C	-6.427380	-5.394498	-0.690361
C	-5.923219	-6.085122	1.907226
C	-7.444162	-5.752727	0.194110
N	-7.198594	-6.070561	1.477953
H	-3.827927	-5.803882	1.466635
H	-6.677322	-5.123894	-1.715945
H	-5.775740	-6.348174	2.955828
H	-8.496231	-5.772885	-0.099157
C	-0.371317	-5.269061	-1.633050
C	0.134310	-5.786721	-0.429651
C	0.529258	-5.142079	-2.703855
C	1.487466	-6.128226	-0.342705
C	1.857290	-5.534249	-2.543838
N	2.339037	-6.007479	-1.379252
H	-0.518933	-5.924653	0.432817
H	0.189432	-4.765339	-3.668413
H	1.908852	-6.528514	0.580363
H	2.568380	-5.508162	-3.371086
C	-3.747715	-2.861584	-4.159352
C	-5.085183	-2.553273	-4.453134
C	-2.751461	-2.222041	-4.915446
C	-5.374151	-1.612343	-5.443456
C	-3.121411	-1.284818	-5.876494
N	-4.406798	-0.972564	-6.124697
H	-5.896162	-3.035341	-3.907174
H	-1.698790	-2.441615	-4.738078
H	-6.396875	-1.330447	-5.704319
H	-2.376444	-0.748340	-6.466713
Ni	-4.812505	0.517546	-7.417525
N	-1.368721	4.531132	-2.738872
N	-3.547181	4.594665	-1.842544
C	-2.673763	4.239457	-2.788249
C	-0.949426	5.180051	-1.642035
N	-1.759777	5.572543	-0.648636
C	-3.063127	5.282728	-0.797946
C	-4.051792	5.723971	0.238410
C	-3.689741	6.490752	1.357299
C	-5.407673	5.374769	0.107047
C	-6.317435	5.776504	1.080301
N	-5.957933	6.500922	2.154517
H	-2.655820	6.803981	1.505653
H	-5.748811	4.796221	-0.750093
H	-4.440471	7.457027	3.173838
H	-7.374085	5.516642	1.009394
C	0.524750	5.447923	-1.534971
C	1.132397	5.832780	-0.328701
C	1.359061	5.301209	-2.655407
C	2.516028	6.026444	-0.287086

C	2.727319	5.540236	-2.536467
N	3.304631	5.881921	-1.368992
H	0.534586	5.975784	0.572216
H	0.941188	5.020463	-3.621881
H	3.017323	6.319349	0.636325
H	3.400206	5.486275	-3.394133
C	-3.186690	3.436455	-3.945643
C	-4.486032	2.902853	-3.924646
C	-2.391678	3.163548	-5.069661
C	-4.911278	2.097364	-4.977658
C	-2.899943	2.363715	-6.095841
N	-4.129251	1.821133	-6.037202
H	-5.150752	3.103260	-3.084898
H	-1.383546	3.571142	-5.146047
H	-5.904137	1.644604	-4.984815
H	-2.325489	2.126484	-6.994416
Ni	5.274674	6.424003	-1.298799
Ni	-8.677604	-6.492335	2.722961
Ni	4.227462	-6.798071	-1.273449
N	-7.990297	-1.163059	7.088453
N	-7.704923	1.167313	7.173452
C	-7.822194	-0.025886	7.791443
C	-8.112002	-1.054372	5.771451
N	-8.099647	0.105681	5.089460
C	-7.865277	1.183842	5.856280
C	-7.750373	2.533144	5.212894
C	-6.980767	3.530325	5.834024
C	-8.415808	2.870150	4.026187
C	-6.889541	4.787402	5.248718
C	-8.313343	4.170953	3.529595
N	-7.549966	5.109211	4.119583
H	-6.465924	3.320867	6.772190
H	-9.042764	2.136534	3.517519
H	-6.286043	5.576977	5.696975
H	-8.877038	4.498739	2.654913
C	-8.239697	-2.343822	5.016637
C	-8.494160	-2.391210	3.637100
C	-8.084406	-3.565650	5.690500
C	-8.582218	-3.628769	3.002974
C	-8.174149	-4.760342	4.983096
N	-8.419154	-4.794420	3.659254
H	-8.628565	-1.467387	3.073512
H	-7.894535	-3.576665	6.763784
H	-8.791720	-3.706475	1.934638
H	-8.045058	-5.726273	5.471132
C	-7.760847	-0.085313	9.256964
C	-7.614088	1.087643	10.020794
C	-7.866040	-1.312717	9.937180
C	-7.570993	0.974676	11.413207
C	-7.810355	-1.307575	11.333965
N	-7.661365	-0.193146	12.053142
H	-7.544787	2.062678	9.538385
H	-7.993649	-2.246604	9.389936

H	-7.461314	1.867324	12.038725
H	-7.891843	-2.244707	11.895370
Ni	15.351737	-1.217643	2.881253
N	8.721156	-1.685046	0.621432
N	8.942376	0.665616	0.577790
C	9.424660	-0.560765	0.839734
C	7.534793	-1.540824	0.022431
N	7.022829	-0.358887	-0.349595
C	7.741211	0.720944	-0.008414
C	7.141675	2.068867	-0.289733
C	7.542308	3.212539	0.417362
C	6.134191	2.230905	-1.255838
C	-4.668971	6.861104	2.286930
C	5.598972	3.497547	-1.485392
N	5.978688	4.588105	-0.791850
H	8.318230	3.149623	1.181424
H	5.781356	1.376065	-1.832872
H	4.837249	3.654346	-2.251066
C	6.715385	-2.771396	-0.243917
C	6.912186	-3.954976	0.483399
C	5.708270	-2.780559	-1.223757
C	6.114560	-5.070230	0.218739
C	4.980649	-3.948298	-1.451255
N	5.171404	-5.078595	-0.744061
H	7.675544	-4.008806	1.260490
H	5.507041	-1.887420	-1.815536
H	6.225250	-5.992390	0.788313
H	4.216250	-3.989522	-2.228970
C	10.817216	-0.689102	1.380510
C	11.658703	0.427556	1.529036
C	11.344595	-1.944476	1.729933
C	12.965154	0.251190	1.994364
C	12.655837	-2.033993	2.198328
N	13.457347	-0.957747	2.322329
H	11.305417	1.427229	1.275131
H	10.741739	-2.847316	1.634271
H	13.650210	1.093411	2.117093
H	13.084652	-2.996506	2.486096
Ni	-7.416549	7.001083	3.488321
C	6.928448	4.438934	0.152606
H	7.194804	5.336891	0.709345
C	14.786985	-2.241655	7.462562
C	13.711528	-1.683636	6.785951
C	13.814604	-1.416220	5.409388
N	14.920971	-1.683273	4.712888
C	15.992664	-2.211564	5.372998
C	15.980209	-2.522279	6.755624
H	14.724130	-2.457136	8.532450
H	12.783959	-1.445335	7.308973
H	12.982133	-0.970999	4.863145
C	19.450763	-3.173429	4.359184
C	18.323677	-2.981178	5.195884
C	17.167478	-2.426099	4.598608

N	17.096792	-2.059748	3.286727
C	18.173112	-2.256230	2.522350
C	19.364929	-2.814419	3.023416
H	20.371674	-3.599608	4.766328
H	18.106755	-1.968796	1.473631
H	20.211254	-2.954053	2.347964
C	-13.153432	-6.140555	-0.154506
C	-12.616959	-4.888156	0.082476
C	-11.309721	-4.806522	0.603751
N	-10.572262	-5.864492	0.914054
C	-11.094007	-7.101429	0.713714
C	-12.387572	-7.295110	0.142003
H	-14.157417	-6.252371	-0.572492
H	-13.182734	-3.980160	-0.134535
H	-10.858052	-3.822877	0.778729
C	-9.965071	-10.681360	0.960063
C	-10.790330	-9.559480	0.708174
C	-10.305844	-8.266476	1.066338
N	-9.114228	-8.129638	1.727614
C	-8.345056	-9.203237	1.921954
C	-8.721257	-10.497358	1.535300
H	-10.317343	-11.681152	0.692384
H	-7.402381	-9.034172	2.440234
H	-8.050785	-11.336807	1.726257
C	-6.027970	3.651787	-10.781612
C	-4.846133	2.895512	-10.967769
C	-4.376617	2.086136	-9.889654
N	-5.110187	1.979058	-8.737674
C	-6.205151	2.728247	-8.581279
C	-6.690948	3.594778	-9.569792
H	-6.400444	4.279592	-11.595638
H	-6.739173	2.612889	-7.639000
H	-7.592337	4.180848	-9.383206
C	-1.210757	0.755587	-11.419066
C	-0.697304	0.078892	-10.328309
C	-1.417591	0.118148	-9.117877
N	-2.577875	0.742631	-8.966936
C	-3.107290	1.396814	-10.032978
C	-2.435609	1.456033	-11.292419
H	-0.681615	0.765023	-12.375660
H	0.245853	-0.466736	-10.393355
H	-1.013968	-0.394197	-8.235808
C	-10.836832	8.491047	6.461690
C	-11.188123	8.238866	5.114451
C	-10.201698	7.692473	4.236637
N	-8.927006	7.474615	4.689260
C	-8.632666	7.705145	5.972021
C	-9.558238	8.202253	6.898592
H	-11.582395	8.907254	7.144479
H	-7.608063	7.502890	6.279196
H	-9.254176	8.369907	7.933141
C	-12.258224	7.385731	1.100752
C	-11.326853	6.790803	0.271183

C	-10.054846	6.498273	0.800384
N	-9.687538	6.772381	2.044763
C	-10.583168	7.370688	2.871641
C	-11.909462	7.685376	2.440416
H	-13.261306	7.627115	0.739474
H	-11.564098	6.549621	-0.766542
H	-9.304756	6.021488	0.157166
C	4.717332	-9.693935	3.225892
C	3.813793	-8.660082	3.386901
C	3.702756	-7.699401	2.360065
N	4.405020	-7.736961	1.236100
C	5.306182	-8.738081	1.067228
C	5.509632	-9.751051	2.052062
H	4.840706	-10.458424	3.997619
H	3.204227	-8.576632	4.288673
H	3.010463	-6.857722	2.481582
C	8.017188	-9.673159	-1.369578
C	7.152765	-9.735354	-0.250462
C	6.124471	-8.753089	-0.130479
N	5.940109	-7.816961	-1.114183
C	6.800445	-7.757156	-2.134003
C	7.862879	-8.657617	-2.295310
H	8.806936	-10.420725	-1.483301
H	6.622404	-6.973950	-2.868724
H	8.528257	-8.561733	-3.154982
C	6.227792	9.227568	3.186585
C	5.204896	8.316234	3.371434
C	4.953952	7.373168	2.353049
N	5.632035	7.317914	1.215161
C	6.647396	8.198092	1.022126
C	6.996994	9.180214	1.997139
H	6.461819	9.973222	3.951075
H	4.607616	8.313611	4.285189
H	4.163952	6.625839	2.494201
C	9.404500	8.776514	-1.472926
C	8.578057	8.950705	-0.337099
C	7.435543	8.107925	-0.192425
N	7.111892	7.203431	-1.169702
C	7.936866	7.031864	-2.205986
C	9.103155	7.787501	-2.391175
H	10.280721	9.416915	-1.605477
H	7.644044	6.278343	-2.935035
H	9.733456	7.605637	-3.263280
C	-12.855571	8.275714	3.341941
C	-12.510394	8.528190	4.635575
C	-12.872314	-8.617161	-0.134456
C	-12.090355	-9.706203	0.114743
C	-2.979705	2.220362	-12.377804
C	-4.129642	2.933512	-12.211954
C	18.288434	-3.306064	6.597798
C	17.165416	-3.081872	7.347928
C	8.108025	10.061626	1.778929
C	8.877716	9.940542	0.659545

C	6.504268	-10.766993	1.858705
C	7.304442	-10.750438	0.754311
H	8.342016	10.814943	2.535021
H	9.741692	10.591456	0.505998
H	17.156588	-3.324089	8.413140
H	19.182941	-3.732262	7.058566
H	-13.868257	-8.735643	-0.567846
H	-12.444580	-10.711585	-0.124823
H	8.081555	-11.506547	0.619647
H	6.626414	-11.539816	2.621428
H	-2.445927	2.240534	-13.330918
H	-4.528753	3.543038	-13.026182
H	-13.234116	8.960068	5.330776
H	-13.859577	8.507230	2.978689
C	-10.205593	0.583924	-7.770250
C	-9.270919	-0.053137	-8.623316
C	-7.910996	-0.115965	-8.190080
N	-7.519396	0.365584	-6.981914
C	-8.428997	0.931243	-6.199595
C	-9.785219	1.081861	-6.551032
H	-11.250635	0.664720	-8.081014
H	-8.084083	1.296548	-5.224355
H	-10.482739	1.570748	-5.868415
C	-6.448822	-2.108601	-11.029816
C	-5.129899	-2.176130	-10.620799
C	-4.747628	-1.466256	-9.474714
N	-5.602354	-0.754577	-8.734294
C	-6.934159	-0.749768	-9.056727
C	-7.386784	-1.391975	-10.248971
H	-6.779024	-2.609548	-11.943887
H	-4.384666	-2.739478	-11.184719
H	-3.705006	-1.447910	-9.160367
C	-8.766270	-1.308669	-10.639743
C	-9.671579	-0.642329	-9.868524
H	-9.075462	-1.789241	-11.570995
H	-10.717703	-0.571003	-10.175680
C	-4.829422	10.035085	7.127013
C	-5.610922	10.455548	6.021992
C	-5.980305	9.469749	5.057185
N	-5.562270	8.181421	5.150780
C	-4.804959	7.831709	6.182870
C	-4.423039	8.716424	7.212045
H	-4.548825	10.760300	7.895498
H	-4.464572	6.789594	6.214149
H	-3.815037	8.362741	8.046935
C	-7.814870	11.652538	2.600325
C	-8.185114	10.702669	1.666654
C	-7.910032	9.355020	1.937439
N	-7.269904	8.943809	3.035286
C	-6.802119	9.870387	3.929764
C	-7.107253	11.254215	3.759562
H	-8.057147	12.708409	2.452708
H	-8.714778	10.975770	0.752499

H	-8.252658	8.574578	1.259312
C	-6.705247	12.212511	4.751071
C	-6.004965	11.824948	5.854868
H	-6.978603	13.260207	4.605261
H	-5.715627	12.555061	6.614454
C	4.381251	10.891040	-2.649757
C	4.864530	9.980298	-3.619305
C	4.996262	8.605554	-3.257722
N	4.722981	8.199972	-1.977934
C	4.248775	9.078694	-1.091344
C	4.047381	10.433614	-1.388266
H	4.275930	11.948074	-2.908609
H	4.043985	8.694970	-0.091987
H	3.662519	11.106087	-0.619847
C	6.253334	7.196942	-6.509607
C	6.304829	5.856802	-6.172194
C	5.886604	5.475074	-4.881002
N	5.465269	6.333045	-3.961673
C	5.428850	7.654896	-4.265923
C	5.797309	8.141709	-5.556447
H	6.558572	7.538310	-7.502386
H	6.655165	5.107008	-6.884085
H	5.906203	4.417527	-4.591356
C	5.696282	9.539123	-5.866072
C	5.228872	10.421102	-4.937035
H	5.988216	9.883254	-6.861175
H	5.133164	11.482631	-5.177316
C	-7.152048	-10.130528	6.354829
C	-8.454436	-9.642669	6.085836
C	-8.577022	-8.542605	5.183350
N	-7.494130	-7.948472	4.618630
C	-6.290125	-8.420125	4.912438
C	-6.061511	-9.516558	5.767725
H	-7.023275	-10.977180	7.034245
H	-5.433716	-7.913146	4.451952
H	-5.044493	-9.859776	5.965892
C	-12.285482	-7.949523	5.424698
C	-12.404338	-6.855233	4.588073
C	-11.270029	-6.416396	3.891922
N	-10.070039	-6.990501	4.011629
C	-9.898910	-8.014411	4.905059
C	-11.017924	-8.550720	5.610655
H	-13.154015	-8.345286	5.958001
H	-13.358550	-6.345305	4.445873
H	-11.339008	-5.579825	3.199312
C	-10.847761	-9.666186	6.498998
C	-9.614193	-10.204732	6.716054
H	-11.728059	-10.070332	7.004373
H	-9.489522	-11.052799	7.393506
C	5.176242	-7.606106	-6.482785
C	4.582657	-8.497297	-5.554129
C	4.268235	-7.989888	-4.257118
N	4.484589	-6.692377	-3.924253

C	5.033122	-5.883517	-4.820658
C	5.409280	-6.293280	-6.116146
H	5.443854	-7.966960	-7.479518
H	5.198218	-4.845580	-4.507367
H	5.869698	-5.585549	-6.808123
C	2.783054	-11.088835	-2.715864
C	2.500937	-10.616352	-1.447411
C	2.872938	-9.304642	-1.122548
N	3.464728	-8.477988	-1.988105
C	3.698149	-8.892319	-3.273040
C	3.390260	-10.230689	-3.663360
H	2.543573	-12.117976	-2.996776
H	2.026535	-11.247971	-0.694561
H	2.710042	-8.916475	-0.117313
C	3.706535	-10.690957	-4.986762
C	4.297307	-9.861796	-5.893812
H	3.473432	-11.725640	-5.249105
H	4.551719	-10.222854	-6.893210
C	18.021905	3.049911	3.440009
C	17.871742	2.303709	2.243424
C	16.940812	1.229925	2.261794
N	16.186925	0.928108	3.348367
C	16.349484	1.644034	4.452949
C	17.264289	2.715311	4.549090
H	18.738492	3.874652	3.479323
H	15.735130	1.364462	5.315566
H	17.365740	3.263491	5.487971
C	17.374657	-0.135781	-1.210615
C	16.512094	-1.219573	-1.138460
C	15.838271	-1.472384	0.066183
N	15.980695	-0.692882	1.140072
C	16.796642	0.403335	1.087632
C	17.541644	0.708524	-0.087080
H	17.940493	0.064678	-2.124444
H	16.375365	-1.892101	-1.986917
H	15.189895	-2.344800	0.176273
C	18.450480	1.821956	-0.077722
C	18.618968	2.580011	1.046674
H	19.023297	2.037361	-0.982650
H	19.332295	3.407861	1.052092

162

CAGEphen+phen+12PdDividedBy3big SCF Done: -4581.86473708 A.U.

Pd	8.065066	0.576892	0.436993
N	1.681891	-2.510834	0.174811
N	0.753385	-4.614201	-0.371719
C	0.638391	-3.339378	-0.004274
C	2.880837	-3.057247	-0.061287
N	3.087394	-4.313719	-0.442000
C	1.992913	-5.084931	-0.581588
C	2.162555	-6.495261	-0.981253
C	1.049357	-7.332499	-1.163622
C	3.441205	-7.039496	-1.187913
C	1.267957	-8.659844	-1.542714

C	3.542306	-8.381127	-1.565442
N	2.483516	-9.175073	-1.740528
H	0.038647	-6.953856	-1.012304
H	4.333803	-6.428732	-1.053048
H	0.419228	-9.336206	-1.692475
H	4.526059	-8.833325	-1.733213
C	4.090382	-2.193066	0.092797
C	5.329689	-2.614478	-0.413683
C	4.028818	-0.947170	0.728920
C	6.436124	-1.784584	-0.290068
C	5.179571	-0.169962	0.819672
N	6.359604	-0.578842	0.312919
H	5.418746	-3.582005	-0.907634
H	3.088313	-0.581212	1.138476
H	7.414606	-2.069154	-0.679973
H	5.155544	0.813083	1.292835
C	-0.739896	-2.810459	0.206765
C	-1.854603	-3.636194	-0.001895
C	-0.971525	-1.486392	0.609218
C	-3.131373	-3.105112	0.190954
C	-2.290461	-1.054713	0.772916
N	-3.356806	-1.839410	0.566088
H	-1.719508	-4.672636	-0.310832
H	-0.136329	-0.809082	0.788979
H	-4.012321	-3.736651	0.032157
H	-2.491152	-0.021892	1.076882
Pd	-8.090434	-0.498822	-0.222249
N	-1.863487	2.662623	-0.816882
N	-3.207089	4.581810	-0.482093
C	-3.041927	3.276469	-0.665731
C	-0.791797	3.468871	-0.756264
N	-0.860715	4.787600	-0.583032
C	-2.084817	5.326921	-0.458281
C	-2.205920	6.787402	-0.285220
C	-3.465275	7.401534	-0.181995
C	-1.064199	7.603590	-0.222566
C	-1.236952	8.980510	-0.057722
N	-2.434206	9.562592	0.042145
H	-4.378737	6.808994	-0.231236
H	-0.067166	7.170480	-0.301047
H	-0.365254	9.642052	-0.004609
C	0.559196	2.851287	-0.875880
C	0.716552	1.477024	-1.110340
C	1.717219	3.631579	-0.745817
C	2.007730	0.952066	-1.199266
C	2.961145	3.005568	-0.848887
N	3.114890	1.694246	-1.069719
H	-0.155474	0.832378	-1.219882
H	1.639654	4.703762	-0.564609
H	2.147708	-0.120105	-1.368884
H	3.877879	3.597567	-0.747955
C	-4.265589	2.420526	-0.674244
C	-5.505783	2.928083	-0.256782

C	-4.197719	1.077631	-1.066053
C	-6.604714	2.078293	-0.202070
C	-5.339006	0.288372	-0.994610
N	-6.516608	0.779543	-0.559415
H	-5.598161	3.972300	0.042803
H	-3.255710	0.647675	-1.402503
H	-7.580715	2.418392	0.150276
H	-5.305619	-0.767570	-1.264665
C	-3.520100	8.788644	-0.020460
H	-4.487943	9.295538	0.061192
C	-9.877881	-0.965483	-4.699265
C	-8.798465	-0.115675	-4.522787
C	-8.232160	0.032981	-3.242958
N	-8.700590	-0.624712	-2.183071
C	-9.768565	-1.463362	-2.336302
C	-10.398471	-1.671246	-3.587373
H	-10.332144	-1.093532	-5.684975
H	-8.377935	0.444793	-5.359336
H	-7.383741	0.699098	-3.078294
C	-11.779051	-3.647251	-0.066267
C	-11.354533	-3.015981	-1.260901
C	-10.249637	-2.135417	-1.169421
N	-9.606306	-1.885212	0.008495
C	-10.022666	-2.490539	1.118469
C	-11.110977	-3.383522	1.117772
H	-12.627495	-4.335797	-0.087216
H	-9.483997	-2.265956	2.039989
H	-11.412273	-3.856063	2.054138
C	9.656588	-2.105166	4.171602
C	8.670389	-1.146326	4.314485
C	8.247771	-0.445543	3.176842
N	8.749273	-0.672339	1.957574
C	9.708113	-1.633457	1.774836
C	10.202931	-2.369607	2.894557
H	10.023743	-2.661538	5.037730
H	8.229143	-0.916469	5.285454
H	7.486588	0.333054	3.260256
C	11.824960	-3.102464	-0.965273
C	11.296852	-2.874730	0.330128
C	10.253580	-1.910802	0.455311
N	9.755815	-1.253750	-0.616011
C	10.266217	-1.487392	-1.814778
C	11.315705	-2.403722	-2.043260
H	12.629581	-3.830223	-1.097602
H	9.829958	-0.931613	-2.652191
H	11.702335	-2.555043	-3.052881
C	11.240899	-3.345740	2.714847
C	11.775309	-3.582055	1.483158
C	-11.976886	-3.215477	-2.542136
C	-11.518871	-2.570321	-3.657592
H	-12.001589	-2.728145	-4.624571
H	-12.829373	-3.895161	-2.608051
H	11.599120	-3.889853	3.591448

H	12.572773	-4.317786	1.355562
C	7.109401	3.891657	-2.962003
C	8.318540	3.870696	-2.224254
C	8.453486	2.868955	-1.233030
N	7.474206	1.947004	-0.981142
C	6.339628	1.993398	-1.678287
C	6.125891	2.959482	-2.680942
H	6.963054	4.644491	-3.740589
H	5.548114	1.280685	-1.437812
H	5.176900	2.950405	-3.218892
C	11.837846	3.605060	0.173129
C	11.886737	2.602214	1.126682
C	10.800380	1.717032	1.261188
N	9.713771	1.813511	0.498210
C	9.644051	2.798435	-0.444451
C	10.691804	3.728321	-0.650030
H	12.671716	4.301007	0.051852
H	12.753346	2.483949	1.779297
H	10.819081	0.917910	2.003214
C	10.536146	4.732177	-1.667669
C	9.398009	4.799838	-2.422858
H	11.348335	5.445051	-1.826363
H	9.290850	5.567497	-3.192388
C	-11.375555	2.157876	3.156876
C	-10.239729	1.384688	3.504935
C	-9.437733	0.877718	2.440765
N	-9.714359	1.148079	1.145159
C	-10.778257	1.877518	0.848913
C	-11.653079	2.402143	1.825469
H	-12.017328	2.557082	3.946378
H	-10.961667	2.071810	-0.213620
H	-12.519054	2.994811	1.524485
C	-6.756790	-0.864683	4.433196
C	-5.973334	-1.358503	3.407019
C	-6.392485	-1.163857	2.082949
N	-7.514190	-0.503262	1.775988
C	-8.275515	0.065612	2.763408
C	-7.930062	-0.133064	4.133939
H	-6.477255	-1.022716	5.477925
H	-5.048604	-1.905503	3.595264
H	-5.778455	-1.545680	1.263279
C	-8.763251	0.406745	5.171768
C	-9.882785	1.124332	4.870147
H	-8.480598	0.226477	6.211223
H	-10.516776	1.524678	5.664709

126

CAGEphen+phen+12PdDividedBy3 SCF Done: -3560.85024034 A.U.

N	-0.000043	1.083944	-0.000280
N	-1.174973	3.138467	0.031607
C	-1.128277	1.810225	0.030417
C	1.128189	1.810223	-0.031107
N	1.174891	3.138463	-0.032540
C	-0.000041	3.792679	-0.000529

C	-0.000038	5.265709	-0.000644
C	1.204288	5.988362	-0.055252
C	-1.204359	5.988378	0.053858
C	1.145164	7.384645	-0.053341
C	-1.145226	7.384660	0.051724
N	-0.000029	8.068382	-0.000860
H	2.162913	5.471911	-0.099602
H	-2.162987	5.471941	0.098303
H	2.066617	7.975561	-0.096347
H	-2.066674	7.975589	0.094657
C	2.446885	1.100915	-0.066970
C	3.640333	1.839425	-0.081057
C	2.544184	-0.298199	-0.088010
C	4.861705	1.174845	-0.113858
C	3.802793	-0.894817	-0.124086
N	4.942353	-0.170937	-0.138898
H	3.609664	2.928585	-0.065313
H	1.645228	-0.914809	-0.076706
H	5.809736	1.715767	-0.120597
H	3.908419	-1.981744	-0.145624
C	-2.446970	1.100911	0.066410
C	-3.640433	1.839407	0.080302
C	-2.544256	-0.298196	0.087820
C	-4.861796	1.174824	0.113279
C	-3.802862	-0.894820	0.124066
N	-4.942431	-0.170956	0.138702
H	-3.609778	2.928563	0.064283
H	-1.645299	-0.914806	0.076675
H	-5.809832	1.715736	0.119880
H	-3.908465	-1.981742	0.145899
Pd	-6.812141	-1.081892	0.140861
Pd	6.812079	-1.081864	-0.140549
C	-10.961846	-3.520030	0.723057
C	-10.044704	-3.297236	1.779231
C	-8.862319	-2.582631	1.470016
N	-8.595368	-2.114143	0.215342
C	-9.474046	-2.332675	-0.761479
C	-10.672871	-3.037068	-0.542215
H	-11.887815	-4.068602	0.913124
H	-9.229386	-1.942789	-1.750056
H	-11.358294	-3.192817	-1.377050
C	-7.132314	-2.449733	4.778671
C	-6.010145	-1.733207	4.396463
C	-5.864596	-1.331283	3.055955
N	-6.776584	-1.619792	2.128229
C	-7.895791	-2.317494	2.488763
C	-8.121009	-2.762253	3.814620
H	-7.263483	-2.771331	5.814903
H	-5.234628	-1.471877	5.118198
H	-4.990322	-0.763890	2.733441
C	7.256072	-0.455556	4.657483
C	6.459038	-1.447255	4.114811
C	6.348678	-1.529826	2.720300

N	6.971053	-0.682299	1.893145
C	7.746590	0.329468	2.396196
C	7.923548	0.456882	3.808205
H	7.382144	-0.371058	5.739877
H	5.934495	-2.169627	4.742012
H	5.749751	-2.318522	2.259919
C	9.942867	3.138726	1.200842
C	9.274644	2.257841	2.087884
C	8.420095	1.270345	1.515604
N	8.218379	1.186214	0.181035
C	8.855041	2.022517	-0.624326
C	9.741460	3.019020	-0.161037
H	10.611239	3.903717	1.604163
H	8.662362	1.915216	-1.697518
H	10.244340	3.679042	-0.870446
C	-9.326588	-3.489872	4.106461
C	-10.248671	-3.747379	3.129901
C	8.775800	1.483776	4.338629
C	9.435233	2.343634	3.511185
H	10.093205	3.113593	3.920834
H	8.892522	1.553581	5.422288
H	-9.494662	-3.833286	5.129578
H	-11.161517	-4.299794	3.363700
C	-7.256224	-0.458477	-4.657563
C	-7.923572	0.454565	-3.808833
C	-7.746651	0.327962	-2.396747
N	-6.971231	-0.683587	-1.893083
C	-6.348974	-1.531697	-2.719730
C	-6.459333	-1.449962	-4.114290
H	-7.382276	-0.374607	-5.740008
H	-5.750141	-2.320191	-2.258881
H	-5.934887	-2.172787	-4.741053
C	-9.942444	3.138324	-1.203092
C	-9.741117	3.019355	0.158862
C	-8.854958	2.022904	0.622761
N	-8.218436	1.186009	-0.182097
C	-8.420048	1.269449	-1.516724
C	-9.274381	2.256783	-2.089603
H	-10.610639	3.903223	-1.606878
H	-10.243879	3.679894	0.867874
H	-8.662397	1.916129	1.696027
C	-9.434929	2.341762	-3.512958
C	-8.775649	1.481283	-4.339878
H	-10.092750	3.111600	-3.923076
H	-8.892354	1.550456	-5.423579
C	7.132712	-2.452420	-4.777498
C	8.121259	-2.764464	-3.813137
C	7.895908	-2.318924	-2.487566
N	6.776699	-1.620938	-2.127584
C	5.864849	-1.332899	-3.055586
C	6.010543	-1.735596	-4.395847
H	7.263992	-2.774626	-5.813527
H	4.990570	-0.765268	-2.733498

H	5.235137	-1.474623	-5.117831
C	10.961642	-3.520712	-0.720788
C	10.672551	-3.037009	0.544174
C	9.473762	-2.332383	0.762889
N	8.595227	-2.114321	-0.214166
C	8.862291	-2.583552	-1.468543
C	10.044651	-3.298440	-1.777202
H	11.887585	-4.069477	-0.910429
H	11.357855	-3.192343	1.379183
H	9.229019	-1.941923	1.751217
C	10.248751	-3.749376	-3.127587
C	9.326814	-3.492349	-4.104411
H	11.161577	-4.302007	-3.360952
H	9.494984	-3.836365	-5.127311

414

CAGEphen+phen+12Pd SCF Done: -11702.1785047 A.U.

N	-2.148480	-4.212583	-2.877914
N	-4.416046	-4.275847	-2.238890
C	-3.431668	-3.870350	-3.045574
C	-1.869370	-4.957793	-1.799429
N	-2.784029	-5.338192	-0.896482
C	-4.056615	-5.001772	-1.169583
C	-5.152606	-5.423417	-0.239708
C	-4.899250	-5.837019	1.078006
C	-6.492431	-5.392058	-0.661353
C	-5.972659	-6.144264	1.917914
C	-7.508187	-5.746062	0.226560
N	-7.254025	-6.090417	1.504167
H	-3.878379	-5.902502	1.455435
H	-6.747387	-5.102319	-1.680477
H	-5.807281	-6.440052	2.956388
H	-8.563002	-5.764654	-0.064168
C	-0.437054	-5.373632	-1.619651
C	0.071333	-5.826139	-0.391574
C	0.453345	-5.326390	-2.704887
C	1.420462	-6.178822	-0.292810
C	1.777135	-5.729740	-2.536432
N	2.262344	-6.135047	-1.345668
H	-0.575155	-5.900932	0.483654
H	0.109586	-5.003740	-3.687650
H	1.841616	-6.518894	0.656179
H	2.475519	-5.774234	-3.374748
C	-3.776320	-2.940329	-4.168240
C	-5.107226	-2.603321	-4.458655
C	-2.770264	-2.335213	-4.939058
C	-5.381025	-1.667539	-5.458125
C	-3.121320	-1.401334	-5.910820
N	-4.401655	-1.057495	-6.152795
H	-5.926891	-3.060831	-3.904617
H	-1.722164	-2.580112	-4.767963
H	-6.400663	-1.374989	-5.722793
H	-2.359987	-0.901893	-6.514084
Pd	-4.822996	0.499789	-7.499114

N	-1.385920	4.590517	-2.759543
N	-3.571866	4.672908	-1.882743
C	-2.692440	4.305754	-2.818173
C	-0.972683	5.238412	-1.659998
N	-1.787702	5.636664	-0.673076
C	-3.092432	5.360059	-0.835552
C	-4.087223	5.817897	0.186875
C	-3.727622	6.576659	1.311664
C	-5.447713	5.497382	0.033848
C	-6.365810	5.915957	0.992074
N	-6.007248	6.630092	2.076043
H	-2.690541	6.871170	1.475543
H	-5.788119	4.928933	-0.830288
H	-4.482902	7.564440	3.113884
H	-7.426918	5.679247	0.893221
C	0.501566	5.499252	-1.541263
C	1.109462	5.815856	-0.315535
C	1.334333	5.414302	-2.668774
C	2.493736	5.999586	-0.260587
C	2.703446	5.645050	-2.540483
N	3.281075	5.915277	-1.352295
H	0.512160	5.910240	0.592149
H	0.915542	5.190252	-3.649714
H	2.990975	6.230155	0.684477
H	3.371759	5.650809	-3.404667
C	-3.200457	3.497408	-3.973638
C	-4.511484	2.993436	-3.971176
C	-2.391521	3.191782	-5.078778
C	-4.937113	2.183557	-5.020925
C	-2.896504	2.388808	-6.104090
N	-4.139292	1.871724	-6.060926
H	-5.188699	3.221840	-3.148819
H	-1.374013	3.577620	-5.142901
H	-5.945147	1.763240	-5.036121
H	-2.311506	2.139383	-6.993204
Pd	5.334233	6.437451	-1.285693
Pd	-8.866472	-6.490696	2.808630
Pd	4.223696	-6.937955	-1.233991
N	-8.301803	-1.003272	7.194295
N	-7.994248	1.325230	7.269037
C	-8.145422	0.138637	7.892532
C	-8.372843	-0.905481	5.872785
N	-8.320776	0.247814	5.181794
C	-8.105120	1.330845	5.946768
C	-7.954608	2.670831	5.292107
C	-7.218924	3.678032	5.937309
C	-8.552741	2.986566	4.064495
C	-7.091058	4.924842	5.336104
C	-8.420012	4.276674	3.548812
N	-7.686869	5.224163	4.164064
H	-6.756211	3.484667	6.905604
H	-9.151858	2.244045	3.535751
H	-6.507127	5.718202	5.807182

H	-8.937275	4.584236	2.638418
C	-8.487271	-2.198483	5.122468
C	-8.669676	-2.249228	3.731843
C	-8.391492	-3.418302	5.809752
C	-8.744926	-3.487331	3.097575
C	-8.463011	-4.616980	5.105982
N	-8.635426	-4.652564	3.768986
H	-8.758279	-1.326670	3.157432
H	-8.259360	-3.426850	6.891714
H	-8.901861	-3.559069	2.019022
H	-8.375889	-5.584512	5.603663
C	-8.134701	0.091601	9.359848
C	-8.001778	1.269597	10.118352
C	-8.274682	-1.128764	10.046345
C	-8.006691	1.168335	11.512323
C	-8.266052	-1.111971	11.444076
N	-8.130595	0.007128	12.158669
H	-7.905995	2.239680	9.630566
H	-8.392325	-2.066130	9.502860
H	-7.908692	2.065324	12.133538
H	-8.375348	-2.043471	12.010100
Pd	15.501906	-1.328387	3.105822
N	8.764192	-1.762361	0.718763
N	9.000170	0.587422	0.662750
C	9.473047	-0.640716	0.933412
C	7.575901	-1.614002	0.124959
N	7.066193	-0.429652	-0.240761
C	7.794702	0.647482	0.085622
C	7.201301	1.995672	-0.206291
C	7.661789	3.158927	0.427992
C	6.136778	2.137533	-1.112428
C	-4.712089	6.965872	2.227941
C	5.604134	3.401355	-1.359079
N	6.048248	4.513441	-0.738713
H	8.481305	3.112336	1.146273
H	5.734875	1.266967	-1.630356
H	4.793329	3.534250	-2.078150
C	6.752900	-2.840459	-0.147609
C	6.986168	-4.049257	0.524741
C	5.704711	-2.819187	-1.083056
C	6.186494	-5.161361	0.251072
C	4.971660	-3.980011	-1.324492
N	5.202598	-5.137310	-0.671977
H	7.780724	-4.127134	1.267637
H	5.473718	-1.905632	-1.630763
H	6.322597	-6.103110	0.783829
H	4.172125	-3.990080	-2.068117
C	10.861446	-0.776616	1.482926
C	11.721034	0.327932	1.608955
C	11.365700	-2.031287	1.865888
C	13.022030	0.144340	2.088868
C	12.670614	-2.131881	2.348154
N	13.489752	-1.065052	2.455801

H	11.388369	1.326593	1.325102
H	10.749787	-2.926746	1.786830
H	13.724786	0.976212	2.186908
H	13.076677	-3.097521	2.657864
Pd	-7.516689	7.182092	3.455373
C	7.054163	4.386374	0.151613
H	7.366176	5.299955	0.658652
C	14.880541	-2.078401	7.857343
C	13.760879	-1.763191	7.103251
C	13.889143	-1.562374	5.717685
N	15.061554	-1.666431	5.091312
C	16.179832	-1.966266	5.820886
C	16.140125	-2.188307	7.221359
H	14.801680	-2.239121	8.935704
H	12.779018	-1.666527	7.569181
H	13.021207	-1.310208	5.106183
C	19.807473	-2.440551	5.060758
C	18.611441	-2.364665	5.816735
C	17.419710	-2.046844	5.119933
N	17.396419	-1.807554	3.773778
C	18.535095	-1.890765	3.083234
C	19.761675	-2.207018	3.696387
H	20.752053	-2.684361	5.554541
H	18.490028	-1.706928	2.010513
H	20.662932	-2.264854	3.082990
C	-13.212758	-6.886915	-0.513621
C	-12.754873	-5.584054	-0.456916
C	-11.482580	-5.348768	0.103757
N	-10.715726	-6.300075	0.616288
C	-11.164618	-7.579375	0.608764
C	-12.411493	-7.933236	0.007479
H	-14.181643	-7.123620	-0.961242
H	-13.350129	-4.756720	-0.848006
H	-11.080281	-4.328333	0.128645
C	-9.921891	-11.020860	1.506371
C	-10.761968	-9.990097	1.023461
C	-10.353821	-8.632089	1.200045
N	-9.220871	-8.349536	1.920679
C	-8.436838	-9.345453	2.346914
C	-8.736104	-10.698167	2.138958
H	-10.220048	-12.064180	1.372927
H	-7.553081	-9.060721	2.917796
H	-8.057450	-11.466848	2.511779
C	-6.319326	3.625661	-10.871667
C	-5.072667	2.988576	-11.075510
C	-4.516079	2.208162	-10.014999
N	-5.237912	2.013470	-8.864795
C	-6.405463	2.643485	-8.693302
C	-6.973572	3.479345	-9.662931
H	-6.751904	4.230601	-11.673117
H	-6.932685	2.449861	-7.759282
H	-7.928604	3.968561	-9.465017
C	-1.276695	1.179183	-11.629871

C	-0.682866	0.529342	-10.564479
C	-1.372664	0.498840	-9.335353
N	-2.573535	1.026815	-9.149024
C	-3.188214	1.640614	-10.190686
C	-2.552012	1.774223	-11.463682
H	-0.773824	1.248239	-12.598096
H	0.299080	0.062449	-10.661683
H	-0.907323	0.017872	-8.465366
C	-10.822434	8.866809	6.586038
C	-11.261157	8.551255	5.278964
C	-10.339988	7.949862	4.363846
N	-9.039906	7.745832	4.752221
C	-8.657625	8.044038	5.999196
C	-9.519538	8.593696	6.955618
H	-11.521204	9.322885	7.292375
H	-7.613195	7.860381	6.248820
H	-9.147910	8.814073	7.957622
C	-12.616052	7.538640	1.391202
C	-11.755438	6.892304	0.525067
C	-10.454301	6.598537	0.979679
N	-9.999946	6.920187	2.181392
C	-10.820659	7.571711	3.041696
C	-12.169926	7.889823	2.689294
H	-13.638920	7.781251	1.091547
H	-12.069613	6.610607	-0.481648
H	-9.756740	6.074174	0.313592
C	5.259041	-10.137963	3.094298
C	4.368441	-9.132106	3.419746
C	4.181722	-8.078764	2.499155
N	4.793401	-8.012303	1.325984
C	5.659191	-8.996394	0.982187
C	5.949591	-10.086825	1.857241
H	5.449376	-10.964876	3.783660
H	3.831964	-9.137094	4.370621
H	3.505668	-7.251866	2.748185
C	8.122669	-9.737284	-1.769057
C	7.373053	-9.878457	-0.577561
C	6.350929	-8.921133	-0.293779
N	6.054652	-7.945397	-1.212694
C	6.803122	-7.816679	-2.314043
C	7.862353	-8.680261	-2.621841
H	8.907738	-10.462496	-1.999638
H	6.528198	-7.016131	-3.000149
H	8.436010	-8.527362	-3.537366
C	6.904342	9.461523	3.005219
C	5.894348	8.585751	3.356957
C	5.544377	7.565924	2.446238
N	6.112235	7.415347	1.259003
C	7.094402	8.272760	0.889934
C	7.551068	9.315228	1.752241
H	7.221410	10.256313	3.685654
H	5.386911	8.664764	4.320410
H	4.768731	6.838857	2.715614

C	9.568400	8.666178	-1.922637
C	8.873957	8.911004	-0.714596
C	7.738273	8.102477	-0.401758
N	7.289253	7.174933	-1.308318
C	7.986916	6.942028	-2.425757
C	9.146465	7.652149	-2.762932
H	10.438950	9.277274	-2.175833
H	7.589279	6.184685	-3.100637
H	9.672086	7.419858	-3.690506
C	-13.045962	8.532380	3.624712
C	-12.610294	8.835833	4.878824
C	-12.819556	-9.305418	-0.083305
C	-12.008475	-10.297758	0.379651
C	-3.177792	2.507135	-12.525888
C	-4.380966	3.115172	-12.327722
C	18.547485	-2.591083	7.235799
C	17.361092	-2.504172	7.911147
C	8.646437	10.157386	1.365772
C	9.296465	9.947211	0.185903
C	6.929725	-11.071461	1.499336
C	7.629859	-10.957518	0.335034
H	8.968073	10.951891	2.043488
H	10.152662	10.564853	-0.095238
H	17.325816	-2.674938	8.989545
H	19.470119	-2.833827	7.768507
H	-13.777997	-9.543013	-0.550963
H	-12.298917	-11.346196	0.280032
H	8.400945	-11.686851	0.075959
H	7.124809	-11.898491	2.186321
H	-2.664525	2.589808	-13.486872
H	-4.845510	3.703086	-13.122828
H	-13.279481	9.307345	5.602219
H	-14.069923	8.761644	3.320487
C	-10.272897	0.148610	-8.107817
C	-9.260338	-0.397084	-8.935697
C	-7.919364	-0.388836	-8.440921
N	-7.623327	0.060042	-7.195684
C	-8.599132	0.543555	-6.440612
C	-9.943925	0.630234	-6.854860
H	-11.305253	0.173140	-8.466733
H	-8.319503	0.884696	-5.435588
H	-10.702495	1.052837	-6.193297
C	-6.208077	-2.130926	-11.311673
C	-4.897676	-2.104526	-10.872620
C	-4.604389	-1.418013	-9.687732
N	-5.538577	-0.823344	-8.937014
C	-6.861198	-0.909525	-9.292528
C	-7.221771	-1.529474	-10.529339
H	-6.472991	-2.614513	-12.255790
H	-4.092890	-2.572562	-11.441640
H	-3.571900	-1.316116	-9.353934
C	-8.588200	-1.534589	-10.971221
C	-9.568764	-0.963970	-10.216541

H	-8.824046	-1.999913	-11.931159
H	-10.604181	-0.953803	-10.565288
C	-4.881461	10.528255	6.881673
C	-5.685888	10.877882	5.768016
C	-6.042807	9.839521	4.853985
N	-5.573266	8.575117	4.994695
C	-4.805754	8.287335	6.036571
C	-4.444794	9.224926	7.026703
H	-4.610611	11.296062	7.611255
H	-4.438797	7.255710	6.105901
H	-3.825525	8.924782	7.874251
C	-8.035926	11.863317	2.377511
C	-8.442245	10.865230	1.511894
C	-8.129788	9.536946	1.829984
N	-7.414078	9.192671	2.905981
C	-6.916003	10.164475	3.736592
C	-7.264569	11.532613	3.516545
H	-8.305411	12.906990	2.194809
H	-9.029591	11.085211	0.619004
H	-8.507982	8.719849	1.215351
C	-6.851455	12.547095	4.445636
C	-6.115291	12.228667	5.547458
H	-7.153498	13.579977	4.257364
H	-5.825591	13.000081	6.264918
C	4.590001	11.134233	-2.165002
C	5.006419	10.329943	-3.251481
C	5.057543	8.910897	-3.079761
N	4.787041	8.368026	-1.848497
C	4.377450	9.152350	-0.845241
C	4.244593	10.541160	-0.965210
H	4.544776	12.219806	-2.286658
H	4.181116	8.663254	0.109093
H	3.908390	11.129207	-0.109839
C	6.164920	7.904536	-6.533866
C	6.115098	6.529819	-6.401424
C	5.672855	5.994459	-5.173858
N	5.330375	6.733660	-4.128741
C	5.402425	8.083521	-4.226092
C	5.791678	8.726976	-5.441394
H	6.482656	8.367943	-7.471698
H	6.397259	5.870224	-7.224368
H	5.598966	4.906986	-5.050298
C	5.783910	10.156953	-5.551941
C	5.381509	10.927163	-4.502449
H	6.085965	10.616981	-6.495891
H	5.346137	12.015424	-4.591836
C	-8.099521	-10.007771	6.995321
C	-9.320248	-9.485641	6.499680
C	-9.249453	-8.427800	5.542049
N	-8.065100	-7.898661	5.149873
C	-6.943645	-8.396281	5.649592
C	-6.902118	-9.465178	6.568427
H	-8.119476	-10.824036	7.722209

H	-6.008415	-7.927413	5.319777
H	-5.946536	-9.837530	6.942488
C	-12.929270	-7.726504	5.139135
C	-12.885547	-6.695909	4.218734
C	-11.644935	-6.332441	3.681013
N	-10.494140	-6.913437	4.040262
C	-10.488700	-7.876233	5.018382
C	-11.727106	-8.339589	5.562655
H	-13.881824	-8.067530	5.553429
H	-13.790159	-6.183484	3.887764
H	-11.584456	-5.562887	2.912110
C	-11.745754	-9.403441	6.527167
C	-10.587007	-9.972222	6.964043
H	-12.711433	-9.745800	6.906308
H	-10.607114	-10.784800	7.694172
C	4.925492	-8.464139	-6.482819
C	4.424104	-9.235151	-5.404256
C	4.115292	-8.554752	-4.185824
N	4.240478	-7.210059	-4.072417
C	4.700146	-6.517760	-5.104483
C	5.073599	-7.098203	-6.334370
H	5.184809	-8.959034	-7.422531
H	4.786192	-5.432686	-4.967510
H	5.458509	-6.477967	-7.146194
C	2.851562	-11.480252	-2.170235
C	2.575721	-10.856179	-0.968236
C	2.897796	-9.500302	-0.831685
N	3.426154	-8.771253	-1.820874
C	3.638109	-9.335468	-3.054076
C	3.391003	-10.731728	-3.242340
H	2.657605	-12.547781	-2.304611
H	2.150157	-11.399741	-0.123292
H	2.757543	-8.997508	0.125351
C	3.695152	-11.364100	-4.495234
C	4.214856	-10.648310	-5.531547
H	3.508768	-12.435758	-4.596978
H	4.461003	-11.137276	-6.477183
C	18.021357	3.329427	2.312202
C	17.910447	2.257723	1.390340
C	17.003116	1.205360	1.710104
N	16.221234	1.240792	2.815825
C	16.344311	2.258107	3.656140
C	17.246679	3.325743	3.456839
H	18.723404	4.143053	2.111641
H	15.702326	2.243296	4.543917
H	17.319684	4.128089	4.193761
C	17.594988	-1.059665	-1.242765
C	16.793367	-2.132957	-0.893799
C	16.117970	-2.094429	0.333503
N	16.188896	-1.049094	1.166307
C	16.928575	0.055427	0.826587
C	17.687201	0.061027	-0.384513
H	18.173295	-1.074548	-2.170465

H	16.711088	-3.015643	-1.529980
H	15.529988	-2.952334	0.667373
C	18.550651	1.167680	-0.688693
C	18.676203	2.214440	0.176872
H	19.127031	1.139842	-1.616203
H	19.361937	3.036270	-0.043175

162

CAGEphen+phen+12ZnDividedBy3big SCF Done: -7884.47984231 A.U.

Zn	9.153648	0.222513	0.566074
N	2.140640	0.588979	-1.016342
N	0.740059	2.021909	-2.275374
C	0.942448	0.963949	-1.494329
C	3.160899	1.373380	-1.394023
N	3.049302	2.442172	-2.176990
C	1.815172	2.754387	-2.609184
C	1.636818	3.935028	-3.474259
C	0.369701	4.278156	-3.975394
C	2.728224	4.749639	-3.820229
C	0.257990	5.407580	-4.790926
C	2.500773	5.856621	-4.642432
N	1.296301	6.179951	-5.118273
H	-0.505911	3.675585	-3.734691
H	3.729496	4.524898	-3.453235
H	-0.716610	5.699692	-5.197163
H	3.331708	6.510981	-4.928055
C	4.544025	1.053779	-0.914716
C	5.630475	1.818296	-1.370185
C	4.807285	0.014659	-0.009818
C	6.910280	1.517692	-0.920144
C	6.120418	-0.209091	0.403101
N	7.157597	0.523728	-0.045579
H	5.466047	2.637295	-2.069796
H	3.993466	-0.604500	0.368671
H	7.775351	2.086740	-1.267649
H	6.360225	-0.997835	1.120423
C	-0.271152	0.160330	-1.145735
C	-1.536838	0.595149	-1.569731
C	-0.203171	-1.032642	-0.411353
C	-2.658951	-0.158758	-1.241837
C	-1.383268	-1.727392	-0.138188
N	-2.593606	-1.298982	-0.534903
H	-1.634568	1.513573	-2.147553
H	0.758331	-1.414025	-0.066460
H	-3.654990	0.159016	-1.556139
H	-1.361624	-2.670310	0.413807
Zn	-4.491301	-2.482470	-0.344737
N	-8.054629	3.402656	1.617394
N	-9.700518	2.392918	0.245968
C	-8.492155	2.449401	0.798414
C	-8.941248	4.381501	1.889572
N	-10.176272	4.405704	1.379488
C	-10.532224	3.406073	0.564441
C	-11.894359	3.411915	-0.011425

C	-12.321076	2.390920	-0.876248
C	-12.799283	4.441337	0.292466
C	-14.074915	4.399131	-0.278594
N	-14.479596	3.428122	-1.100992
H	-11.651998	1.571722	-1.139701
H	-12.510814	5.253757	0.959407
H	-14.800877	5.189399	-0.058244
C	-8.526807	5.478266	2.793527
C	-7.244468	5.501114	3.365139
C	-9.406275	6.526862	3.104158
C	-6.908517	6.561087	4.209874
C	-8.963570	7.538411	3.962489
N	-7.743548	7.559909	4.505205
H	-6.529885	4.706002	3.151972
H	-10.411585	6.549001	2.683407
H	-5.914582	6.603440	4.670321
H	-9.630083	8.368510	4.220829
C	-7.540913	1.343725	0.471028
C	-7.916917	0.294694	-0.381088
C	-6.245484	1.319777	1.010409
C	-7.001190	-0.715628	-0.653178
C	-5.396065	0.270468	0.680039
N	-5.755821	-0.733653	-0.141310
H	-8.918036	0.280915	-0.813040
H	-5.925257	2.118002	1.680513
H	-7.271871	-1.553909	-1.297957
H	-4.384334	0.219351	1.089077
C	-13.618769	2.449400	-1.389687
H	-13.978336	1.666400	-2.067036
C	-4.700791	-3.305138	-5.254550
C	-5.217652	-2.112866	-4.777546
C	-5.144960	-1.846534	-3.397270
N	-4.593604	-2.687480	-2.527477
C	-4.060972	-3.851803	-2.980173
C	-4.101172	-4.218051	-4.353170
H	-4.752609	-3.550888	-6.318353
H	-5.685197	-1.388421	-5.446680
H	-5.556785	-0.920296	-2.988467
C	-2.374345	-6.855764	-1.489788
C	-2.931686	-5.997359	-2.468691
C	-3.449039	-4.750550	-2.023650
N	-3.403135	-4.372942	-0.723195
C	-2.880890	-5.198042	0.175533
C	-2.351767	-6.458359	-0.163937
H	-1.973738	-7.827589	-1.789346
H	-2.888219	-4.853572	1.213856
H	-1.938930	-7.102936	0.614046
C	11.282588	0.926581	-3.857290
C	10.286781	-0.034537	-3.785573
C	9.629991	-0.244249	-2.558711
N	9.929390	0.443295	-1.459373
C	10.894645	1.396153	-1.514518
C	11.614633	1.680905	-2.705311

H	11.813784	1.110060	-4.794855
H	10.009123	-0.627553	-4.658716
H	8.838720	-0.994724	-2.468530
C	12.438203	3.838606	0.875509
C	12.189633	3.140677	-0.331961
C	11.183445	2.140303	-0.310305
N	10.472132	1.849781	0.812243
C	10.729924	2.517534	1.934746
C	11.707542	3.526045	2.009531
H	13.205840	4.616268	0.903030
H	10.143158	2.242733	2.814244
H	11.878893	4.044466	2.954455
C	12.627929	2.700209	-2.692895
C	12.904339	3.400060	-1.552518
C	-2.998157	-6.338041	-3.863442
C	-3.554069	-5.480683	-4.769268
H	-3.600360	-5.746487	-5.827808
H	-2.596055	-7.300855	-4.186912
H	13.176067	2.905601	-3.615124
H	13.677078	4.172132	-1.551048
C	8.416600	-0.553464	5.398003
C	9.229251	-1.389564	4.593805
C	9.302321	-1.082726	3.209181
N	8.632465	-0.038260	2.660335
C	7.868768	0.726785	3.435803
C	7.732857	0.503842	4.818880
H	8.336757	-0.748207	6.470640
H	7.346176	1.554244	2.946222
H	7.101297	1.164105	5.415809
C	11.630365	-3.754848	1.985859
C	11.674855	-3.408158	0.646082
C	10.929131	-2.300804	0.202203
N	10.171674	-1.572170	1.018961
C	10.118225	-1.896894	2.338329
C	10.838219	-2.992957	2.879699
H	12.203165	-4.606762	2.361228
H	12.279539	-3.972904	-0.065577
H	10.954492	-1.994969	-0.845972
C	10.743348	-3.276564	4.286418
C	9.971087	-2.507206	5.110108
H	11.306956	-4.122055	4.687345
H	9.909228	-2.729281	6.177809
C	-8.414432	-5.382247	0.797469
C	-7.558412	-4.822478	1.777178
C	-6.466724	-4.035971	1.318888
N	-6.248181	-3.802898	0.002067
C	-7.062034	-4.344565	-0.896051
C	-8.162918	-5.148695	-0.543377
H	-9.265067	-5.993947	1.108666
H	-6.840764	-4.133007	-1.946193
H	-8.801915	-5.569236	-1.321864
C	-4.870835	-3.076139	4.578428
C	-3.807133	-2.333099	4.095296

C	-3.657129	-2.191522	2.703876
N	-4.494799	-2.737255	1.827832
C	-5.551598	-3.460087	2.282416
C	-5.780946	-3.668744	3.670709
H	-5.017596	-3.209434	5.653292
H	-3.092496	-1.860805	4.771321
H	-2.829086	-1.610631	2.288504
C	-6.903418	-4.458239	4.097625
C	-7.754162	-5.016987	3.187681
H	-7.061965	-4.606058	5.168198
H	-8.602121	-5.620257	3.519891

126

CAGEphen+phen+12ZnDividedBy3 SCF Done: -6863.48200908 A.U.

N	0.000310	1.418530	-0.000121
N	-1.174272	3.473933	-0.008554
C	-1.128166	2.145316	-0.004912
C	1.128786	2.145319	0.004898
N	1.174873	3.473939	0.008910
C	0.000300	4.127963	0.000261
C	0.000290	5.601409	0.000337
C	1.204768	6.324037	-0.046989
C	-1.204197	6.324017	0.047733
C	1.145641	7.720337	-0.045878
C	-1.145085	7.720318	0.046742
N	0.000273	8.404218	0.000462
H	2.163508	5.807435	-0.087619
H	-2.162931	5.807401	0.088337
H	2.067366	8.311158	-0.084465
H	-2.066818	8.311123	0.085401
C	2.451654	1.442110	0.004424
C	3.635703	2.191321	0.099105
C	2.564587	0.047490	-0.096404
C	4.859546	1.532775	0.096894
C	3.833496	-0.531461	-0.107312
N	4.963542	0.194188	-0.007419
H	3.590520	3.277378	0.174490
H	1.671702	-0.573769	-0.171456
H	5.795775	2.088609	0.182953
H	3.959970	-1.612700	-0.202605
C	-2.451031	1.442094	-0.004595
C	-3.635087	2.191324	-0.099047
C	-2.563946	0.047452	0.095944
C	-4.858926	1.532774	-0.096920
C	-3.832848	-0.531508	0.106794
N	-4.962910	0.194173	0.007124
H	-3.589901	3.277397	-0.174192
H	-1.671044	-0.573806	0.170809
H	-5.795173	2.088608	-0.182803
H	-3.959315	-1.612764	0.201884
Zn	-6.901106	-0.647543	0.034237
Zn	6.901837	-0.647405	-0.034837
C	-8.676981	-4.956111	-1.464816
C	-8.078169	-4.781265	-0.192876

C	-7.545417	-3.500495	0.104773
N	-7.596369	-2.468362	-0.779096
C	-8.170888	-2.657984	-1.964782
C	-8.722620	-3.893199	-2.351017
H	-9.102018	-5.926326	-1.734620
H	-8.203535	-1.797757	-2.636940
H	-9.181649	-3.995856	-3.335806
C	-6.261214	-4.031281	3.587753
C	-5.756323	-2.763140	3.828065
C	-5.866155	-1.784668	2.822387
N	-6.439749	-2.028946	1.647017
C	-6.932631	-3.267176	1.392004
C	-6.869170	-4.321116	2.341734
H	-6.196937	-4.810706	4.351445
H	-5.284777	-2.512947	4.780008
H	-5.481589	-0.772302	2.979546
C	8.904076	2.266047	3.441991
C	7.753923	1.574874	3.788488
C	7.129779	0.764015	2.822361
N	7.602392	0.630847	1.585417
C	8.722130	1.309028	1.225945
C	9.423740	2.151165	2.129318
H	9.414053	2.900731	4.171362
H	7.330914	1.647795	4.791935
H	6.220140	0.205546	3.063452
C	10.802346	1.666370	-1.873324
C	10.371198	1.850599	-0.536488
C	9.200695	1.159584	-0.128999
N	8.503830	0.351161	-0.972459
C	8.934475	0.191585	-2.222076
C	10.083084	0.835758	-2.716168
H	11.700219	2.179031	-2.228003
H	8.351120	-0.476394	-2.859931
H	10.392885	0.672013	-3.749719
C	-7.417713	-5.607119	2.008097
C	-7.998017	-5.827226	0.790898
C	10.606106	2.836760	1.684439
C	11.061168	2.691098	0.404440
H	11.961628	3.214264	0.074745
H	11.137344	3.478110	2.391270
H	-7.365775	-6.407593	2.749496
H	-8.416064	-6.806414	0.546591
C	-8.903209	2.266437	-3.442321
C	-9.422383	2.152026	-2.129413
C	-8.720819	1.309711	-1.226175
N	-7.601564	0.630878	-1.585996
C	-7.129421	0.763616	-2.823154
C	-7.753535	1.574636	-3.789168
H	-9.413182	2.901251	-4.171582
H	-6.220191	0.204622	-3.064595
H	-7.330879	1.647177	-4.792789
C	-10.799476	1.668790	1.873951
C	-10.080213	0.838057	2.716679

C	-8.932150	0.193197	2.222208
N	-8.502041	0.352245	0.972342
C	-9.198888	1.160804	0.129007
C	-10.368868	1.852486	0.536870
H	-11.696943	2.181960	2.228925
H	-10.389607	0.674752	3.750421
H	-8.348802	-0.474905	2.859946
C	-11.058836	2.693107	-0.403950
C	-10.604235	2.838276	-1.684167
H	-11.958906	3.216779	-0.073996
H	-11.135460	3.479738	-2.390907
C	6.260989	-4.030889	-3.588576
C	6.867413	-4.321240	-2.341930
C	6.931301	-3.267210	-1.392322
N	6.440166	-2.028417	-1.648025
C	5.867926	-1.783681	-2.823955
C	5.757844	-2.762188	-3.829574
H	6.196494	-4.810373	-4.352189
H	5.484675	-0.770897	-2.981644
H	5.287476	-2.511579	-4.781990
C	8.670769	-4.957954	1.466416
C	8.716970	-3.894970	2.352500
C	8.167242	-2.659091	1.965540
N	7.594070	-2.468899	0.779294
C	7.542579	-3.501106	-0.104478
C	8.073391	-4.782519	0.193882
H	9.094281	-5.928685	1.736761
H	9.174949	-3.998059	3.337731
H	8.200450	-1.798783	2.637570
C	7.992860	-5.828543	-0.789792
C	7.414014	-5.607885	-2.007584
H	8.409428	-6.808218	-0.544911
H	7.361774	-6.408396	-2.748922

330

CAGEpy+py+NO3 SCF Done: -11190.7470068 A.U.

N	-2.372603	-3.724610	4.412098
N	-4.070522	-3.862038	2.778589
C	-2.965016	-4.323332	3.372350
C	-2.940068	-2.592275	4.842013
N	-4.044156	-2.061914	4.304290
C	-4.575200	-2.725958	3.271919
C	-5.758057	-2.134232	2.590016
C	-6.325266	-0.933653	3.040923
C	-6.291693	-2.729347	1.439898
C	-7.372765	-0.362550	2.325307
C	-7.337241	-2.106618	0.763390
N	-7.853963	-0.941045	1.203418
H	-5.938183	-0.431702	3.926879
H	-5.880469	-3.660838	1.052609
H	-7.798956	0.610659	2.605063
H	-7.731693	-2.519149	-0.171438
C	-2.270273	-1.844969	5.941385
C	-2.757794	-0.600882	6.361489

C	-1.098576	-2.334219	6.536691
C	-2.060487	0.123208	7.324967
C	-0.449365	-1.566860	7.498602
N	-0.924913	-0.358155	7.870596
H	-3.662352	-0.180353	5.923757
H	-0.678570	-3.293207	6.235641
H	-2.386821	1.123892	7.628296
H	0.505181	-1.884117	7.939951
C	-2.330999	-5.545811	2.807845
C	-2.887177	-6.192780	1.695144
C	-1.128857	-6.040131	3.330216
C	-2.222443	-7.277484	1.132521
C	-0.509503	-7.128988	2.721907
N	-1.051663	-7.722429	1.638787
H	-3.816641	-5.837969	1.250922
H	-0.659371	-5.567559	4.191914
H	-2.588461	-7.766982	0.219657
H	0.458965	-7.499657	3.074852
Pd	-0.107389	-9.281901	0.664019
N	3.985052	-4.308451	-2.184848
N	2.286143	-4.366521	-3.822167
C	2.867657	-4.824435	-2.707662
C	4.513566	-3.265502	-2.834302
N	3.994621	-2.743337	-3.950923
C	2.877465	-3.319809	-4.408252
C	2.222756	-2.724453	-5.605136
C	2.737064	-1.565383	-6.200178
C	1.039918	-3.269034	-6.125648
C	0.407131	-2.635705	-7.190649
N	0.908564	-1.504331	-7.732701
H	3.651018	-1.105597	-5.826239
H	0.599493	-4.165077	-5.689766
H	2.402800	-0.034700	-7.703808
H	-0.554272	-2.992549	-7.584532
C	5.710795	-2.608703	-2.242897
C	6.299982	-1.494532	-2.857778
C	6.236974	-3.047595	-1.021328
C	7.361314	-0.850014	-2.230274
C	7.296774	-2.357046	-0.439417
N	7.834882	-1.275087	-1.038771
H	5.919748	-1.113907	-3.805006
H	5.808531	-3.906817	-0.506618
H	7.806975	0.064767	-2.644791
H	7.686160	-2.642341	0.543819
C	2.204930	-5.939285	-1.977600
C	0.989668	-6.470186	-2.428286
C	2.745905	-6.439045	-0.784177
C	0.342379	-7.446448	-1.675228
C	2.054353	-7.418047	-0.078144
N	0.871375	-7.897950	-0.519420
H	0.530777	-6.109245	-3.347919
H	3.685062	-6.050653	-0.391778
H	-0.636534	-7.835483	-1.975245

H	2.407770	-7.786525	0.894775
Pd	9.345092	-0.177169	-0.151504
Pd	-9.340502	0.052317	0.165922
Pd	0.168183	0.733086	9.244872
N	-4.110555	3.937824	-2.573691
N	-2.489013	3.818261	-4.284311
C	-3.032497	4.405269	-3.211985
C	-4.643362	2.811725	-3.060185
N	-4.164310	2.162545	-4.126802
C	-3.079847	2.693457	-4.702632
C	-2.459221	1.956693	-5.837203
C	-1.294046	2.433409	-6.454743
C	-2.985003	0.732363	-6.269071
C	-0.687335	1.672207	-7.448656
C	-2.330695	0.014474	-7.266772
N	-1.199192	0.482582	-7.832535
H	-0.844429	3.376089	-6.144702
H	-3.886615	0.322301	-5.815897
H	0.263150	1.975017	-7.909173
H	-2.688810	-0.971919	-7.580322
C	-5.810597	2.224972	-2.347123
C	-6.394937	1.027583	-2.784549
C	-6.311003	2.820502	-1.182245
C	-7.424677	0.459104	-2.041554
C	-7.341823	2.201677	-0.479859
N	-7.874532	1.038553	-0.907185
H	-6.032931	0.524974	-3.680762
H	-5.886315	3.749805	-0.804626
H	-7.861864	-0.512194	-2.310554
H	-7.710814	2.615593	0.464719
C	-2.361574	5.612356	-2.657296
C	-1.203632	6.131314	-3.253913
C	-2.833320	6.219144	-1.486185
C	-0.550889	7.205422	-2.657738
C	-2.133116	7.288459	-0.933673
N	-1.009685	7.758562	-1.513971
H	-0.796002	5.686070	-4.160804
H	-3.727577	5.847647	-0.987321
H	0.395128	7.598097	-3.055044
H	-2.446698	7.740923	0.013322
Pd	0.097891	9.286272	-0.669844
N	2.576755	4.336374	3.688855
N	4.200827	4.177380	1.984313
C	3.131611	4.752647	2.545121
C	3.142786	3.269239	4.263644
N	4.216367	2.639532	3.773510
C	4.710410	3.121503	2.628245
C	5.864187	2.412571	2.010763
C	6.383141	2.826215	0.777073
C	6.417034	1.274835	2.615777
C	2.054710	-0.973546	-7.259797
C	7.434487	0.582420	1.966907
N	7.902383	0.985659	0.765456

H	5.983827	3.703546	0.270085
H	6.039747	0.912382	3.571547
H	7.847673	-0.350903	2.373402
C	2.510564	2.718346	5.493138
C	1.358084	3.304818	6.035307
C	3.011346	1.558334	6.097710
C	0.738471	2.708729	7.128920
C	2.344963	1.006700	7.188859
N	1.225599	1.576241	7.680894
H	0.927852	4.202287	5.592156
H	3.902210	1.067275	5.708174
H	-0.203821	3.095365	7.540718
H	2.682941	0.068791	7.642055
C	2.484922	5.882866	1.824085
C	2.966598	6.309029	0.579434
C	1.338229	6.503304	2.339749
C	2.286400	7.303223	-0.118997
C	0.704687	7.494275	1.596759
N	1.172888	7.871634	0.387026
H	3.852981	5.854427	0.139164
H	0.923048	6.197877	3.299700
H	2.606809	7.612683	-1.119534
H	-0.233648	7.956225	1.933078
Pd	-0.161607	-0.602076	-9.253929
C	7.400715	2.090115	0.175732
H	7.782804	2.358238	-0.815206
C	2.863794	12.825385	1.109817
C	2.121989	12.012968	1.971928
C	1.325088	11.007737	1.433273
N	1.257111	10.802434	0.102826
C	1.962021	11.581111	-0.742162
C	2.777396	12.605573	-0.264249
H	3.498736	13.621578	1.506735
H	2.146191	12.153169	3.053780
H	0.685129	10.381999	2.056447
C	-2.604453	12.597117	-2.924112
C	-2.523604	12.565738	-1.532575
C	-1.727732	11.601655	-0.916092
N	-1.036455	10.701947	-1.644249
C	-1.098792	10.725059	-2.990506
C	-1.876445	11.661395	-3.664376
H	-3.224090	13.342993	-3.428440
H	-0.468759	10.008984	-3.519583
H	-1.896551	11.652890	-4.755325
C	-12.791549	2.576818	-2.083251
C	-11.928098	1.758474	-2.816981
C	-10.949848	1.033932	-2.143441
N	-10.818480	1.107938	-0.803915
C	-11.647199	1.887990	-0.081365
C	-12.648192	2.636991	-0.697718
H	-13.568075	3.157533	-2.587641
H	-12.007817	1.667831	-3.901448
H	-10.286940	0.339199	-2.660728

C	-12.749552	-2.455187	2.498037
C	-12.639960	-2.516181	1.109481
C	-11.650103	-1.772677	0.468903
N	-10.800063	-0.996780	1.170991
C	-10.898873	-0.921979	2.513295
C	-11.864322	-1.641355	3.210449
H	-13.516877	-3.031559	3.021157
H	-10.220710	-0.229962	3.014132
H	-11.917505	-1.549947	4.296483
C	1.887894	-12.922550	-1.807974
C	0.502997	-12.773957	-1.745770
C	-0.038795	-11.716418	-1.017025
N	0.755370	-10.838340	-0.372058
C	2.095621	-10.973918	-0.416931
C	2.696698	-12.007025	-1.128908
H	2.334285	-13.743185	-2.375365
H	2.677224	-10.265788	0.174461
H	3.784844	-12.087799	-1.136373
C	-2.215791	-12.491810	3.596294
C	-2.996053	-11.657128	2.791306
C	-2.363679	-10.748557	1.948488
N	-1.019807	-10.656860	1.896033
C	-0.253256	-11.458286	2.662357
C	-0.827064	-12.390256	3.525543
H	-2.687102	-13.214013	4.267812
H	-4.086050	-11.706705	2.801383
H	-2.923356	-10.113738	1.260528
C	-2.857872	1.296856	-12.785240
C	-2.823922	-0.080557	-12.572206
C	-2.028907	-0.594302	-11.549005
N	-1.293427	0.219551	-10.765197
C	-1.310616	1.552486	-10.964133
C	-2.085065	2.126091	-11.967533
H	-3.475858	1.721551	-13.580451
H	-0.648622	2.147968	-10.334314
H	-2.067750	3.208752	-12.102382
C	2.439222	-2.960531	-12.573845
C	1.676691	-3.671919	-11.643253
C	0.928471	-2.968390	-10.704677
N	0.927299	-1.620626	-10.674569
C	1.652907	-0.920006	-11.568885
C	2.421725	-1.566940	-12.534960
H	3.036689	-3.488538	-13.321433
H	1.647195	-4.762691	-11.640502
H	0.273371	-3.472767	-9.993317
C	-2.472682	-1.066834	12.869211
C	-1.729023	-1.921082	12.050196
C	-0.968057	-1.376211	11.020803
N	-0.936859	-0.046945	10.797991
C	-1.644062	0.790555	11.582670
C	-2.424207	0.306036	12.631261
H	-3.079601	-1.468830	13.684494
H	-1.723992	-3.001402	12.203606

H	-0.326825	-1.992113	10.389171
C	2.922290	3.059351	12.462109
C	2.852389	1.666946	12.453642
C	2.041564	1.029130	11.516268
N	1.325068	1.737807	10.620591
C	1.377209	3.084506	10.621627
C	2.168724	3.779168	11.530728
H	3.553169	3.580156	13.186937
H	0.729315	3.597772	9.910199
H	2.179588	4.869975	11.505019
C	12.697570	-3.072435	-2.082735
C	11.821141	-2.363374	-2.908858
C	10.871777	-1.529075	-2.327300
N	10.779883	-1.394216	-0.989363
C	11.621432	-2.067441	-0.179127
C	12.595416	-2.917031	-0.701040
H	13.452531	-3.735030	-2.513599
H	11.869128	-2.441290	-3.996173
H	10.201297	-0.912480	-2.926786
C	12.856864	2.564043	1.718241
C	12.720680	2.416659	0.338548
C	11.700800	1.610984	-0.164990
N	10.847194	0.973159	0.660783
C	10.970749	1.100971	1.996885
C	11.966887	1.891845	2.560651
H	13.647893	3.192722	2.134972
H	10.285475	0.513299	2.608726
H	12.039455	1.965564	3.646898
O	2.562905	-10.125941	2.388952
O	-2.811906	-10.286525	-0.946961
O	-1.494990	3.469982	9.974189
O	3.575079	-0.665971	9.726243
O	-3.271110	9.842793	0.804833
O	1.743190	10.000740	-3.435819
O	9.715337	-3.485816	1.482923
O	10.276359	1.311134	-2.934440
O	-10.304139	-1.855350	-2.347951
O	-9.730100	3.560216	1.325551
O	3.261584	0.735613	-9.796322
O	-3.598664	-1.981922	-9.537164
N	2.359299	1.531643	-9.494270
O	2.200871	1.872937	-8.291346
O	1.567205	1.968258	-10.366268
N	-2.696147	-2.745524	-9.160703
O	-2.506725	-2.934267	-7.929093
O	-1.930984	-3.296003	-9.991256
N	2.533922	9.178216	-2.909728
O	2.342944	7.943878	-3.079194
O	3.468428	9.565977	-2.191981
N	-2.341617	9.540767	1.568700
O	-2.172515	8.338527	1.907227
O	-1.531425	10.411774	1.972830
N	-9.444662	-2.603661	-1.818963

O	-9.773667	-3.457878	-0.981615
O	-8.228581	-2.448094	-2.110919
N	-9.381805	2.706414	2.155320
O	-8.158993	2.546168	2.415502
O	-10.229962	1.961036	2.706224
N	-2.300511	2.930349	9.175220
O	-3.220846	2.209154	9.589926
O	-2.139895	3.091336	7.935477
N	2.655942	-1.457443	9.465241
O	2.459011	-1.819115	8.274260
O	1.882424	-1.865733	10.367142
N	2.104666	-9.215248	3.123011
O	1.349187	-9.482511	4.070155
O	2.382211	-8.013901	2.861298
N	-2.328775	-9.496650	-1.796042
O	-1.576073	-9.908532	-2.692254
O	-2.573639	-8.264279	-1.698672
N	9.453516	2.153308	-2.496478
O	9.830959	3.106342	-1.797732
O	8.225437	1.996572	-2.733194
N	9.398664	-2.516964	2.190515
O	8.185035	-2.302211	2.453730
O	10.268468	-1.714487	2.611825
H	0.830185	-11.327776	2.590461
H	-0.174557	-13.014343	4.138086
H	-1.118112	-11.548895	-0.958414
H	-0.170833	-13.458497	-2.263277
H	-13.292676	3.265587	-0.081322
H	-11.483599	1.921632	0.999362
H	-11.513444	-1.806953	-0.615655
H	-13.302737	-3.141101	0.508933
H	-1.593562	1.856891	11.345260
H	-2.991232	1.013559	13.238171
H	1.972743	-0.060972	11.459601
H	3.426045	1.059884	13.155675
H	3.341684	13.207836	-0.977890
H	1.880898	11.358252	-1.809648
H	-1.652645	11.525600	0.172125
H	-3.077437	13.270100	-0.910015
H	11.541815	1.484395	-1.239458
H	13.385361	2.925975	-0.360899
H	11.490840	-1.931863	0.898286
H	13.251708	-3.453118	-0.013768
H	-1.988229	-1.665574	-11.332437
H	-3.413724	-0.769251	-13.179077
H	1.626442	0.170203	-11.486712
H	3.004340	-0.966424	-13.235238

9

CoNO32 SCF Done: -1942.84557401 A.U.

Co	0.000000	0.000000	0.000000
O	0.000000	1.066889	1.603568
O	-0.000000	-1.066889	1.603568
N	0.000000	0.000000	2.340637

O -0.000000 -1.066889 -1.603568
O 0.000000 1.066889 -1.603568
N 0.000000 0.000000 -2.340637
O -0.000000 -0.000000 -3.524042
O -0.000000 -0.000000 3.524042
9
CuNO32 SCF Done: -2200.54046856 A.U.
Cu 0.000000 0.000000 0.000000
O 0.000000 1.071425 1.684250
O -0.000000 -1.071425 1.684250
N 0.000000 0.000000 2.402050
O -0.000000 -1.071425 -1.684250
O 0.000000 1.071425 -1.684250
N 0.000000 0.000000 -2.402050
O 0.000000 -0.000000 -3.590573
O -0.000000 -0.000000 3.590573
9
FeNO32Q SCF Done: -1823.82625930 A.U.
Fe 0.000000 0.000000 0.000000
O 0.000000 1.080044 1.729225
O -0.000000 -1.080044 1.729225
N 0.000000 0.000000 2.444809
O -1.080044 0.000000 -1.729225
O 1.080044 -0.000000 -1.729225
N 0.000000 0.000000 -2.444809
O 0.000000 0.000000 -3.633135
O -0.000000 -0.000000 3.633135
9
FeNO32Sep SCF Done: -1823.71691832 A.U.
Fe 0.000000 0.000000 0.062388
O 0.000000 1.121522 1.552913
O -0.000000 -1.121522 1.552913
N 0.000000 0.000000 2.413510
O -1.075350 0.000000 -1.681576
O 1.075350 -0.000000 -1.681576
N 0.000000 -0.000000 -2.404209
O 0.000000 -0.000000 -3.588654
O 0.000000 0.000000 3.635079
9
FeNO32T SCF Done: -1823.78179992 A.U.
Fe 0.000000 0.000000 0.046897
O -0.000000 1.061502 1.653422
O -0.000000 -1.061502 1.653422
N 0.000000 0.000000 2.401590
O -1.076385 0.000000 -1.706461
O 1.076385 -0.000000 -1.706461
N -0.000000 -0.000000 -2.421696
O -0.000000 -0.000000 -3.611686
O 0.000000 0.000000 3.582943
9
FeNO32 SCF Done: -1823.73211255 A.U.
Fe 0.000000 0.000000 0.000000
O 0.000000 1.064906 1.633226

O -0.000000 -1.064906 1.633226
N 0.000000 0.000000 2.373646
O -1.064906 0.000000 -1.633226
O 1.064906 -0.000000 -1.633226
N 0.000000 0.000000 -2.373646
O 0.000000 0.000000 -3.556003
O -0.000000 -0.000000 3.556003
36
linkerPuntual+12Pd SCF Done: -1020.94694644 A.U.
N -1.310073 0.377693 -0.115920
N 0.982140 0.945696 -0.115993
C -0.316302 1.276450 -0.117303
C -0.947290 -0.912167 -0.117291
N 0.327942 -1.323421 -0.115919
C 1.263605 -0.364314 -0.117409
C 2.705756 -0.780094 -0.068026
C 3.074942 -2.134718 -0.029380
C 3.739484 0.170057 -0.030322
C 5.064034 -0.253900 0.087169
N 5.403319 -1.557790 0.167909
H 2.320312 -2.918881 -0.089809
H 3.518171 1.235565 -0.091493
H 4.725971 -3.530121 0.115621
H 5.880039 0.472322 0.113934
C -2.028453 -1.953202 -0.067828
C -1.722437 -3.323475 -0.029093
C -3.386214 -1.595627 -0.030244
C -2.751896 -4.258550 0.088398
C -4.359578 -2.588973 0.087212
N -4.050805 -3.900430 0.168110
H -0.688975 -3.664560 -0.089469
H -3.687997 -0.550064 -0.091516
H -2.530951 -5.328319 0.115986
H -5.420259 -2.327750 0.113797
C -0.677298 2.733277 -0.067761
C 0.311261 3.730289 -0.028983
C -2.017014 3.153448 -0.030116
C -0.062358 5.069893 0.088586
C -2.312127 4.512526 0.087355
N -1.352544 5.458279 0.168241
H 1.367679 3.468826 -0.089364
H -2.829116 2.429041 -0.091386
H 0.694244 5.857785 0.116206
H -3.349051 4.856129 0.114006
C 4.421898 -2.480945 0.088116
36
linker SCF Done: -1020.95076868 A.U.
N -0.301119 -1.329273 0.000019
N -1.000634 0.925353 0.000053
C -1.253127 -0.388809 0.000036
C 0.963228 -0.890857 0.000034
N 1.301682 0.403838 0.000030
C 0.289833 1.279588 0.000035

C	0.618282	2.729533	0.000044
C	1.950089	3.169579	-0.000489
C	-0.393887	3.700541	0.000506
C	-0.028102	5.049000	0.000442
N	1.239431	5.471612	-0.000126
H	2.766242	2.446857	-0.000881
H	-1.441812	3.400061	0.000919
H	3.234245	4.910611	-0.001035
H	-0.802406	5.825010	0.000730
C	2.054724	-1.900237	0.000048
C	3.401706	-1.509108	0.000639
C	1.769981	-3.273657	-0.000596
C	4.386670	-2.500061	0.000593
C	2.834832	-4.178216	-0.000607
N	4.118966	-3.809093	-0.000081
H	3.665400	-0.451326	0.001135
H	0.736026	-3.619159	-0.001084
H	5.445851	-2.217437	0.000989
H	2.635796	-5.256235	-0.001211
C	-2.673041	-0.829326	0.000062
C	-3.720032	0.104032	-0.000428
C	-3.007872	-2.191396	0.000500
C	-5.035857	-0.365805	-0.000447
C	-4.358563	-2.548850	0.000454
N	-5.358321	-1.662439	-0.000074
H	-3.502211	1.172205	-0.000796
H	-2.223688	-2.948686	0.000882
H	-5.869892	0.345620	-0.000926
H	-4.643453	-3.607424	0.000721
C	2.201113	4.544034	-0.000525

9

NiNO32Q SCF Done: -2068.22508312 A.U.

Ni	0.000000	0.000000	0.000000
O	-0.000000	1.089883	1.568047
O	-0.000000	-1.089883	1.568047
N	-0.000000	0.000000	2.349171
O	-0.000000	-1.089883	-1.568047
O	0.000000	1.089883	-1.568047
N	0.000000	0.000000	-2.349171
O	0.000000	-0.000000	-3.557550
O	-0.000000	-0.000000	3.557550

9

NiNO32T SCF Done: -2068.38832666 A.U.

Ni	0.000000	0.000000	0.000000
O	0.000000	1.074090	1.676380
O	-0.000000	-1.074090	1.676380
N	0.000000	0.000000	2.397152
O	-0.000000	-1.074090	-1.676380
O	0.000000	1.074090	-1.676380
N	0.000000	0.000000	-2.397152
O	0.000000	-0.000000	-3.585346
O	-0.000000	-0.000000	3.585346

9

NiNO32 SCF Done: -2068.39328174 A.U.

Ni	0.000000	0.000000	0.000000
O	0.000000	1.061954	1.570487
O	-0.000000	-1.061954	1.570487
N	0.000000	-0.000000	2.310825
O	-0.000000	-1.061954	-1.570487
O	0.000000	1.061954	-1.570487
N	0.000000	-0.000000	-2.310825
O	0.000000	0.000000	-3.495198
O	-0.000000	0.000000	3.495198

1

Ni SCF Done: -1508.01846137 A.U.

Ni	0.000000	0.000000	0.000000

4

NO3- SCF Done: -280.125157595 A.U.

O	1.179456	0.429377	0.000002
N	0.000003	0.000042	-0.000006
O	-0.217854	-1.236110	0.000002
O	-0.961605	0.806696	0.000002

9

PdNO32 SCF Done: -688.115954579 A.U.

Pd	0.000000	0.000000	0.000000
O	-0.000000	1.070057	1.754275
O	-0.000000	-1.070057	1.754275
N	0.000000	0.000000	2.482267
O	-0.000000	-1.070057	-1.754275
O	0.000000	1.070057	-1.754275
N	0.000000	0.000000	-2.482267
O	-0.000000	-0.000000	-3.666569
O	-0.000000	-0.000000	3.666569

1

Pd SCF Done: -127.883448225 A.U.

Pd	0.000000	0.000000	0.000000

67

Phen2Py2Co+2 SCF Done: -3021.05473955 A.U.

C	-4.522750	-0.184141	-2.219665
C	-4.077497	-0.923204	-1.097129
C	-3.664856	0.718288	-2.822802
C	-2.363054	0.869439	-2.304099
N	-1.915359	0.192480	-1.253351
H	-3.975283	1.304296	-3.689700
H	-1.667111	1.572084	-2.771524
C	-2.539493	-3.095878	2.225173
C	-1.246246	-2.829826	2.639316
C	-3.081505	-2.379834	1.131873
C	-0.509215	-1.834816	1.974315
C	-2.256394	-1.406468	0.505561
N	-0.999352	-1.136429	0.952346
H	-0.793113	-3.370023	3.472146
H	0.507827	-1.588698	2.283648
C	-2.752322	-0.682716	-0.641494
Co	-0.000002	0.304142	-0.000005
C	4.522754	-0.184146	2.219654

C	4.077493	-0.923217	1.097126
C	2.752317	-0.682728	0.641495
N	1.915360	0.192476	1.253348
C	2.363064	0.869445	2.304086
C	3.664868	0.718294	2.822785
H	5.536148	-0.334957	2.600021
H	1.667127	1.572099	2.771506
H	3.975302	1.304309	3.689676
C	2.539475	-3.095908	-2.225157
C	1.246230	-2.829854	-2.639301
C	0.509203	-1.834835	-1.974308
N	0.999344	-1.136442	-0.952344
C	2.256384	-1.406485	-0.505555
C	3.081490	-2.379859	-1.131862
H	3.144377	-3.854031	-2.728741
H	0.793093	-3.370054	-3.472127
H	-0.507837	-1.588714	-2.283643
C	4.414235	-2.599308	-0.640635
C	4.895957	-1.893783	0.424279
H	5.911708	-2.064150	0.788215
H	5.037422	-3.344470	-1.140020
H	-3.144399	-3.853994	2.728762
H	-5.536143	-0.334951	-2.600035
N	-0.936068	1.700337	1.107943
C	-1.026273	1.566906	2.445076
C	-1.520123	2.770504	0.535468
C	-1.690157	2.487525	3.252502
C	-2.205505	3.736501	1.267842
C	-2.294093	3.596905	2.655173
H	-0.553184	0.685129	2.877114
H	-1.430326	2.847595	-0.547543
H	-1.732347	2.329832	4.331800
H	-2.661311	4.583630	0.752040
H	-2.824736	4.336909	3.258829
N	0.936077	1.700334	-1.107948
C	1.520153	2.770486	-0.535466
C	1.026264	1.566921	-2.445084
C	2.205543	3.736481	-1.267835
C	1.690154	2.487540	-3.252505
C	2.294114	3.596902	-2.655168
H	1.430370	2.847565	0.547547
H	0.553152	0.685159	-2.877129
H	2.661369	4.583596	-0.752026
H	1.732327	2.329862	-4.331806
H	2.824763	4.336906	-3.258820
C	-4.414250	-2.599280	0.640645
C	-4.895966	-1.893762	-0.424276
H	-5.911717	-2.064127	-0.788213
H	-5.037441	-3.344436	1.140034

67

Phen2Py2Cu+2 SCF Done: -3278.70725283 A.U.

C	4.622628	-0.231572	2.244925
C	4.170564	-0.957910	1.116354

C	3.770544	0.663994	2.865296
C	2.465552	0.818928	2.354903
N	2.012415	0.154058	1.300308
H	4.087468	1.239747	3.736732
H	1.770306	1.515947	2.833596
C	2.648528	-3.092802	-2.235490
C	1.355914	-2.833789	-2.656482
C	3.181262	-2.389481	-1.128814
C	0.608555	-1.855860	-1.977442
C	2.346534	-1.430401	-0.489365
N	1.092221	-1.175554	-0.943164
H	0.913992	-3.365730	-3.500593
H	-0.412006	-1.610575	-2.281223
C	2.840952	-0.714823	0.670645
Cu	0.000000	0.319618	0.000003
C	-4.622626	-0.231593	-2.244930
C	-4.170564	-0.957919	-1.116351
C	-2.840952	-0.714828	-0.670642
N	-2.012414	0.154047	-1.300313
C	-2.465551	0.818906	-2.354915
C	-3.770542	0.663967	-2.865307
H	-5.638803	-0.387516	-2.615807
H	-1.770305	1.515922	-2.833613
H	-4.087465	1.239712	-3.736750
C	-2.648531	-3.092779	2.235516
C	-1.355917	-2.833764	2.656506
C	-0.608557	-1.855841	1.977457
N	-1.092223	-1.175544	0.943173
C	-2.346535	-1.430395	0.489375
C	-3.181264	-2.389469	1.128833
H	-3.263437	-3.838227	2.746105
H	-0.913996	-3.365698	3.500621
H	0.412003	-1.610553	2.281237
C	-4.514762	-2.610537	0.641643
C	-4.992879	-1.916783	-0.431800
H	-6.009487	-2.086845	-0.793582
H	-5.140986	-3.346219	1.151228
H	3.263434	-3.838254	-2.746074
H	5.638805	-0.387492	2.615803
N	0.960292	1.761701	-1.157486
C	1.031410	1.629074	-2.493074
C	1.565756	2.810813	-0.575510
C	1.702443	2.545185	-3.300719
C	2.261678	3.770851	-1.307150
C	2.330764	3.638268	-2.696913
H	0.539037	0.753494	-2.919854
H	1.484940	2.872282	0.510780
H	1.732772	2.398432	-4.381983
H	2.739881	4.606073	-0.792132
H	2.868132	4.373938	-3.300146
N	-0.960292	1.761713	1.157475
C	-1.565757	2.810818	0.575490
C	-1.031408	1.629099	2.493066

C	-2.261680	3.770863	1.307121
C	-1.702439	2.545219	3.300702
C	-2.330763	3.638294	2.696885
H	-1.484944	2.872275	-0.510801
H	-0.539030	0.753526	2.919854
H	-2.739884	4.606078	0.792094
H	-1.732765	2.398478	4.381968
H	-2.868130	4.373971	3.300111
C	4.514760	-2.610546	-0.641623
C	4.992879	-1.916781	0.431812
H	6.009487	-2.086840	0.793596
H	5.140984	-3.346233	-1.151202

67

Phen2Py2Fe+2Q SCF Done: -2902.01423680 A.U.

C	4.276171	-0.255677	2.528538
C	3.939590	-1.025199	1.388647
C	3.392990	0.705617	2.988301
C	2.171833	0.884716	2.311388
N	1.825688	0.175787	1.240737
H	3.624131	1.317873	3.861672
H	1.452919	1.633350	2.654542
C	2.758983	-3.231738	-2.057861
C	1.537526	-2.935266	-2.638094
C	3.182252	-2.516071	-0.911619
C	0.747811	-1.916343	-2.072472
C	2.311257	-1.511166	-0.411133
N	1.123673	-1.225474	-1.002115
H	1.182544	-3.472865	-3.519016
H	-0.222760	-1.654056	-2.503218
C	2.689790	-0.760775	0.766061
Fe	0.000010	0.412834	-0.000006
C	-4.276149	-0.255694	-2.528551
C	-3.939562	-1.025222	-1.388666
C	-2.689760	-0.760797	-0.766083
N	-1.825665	0.175773	-1.240754
C	-2.171815	0.884706	-2.311401
C	-3.392973	0.705605	-2.988313
H	-5.230028	-0.426694	-3.033886
H	-1.452905	1.633344	-2.654552
H	-3.624117	1.317863	-3.861680
C	-2.758923	-3.231793	2.057811
C	-1.537461	-2.935326	2.638035
C	-0.747752	-1.916395	2.072418
N	-1.123626	-1.225515	1.002073
C	-2.311216	-1.511200	0.411099
C	-3.182204	-2.516114	0.911582
H	-3.399883	-4.013569	2.473213
H	-1.182469	-3.472935	3.518947
H	0.222822	-1.654112	2.503157
C	-4.434916	-2.762111	0.251366
C	-4.800202	-2.043173	-0.850532
H	-5.756003	-2.233027	-1.344069
H	-5.093444	-3.536568	0.651069

H	3.399948	-4.013508	-2.473266
H	5.230048	-0.426677	3.033875
N	1.040266	1.938242	-1.211236
C	1.308612	1.708861	-2.509862
C	1.572916	3.029729	-0.632150
C	2.105611	2.556546	-3.276601
C	2.381009	3.931405	-1.321892
C	2.652575	3.692206	-2.672066
H	0.872248	0.805307	-2.941309
H	1.338156	3.178533	0.424485
H	2.292881	2.327378	-4.327347
H	2.788509	4.803441	-0.807000
H	3.281692	4.378771	-3.243648
N	-1.040318	1.938160	1.211279
C	-1.573066	3.029610	0.632214
C	-1.308604	1.708757	2.509913
C	-2.381202	3.931226	1.321984
C	-2.105641	2.556381	3.276681
C	-2.652706	3.692004	2.672167
H	-1.338352	3.178433	-0.424429
H	-0.872162	0.805232	2.941343
H	-2.788782	4.803233	0.807107
H	-2.292859	2.327198	4.327432
H	-3.281855	4.378522	3.243771
C	4.434960	-2.762073	-0.251397
C	4.800237	-2.043142	0.850509
H	5.756035	-2.232998	1.344050
H	5.093492	-3.536523	-0.651103

67

Phen2Py2Fe+2T SCF Done: -2901.99729771 A.U.

C	3.995122	-0.111108	2.631394
C	3.731660	-0.931015	1.508279
C	3.079362	0.863905	2.986904
C	1.904085	1.014448	2.228800
N	1.622322	0.255263	1.169563
H	3.250620	1.516967	3.844280
H	1.168984	1.775771	2.491408
C	2.732842	-3.290368	-1.899379
C	1.538569	-3.014474	-2.545091
C	3.106953	-2.528258	-0.766107
C	0.725300	-1.970643	-2.061709
C	2.215989	-1.503134	-0.355026
N	1.057512	-1.235862	-1.006993
H	1.222197	-3.588360	-3.417862
H	-0.222660	-1.725347	-2.549052
C	2.524034	-0.702607	0.799344
Fe	0.000004	0.373091	-0.000005
C	-3.995116	-0.111100	-2.631403
C	-3.731659	-0.931004	-1.508284
C	-2.524031	-0.702601	-0.799351
N	-1.622314	0.255262	-1.169574
C	-1.904071	1.014443	-2.228816
C	-3.079349	0.863905	-2.986919

H	-4.914856	-0.252668	-3.204231
H	-1.168964	1.775759	-2.491428
H	-3.250602	1.516962	-3.844299
C	-2.732847	-3.290361	1.899372
C	-1.538569	-3.014477	2.545079
C	-0.725294	-1.970653	2.061693
N	-1.057505	-1.235868	1.006979
C	-2.215987	-1.503130	0.355018
C	-3.106957	-2.528247	0.766103
H	-3.387269	-4.090842	2.253579
H	-1.222197	-3.588367	3.417847
H	0.222671	-1.725365	2.549032
C	-4.321159	-2.739086	0.026348
C	-4.621208	-1.969907	-1.062543
H	-5.547491	-2.134633	-1.617484
H	-5.003363	-3.527602	0.351987
H	3.387259	-4.090854	-2.253583
H	4.914860	-0.252680	3.204224
N	1.103566	1.862205	-1.191174
C	1.479328	1.606117	-2.457751
C	1.569063	2.980976	-0.607385
C	2.318514	2.448897	-3.184420
C	2.409340	3.882530	-1.258238
C	2.793834	3.613062	-2.574463
H	1.093159	0.685461	-2.899881
H	1.251709	3.156923	0.422252
H	2.592316	2.192865	-4.209683
H	2.754834	4.777902	-0.738007
H	3.452676	4.297066	-3.114629
N	-1.103572	1.862180	1.191184
C	-1.569104	2.980938	0.607399
C	-1.479301	1.606090	2.457770
C	-2.409386	3.882478	1.258265
C	-2.318488	2.448857	3.184452
C	-2.793846	3.613009	2.574500
H	-1.251775	3.156887	-0.422245
H	-1.093103	0.685445	2.899897
H	-2.754910	4.777840	0.738037
H	-2.592264	2.192825	4.209723
H	-3.452691	4.297002	3.114676
C	4.321150	-2.739105	-0.026348
C	4.621203	-1.969926	1.062542
H	5.547483	-2.134658	1.617486
H	5.003350	-3.527627	-0.351984

67

Phen2Py2Fe+2 Binary file

C	-4.140758	-0.136838	-2.439040
C	-3.803611	-0.925959	-1.313816
C	-3.243704	0.819427	-2.883652
C	-2.015263	0.981646	-2.214968
N	-1.663304	0.251635	-1.157160
H	-3.469737	1.449410	-3.745659
H	-1.300820	1.730834	-2.557906

C	-2.530557	-3.191788	2.068177
C	-1.288475	-2.889537	2.601295
C	-2.998694	-2.459210	0.951603
C	-0.524993	-1.851842	2.033460
C	-2.152155	-1.441676	0.444553
N	-0.945241	-1.136923	0.991624
H	-0.892307	-3.438600	3.457215
H	0.452370	-1.592490	2.444127
C	-2.548309	-0.680601	-0.702552
Fe	-0.000061	0.341546	0.000012
C	4.140857	-0.136254	2.438929
C	3.803671	-0.925641	1.313904
C	2.548288	-0.680487	0.702707
N	1.663225	0.251720	1.157224
C	2.015174	0.981887	2.214939
C	3.243709	0.819945	2.883506
H	5.098665	-0.285539	2.943298
H	1.300505	1.730817	2.557934
H	3.469744	1.450077	3.745405
C	2.530168	-3.192535	-2.067240
C	1.287984	-2.890468	-2.600233
C	0.524612	-1.852557	-2.032655
N	0.945050	-1.137257	-0.991139
C	2.152036	-1.441865	-0.444169
C	2.998511	-2.459558	-0.951015
H	3.145670	-3.987914	-2.494511
H	0.891661	-3.439841	-3.455883
H	-0.452780	-1.593302	-2.443305
C	4.266242	-2.689669	-0.312258
C	4.654161	-1.950604	0.770558
H	5.620698	-2.132127	1.245911
H	4.918189	-3.470051	-0.711119
H	-3.146145	-3.987017	2.495603
H	-5.098486	-0.286358	-2.943492
N	-0.983597	1.746864	1.121554
C	-1.251280	1.541917	2.427677
C	-1.494181	2.856570	0.550206
C	-2.013037	2.418432	3.196768
C	-2.267190	3.784689	1.243643
C	-2.535341	3.566697	2.596831
H	-0.839950	0.636169	2.871886
H	-1.270184	3.005208	-0.505234
H	-2.192532	2.195703	4.250223
H	-2.650559	4.664132	0.722909
H	-3.138249	4.274446	3.170428
N	0.983577	1.746295	-1.122040
C	1.494628	2.855979	-0.551082
C	1.251099	1.540771	-2.428109
C	2.268001	3.783538	-1.244862
C	2.013268	2.416668	-3.197493
C	2.536064	3.564925	-2.597964
H	1.270711	3.005057	0.504318
H	0.839406	0.635004	-2.871960

H	2.651732	4.663017	-0.724457
H	2.192690	2.193498	-4.250868
H	3.139274	4.272202	-3.171825
C	-4.266329	-2.689566	0.312742
C	-4.654114	-1.950849	-0.770349
H	-5.620557	-2.132559	-1.245820
H	-4.918298	-3.469846	0.711768

67

Phen2Py2Ni+2 SCF Done: -3146.55840220 A.U.

C	-3.628484	-0.495227	-2.971684
C	-3.815304	-0.845701	-1.613309
C	-2.445928	0.102479	-3.373195
C	-1.448713	0.345816	-2.415103
N	-1.606119	0.022510	-1.130992
H	-2.273959	0.384096	-4.413266
H	-0.495570	0.810451	-2.681116
C	-4.204272	-1.883061	2.498796
C	-3.157995	-1.580959	3.351287
C	-4.106951	-1.544298	1.126275
C	-2.021360	-0.928722	2.825578
C	-2.912194	-0.901285	0.698557
N	-1.904263	-0.597187	1.548682
H	-3.201069	-1.831222	4.412922
H	-1.183089	-0.667550	3.480518
C	-2.762125	-0.556989	-0.700278
Ni	-0.182314	0.402965	0.118129
C	3.794625	0.954059	2.635953
C	3.484728	-0.229736	1.924532
C	2.246802	-0.260144	1.243062
N	1.366527	0.779164	1.237313
C	1.680363	1.879626	1.919129
C	2.888216	2.002129	2.632818
H	4.738469	1.032837	3.181167
H	0.960128	2.697737	1.900505
H	3.093173	2.926999	3.174565
C	2.233549	-3.661788	-0.285768
C	0.994171	-3.591335	-0.901055
C	0.225192	-2.416519	-0.791248
N	0.645997	-1.355092	-0.108758
C	1.855604	-1.418760	0.509744
C	2.701679	-2.549677	0.454088
H	2.845938	-4.563579	-0.362969
H	0.600175	-4.432791	-1.473389
H	-0.749350	-2.341757	-1.274030
C	3.962422	-2.497767	1.145444
C	4.337250	-1.386501	1.850533
H	5.294933	-1.363162	2.375287
H	4.617955	-3.370382	1.101946
H	-5.104413	-2.379619	2.869810
H	-4.422310	-0.701479	-3.694072
N	-0.787712	2.228334	0.153598
C	-1.702224	2.653887	1.044873
C	-0.291257	3.085156	-0.760626

C	-2.149742	3.972958	1.060680
C	-0.692930	4.418203	-0.804238
C	-1.636523	4.873022	0.121882
H	-2.070909	1.908401	1.750828
H	0.433670	2.668432	-1.465134
H	-2.891606	4.283345	1.798722
H	-0.269665	5.084884	-1.557806
H	-1.969989	5.913487	0.110336
N	1.925211	0.882368	-2.090633
C	2.979116	1.477957	-1.517603
C	2.143469	-0.252459	-2.767858
C	4.278296	0.967998	-1.578909
C	3.402302	-0.845940	-2.888606
C	4.494122	-0.222862	-2.277538
H	2.781594	2.408742	-0.973800
H	1.270933	-0.721271	-3.236333
H	5.101147	1.500090	-1.097203
H	3.522226	-1.769474	-3.458640
H	5.496360	-0.651404	-2.353877
C	-5.150633	-1.821051	0.178698
C	-5.012713	-1.480645	-1.136053
H	-5.810948	-1.690794	-1.851296
H	-6.061843	-2.309908	0.531099

67

Phen2Py2Pd+2 Binary file

C	-3.668294	-0.374620	-3.046922
C	-3.914044	-0.759238	-1.708413
C	-2.455728	0.195130	-3.389303
C	-1.487187	0.369139	-2.390102
N	-1.695696	0.006393	-1.119447
H	-2.235973	0.506996	-4.411490
H	-0.512816	0.813187	-2.616484
C	-4.583692	-1.888157	2.335469
C	-3.584996	-1.658610	3.262432
C	-4.381641	-1.518826	0.982560
C	-2.388782	-1.049413	2.824733
C	-3.132703	-0.920011	0.644036
N	-2.174417	-0.692305	1.568735
H	-3.708571	-1.933187	4.311704
H	-1.582094	-0.848198	3.538473
C	-2.888570	-0.542447	-0.739410
Pd	-0.099001	0.355988	0.167062
C	4.203153	0.773730	2.351131
C	3.778200	-0.413611	1.707852
C	2.480500	-0.414531	1.143600
N	1.654879	0.670848	1.201194
C	2.074918	1.776232	1.812271
C	3.349924	1.863215	2.402955
H	5.197158	0.821315	2.802717
H	1.386483	2.622578	1.835078
H	3.646131	2.791092	2.894974
C	2.232478	-3.864412	-0.270990
C	0.951777	-3.781927	-0.791833

C	0.224865	-2.582984	-0.662067
N	0.731338	-1.517480	-0.048094
C	1.986418	-1.582169	0.480859
C	2.789336	-2.743816	0.391490
H	2.814370	-4.784734	-0.364238
H	0.493471	-4.629989	-1.303165
H	-0.784354	-2.488424	-1.066147
C	4.106705	-2.719169	0.967697
C	4.581009	-1.601751	1.597488
H	5.582074	-1.596806	2.034357
H	4.724489	-3.617071	0.895883
H	-5.528685	-2.350848	2.631037
H	-4.444606	-0.530748	-3.800200
N	-0.747042	2.299201	0.310194
C	-1.564507	2.685797	1.310199
C	-0.365609	3.188605	-0.630296
C	-2.018754	3.998467	1.413741
C	-0.784521	4.516355	-0.587951
C	-1.622101	4.931670	0.451421
H	-1.857146	1.914535	2.025102
H	0.280578	2.807828	-1.426052
H	-2.678125	4.276920	2.237760
H	-0.456465	5.208952	-1.365190
H	-1.965198	5.967496	0.508188
N	1.888160	1.047077	-2.245449
C	2.909124	1.712572	-1.690444
C	2.138406	-0.167912	-2.749688
C	4.204803	1.197127	-1.598846
C	3.396875	-0.774237	-2.708885
C	4.453341	-0.076941	-2.116831
H	2.688497	2.709263	-1.291745
H	1.292694	-0.693508	-3.207753
H	5.000462	1.788909	-1.141842
H	3.543690	-1.765391	-3.142713
H	5.454201	-0.512928	-2.071079
C	-5.383792	-1.726453	-0.023343
C	-5.160072	-1.356109	-1.316108
H	-5.925440	-1.508951	-2.080077
H	-6.332335	-2.184029	0.267087

67

Phen2Py2Zn+2 Binary file

C	-4.258067	-0.238122	-2.511623
C	-3.915633	-1.013884	-1.377860
C	-3.382748	0.732928	-2.966113
C	-2.163359	0.916411	-2.287803
N	-1.813335	0.199882	-1.224300
H	-3.619244	1.349476	-3.834968
H	-1.449765	1.673129	-2.623609
C	-2.712318	-3.258927	2.035040
C	-1.492382	-2.961864	2.617496
C	-3.144119	-2.526952	0.902218
C	-0.715465	-1.922585	2.070058
C	-2.282107	-1.505967	0.417765

N	-1.101230	-1.216448	1.014852
H	-1.130045	-3.512084	3.487583
H	0.251483	-1.656321	2.507089
C	-2.666650	-0.745919	-0.753691
Zn	-0.000513	0.423003	-0.000537
C	4.256579	-0.237992	2.512604
C	3.914352	-1.014298	1.379217
C	2.665818	-0.746162	0.754271
N	1.812621	0.200163	1.223854
C	2.162375	0.917203	2.287179
C	3.381387	0.733718	2.966073
H	5.209350	-0.412461	3.018937
H	1.448682	1.674094	2.622416
H	3.617785	1.350713	3.834640
C	2.711297	-3.261079	-2.032786
C	1.491704	-2.963658	-2.615929
C	0.715215	-1.923573	-2.069571
N	1.101005	-1.216930	-1.014631
C	2.281391	-1.506803	-0.416942
C	3.143035	-2.528598	-0.900346
H	3.344162	-4.056344	-2.434716
H	1.129419	-3.514286	-3.485782
H	-0.251409	-1.657019	-2.507145
C	4.394114	-2.776300	-0.237809
C	4.766936	-2.045172	0.853284
H	5.721514	-2.236264	1.348685
H	5.045177	-3.562505	-0.626706
H	-3.345549	-4.053505	2.437758
H	-5.211130	-0.412561	-3.017422
N	-1.025561	1.904092	1.237619
C	-1.296111	1.671875	2.534153
C	-1.548115	3.000652	0.661439
C	-2.081735	2.526789	3.305195
C	-2.346823	3.908549	1.354051
C	-2.617999	3.669317	2.704321
H	-0.871270	0.761145	2.961666
H	-1.314397	3.148277	-0.395365
H	-2.270428	2.296363	4.355392
H	-2.747685	4.785009	0.841487
H	-3.239601	4.360629	3.278428
N	1.025022	1.903201	-1.239756
C	1.549773	2.998821	-0.663772
C	1.296347	1.669486	-2.535863
C	2.351571	3.904196	-1.356110
C	2.085076	2.521806	-3.306598
C	2.623695	3.663316	-2.705891
H	1.315300	3.147835	0.392667
H	0.869681	0.759557	-2.963278
H	2.754090	4.779988	-0.843703
H	2.274217	2.290223	-4.356457
H	3.247811	4.352583	-3.279728
C	-4.395551	-2.774633	0.240374
C	-4.768556	-2.043924	-0.850896

H -5.723467 -2.234704 -1.345769
H -5.046488 -3.560690 0.629769
22
PHENCl SCF Done: -1030.68173011 A.U.
C -2.709423 -1.454158 -0.000001
C -1.390232 -0.941116 -0.000002
C -1.230162 0.475858 0.000002
N -2.282202 1.318954 0.000002
C -3.500375 0.811847 -0.000003
C -3.775051 -0.575186 0.000003
H -2.867275 -2.536211 -0.000001
H -4.331258 1.528770 0.000011
H -4.807453 -0.931829 0.000009
C 2.537638 0.726929 0.000006
C 2.659725 2.102839 0.000003
C 1.482186 2.882173 -0.000003
N 0.267461 2.369051 -0.000005
C 0.122245 1.028671 -0.000001
C 1.248269 0.145182 0.000002
H 3.416774 0.081145 0.000009
H 3.640286 2.583789 0.000006
H 1.553752 3.977306 -0.000012
C 1.016791 -1.279536 -0.000001
C -0.243643 -1.801419 -0.000003
H -0.386207 -2.883623 -0.000005
Cl 2.386868 -2.374582 -0.000001
31
PhenNO32Co SCF Done: -2514.16563942 A.U.
Co 1.070551 -0.000110 0.000019
C -2.721753 -2.741136 0.764355
C -2.823093 -1.380994 0.384169
C -1.608356 -0.688301 0.190273
N -0.384334 -1.255480 0.351010
C -0.309313 -2.532833 0.710093
C -1.467255 -3.308827 0.925443
H -3.627920 -3.329792 0.927698
H 0.700661 -2.933933 0.825617
H -1.357101 -4.354275 1.218512
C -2.721368 2.741483 -0.764314
C -1.466790 3.308973 -0.925484
C -0.308958 2.532803 -0.710181
N -0.384156 1.255467 -0.351070
C -1.608259 0.688482 -0.190264
C -2.822899 1.381361 -0.384105
H -3.627451 3.330277 -0.927620
H -1.356487 4.354398 -1.218581
H 0.701070 2.933751 -0.825762
O 1.686756 1.050048 1.884225
O 1.686291 -1.049969 -1.883956
N 2.457036 1.724341 1.154914
O 3.182951 2.601130 1.549183
O 2.407523 1.417927 -0.113086
N 2.456508 -1.724657 -1.154957

O 2.407245 -1.418363 0.113068
O 3.182290 -2.601435 -1.549504
C -4.052891 0.660111 -0.183128
C -4.052984 -0.659551 0.183263
H -4.996891 1.190172 -0.330542
H -4.997059 -1.189466 0.330722
31
PhenNO32Cu SCF Done: -2771.82361581 A.U.
Cu -1.166814 0.000181 -0.000130
C 2.754094 -2.709126 -0.866897
C 2.840614 -1.362320 -0.436084
C 1.617041 -0.685036 -0.222320
N 0.413166 -1.268546 -0.420153
C 0.346499 -2.528655 -0.821194
C 1.511313 -3.290239 -1.056606
H 3.669784 -3.278844 -1.045125
H -0.661984 -2.931651 -0.952933
H 1.416599 -4.326337 -1.385861
C 2.755235 2.707739 0.867205
C 1.512697 3.289323 1.057083
C 0.347575 2.528148 0.821898
N 0.413707 1.267983 0.420959
C 1.617328 0.684027 0.222938
C 2.841187 1.360878 0.436455
H 3.671164 3.277136 1.045233
H 1.418422 4.325480 1.386277
H -0.660732 2.931560 0.953726
O -1.680572 1.292870 -1.930091
O -1.683752 -1.291389 1.930029
N -2.408636 1.947175 -1.155724
O -3.038330 2.929908 -1.468860
O -2.453283 1.522102 0.082014
N -2.411239 -1.945609 1.155131
O -2.453935 -1.521316 -0.082959
O -3.042043 -2.927735 1.468012
C 4.071777 0.650892 0.207582
C 4.071503 -0.652783 -0.207424
H 5.015000 1.176517 0.374526
H 5.014505 -1.178745 -0.374551
31
PhenNO32Fe3 SCF Done: -2395.11161899 A.U.
Fe -1.099599 0.000390 0.000171
C 2.734666 -2.737737 -0.769784
C 2.833699 -1.379723 -0.387449
C 1.617788 -0.686235 -0.192462
N 0.393191 -1.255977 -0.354798
C 0.322714 -2.535804 -0.717427
C 1.479606 -3.308190 -0.933595
H 3.641670 -3.324922 -0.933542
H -0.685142 -2.940712 -0.838497
H 1.372180 -4.353406 -1.228512
C 2.734802 2.738486 0.769123
C 1.479774 3.309016 0.932954

C	0.322839	2.536726	0.716678
N	0.393286	1.256919	0.353947
C	1.617837	0.687090	0.191620
C	2.833784	1.380496	0.386695
H	3.641834	3.325605	0.932972
H	1.372430	4.354212	1.227971
H	-0.685025	2.941614	0.837745
O	-1.675872	1.223728	-1.828390
O	-1.671717	-1.225734	1.828678
N	-2.480846	1.833388	-1.075739
O	-3.194897	2.737218	-1.428809
O	-2.478346	1.424343	0.160191
N	-2.477606	-1.835035	1.076678
O	-2.478404	-1.423791	-0.158472
O	-3.189735	-2.740407	1.429754
C	4.063910	0.659616	0.184153
C	4.063865	-0.658920	-0.184865
H	5.007809	1.189606	0.332583
H	5.007730	-1.188986	-0.333241

31

PhenNO32Fe5 SCF Done: -2395.13468444 A.U.

Fe	-1.342357	0.000346	0.000765
C	2.739592	-2.657682	-1.000470
C	2.807821	-1.334500	-0.503847
C	1.577257	-0.674378	-0.255030
N	0.376419	-1.261819	-0.475908
C	0.335038	-2.507685	-0.934046
C	1.503393	-3.243996	-1.214142
H	3.662972	-3.204829	-1.207383
H	-0.664577	-2.930634	-1.068454
H	1.418064	-4.264659	-1.591061
C	2.740330	2.657835	0.999610
C	1.504293	3.244526	1.213183
C	0.335733	2.508397	0.933427
N	0.376796	1.262381	0.475702
C	1.577458	0.674627	0.254748
C	2.808203	1.334517	0.503290
H	3.663862	3.204800	1.206329
H	1.419267	4.265328	1.589796
H	-0.663779	2.931603	1.067835
O	-1.699459	1.010831	-1.907097
O	-1.697717	-1.011713	1.907462
N	-2.360944	1.928941	-1.330104
O	-2.814916	2.882972	-1.904998
O	-2.498762	1.769543	-0.053279
N	-2.359420	-1.929704	1.330361
O	-2.498690	-1.769155	0.053868
O	-2.812009	-2.884587	1.904914
C	4.039313	0.639146	0.240744
C	4.039127	-0.639442	-0.241383
H	4.981467	1.157130	0.435854
H	4.981126	-1.157659	-0.436622

31

PhenNO32Fe7 SCF Done: -2395.06872473 A.U.

Fe	-1.125014	-0.002337	-0.001392
C	2.796300	-2.722926	-0.786769
C	2.875549	-1.382258	-0.400329
C	1.653286	-0.671757	-0.196201
N	0.419190	-1.285052	-0.374950
C	0.378544	-2.585032	-0.744346
C	1.526315	-3.330312	-0.958435
H	3.711431	-3.296116	-0.952870
H	-0.620913	-3.013379	-0.856753
H	1.439993	-4.376402	-1.254729
C	2.792735	2.724700	0.788021
C	1.522334	3.330491	0.959081
C	0.375332	2.583628	0.743711
N	0.417802	1.284492	0.373834
C	1.652494	0.672472	0.195972
C	2.873979	1.384303	0.401201
H	3.707139	3.298853	0.954979
H	1.434473	4.376313	1.255827
H	-0.624670	3.010854	0.855502
O	-1.793199	0.440152	-1.922731
O	-1.789787	-0.442733	1.922516
N	-2.543967	1.414167	-1.535930
O	-3.184811	2.083567	-2.285474
O	-2.523669	1.586285	-0.263293
N	-2.544991	-1.413377	1.536740
O	-2.528059	-1.584566	0.263676
O	-3.187021	-2.080986	2.286842
C	4.103111	0.658010	0.190802
C	4.103823	-0.654505	-0.189090
H	5.047450	1.186822	0.343788
H	5.048767	-1.182433	-0.341400

31

PhenNO32Fe9 SCF Done: -2394.97306217 A.U.

Fe	-1.115351	-0.001149	-0.000888
C	2.784645	-2.782705	-0.783282
C	2.882361	-1.409435	-0.397332
C	1.647573	-0.688062	-0.195825
N	0.433365	-1.278997	-0.364218
C	0.366257	-2.621182	-0.733866
C	1.501478	-3.366306	-0.942547
H	3.693320	-3.364137	-0.947689
H	-0.642091	-3.025301	-0.832935
H	1.403785	-4.414716	-1.230282
C	2.781456	2.785019	0.783245
C	1.497613	3.367233	0.942085
C	0.363239	2.620904	0.733110
N	0.431838	1.278753	0.363566
C	1.646744	0.689183	0.195550
C	2.880727	1.411853	0.397364
H	3.689465	3.367410	0.947931
H	1.398683	4.415548	1.229748
H	-0.645557	3.023945	0.831851

O -1.776327 0.432755 -1.927659
O -1.772186 -0.434997 1.927886
N -2.533684 1.404250 -1.546940
O -3.174228 2.068463 -2.301133
O -2.520595 1.579176 -0.274564
N -2.531333 -1.405420 1.548201
O -2.520884 -1.579641 0.275615
O -3.171254 -2.069355 2.303158
C 4.067855 0.713097 0.200006
C 4.068681 -0.709417 -0.199649
H 5.025411 1.218872 0.341703
H 5.026821 -1.214160 -0.341079
31
PhenNO32Fe SCF Done: -2395.11737413 A.U.
Fe -1.136520 0.000132 0.000036
C 2.661848 -2.722992 -0.825589
C 2.758869 -1.372116 -0.415606
C 1.543197 -0.684192 -0.203603
N 0.316839 -1.249840 -0.368318
C 0.249277 -2.522803 -0.752461
C 1.406885 -3.289576 -0.993061
H 3.569061 -3.305215 -1.004900
H -0.756962 -2.933623 -0.863267
H 1.297763 -4.329014 -1.307611
C 2.662326 2.722984 0.825121
C 1.407451 3.289981 0.991877
C 0.249714 2.523475 0.751196
N 0.316972 1.250301 0.367615
C 1.543272 0.684317 0.203436
C 2.759091 1.371950 0.415619
H 3.569620 3.305024 1.004611
H 1.298501 4.329588 1.305935
H -0.756445 2.934591 0.861601
O -1.489471 0.747465 -1.805126
O -1.488219 -0.748016 1.805538
N -2.359389 1.572597 -1.345256
O -2.961425 2.355416 -2.026042
O -2.504386 1.471446 -0.071202
N -2.358507 -1.572965 1.345977
O -2.504934 -1.470957 0.072112
O -2.959715 -2.356314 2.026882
C 3.988771 0.654763 0.199418
C 3.988677 -0.655290 -0.198931
H 4.932846 1.181022 0.360179
H 4.932676 -1.181776 -0.359385
31
PhenNO32Ni SCF Done: -2639.67723755 A.U.
Ni -0.989576 -0.000137 -0.000134
C 2.768296 -2.769341 -0.659011
C 2.876528 -1.395818 -0.330155
C 1.664275 -0.694507 -0.163530
N 0.438762 -1.261227 -0.304165
C 0.355021 -2.547849 -0.622285

C	1.511585	-3.335832	-0.804067
H	3.671351	-3.368871	-0.798334
H	-0.655975	-2.943930	-0.742925
H	1.396830	-4.390120	-1.061150
C	2.768044	2.769328	0.659196
C	1.511284	3.335748	0.804072
C	0.354790	2.547678	0.622188
N	0.438636	1.261061	0.304097
C	1.664210	0.694413	0.163614
C	2.876395	1.395800	0.330387
H	3.671048	3.368912	0.798616
H	1.396422	4.390041	1.061088
H	-0.656223	2.943745	0.742724
O	-1.929915	1.491286	-1.899604
O	-1.930435	-1.490748	1.899418
N	-2.547679	1.911912	-0.920062
O	-3.348942	2.819357	-0.925914
O	-2.286941	1.338672	0.248380
N	-2.548241	-1.911690	0.920061
O	-2.287031	-1.339150	-0.248704
O	-3.349947	-2.818737	0.926283
C	4.106322	0.666797	0.157395
C	4.106385	-0.666736	-0.157000
H	5.050538	1.201700	0.283848
H	5.050653	-1.201580	-0.283323

31

PhenNO32PdCl SCF Done: -1718.86646312 A.U.

Pd	1.375902	-0.122094	0.004520
C	-2.945534	-2.220725	0.487296
C	-2.796090	-0.840777	0.210818
C	-1.474895	-0.347020	0.096571
N	-0.383153	-1.146339	0.249739
C	-0.538451	-2.435773	0.522476
C	-1.819001	-3.009481	0.643077
H	-3.946042	-2.645439	0.578804
H	0.372128	-3.019250	0.665872
H	-1.905007	-4.074704	0.862603
C	-1.987494	3.285574	-0.612862
C	-0.659809	3.660152	-0.722417
C	0.357183	2.697107	-0.558948
N	0.071730	1.429069	-0.289818
C	-1.227135	1.037864	-0.181993
C	-2.308010	1.932917	-0.339576
H	-2.788562	4.017924	-0.738509
H	-0.380552	4.692714	-0.937655
H	1.412876	2.950567	-0.664264
O	2.558801	1.929052	1.642211
O	1.852949	-2.449805	-1.617024
N	3.325940	1.851811	0.685985
O	4.337492	2.499879	0.536771
O	3.015445	1.014370	-0.306018
N	2.569826	-2.636560	-0.637192
O	2.525813	-1.745006	0.355525

O	3.299241	-3.587228	-0.465468
C	-3.645748	1.427696	-0.219659
C	-3.879224	0.103362	0.040135
H	-4.486775	2.112147	-0.340345
Cl	-5.519567	-0.466998	0.173360

31

PhenNO32Pd SCF Done: -1259.40110119 A.U.

Pd	-0.949879	-0.000157	-0.000162
C	2.952099	-2.786841	-0.563643
C	3.036280	-1.401643	-0.281599
C	1.815633	-0.702284	-0.142987
N	0.603227	-1.307473	-0.276804
C	0.541576	-2.603932	-0.554109
C	1.709178	-3.380734	-0.699888
H	3.867493	-3.373185	-0.675309
H	-0.454223	-3.031247	-0.680819
H	1.612211	-4.444444	-0.922646
C	2.951442	2.787418	0.563782
C	1.708374	3.381050	0.699841
C	0.540956	2.604018	0.553869
N	0.602897	1.307566	0.276557
C	1.815458	0.702638	0.142931
C	3.035944	1.402243	0.281736
H	3.866695	3.373954	0.675595
H	1.611150	4.444737	0.922599
H	-0.454934	3.031160	0.680407
O	-1.803639	2.220308	-1.622501
O	-1.803046	-2.219985	1.622770
N	-2.546069	2.280868	-0.645910
O	-3.421253	3.099852	-0.473192
O	-2.364333	1.401299	0.340936
N	-2.545401	-2.281240	0.646168
O	-2.363728	-1.402201	-0.341177
O	-3.420440	-3.100466	0.473856
C	4.266212	0.671297	0.133516
C	4.266375	-0.670447	-0.133190
H	5.208756	1.213043	0.241191
H	5.209051	-1.211991	-0.240719

31

PhenNO32Zn SCF Done: -2910.72302498 A.U.

Zn	-1.276854	-0.000268	-0.000311
C	2.778407	-2.672567	-0.964286
C	2.850410	-1.341687	-0.485476
C	1.619806	-0.679475	-0.248283
N	0.424158	-1.268440	-0.467214
C	0.373671	-2.517250	-0.906164
C	1.542849	-3.261117	-1.171907
H	3.701108	-3.224267	-1.162502
H	-0.628954	-2.935028	-1.036026
H	1.458080	-4.286875	-1.534734
C	2.778236	2.672285	0.964573
C	1.542632	3.260821	1.171964
C	0.373504	2.516761	0.906569

N	0.424026	1.267760	0.468145
C	1.619733	0.678886	0.249258
C	2.850305	1.341251	0.486210
H	3.700896	3.224127	1.162582
H	1.457780	4.286733	1.534335
H	-0.629135	2.934526	1.036360
O	-1.713278	0.836110	-1.885905
O	-1.715752	-0.835141	1.885869
N	-2.368732	1.812760	-1.380832
O	-2.830459	2.699772	-2.052141
O	-2.486028	1.779324	-0.104640
N	-2.370634	-1.811781	1.380323
O	-2.486162	-1.778766	0.103914
O	-2.833422	-2.698581	2.051190
C	4.081607	0.642667	0.232322
C	4.081666	-0.643034	-0.231558
H	5.023935	1.163165	0.419636
H	5.024040	-1.163435	-0.418905

45

PhenPy2Co+2 SCF Done: -2449.73246905 A.U.

C	-3.817615	-3.255556	0.181417
C	-3.043381	-2.962598	1.309010
C	-3.552875	-2.581417	-1.014868
C	-2.530066	-1.637050	-1.042409
N	-1.782262	-1.362225	0.044456
H	-3.215045	-3.464537	2.263126
H	-4.131465	-2.779640	-1.919112
H	-1.393866	-1.766267	2.053745
H	-2.297955	-1.081848	-1.954881
C	-3.817616	3.255556	-0.181417
C	-3.552875	2.581417	1.014868
C	-3.043382	2.962597	-1.309010
C	-2.530066	1.637050	1.042409
C	-2.032087	2.012199	-1.199996
N	-1.782263	1.362225	-0.044456
H	-4.131465	2.779640	1.919112
H	-3.215046	3.464536	-2.263126
H	-2.297954	1.081849	1.954881
H	-1.393867	1.766266	-2.053745
C	-2.032086	-2.012199	1.199996
Co	-0.389948	0.000000	0.000000
C	3.440148	2.829310	0.255195
C	3.524024	1.421114	0.125768
C	2.302957	0.711376	0.057565
N	1.079897	1.316342	0.097042
C	1.032264	2.643785	0.227617
C	2.194334	3.433391	0.310175
H	4.353353	3.426969	0.313346
H	0.044883	3.104440	0.269265
H	2.095676	4.515113	0.415616
C	3.440148	-2.829310	-0.255196
C	2.194334	-3.433391	-0.310176
C	1.032264	-2.643784	-0.227617

N	1.079897	-1.316342	-0.097043
C	2.302957	-0.711376	-0.057565
C	3.524024	-1.421114	-0.125768
H	4.353353	-3.426968	-0.313346
H	2.095676	-4.515113	-0.415616
H	0.044883	-3.104440	-0.269265
C	4.756137	-0.681416	-0.061886
C	4.756137	0.681416	0.061886
H	5.698561	1.231131	0.112702
H	5.698561	-1.231131	-0.112701
H	-4.616327	3.999396	-0.234505
H	-4.616326	-3.999397	0.234505

45

PhenPy2Cu+2 SCF Done: -2707.40032039 A.U.

C	3.713698	-3.457906	-0.648647
C	2.648580	-3.293195	-1.540508
C	3.819441	-2.597525	0.449044
C	2.863380	-1.598628	0.614230
N	1.836181	-1.447381	-0.245016
H	2.529772	-3.941058	-2.411138
H	4.633290	-2.693615	1.170304
H	0.868607	-2.124500	-1.963447
H	2.913363	-0.894973	1.448243
C	3.713703	3.457897	0.648648
C	3.819455	2.597496	-0.449026
C	2.648556	3.293225	1.540483
C	2.863381	1.598614	-0.614220
C	1.724596	2.279469	1.301128
N	1.836158	1.447398	0.245007
H	4.633323	2.693561	-1.170270
H	2.529734	3.941109	2.411096
H	2.913371	0.894945	-1.448220
H	0.868545	2.124576	1.963392
C	1.724632	-2.279427	-1.301159
Cu	0.449693	0.000007	-0.000000
C	-3.444485	2.724870	-0.804087
C	-3.521574	1.368461	-0.400781
C	-2.296325	0.689632	-0.195440
N	-1.087040	1.293411	-0.355105
C	-1.038828	2.567764	-0.738541
C	-2.204655	3.319451	-0.977516
H	-4.362544	3.292137	-0.977606
H	-0.048413	3.011053	-0.860373
H	-2.115572	4.360308	-1.293366
C	-3.444475	-2.724884	0.804089
C	-2.204643	-3.319450	0.977551
C	-1.038818	-2.567759	0.738577
N	-1.087032	-1.293413	0.355117
C	-2.296320	-0.689645	0.195433
C	-3.521567	-1.368481	0.400766
H	-4.362531	-3.292154	0.977608
H	-2.115556	-4.360299	1.293428
H	-0.048403	-3.011041	0.860431

C	-4.753723	-0.655813	0.194169
C	-4.753726	0.655783	-0.194202
H	-5.696037	1.184936	-0.352502
H	-5.696030	-1.184975	0.352456
H	4.452203	4.247681	0.807253
H	4.452188	-4.247700	-0.807249

45

PhenPy2Fe+2Q SCF Done: -2330.69055378 A.U.

C	-3.262664	4.083820	-0.422169
C	-3.766632	2.873412	-0.911050
C	-2.007118	4.098396	0.194314
C	-1.299505	2.905304	0.301118
N	-1.786409	1.735593	-0.168594
H	-4.741103	2.819985	-1.400118
H	-1.578678	5.022044	0.587867
H	-3.354714	0.756140	-1.134619
H	-0.314029	2.869473	0.772997
C	-3.265980	-4.081899	0.421996
C	-2.010171	-4.097521	-0.193927
C	-3.769208	-2.871052	0.910551
C	-1.301568	-2.904999	-0.300517
C	-3.001248	-1.720719	0.765091
N	-1.787754	-1.734863	0.168886
H	-1.582285	-5.021541	-0.587209
H	-4.743853	-2.816814	1.399182
H	-0.315856	-2.869986	-0.771966
H	-3.355709	-0.754091	1.134143
C	-2.999647	1.722459	-0.765340
Fe	-0.687874	-0.000065	0.000273
C	3.281681	0.332309	2.818786
C	3.340877	0.166296	1.412480
C	2.108108	0.085124	0.716242
N	0.905093	0.165701	1.356061
C	0.880209	0.323708	2.680490
C	2.052131	0.410461	3.452487
H	4.208850	0.397489	3.394082
H	-0.104850	0.382849	3.151780
H	1.978385	0.537427	4.533927
C	3.280898	-0.333674	-2.819194
C	2.051170	-0.411532	-3.452586
C	0.879462	-0.324479	-2.680296
N	0.904721	-0.166461	-1.355879
C	2.107912	-0.086180	-0.716360
C	3.340487	-0.167662	-1.412904
H	4.207906	-0.399089	-3.394720
H	1.977123	-0.538498	-4.534006
H	-0.105731	-0.383392	-3.151334
C	4.573413	-0.080525	-0.678934
C	4.573600	0.078857	0.678204
H	5.515182	0.143098	1.228015
H	5.514844	-0.145001	-1.228977
H	-3.845819	-5.002867	0.521509
H	-3.841731	5.005254	-0.521861

45
PhenPy2Fe+2 SCF Done: -2330.62710222 A.U.

C	-3.843099	3.283442	0.271659
C	-3.699829	2.518141	-0.889951
C	-2.949897	3.081378	1.329109
C	-1.944349	2.129826	1.183499
N	-1.811092	1.387510	0.064082
H	-4.373579	2.643499	-1.739678
H	-3.024115	3.655733	2.254486
H	-2.543492	0.945350	-1.835646
H	-1.211825	1.958652	1.979088
C	-3.842998	-3.283593	-0.272551
C	-2.948860	-3.081938	-1.329287
C	-3.700668	-2.517934	0.888938
C	-1.943353	-2.130434	-1.183098
C	-2.677885	-1.575156	0.951362
N	-1.811001	-1.387759	-0.063801
H	-3.022326	-3.656575	-2.254549
H	-4.375161	-2.642968	1.738122
H	-1.210115	-1.959588	-1.978097
H	-2.545042	-0.944921	1.835126
C	-2.677059	1.575310	-0.951788
Fe	-0.405852	-0.000073	0.000359
C	3.467952	-2.814847	0.377023
C	3.546264	-1.413271	0.186388
C	2.322793	-0.709790	0.086041
N	1.101986	-1.319670	0.144931
C	1.061390	-2.640651	0.335042
C	2.225162	-3.421969	0.457138
H	4.384030	-3.404803	0.462405
H	0.074707	-3.102036	0.393223
H	2.131171	-4.498256	0.611272
C	3.467511	2.815167	-0.377158
C	2.224626	3.422164	-0.456752
C	1.060982	2.640702	-0.334361
N	1.101791	1.319705	-0.144421
C	2.322684	0.709957	-0.085965
C	3.546043	1.413582	-0.186674
H	4.383495	3.405230	-0.462810
H	2.130463	4.498462	-0.610702
H	0.074226	3.101988	-0.392110
C	4.778345	0.678055	-0.092201
C	4.778452	-0.677608	0.091484
H	5.720706	-1.224825	0.166652
H	5.720513	1.225376	-0.167688
H	-4.639214	-4.027576	-0.353325
H	-4.639321	4.027469	0.351976

45
PhenPy2Ni+2 SCF Done: -2575.26977701 A.U.

C	3.777394	3.234298	-0.000327
C	3.260097	2.748064	1.204792
C	3.259537	2.748186	-1.205255
C	2.245855	1.794487	-1.165818

N	1.752516	1.332741	0.000048
H	3.634063	3.100932	2.167799
H	3.633062	3.101147	-2.168399
H	1.810275	1.391318	2.082917
H	1.809291	1.391549	-2.082844
C	3.777334	-3.234344	-0.000256
C	3.259950	-2.748166	1.204849
C	3.259593	-2.748144	-1.205198
C	2.246285	-1.794435	1.165759
C	2.245937	-1.794415	-1.165789
N	1.752511	-1.332727	0.000063
H	3.633826	-3.101102	2.167866
H	3.633186	-3.101059	-2.168332
H	1.810086	-1.391440	2.082933
H	1.809471	-1.391400	-2.082827
C	2.246403	1.794361	1.165730
Ni	0.379948	0.000009	0.000248
C	-3.406401	-2.841374	-0.000098
C	-3.495747	-1.427692	-0.000117
C	-2.277129	-0.712207	0.000029
N	-1.051622	-1.311673	0.000175
C	-0.997282	-2.644770	0.000198
C	-2.157508	-3.441899	0.000068
H	-4.316639	-3.446313	-0.000213
H	-0.009510	-3.105274	0.000334
H	-2.053876	-4.528281	0.000101
C	-3.406403	2.841382	-0.000001
C	-2.157511	3.441908	0.000185
C	-0.997284	2.644780	0.000288
N	-1.051625	1.311683	0.000216
C	-2.277130	0.712216	0.000052
C	-3.495749	1.427700	-0.000068
H	-4.316641	3.446320	-0.000094
H	-2.053880	4.528290	0.000257
H	-0.009512	3.105283	0.000445
C	-4.727697	0.684406	-0.000234
C	-4.727696	-0.684399	-0.000258
H	-5.670339	-1.236082	-0.000384
H	-5.670341	1.236088	-0.000339
H	4.573660	-3.982647	-0.000381
H	4.573741	3.982579	-0.000474

53

PhenPy2NO32Co SCF Done: -3010.45576791 A.U.

C	4.050965	3.075053	-0.849655
C	3.317889	3.158778	0.335425
C	3.701962	2.097782	-1.785348
C	2.640403	1.240356	-1.503075
N	1.941788	1.326430	-0.355610
H	3.545337	3.908878	1.095218
H	4.240366	1.994550	-2.729371
H	1.649415	2.300376	1.454704
H	2.309766	0.466111	-2.198691
C	4.050992	-3.074928	0.849832

C	3.701780	-2.097807	1.785602
C	3.318111	-3.158535	-0.335377
C	2.640218	-1.240402	1.503276
C	2.268176	-2.265395	-0.553266
N	1.941795	-1.326358	0.355684
H	4.240025	-1.994673	2.729727
H	3.545726	-3.908518	-1.095235
H	2.309428	-0.466269	2.198944
H	1.649768	-2.300095	-1.454809
C	2.267972	2.265604	0.553267
Co	0.506933	0.000023	0.000002
C	-3.332768	-2.728385	0.774562
C	-3.413676	-1.371865	0.391766
C	-2.193481	-0.688255	0.194390
N	-0.980446	-1.273323	0.342754
C	-0.929730	-2.549548	0.701553
C	-2.088113	-3.313629	0.931115
H	-4.248254	-3.301957	0.939705
H	0.060724	-2.989865	0.811594
H	-1.986826	-4.360370	1.221785
C	-3.332882	2.728270	-0.774628
C	-2.088251	3.313553	-0.931227
C	-0.929835	2.549522	-0.701656
N	-0.980498	1.273311	-0.342805
C	-2.193510	0.688198	-0.194421
C	-3.413733	1.371758	-0.391794
H	-4.248392	3.301805	-0.939772
H	-1.987006	4.360287	-1.221940
H	0.060599	2.989873	-0.811733
O	-1.624514	0.892701	2.744910
O	-1.624373	-0.893006	-2.744970
N	-0.494518	1.316415	2.505649
O	-0.208929	2.521594	2.554272
O	0.428940	0.481092	2.163703
N	-0.494291	-1.316457	-2.505650
O	-0.208358	-2.521546	-2.554511
O	0.428988	-0.480926	-2.163732
C	-4.644886	0.655985	-0.189428
C	-4.644858	-0.656135	0.189430
H	-5.586503	-1.188667	0.342595
H	-5.586553	1.188483	-0.342579
H	4.878949	-3.761668	1.044008
H	4.878913	3.761816	-1.043792

53

PhenPy2NO32Cu SCF Done: -3268.10922460 A.U.

C	-4.094394	3.081123	1.048489
C	-3.420805	3.182695	-0.170525
C	-3.689749	2.106962	1.965599
C	-2.629360	1.266249	1.629807
N	-1.993340	1.371538	0.450882
H	-3.696070	3.933779	-0.913370
H	-4.183409	1.995002	2.932830
H	-1.784492	2.350427	-1.365371

H	-2.253233	0.488867	2.300105
C	-4.094262	-3.078669	-1.053367
C	-3.684787	-2.107588	-1.971578
C	-3.425610	-3.177550	0.168583
C	-2.624685	-1.267179	-1.633995
C	-2.373839	-2.300262	0.440402
N	-1.993467	-1.369834	-0.452273
H	-4.174437	-1.997729	-2.941085
H	-3.704634	-3.926203	0.912478
H	-2.245232	-0.492323	-2.305335
H	-1.793187	-2.344002	1.367602
C	-2.369001	2.304916	-0.440675
Cu	-0.515334	0.000431	0.000468
C	3.419515	-2.746869	-0.706484
C	3.489409	-1.378796	-0.360716
C	2.262867	-0.694985	-0.186193
N	1.065263	-1.300311	-0.348114
C	1.019701	-2.584898	-0.662610
C	2.184144	-3.351487	-0.854975
H	4.342236	-3.315667	-0.847017
H	0.028977	-3.029002	-0.765650
H	2.095396	-4.408515	-1.110104
C	3.421857	2.744376	0.707766
C	2.186987	3.349758	0.857360
C	1.021914	2.584045	0.665396
N	1.066361	1.299567	0.350344
C	2.263458	0.693425	0.187641
C	3.490586	1.376374	0.361512
H	4.345056	3.312514	0.847826
H	2.099126	4.406728	1.113026
H	0.031561	3.028792	0.769278
O	1.602357	1.166234	-2.699058
O	1.597736	-1.170083	2.699231
N	0.449015	1.514602	-2.445677
O	0.111192	2.709028	-2.398132
O	-0.434986	0.617156	-2.186173
N	0.443129	-1.515012	2.447125
O	0.101431	-2.708443	2.401169
O	-0.438196	-0.614953	2.187326
C	4.721763	0.658682	0.172610
C	4.721202	-0.661899	-0.172804
H	5.662075	-1.199690	-0.311900
H	5.663092	1.195857	0.311001
H	-4.922175	-3.752130	-1.290275
H	-4.922444	3.754899	1.284025

53

PhenPy2NO32FeQ SCF Done: -2891.42532572 A.U.

C	-4.191677	3.257920	1.023802
C	-3.481536	3.343934	-0.175805
C	-3.835465	2.268588	1.944285
C	-2.787008	1.404021	1.629466
N	-2.108414	1.487541	0.472834
H	-3.718643	4.102581	-0.924497

H	-4.358741	2.163002	2.896869
H	-1.851385	2.467846	-1.334353
H	-2.457299	0.616110	2.311583
C	-4.190801	-3.259475	-1.021477
C	-3.836876	-2.269335	-1.941976
C	-3.479118	-3.345183	0.177242
C	-2.789000	-1.403747	-1.628067
C	-2.444099	-2.439050	0.417011
N	-2.108843	-1.487043	-0.472349
H	-4.361477	-2.163920	-2.893847
H	-3.714485	-4.104387	0.925918
H	-2.461033	-0.615149	-2.310180
H	-1.848480	-2.467699	1.334183
C	-2.445806	2.438833	-0.416441
Fe	-0.540872	0.000087	-0.001002
C	3.588496	-2.785449	-0.529708
C	3.644539	-1.396510	-0.269741
C	2.409964	-0.710111	-0.140223
N	1.220549	-1.339890	-0.262023
C	1.191144	-2.641006	-0.499154
C	2.360944	-3.411936	-0.643835
H	4.518418	-3.350848	-0.633315
H	0.201048	-3.097288	-0.574067
H	2.284499	-4.483440	-0.835993
C	3.589318	2.784779	0.530518
C	2.361959	3.411935	0.642982
C	1.191917	2.641601	0.496945
N	1.220949	1.340464	0.260030
C	2.410160	0.710051	0.139736
C	3.644951	1.395775	0.270775
H	4.519410	3.349691	0.635268
H	2.285851	4.483511	0.834872
H	0.201956	3.098390	0.570551
O	1.426901	0.878206	-2.818299
O	1.428367	-0.878980	2.816966
N	0.345592	1.346563	-2.491788
O	0.053708	2.537955	-2.597411
O	-0.556938	0.542791	-1.989718
N	0.346192	-1.345576	2.490901
O	0.052140	-2.536405	2.597598
O	-0.554810	-0.540566	1.988219
C	4.877064	0.669070	0.130989
C	4.876870	-0.670491	-0.128315
H	5.817714	-1.216597	-0.232221
H	5.818075	1.214652	0.236140
H	-5.007051	-3.954042	-1.236740
H	-5.008468	3.951635	1.239754

53

PhenPy2NO32Fe SCF Done: -2891.40683816 A.U.

C	-4.029358	3.189662	0.704034
C	-3.242028	3.255094	-0.446771
C	-3.745622	2.196411	1.644005
C	-2.693479	1.314793	1.402380

N	-1.933104	1.380007	0.291910
H	-3.417984	4.009401	-1.216229
H	-4.328638	2.097483	2.561861
H	-1.582541	2.344769	-1.515024
H	-2.420667	0.531284	2.111191
C	-4.031491	-3.181121	-0.728490
C	-3.732606	-2.193834	-1.670015
C	-3.259759	-3.242194	0.433094
C	-2.681252	-1.313767	-1.419348
C	-2.225665	-2.323414	0.615511
N	-1.935678	-1.374946	-0.298667
H	-4.303214	-2.098339	-2.595993
H	-3.448180	-3.991554	1.204426
H	-2.396801	-0.534754	-2.128541
H	-1.613035	-2.330011	1.518786
C	-2.208093	2.334441	-0.620669
Fe	-0.463354	0.000613	0.000780
C	3.387531	-2.744670	-0.736672
C	3.469241	-1.383855	-0.366618
C	2.250138	-0.696744	-0.174755
N	1.033636	-1.281083	-0.305527
C	0.983386	-2.558644	-0.665007
C	2.140809	-3.326966	-0.891039
H	4.302043	-3.320805	-0.898476
H	-0.008790	-2.996222	-0.778393
H	2.037369	-4.374038	-1.180778
C	3.395338	2.734466	0.740235
C	2.150289	3.320243	0.894992
C	0.990666	2.555208	0.669284
N	1.037249	1.277273	0.310471
C	2.252148	0.689472	0.179256
C	3.473201	1.373383	0.370283
H	4.311496	3.308138	0.901468
H	2.049867	4.367693	1.184402
H	-0.000248	2.995640	0.782421
O	1.515357	0.583948	-2.670637
O	1.499765	-0.605222	2.663987
N	0.395101	1.074705	-2.591614
O	0.084406	2.151220	-3.094939
O	-0.545429	0.420221	-1.957792
N	0.368121	-1.072766	2.606548
O	0.035056	-2.123834	3.147298
O	-0.561365	-0.419566	1.955533
C	4.703724	0.652228	0.180450
C	4.701809	-0.665975	-0.177705
H	5.642973	-1.201529	-0.324058
H	5.646418	1.185306	0.325978
H	-4.848937	-3.886915	-0.896728
H	-4.847081	3.896756	0.865357

53

PhenPy2NO32Ni SCF Done: -3135.95885614 A.U.

C	-3.718049	3.143235	1.254258
C	-3.373318	3.034466	-0.094748

C	-3.082092	2.317566	2.185907
C	-2.125859	1.406149	1.742126
N	-1.812208	1.313951	0.435109
H	-3.834001	3.668188	-0.854482
H	-3.316691	2.371323	3.250470
H	-2.050186	1.989377	-1.502429
H	-1.585852	0.722844	2.409082
C	-3.912108	-2.951683	-1.255993
C	-3.249533	-2.203391	-2.234165
C	-3.537618	-2.799265	0.079377
C	-2.237050	-1.330163	-1.845384
C	-2.515855	-1.906651	0.406135
N	-1.889814	-1.195805	-0.550560
H	-3.506826	-2.290631	-3.291265
H	-4.018787	-3.368697	0.876355
H	-1.674574	-0.718040	-2.554553
H	-2.156912	-1.760333	1.433052
C	-2.407957	2.103470	-0.476356
Ni	-0.480965	0.032484	-0.095651
C	3.217943	-2.862482	-0.624701
C	3.347503	-1.492327	-0.300111
C	2.153091	-0.755104	-0.171297
N	0.919842	-1.283887	-0.377162
C	0.821841	-2.576635	-0.656352
C	1.956428	-3.400347	-0.791883
H	4.111466	-3.483323	-0.727293
H	-0.178331	-2.985677	-0.777137
H	1.815428	-4.458136	-1.017062
C	3.368783	2.644732	0.732539
C	2.138542	3.266516	0.839519
C	0.960267	2.522087	0.642637
N	0.985463	1.224876	0.358063
C	2.189721	0.615315	0.209761
C	3.422853	1.270865	0.406269
H	4.295246	3.203783	0.885425
H	2.057943	4.330075	1.067238
H	-0.015341	2.997145	0.714629
O	0.088807	2.982936	-2.122712
O	-0.074973	-2.951822	1.965293
N	0.475311	1.825961	-2.366301
O	-0.390525	0.880752	-2.366572
O	1.664267	1.555946	-2.554421
N	0.146681	-1.813968	2.427397
O	-0.843629	-1.091501	2.767372
O	1.297930	-1.368609	2.527832
C	4.633014	0.511429	0.249608
C	4.596238	-0.814582	-0.082520
H	5.522322	-1.384330	-0.189157
H	5.588569	1.017800	0.404342
H	-4.708476	-3.646736	-1.534502
H	-4.470631	3.867112	1.577588

53

PhenPy2NO32Pd SCF Done: -1755.68748500 A.U.

C	3.732883	3.267111	1.228568
C	3.111529	2.462395	2.188101
C	3.401965	3.095266	-0.116487
C	2.457650	2.131769	-0.472311
N	1.866009	1.371128	0.470592
H	3.342405	2.556879	3.250567
H	3.859914	3.701537	-0.899836
H	1.665973	0.834819	2.471891
H	2.144926	1.961051	-1.510477
C	3.732703	-3.267122	-1.228357
C	3.402475	-3.094513	0.116768
C	3.110652	-2.463139	-2.188054
C	2.458137	-2.131006	0.472488
C	2.175906	-1.515784	-1.776918
N	1.865869	-1.371031	-0.470554
H	3.860970	-3.700210	0.900239
H	3.340953	-2.558274	-3.250585
H	2.145777	-1.959837	1.510683
H	1.664807	-0.835987	-2.472170
C	2.176725	1.515129	1.776874
Pd	0.417802	0.000093	0.000054
C	-3.481744	-2.793912	-0.497033
C	-3.560998	-1.401578	-0.256825
C	-2.341705	-0.699516	-0.143186
N	-1.134071	-1.304810	-0.297812
C	-1.077891	-2.613064	-0.499120
C	-2.243849	-3.396077	-0.608258
H	-4.400009	-3.380028	-0.584250
H	-0.090059	-3.062522	-0.581263
H	-2.145938	-4.469626	-0.773689
C	-3.481468	2.794451	0.497127
C	-2.243514	3.396491	0.608348
C	-1.077630	2.613361	0.499231
N	-1.133947	1.305113	0.297892
C	-2.341641	0.699944	0.143236
C	-3.560865	1.402122	0.256916
H	-4.399675	3.380653	0.584381
H	-2.145494	4.470035	0.773751
H	-0.089752	3.062710	0.581420
O	-1.134927	-0.932346	2.657297
O	-1.133771	0.930225	-2.657400
N	-0.083449	-1.590502	2.591017
O	1.018174	-1.059950	2.931135
O	-0.078756	-2.760202	2.155407
N	-0.082985	1.589632	-2.591381
O	1.019131	1.060282	-2.931584
O	-0.079628	2.759391	-2.156022
C	-4.791888	0.673313	0.118621
C	-4.791949	-0.672656	-0.118485
H	-5.733049	-1.219549	-0.211746
H	-5.732936	1.220284	0.211953
H	4.467729	-4.019058	-1.527213
H	4.467910	4.019032	1.527463

53
PhenPy2NO32Zn SCF Done: -3407.00603942 A.U.

C	-4.141487	3.235107	0.969890
C	-3.331936	3.428642	-0.153383
C	-3.866620	2.164850	1.830284
C	-2.796029	1.323034	1.532368
N	-2.034150	1.506968	0.440830
H	-3.502482	4.252394	-0.845556
H	-4.471703	1.985342	2.721174
H	-1.601761	2.619114	-1.235369
H	-2.507649	0.479712	2.153838
C	-4.039288	-3.342537	-0.894541
C	-3.723994	-2.401343	-1.876557
C	-3.313820	-3.342755	0.301295
C	-2.694956	-1.490741	-1.631111
C	-2.304011	-2.400007	0.472732
N	-2.006131	-1.490731	-0.475783
H	-4.262449	-2.364907	-2.826647
H	-3.520557	-4.062066	1.093489
H	-2.395179	-0.725938	-2.351780
H	-1.691618	-2.338271	1.372892
C	-2.282997	2.535416	-0.383291
Zn	-0.496730	0.040490	-0.070951
C	3.570128	-2.768594	-0.684452
C	3.634103	-1.403916	-0.309461
C	2.406628	-0.703176	-0.199138
N	1.215714	-1.297735	-0.420588
C	1.178092	-2.571108	-0.769864
C	2.336581	-3.351401	-0.920691
H	4.490397	-3.343318	-0.781222
H	0.182507	-2.995118	-0.941688
H	2.252564	-4.400441	-1.208081
C	3.615045	2.704629	0.792893
C	2.394967	3.349121	0.883533
C	1.223230	2.621186	0.609673
N	1.240198	1.346331	0.266114
C	2.418354	0.693122	0.178273
C	3.659078	1.336480	0.434418
H	4.547274	3.236849	0.997647
H	2.330133	4.406170	1.159651
H	0.239159	3.096821	0.668794
O	1.368636	0.884734	-2.754277
O	1.409554	-1.167659	2.657215
N	0.241463	1.381383	-2.621426
O	-0.062365	2.519832	-3.009010
O	-0.682048	0.667267	-2.042973
N	0.194934	-1.323171	2.631935
O	-0.390244	-2.261294	3.181433
O	-0.535419	-0.459616	1.991583
C	4.883598	0.594071	0.314131
C	4.869544	-0.719957	-0.044681
H	5.804232	-1.282112	-0.134602
H	5.826275	1.107797	0.516831

H	-4.839699	-4.065844	-1.062301
H	-4.971909	3.910945	1.178206

45

PhenPy2Pd+2 SCF Done: -1195.00096196 A.U.

C	3.861695	3.346288	0.000005
C	3.345481	2.857654	1.203941
C	3.345373	2.857781	-1.203934
C	2.335021	1.900410	-1.166571
N	1.839989	1.434382	-0.000005
H	3.717299	3.210296	2.167805
H	3.717103	3.210525	-2.167795
H	1.905192	1.496504	2.086079
H	1.905007	1.496722	-2.086087
C	3.861715	-3.346272	-0.000007
C	3.345450	-2.857694	1.203929
C	3.345434	-2.857720	-1.203946
C	2.335084	-1.900339	1.166557
C	2.335071	-1.900360	-1.166580
N	1.839990	-1.434386	-0.000014
H	3.717233	-3.210374	2.167792
H	3.717204	-3.210420	-2.167807
H	1.905112	-1.496598	2.086070
H	1.905086	-1.496637	-2.086096
C	2.335123	1.900289	1.166566
Pd	0.364806	-0.000003	-0.000009
C	-3.555071	-2.840865	0.000003
C	-3.629663	-1.426121	-0.000002
C	-2.406238	-0.714739	0.000002
N	-1.192734	-1.343883	0.000008
C	-1.147133	-2.675869	0.000012
C	-2.316069	-3.459735	0.000011
H	-4.473611	-3.433065	-0.000002
H	-0.160492	-3.141647	0.000019
H	-2.226569	-4.547335	0.000015
C	-3.555071	2.840863	0.000018
C	-2.316069	3.459732	0.000028
C	-1.147133	2.675866	0.000021
N	-1.192733	1.343881	0.000009
C	-2.406237	0.714737	0.000004
C	-3.629662	1.426118	0.000006
H	-4.473611	3.433063	0.000019
H	-2.226569	4.547333	0.000040
H	-0.160492	3.141645	0.000027
C	-4.861406	0.683861	-0.000000
C	-4.861406	-0.683864	-0.000004
H	-5.803447	-1.236527	-0.000011
H	-5.803447	1.236525	-0.000003
H	4.655317	-4.097448	-0.000004
H	4.655289	4.097473	0.000010

45

PhenPy2Zn+2 SCF Done: -2846.29476414 A.U.

C	3.311062	3.909645	-0.738726
C	2.091721	4.000234	-0.059503

C	3.748219	2.658004	-1.185078
C	2.954075	1.543294	-0.936149
N	1.777304	1.631695	-0.278572
H	1.714183	4.957834	0.303868
H	4.692465	2.544009	-1.720844
H	0.393563	2.863098	0.672461
H	3.256718	0.546164	-1.266115
C	3.309800	-3.910400	0.738904
C	3.747489	-2.658826	1.184925
C	2.090309	-4.000684	0.059911
C	2.953705	-1.543880	0.935905
C	1.352045	-2.840243	-0.149470
N	1.776790	-1.631987	0.278550
H	4.691869	-2.545065	1.720504
H	1.712361	-4.958221	-0.303201
H	3.256762	-0.546793	1.265622
H	0.392446	-2.863079	-0.672004
C	1.353070	2.840019	0.149767
Zn	0.616047	0.000037	-0.000076
C	-3.302995	0.638523	2.766384
C	-3.362498	0.320516	1.386610
C	-2.129949	0.165913	0.702882
N	-0.929695	0.320992	1.330096
C	-0.900920	0.619626	2.629285
C	-2.073429	0.786424	3.387326
H	-4.229980	0.764645	3.331708
H	0.084163	0.731682	3.090309
H	-1.999464	1.029334	4.448718
C	-3.303177	-0.637995	-2.766370
C	-2.073651	-0.786025	-3.387363
C	-0.901094	-0.619344	-2.629373
N	-0.929784	-0.320700	-1.330184
C	-2.129995	-0.165497	-0.702919
C	-3.362589	-0.319978	-1.386595
H	-4.230198	-0.764023	-3.331654
H	-1.999757	-1.028947	-4.448758
H	0.083958	-0.731506	-3.090438
C	-4.595127	-0.152204	-0.665937
C	-4.595083	0.152868	0.666004
H	-5.536628	0.276051	1.205720
H	-5.536706	-0.275291	-1.205613
H	3.911533	-4.804620	0.919209
H	3.913087	4.803684	-0.918952

22

phen SCF Done: -571.213874682 A.U.

C	-2.834202	0.841696	0.000051
C	-1.419585	0.872567	0.000008
C	-0.730672	-0.379070	-0.000012
N	-1.384166	-1.559318	-0.000069
C	-2.703483	-1.553958	-0.000031
C	-3.485901	-0.376410	-0.000003
H	-3.392754	1.782091	0.000107
H	-3.198643	-2.533464	-0.000148

H	-4.576440	-0.439954	0.000083
C	2.834202	0.841696	-0.000051
C	3.485901	-0.376410	0.000003
C	2.703483	-1.553958	0.000031
N	1.384166	-1.559318	0.000069
C	0.730672	-0.379070	0.000011
C	1.419585	0.872567	-0.000008
H	3.392754	1.782091	-0.000107
H	4.576440	-0.439954	-0.000082
H	3.198643	-2.533464	0.000147
C	-0.681817	2.105198	0.000010
C	0.681817	2.105198	-0.000010
H	-1.238471	3.046408	0.000029
H	1.238471	3.046408	-0.000029

11

py SCF Done: -248.109830850 A.U.

C	-0.000433	1.387242	-0.000025
C	1.200462	0.673227	-0.000362
C	-1.200808	0.672730	0.000346
C	1.143445	-0.725185	0.000789
C	-1.142921	-0.725907	-0.000811
N	0.000308	-1.416994	-0.000010
H	2.165068	1.186330	-0.000905
H	-2.165821	1.185039	0.001045
H	2.069995	-1.312336	-0.000288
H	-2.069342	-1.313337	0.000511
H	-0.000523	2.480615	0.000086

9

ZnNO32 SCF Done: -2339.42003849 A.U.

Zn	0.000000	0.000000	0.000000
O	0.000000	1.081514	1.707700
O	-0.000000	-1.081514	1.707700
N	0.000000	0.000000	2.419854
O	-1.081514	0.000000	-1.707700
O	1.081514	-0.000000	-1.707700
N	0.000000	0.000000	-2.419854
O	0.000000	0.000000	-3.608872
O	-0.000000	-0.000000	3.608872
